# Large-scale rewiring of innate immunity circuitry and microRNA regulation during initial rice blast infection

**DOI:** 10.1038/srep25493

**Published:** 2016-05-06

**Authors:** Ze-Yuan Li, Jing Xia, Zheng Chen, Yang Yu, Quan-Feng Li, Yu-Chan Zhang, Jin-Ping Zhang, Cong-Ying Wang, Xiao-Yuan Zhu, Weixiong Zhang, Yue-Qin Chen

**Affiliations:** 1 State Key Laboratory for Biocontrol, School of Life Science, Sun Yat-Sen University, Guangzhou 510275, P. R. China; 2 Institute for Systems Biology, Jianghan University, Wuhan, Hubei 430056, China; 3 Department of Computer Science and Engineering, Washington University in St. Louis, St. Louis, MO 63130, USA; 4 Plant Protection Research Institute, Guangdong Academy of Agricultural Sciences, Guangzhou, 510640, China.; 5 Department of Genetics, Washington University in St. Louis, St. Louis, MO, 63130, USA

## Abstract

Rice blast is a recurrent fungal disease, and resistance to fungal infection is a complex trait. Therefore, a comprehensive examination of rice transcriptome and its variation during fungal infection is necessary to understand the complex gene regulatory networks. In this study, adopting Next-Generation Sequencing we profiled the transcriptomes and microRNAomes of rice varieties, one susceptible and the other resistant to *M. oryzae*, at multiple time points during the fungal infection. Our results revealed a substantial variation in the plant transcriptome and microRNAome as well as change to rice innate immunity during fungal infection. A number of putative R gene candidates were identified from a perturbed rice transcriptome analysis. The expression of genes and non-coding RNA molecules changed in both fungal resistant and susceptible plants during *M. oryzae* invasion discovered distinct pathways triggered in the susceptible and resistant plants. In addition, a number of fungus genes in the susceptible and resistant plants were constantly expressed at different time points, suggesting that they were likely to be the potential AVR genes. Our results revealed large-scale rewiring of innate immunity circuitry and microRNA regulation during initial rice blast infection, which would help to develop more robust blast-resistant rice plants.

Rice blast, caused by the infection of an ascomycete fungus *Magnaporthe oryzae*, is a recurrent rice disease^1^,^2^, which accounts for 10–30% annual rice yield reduction worldwide. A deep understanding of the disease mechanism of rice blast will undoubtedly help understand disease etiology and progression and design novel strategies for developing durably blast-resistant rice cultivars.

Much effort has been devoted to curtailing this devastating disease. Among such effort are extensive studies of molecular mechanism of pathogen-host interaction, aiming at identifying the genes responsible for resistance to *M. oryzae* infection. Using rice-*M.oryzae* and other model systems, it has been shown that plants in general have evolved an effective innate immunity against pathogen invasion; they mount an immune response through a series of perception of pathogens, signal transduction, and ultimately a hypersensitive cell death to restrain the effect of pathogen invasion^3^,^4^. In the case of *M. oryzae* invasion, the fungus produces effectors through its fungal hyphae to facilitate disease development and to modulate host response. Rice, on the other hand, adopts resistance (R) genes to combat against avirulence (AVR) effectors of *M. oryzae*^5^. All of the currently known R genes, except one, for rice blast belong to the NBS-LRR defense gene family, which consist of cell surface leucine-rich repeats (LRR) and intracellular nucleotide binding sites (NBS) to recognize specific pathogen effectors and trigger resistance responses^6^. Recent results indicate that NBS-LRR defense genes are not only activated upon pathogen infection, but also regulated by small non-coding RNAs, for example, microRNAs (miRNAs)^7^,^8^.

MiRNAs, a class of small noncoding RNAs of ~21-nt in length, are effective posttranscriptional gene regulators. They primarily target protein-coding genes through complementary base pairing, resulting in mRNA degradation or translational repression and thereby regulating many cellular functions and processes, including cell proliferation, differentiation, and apoptosis as well as stress response and pathogen defense^9^. A large number of miRNAs have been identified in diverse plant species^10^,^11^, some of which have been shown to regulate not only programmed development but also physiological processes^8^,^9^,^12^. Notably, several miRNAs are found to be involved in response to pathogen infection via regulating NBS-LRR genes^13^. For instance, nta-miR6019 (22-nt) and nta-miR6020 (21-nt) guide sequence-specific cleavage of transcripts of the TIR-NBS-LRR immune receptor N that confers resistance to tobacco mosaic virus (TMV) in tobacco plant^7^. A super miRNA family miR482 targets NBS-LRR disease resistance genes with coiled-coil domains at their N terminus in *Nicotiana benthamiana*^14^. Furthermore, some miRNAs modulate cleavage of NBS-LRR genes and produce tandem siRNAs arranged in phasing^15^, which are reminiscent of trans-acting siRNAs (ta-siRNAs). A recent study by deep sequencing small RNA libraries from susceptible and resistant lines identified a group of known rice miRNAs differentially expressed upon *M. oryzae* infection^8^,^16^and showed that repression of miRNA biogenesis by silencing OsDCL1 activates the basal resistance to *M. oryzae*, suggesting the importance of miRNA regulation in combating pathogen invasion.

Despite the extensive previous studies, incorporation of resistance genes in rice production, however, has not been as effective as expected in gaining and retaining durable resistance to *M. oryzae*^17^. This disappointing outcome of breeding evidently reveals that our current knowledge of molecular mechanism of fungal infection is far from completion. Importantly, resistance to fungal infection is a complex trait, which requires orchestrated cooperation of a large number of genes as well as their intricate interactions. This observation apparently calls for a comprehensive examination of rice transcriptome and its variation during *M. oryzae* infection. Knowledge of genome-wide response can help understand the complex gene regulatory networks underlying the infection and develop more robust blast-resistant rice plants. In this study, adopting Next-Generation Sequencing (NGS) we profiled the transcriptomes and microRNAomes of two rice varieties, one susceptible and the other resistant to *M. oryzae*, at multiple time points during the first 48 hours of the fungal infection. We observed dramatic variations of the transcriptomes of the host and the pathogen and identified several rice miRNAs that play potential roles during stress response.

## Results

### Distinct transcriptomic responses during rice blast infection

**Figure 1:**
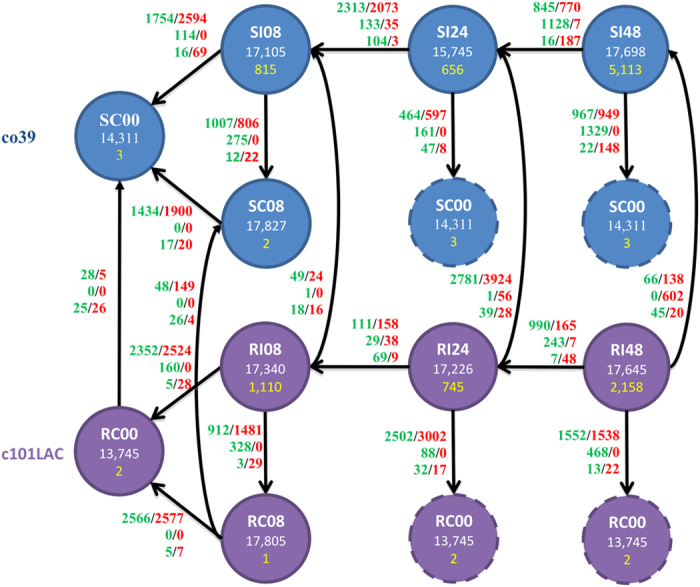
A summary of the transcriptome and microRNAome variations during *M. oryzae* infection. Circle indicates different samples of two rice varieties (blue circle: co39/susceptible and purple circle: c101LAC/resistant). The numbers in each circle indicate the expressed rice genes (in white) and fungus genes (in yellow) respectively, the words above the number are names of samples. There were a total of 19 comparisons; the arrows connecting two circles indicate the DE comparison between two samples (source as control and destination as case). The numbers besides each arrow are the number of DE genes. The first row is the number of the DE rice genes and the second row is the number of the DE fungus genes, followed by the third row representing DE rice miRNAs. Green indicates up-regulated and red indicates down-regulated. In the naming of experiments/samples, S represents susceptible rice plant CO39, R represent resistant rice plant C101LAC; C is control experiment and I represents infection experiment; the numbers represent the time point post inoculation.

In order to investigate variations of rice transcriptome and microRNAome in response to blast infection, we sequenced, profiled, compared and analyzed the gene expressions of two rice cultivars, one susceptible (CO39) and the other resistant (C101LAC) to *M. oryzae*, at different time points after *M. oryzae* infection (Fig. 1). CO39 (*O. sativa* ssp. *indica*) is a rice cultivar that is susceptible to most strains of *M. oryzae*, and C101LAC is a nearly isogenic line of CO39 carrying a single R gene *Pi1*. *Pi1* confers a broad spectrum of resistance to many rice blast isolates in China, and has been recently characterized as an allele at the *Pik* locus^18^ whose function has been determined by two adjacent genes of the NBS-LRR family^15^,^19^,^20^. Plants of the two cultivars were collected at 8, 24 and 48 hours post inoculation (hpi) after being sprayed with blast isolate GD98288 onto the leaves of the two rice cultivars (see Methods) and showing drastically different phenotypes of fungal resistance (Fig. S1). As a control, mock controls were harvested at 0 and 8 hours after being sprayed with water.

Sequencing profiling produced more than 120 million raw reads in all mRNA libraries and more than 183 million raw reads in all small-RNA libraries (Table S2). The processed RNA-seq reads were mapped to the genomes of *O. sativa* ssp. *indica*, together with the genome of *M. oryzae* (see Methods). More than 10 million reads (~93% of the raw reads) can be mapped to the reference genome, and a large number of genes in rice, ranging from 13,000 to 18,000, were detected as expressed at different time points during fungal infection (Fig. 1 and Table S2). As shown, the plants that were fungal infected had more genes expressed than the control at 0, 24, and 48hpi, indicating a widespread gene induction in response to the infection. In addition, in the two normal controls, more genes were expressed at 8 hpi than at 0 hpi.

**Figure 2:**
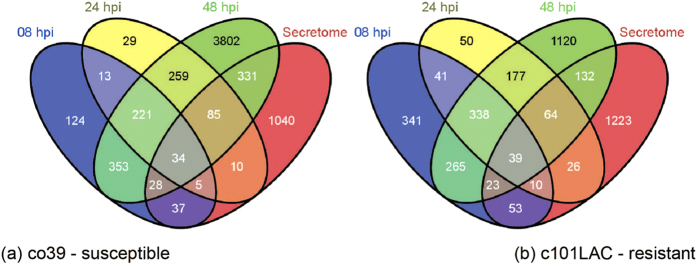
Distribution of expressed fungal genes in (**a**) susceptible and (**b**) resistant plants along the time of infection and secretome. The blue, yellow, and green circles show how many the expressed fungus genes are overlapped at different time points, while the red circle indicates how many of those DE fungus genes are secretome genes, the overlapped parts of secretome genes were expressed at least at one time point, non-overlapping portion were not detected in any time points. A total of 34 and 39 secretory protein genes were expressed in susceptible plant (Co39) and resistant plant(c101LAC),respectively, at 3 time points including 8, 24, and 48 hours after infection.

A substantial number of *M. Oryzae* genes were also detected in the infected plants, but not in the normal control, as expected. Because pathogen-secreted proteins contribute to the majority of known effectors of filamentous fungi^21^, we compared the expressed fungus genes with computational predicted secreted genes. Out of 5,331 and 2,679 fungus genes that were expressed in the fungal-susceptible and resistant plants, 530 and 347 were secreted proteins which expressed at least at one time point in the two cultivars (Fig. 2). Note that 34 and 39 of fungus genes in the susceptible and resistant plants, respectively, were expressed throughout the whole period that we examined including 8, 24, and 48 hours after infection.

### Transcriptome and innate immunity variations in response to fungal infection

*M. Oryzae* produces virulence (AVR) effectors to facilitate its invasion into the host and consequently causes disease development. On the other hand, many AVR effectors are recognized by host resistance (R) proteins that trigger the host’s hypersensitive resistance (HR) responses. To characterize the global transcriptome variations in response to the fungus infection, we first looked for genes that were differentially expressed (DE) across the fungal-susceptible and fungal-resistant rice (Fig. 1). The large number of DE genes across the two rice plants at four different time points that we examined provided insight into the biological functions these genes may have and potential signaling pathways that they may be involved with.

A total of 209 genes were differentially expressed between the susceptible control (SC) and the resistant control (RC) at 0 or 8 hours with no infection. A Gene Ontology (GO) functional analysis revealed that these DE genes were enriched with some of the well-know plant disease related processes, such as phenylpropanoid metabolic process, oxidation-reduction process (FDR < 4.77 × 10^−06^) and fatty acid biosynthetic process. Notably, heme binding (FDR < 4.54 ×× 10^−07^), tetra pyrrole binding (FDR < 4.54 × 10^−07^) and iron ion binding (FDR < 9.28 × 10^−07^) were found enriched (Table S3). This set of *SC vs RC* DE genes between the fungal-susceptible and fungal-resistant rice under the normal conditions represented an intrinsic difference between the two cultivars.

In contrast, more than 6,000 unique rice genes were differentially expressed across the susceptible infected (SI) with resistant infected (RI) rice at 8, 24, or 48 hpi. A GO functional analysis showed that these DE genes were enriched with biological processes such as photosynthesis (FDR < 1.54 × 10^−27^) and response to abiotic stimulus (FDR < 8.05 × 10^−24^) (Table S3). In contrast to the *SC vs RC* DE genes under the condition of no fungal infection, this set of *SI vs RI* DE genes revealed that a substantial number of genes having stress related functions were induced in response to the infection.

Furthermore, the DE genes between the infected plants and the normal controls provided additional information. In order to minimize the effect of interference factors, we only compared gene expressions profiled at the same time of a day. In both fungal-susceptible and fungal-resistant plants, a large number of genes were DE as a result of *M. oryzae* infection, revealing drastic transcriptome perturbations. Particularly, 4,478 and 7,419 unique genes were DE at 8, 24, or 48 hpi in the fungal-susceptible and fungal-resistant rice plants, respectively. A GO enrichment analysis indicated that both sets of DE genes were enriched with similar biological processes, such as photosynthesis, plastid organization, and thylakoid membrane organization, and appeared in the same cellular components, such as plastid, chloroplast, and thylakoid (Table S3). These two sets of DE genes between the infected and control rice plants, referred to as *SI vs SC* and *RI vs RC* for susceptible and resistant rice respectively, suggested the involvement of pathways related to the host defense responses and disease resistance.

### Putative R gene candidates from a perturbed transcriptome analysis

Although the conventional map-based cloning technique has been widely adopted for identification of R genes, only a small number of blast R genes have so far been discovered^22^,^23^. A systematic analysis of the transcriptomes that were perturbed during *M. oryzae* infection provided an opportunity for identifying putative R genes in rice that contribute to resistance to blast virulence.

R genes typically function in a gene-to-gene fashion and the known rice R genes, except one, belong to the family of the NBS-LRR defense gene that contains specific protein domains^6^. We started our search of blast R genes from the genes responded to *M. oryzae* infection. In order to identify rice genes that contribute to the fungal-resistance phenotype, we focused on genes that were differentially expressed across (1) the susceptible and resistant cultivars under the normal condition (i.e., belonging to *SC vs RC*), (2) the susceptible and resistant cultivars during the fungal infection (i.e., belonging to *SI vs RI*), and (3) the fungal infected and in the resistant rice plant with respect to the normal controls (i.e., belonging to *RI vs RC*). This resulted in 79 unique genes (Table S4).

**Figure 3:**
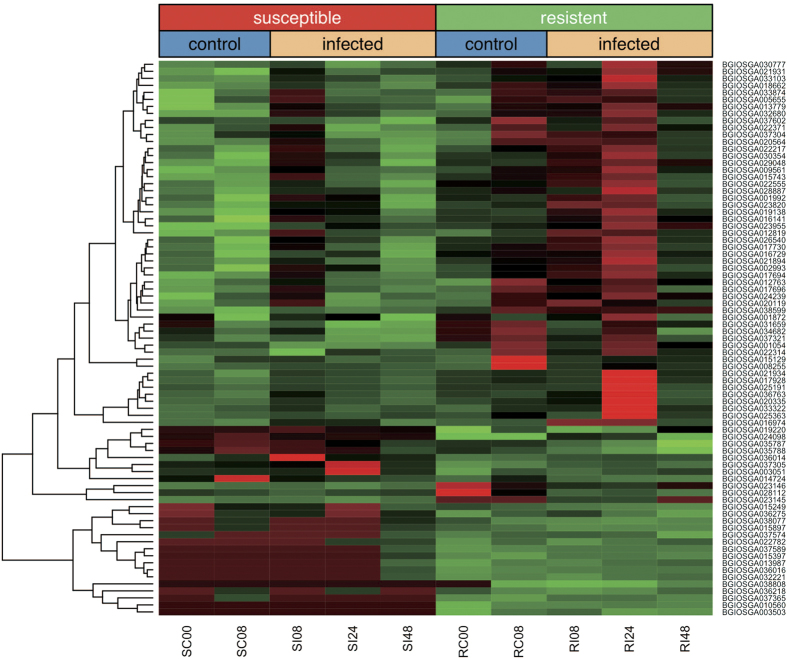
A heatmap of differentially expressed 79 mRNA genes in Resistant v.s. Susceptible rice at 0, 8, 24, and 48 hours after infection, these 79 candidate rice genes differentially expressed in the two rice plants across all conditions including *SC vs RC, SI vs RI* and *RI vs RC*. S represents susceptible rice plant CO39, R represent resistantrice plant C101LAC; C is control experiment and I represents infection experiment; Heat colours reflect the expression amount of genes (light green: fewer - dark red: more).

The expression patterns of the 79 candidate genes in the two rice plants across all conditions are shown in Fig. 3. Under the normal growth condition with no fungal inoculation, 55 (69.6%) and 2 (2.5%) out of the 79 genes were induced in the fungal-susceptible rice plant in reference to the resistant plant at the 8 and 0 hour (i.e. SC vs RC), respectively. Under infection, in comparison, only 3 (5.5%) out of the 55 genes induced in the SC vs RC were significantly up-regulated in the resistant plant with respect to the susceptible cultivar at 8 hpi, but as infection progresses, 53(96.4%) out of the 55 genes were up-regulated comparing the resistant with the susceptible cultivars at 24 hpi. This suggested that this set of induced genes were similarly invoked at the beginning of fungal infection in both susceptible and resistant rice. However, at a late stage of 24 hpi, these genes may contribute to fungal resistance by maintaining their relative higher expression levels in the fungal-resistant plant, as they were in the normal control at 8 hours. A GO enrichment analysis on the 79 candidate fungal-resistant genes revealed that the three most significantly enriched molecular functions were transferase activity (GO: 0016757, FDR < 0.0015), iron ion binding (GO:0005506, FDR < 0.0015), and heme binding (GO:0020037, FDR < 0.0015), and the most significantly enriched biological processes included oxidation-reduction process (GO:0055114, FDR < 0.049) and fatty acid biosynthetic process (GO:0006633, FDR < 0.049). Iron ion has recently been considered as a nutritional immunity and Fe limitation as a universal strategy in innate defense^24^, and the modes of heme binding and substrate access for cytochrome P450 CYP74A are revealed to be important for plant defense^25^. These findings suggest metal homeostasis plays important roles at the pathogen–host interface and metal ion binding molecules might be novel kind of R gene candidates.

Importantly, among the 79 candidate genes analyzed, BGIOSGA036218 and BGIOSGA038808 contain the NB-ARC domain, which is known to be essential for most known R genes^26^. BGIOSGA038808 was also annotated to code a known R gene, PIKM2. In addition, BGIOSA021931 and BIOSA021934 were annotated to be plant disease response proteins (IPR004265). Taken together, these results suggested that this set of differentially expressed genes were excellent candidates for R genes to combat *M. oryzae* virulence.

### Perturbed microRNAome in response to blast infection

Small non-coding RNAs, particularly miRNAs, have been recognized as important for plant immune responses against viruses, bacteria, oomycete, and fungi^8^,^27^,^28^,^29^,^30^. In order to appreciate their regulatory functions, we analyzed perturbed microRNAome in response to blast infection. Among the known rice miRNAs (miRBase version21), five miRNA families (miR156, miR159, miR166, miR167 and miR168) were highly expressed as detected by the sequencing based small RNA profiling. These five miRNA families contributed to 88.9% of the total sequence reads mapped to the annotated miRNA hairpins (Table S5). Interestingly, a recently reported miRNA, miR5794, was highly expressed (278 K reads), which was next to the five most abundant miRNA families. We also searched for novel rice miRNAs utilizing the sequencing data and identified thirteen new miRNAs (Table S6) based on a set of stringent criteria (see Methods). An example novel miRNA with its hairpin structure and alignment of corresponding sequencing reads is shown in Fig. S2.

**Figure 4:**
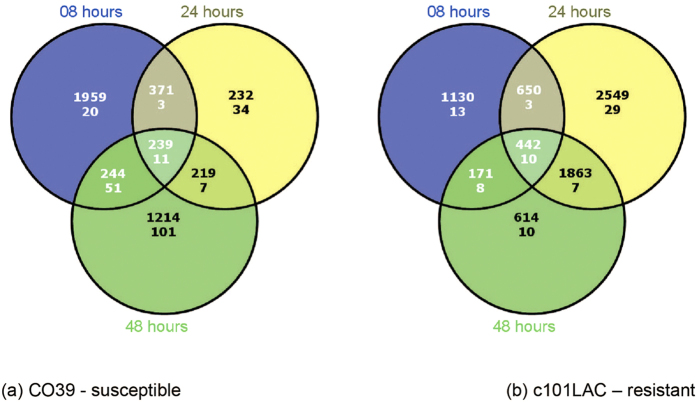
Distributions of differential expressed rice mRNA genes (first row) and miRNAs (second row) in Infection v.s. Control libraries at 8, 24, and 48 hours after infection. Blue circles represent *SI* 08 *vs SC* 08 and *RI* 08 *vs RC* 08, Yellow circles represent *SI* 24 *vs SC* 00 and *RI* 24 *vs RC* 00, Green circles represent *SI* 48 *vs SC* 00 and *RI* 48 *vs RC* 00. Three comparisons overlapped in susceptible plant (Co39) and resistant plant (c101LAC), there were 239 DE rice miRNA genes and 11 DE miRNAs in all 3 comparisons in susceptible plant, and there were 442 DE rice miRNA genes and 10 DE miRNAs in all 3 comparisons in resistant plant.

A substantial number of miRNAs were DE under blast infection (Figs 1 and 4). Overall, 330 expressed miRNAs, including 320 known and 10 novel miRNAs, were DE by at least 2-fold in one of the 19 comparisons between different time points or across the two rice cultivars. Interestingly, significantly more miRNAs were DE in the blast-susceptible rice than in the blast-resistant rice after 48-hpi. Specifically, 169 miRNAs were DE in the disease-susceptible cultivar after 48-hpi, while only 33 miRNAs were DE in the disease-resistant cultivar, with respect to their respective controls. Consistently, 203 DE miRNAs were observed between 48-hpi and 24-hpi for the fungal-susceptible rice versus 55 DE miRNAs in the same comparison for the fungal-resistant rice (Figs 1 and 4). Taken together, a more perturbed microRNAome appeared in the disease-susceptible cultivar than in the disease-resistant cultivar and the DE miRNAs may in part contribute to the phenotypic difference in response to blast infection.

We experimentally validated the expression of 10 DE miRNAs, including 8 known (miR156a, miR397b, miR399a/d/e, miR528, miR530-5p, miR827a, miR1318, and miR1320) and 2 novel (novel-1 and 2) miRNAs, using real-time quantitative RT-PCR (qRT-PCR). The results showed an excellent concordance between the sequencing based profiling and qRT-PCR results of most of the miRNAs tested (Fig. S3), supporting the observation that these miRNAs had substantially different expressions between the blast-resistant and blast-susceptible cultivars during the fungal infection.

### MicroRNAs are regulators of transcriptome variations and innate immunity

Differentially expressed miRNAs have the potential to target mRNA transcripts in rice and fungus to exert their functions that may ultimately lead to phenotypic variation. In order to appreciate their potential regulatory roles, we searched for putative miRNA targets in both rice and fungus (see Methods). In particular, those target genes that exhibited differential expression that was anti-correlated with differential expression of targeting miRNAs were identified for further analysis. This analysis resulted in 213 pairs of anti-correlated DE fungus genes and DE rice miRNAs (Table S7) and 789 pairs of anti-correlated genes and miRNAs in rice (Table S8).

**Figure 5:**
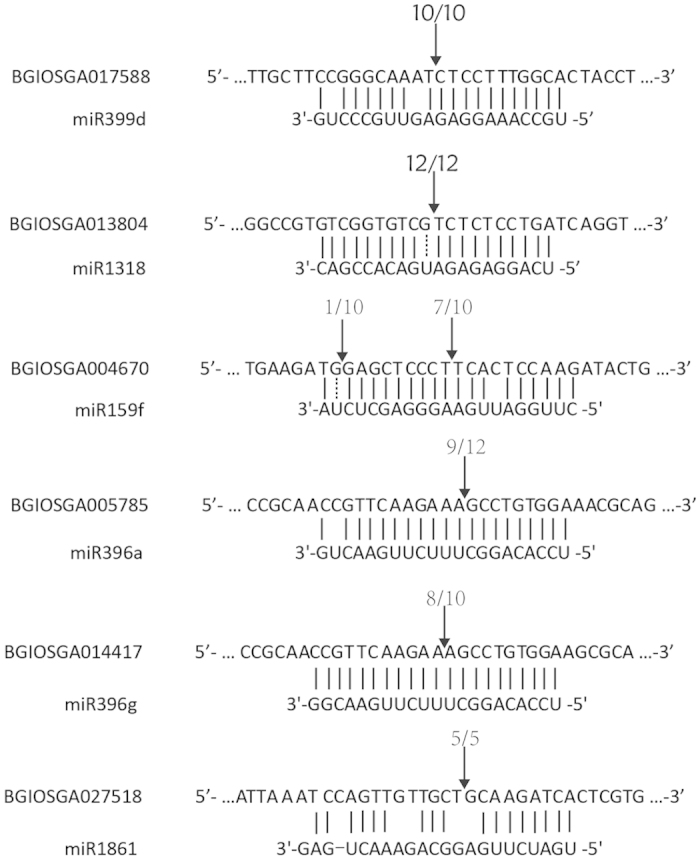
Experimental validation of some selected miRNA/target pairs and miRNA cleavage sites. Six pairs of complementary sequence of miRNAs and their target genes were shown, the first row is the sequence of target genes, the second row is the sequence of miRNAs. Watson-Crick pairing (vertical bar), G:U wobble pairing (imaginary line) and mismatched bases pairing (blank space) are indicated. Arrows indicated the cleavage sites validation by 5′ RLM-RACE, numbers indicate the fraction of cloned PCR products terminating at different positions.

It has been evidenced that some plant miRNAs target only one gene, while some might target to several genes. We selected two miRNAs, miR399 and miR1318 for validation of their potential targets. miR399 and miR1318 are the two most differentially expressed miRNAs during the infection. miR1318 is a rice specific miRNA, its targets had never tested before. According to PMRD (plant microRNA database), several genes were predicted to be the targets of miR1318. Thus we employed RLM-RACE to test all the possible targets. However, only BGIOSGA013804 (a calcium-binding allergen Ole e 8) was experimentally validated as the target of miR1318 (Fig. 5). miR399 had been reported to target several genes, including *GmPHO2* (*Glyma13g31290*) and *GmPT5* (*Glyma10g04230*) in soybean^31^, *HvPHO2* in barley^32^, PHO2/UBC24 in Arabidopsis^33^. In rice, 7 targets of miRNA399 were predicted by psRNATarget, and we validated one of targets was BGIOSGA017588, an ubiquitin conjugating enzyme, indicating that miRNAs were the regulators of the ubiquitin conjugating enzyme or calcium-binding allergens, which was reported to play important roles in plant immunity^34^,^35^. Furthermore, we validated several other miRNAs and their potential targets by RLM-RACE, which showed negatively correlated expressed patterns in several conditions in the blast-susceptible and resistant cultivar (Fig.5 and Table S8). For example, BGIOSGA004670, the homolog of GAMYB in Arabidopsis, a target of miR159f, was up-regulated in response to fungal infection, while miR159f was down-regulated in several conditions in the blast-susceptible cultivar. These results suggested that GAMYB-like genes in rice might be actively involved in the defense of fungal infection via the regulation of miR159 in rice. More interestingly, a glyoxalase gene (BGIOSGA027518) was predicted and validated as the target of miR1861, a rice specific miRNA. It was reported that miR1861 is responded to arsenate and arsenite stress^36^, and glyoxalase is also reported to be associated with biotic and abiotic stimulation in *A. thaliana*^37^,^38^. These observations suggested that miR1861 and its mediated glyoxalase pathway could play a role in innate immunity.

## Discussion

The ability of a plant to recognize the presence of a pathogen and mount an effective immune response is fundamental to survival. On the other hand, the pathogen can also manipulate the physiological process of the plant to establish a suitable environment for its growth^39^. Recent development of molecular biology and bioinformatics technology has made it possible to better understand the molecular mechanism during plant-pathogen interaction and provide the possibility to improve plant disease resistance through genetic engineering. In this study, we applied Next-Gen Sequencing (NGS) to investigate the variations of rice transcriptome and microRNAome during the process of blast infection and to explore host and fungi symbiosis. Our results revealed a substantial variation in the plant transcriptome and microRNAome as well as change to rice innate immunity during fungal infection. The expression of genes and non-coding RNA molecules changed in both fungal resistant and susceptible plants during *M. oryzae* invasion indicated that distinct pathways were triggered in the susceptible and resistant plants. In parallel, the fungi also altered its gene expression during infection, suggesting that the plant and the fungi interacted and responded to each other via various molecular mechanisms.

The complexity of the miRNA regulation in plant innate immunity has been uncovered in recent studies, showing that miRNAs play a critical role in disease resistance responses^8^,^27^,^28^,^40^,^41^,^42^. Particularly, several studies have shown bacterial elicitor flg22 infection can induce accumulation of miR393, which has a well-studied target gene, F-box auxin receptor. Suppressing auxin signaling by miR393 positively contributes to pathogen associated molecular pattern (PAMP)-triggered immunity (PTI)^27^. In *Pinus taeda*, the expression of 10 miRNA (pta-miRNA) families was significantly repressed in the fusiform rust fungus (*Cronartium quercuum*) infected galled stem compared to healthy stem^43^. In addition, the expression of ~82 plant disease-related transcripts was detected to be altered in response to miRNA regulation in pine^43^.

Furthermore, the recent results suggest miRNA regulation of innate immunity to be a new mechanism of immune response in plants. In particular, a recent study shows that several miRNA families target NBS-LRR plant innate immune receptors in legumes^13^ and Solanaceae^7^. An earlier study also predicts miRNA families targeting TIR-NBS-LRR class genes, induced in Turnip mosaic virus infected plants in Brassica^44^. Carrying miRNA target sites in genes could allow miRNAs to fine tune the expression of NBS-LRR genes through altered expression of miRNAs. Li *et al.*^8^ find that a set of miRNAs is involved in immunity against the blast fungus *M.oryzae*^8^. In this study, through analyses of transcriptome and microRNAome, we also revealed a genome-wide variation of microRNAome in response to blast infection and that many miRNAs are regulators of transcriptome variation and innate immunity by targeting to both rice and fungus genes. These findings help gain new insights into miRNA functions in plant innate immunity.

The NBS-LRR immune receptors play central roles against pathogen infection in plants. Although some R genes and AVR genes have been found and identified by traditional cloning techniques, only a small number of blast R genes have been identified so far^22^,^23^. For example, a total of nine AVR genes in *M. oryzae* and 13 resistance (R) genes in rice have been cloned^45^. Notably, R genes usually perform their function in the gene-to-gene manner, and the pathogen evolves rapidly and the corresponding AVR gene is easy to lose avirulence. Therefore, a systematic identification of R genes is necessary. In the current study, 79 putative R genes that are associated with the expressed, secreted genes were found. Interestingly, two of them contain the NB-ARC domain, which is known to be essential in many known R genes^26^. In addition, our results also showed that a number of fungus genes in the susceptible and resistant plants were constantly expressed at different time points during infection, suggesting that they were likely to be the potential AVR genes. Further studies are necessary to experimentally validate these putative R genes and their interaction with miRNA regulation.

## Materials and Methods

### Rice plants and fungal infection

The rice seeds were sown in 30 cm × 20 cm × 5 cm trays in a green house, in seven rows and each row with 15–20 plants. Inoculation with blast spore was performed at the 3.5 leaf stage. A 20-ml spore suspension (10^5^ spores/ml) was applied to each tray using an airbrush connected to a source of compressed air. The plants were then held in dark for 24 h at 95–100% relative humidity and 28 °C, after which they were transferred to a green house where the ambient temperature was maintained at 28 °C. The fully expanded third leaf, which would show most severe symptom, was collected from 25 individual seedlings as one sample at each time point. Samples were collected at 8, 24 and 48 hours post inoculation, control samples were collected at 0 and 8 hours after being sprayed with water. Each sample was collected in duplicate; one of the duplicates was used for RNA-seq, another one for experiment validation.

### RNA extraction and RNA-Seq

Total RNA was extracted with TRIzol (Invitrogen, USA) from each sample according to the manufacturer’s protocol. NanoDrop ND-1000 spectrophotometer (NanoDrop, USA) was used to quantify the concentration of total RNA.RNA-seq was performed via HiSeq 2000(Illumina, USA) at Beijing Genome Institute (BGI) (Shenzhen, CHN). Briefly, oligo(dT) magnetic beads was employed to enrich mRNA and then interrupted mRNA to short fragments (about 200 bp), the mRNA fragments was treated as templates to synthesize the first and second strand cDNA. Following by ligating sequencing adaptors to the fragments, the fragments were purified by agarose gel electrophoresis and enriched by PCR amplification. HiSeq 2000 was used to sequence the library products. The sequencing data have been submitted to public database, the NCBI’s GEO database, the accession number is GSE77333.

### Small RNA extraction and Sequencing

Small RNA libraries were constructed using previously described methods^46^. Briefly, 10–30 nt small RNAs were purified using a 15% denaturing polyacrylamide gel, then ligated with 5′ and 3′ adapters, then converted to cDNA by RT-PCR using Superscript II reverse transcriptase (Invitrogen), small RNAs were amplified by PCR, gel-purified then submitted for deep sequencing using the Illumina Genome analyzer (Illumina) at the Beijing Genomics Institute (BGI, Shenzhen, China). All small RNA sequences have been submitted to public database, the NCBI’s GEO database, the accession number is GSE77333.

### Identification of novel miRNAs

We identified novel miRNAs by applying the previously described method to the rice genome^47^. Briefly, four criteria were adopted for calling a candidate miRNA: 1) occurrence of miRNA reads on the arms of predicted hairpin structures; 2) presence of no less than 10 miRNA reads of the highest frequency on predicted hairpins; 3) presence of possible miRNA* sequencing reads unless specifically stated (see below), and 4) presence of 2–3 nt 3′ overhangs on miRNA/miRNA* duplexes. Each candidate miRNA was visually reviewed. The read with the highest read count, cumulatively from all small-RNA libraries, was preferentially selected as the mature miRNA sequence.

The expression of a miRNA was quantified following the normalization and miRNA expression analysis method described previously^48^.

### Target gene prediction and validation by RLM-5′RACE

The Target Finder program release 1.6 (http://jcclab.science.oregonstate.edu/node/view/56334) was used to query all known and novel rice miRNA sequences against all annotated rice transcript sequences from MSU7 gene annotation and BGI Rise Genome Database with the cutoff alignment score of 5. The resulting miRNAs targets predictions were filtered with a cutoff score of 4. Oligotex mRNA kit(QIAGEN, GER) was used to enrich mRNA from total RNA, following the manufacturer’s instructions. The quality of the mRNA was checked on a NanoDrop ND-1000 spectrophotometer (NanoDrop, USA).To confirm the predicted miRNA targets, RLM-RACE (5′ RNA ligase mediated rapid amplification of cDNA ends) was performed using the FirstChoice RLM-RACE Kit (Ambion, USA) according to the manufacturer’s instructions. The primers of RLM-RACE were shown in Table S1. APrep DNA Gel Extraction Kit was used to purify RLM-RACE products (Axygen, USA), and then the purified products were ligated into the pEASY-T3 vector (TransGen Biotech, CHN) and sequenced.

### Read alignment and quantification

To simultaneously measure the expression of genes in *O. sativa* and identify whether any fungal genes were expressed in the *M. oryzae* infected leaf tissues, we aligned short reads against genome sequences and annotations from both organisms as the reference. The *O. sativa indica* genome and annotations were retrieved from the Ensemble Plants Repository Release 8^49^. For *M. oryzae*, sequences and annotations of supercontigs of version 8 were downloaded from the Magnaporthe comparative Database^50^. All reads mapping was carried out using TopHat 2.0.11^51^. HTSeq 0.6.1p2^52^ with intersection-nonempty option was adopted to count the number of reads in each gene given the alignment results of Tophat. Differentially expressed (DE) transcripts were then identified using edger^53^, a count-based differential expression test tool based on Negative Binomial Distribution, with a False Discovery Rate (FDR) no more than1%. This tool could support the analysis of no replicate libraries^53^,^54^. The DE test is performed on rice and fungal genes altogether. Expressed genes with low abundance were filtered out and only genes that have at least one count per million in at least one sample were kept for further analysis. We conducted pair-wise comparisons to track changes in transcripts expression of different treatments, plant cultivars, and along the time course. A list of all comparisons performed is shown in Fig. 1.

### Function enrichment analysis

For differentially expressed *M. oryzae* genes, Functional Annotation Chart in Database for Annotation, Visualization and Integrated Discovery (DAVID) was used to identify overrepresented functional annotations. Each annotation was assigned with a *p*-value to examine the significance of gene-term enrichment with modified Fisher’s exact test. A False Discovery Rate was also calculated as a multiple testing correction^55^,^56^. The GO annotation from the Ensembl Plant Biomart database^57^ was used to analyze differentially expressed *O. sativa indica* genes. The statistical significance of GO term enrichment was measured by Fisher’s exact test. False Discovery Rate based on Benjamini-Hochberg multiple testing correction^58^ was computed for statistical significance.

### qRT-PCR

Total RNAs were reverse transcribed using the PrimeScript RT reagent kit (Takara, Japan), according to the manufacturer’s protocol. All the qRT-PCR reactions were performed in 96-well plates on a QuantStudioTM 6 Flex Real-Time PCR Systerm (Applied biosystem, USA),using SYBR Premix Ex Taq (Takara, Japan). 5.8 s rRNA was chosen as the internal reference gene. The real-time PCR was performed using the SYBR Premix Ex Taq II kit following the manufacturer’s protocol (Takara, Japan). The reactions were amplified for 1 min at 95 °C, and then followed by 40 cycles of 95 °C for 10 s and 60 °C for 30 s. The real-time PCR primers of miRNAs were shown in Table S1. The comparative Ct method was used to quantify miRNA expression. All experiments were done in triplicate, and the results were represented in mean ± standard deviation (s.d.).

## Additional Information

**How to cite this article**: Li, Z.-Y. *et al.* Large-scale rewiring of innate immunity circuitry and microRNA regulation during initial rice blast infection. *Sci. Rep.* **6**, 25493; doi: 10.1038/srep25493 (2016).

## Supplementary Material

Supplementary Information
