# Supplementary material for: Large-scale rewiring of innate immunity circuitry and microRNA regulation during initial rice blast infection
Source: Sci Rep. 2016 May 6;6:25493. doi: 10.1038/srep25493 (PMC4858701; doi:10.1038/srep25493)
Supplement: Supplementary Information [file srep25493-s1.pdf]

# Supplementary Information

**Title: Large-scale Rewiring of Innate Immunity Circuitry and MicroRNA Regulation During Initial Rice Blast Infection**

**Authors:** Ze-Yuan Li[1&],Jing Xia[2,3&], Zheng Chen[2,3] ,Yang Yu[1], Quan-Feng Li[1], Yu-Chan Zhang, Jin-Ping Zhang[1], Cong-Ying Wang[4], Xiao-Yuan Zhu[4], Weixiong Zhang[2,3,5]* and Yue-Qin Chen[1]*

&ZYL and JX contributed equally to this work.

**Affiliation:**

1. State Key Laboratory for Biocontrol, School of Life Science, Sun Yat-Sen University, Guangzhou 510275, P. R. China
2. Institute forSystems Biology, Jianghan University, Wuhan, Hubei 430056, China;
3. Department of Computer Science and Engineering, Washington University in St. Louis, St. Louis, MO 63130, USA;
4. Plant ProtectionResearchInstitute GuangdongAcademy of AgriculturalScience
5. Department of Genetics, Washington University in St. Louis, St. Louis, MO, 63130, USA.

***Corresponding authors:**

School of Life Science, Sun Yat-Sen University, Guangzhou 510275, P. R. China, Phone: 86-20-84112739, Fax: 86-20-84036551; Email: lsscyq@mail.sysu.edu.cn to Y.-Q. Chen

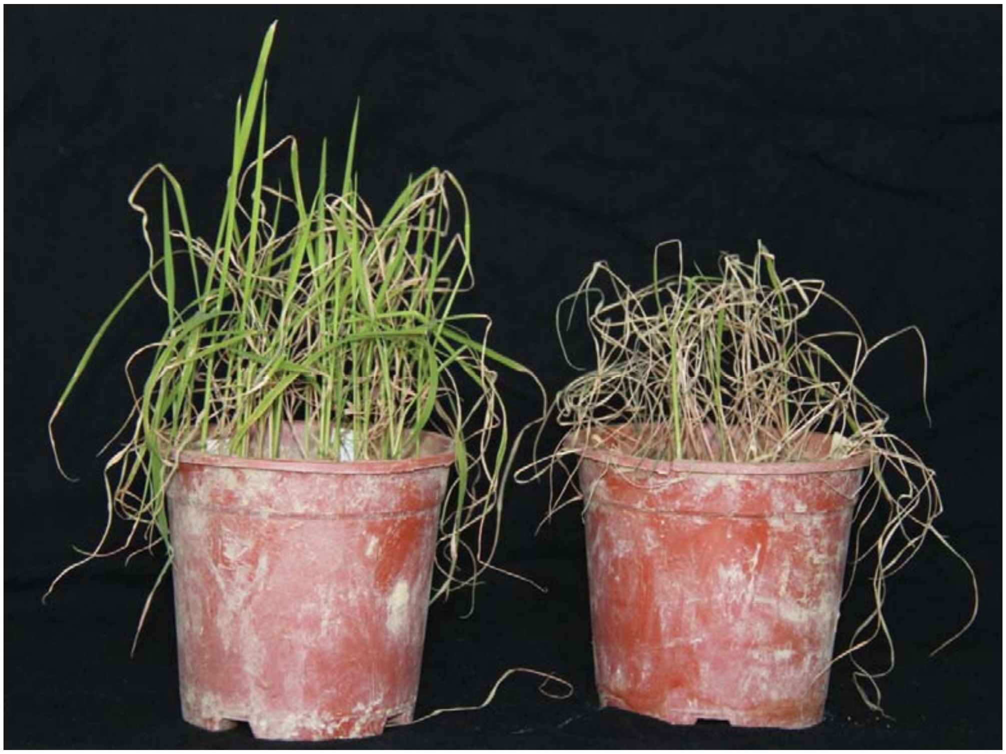

**Supplemental Figure S1**. Phenotypes of two rice cultivars in response to infection of a broadly virulent blast isolate GD98288. Grown in the pot to the left is a blast-resistant cultivar C101LAC and to the right a blast-susceptible cultivar CO39. Infection was administrated on rice seedlings by spray of GD98288 and the photos were taken 10 days post inoculation.

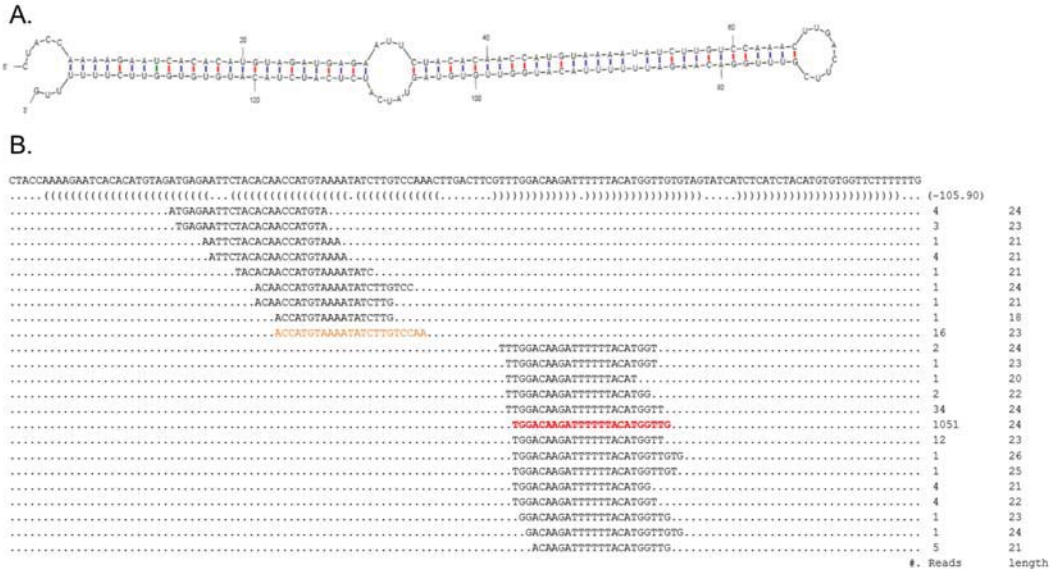

**Supplemental Figure S2**. An example of a novel miRNA (novel-1). (A) Hairpin structures of novel-1 the miRNAs annotated in red and yellow on both arms. (B) Alignment of sequencing reads to the hairpin of nove-1 with the mature miRNA sequence (most abundant read colored in red) and miRNA* (yellow).

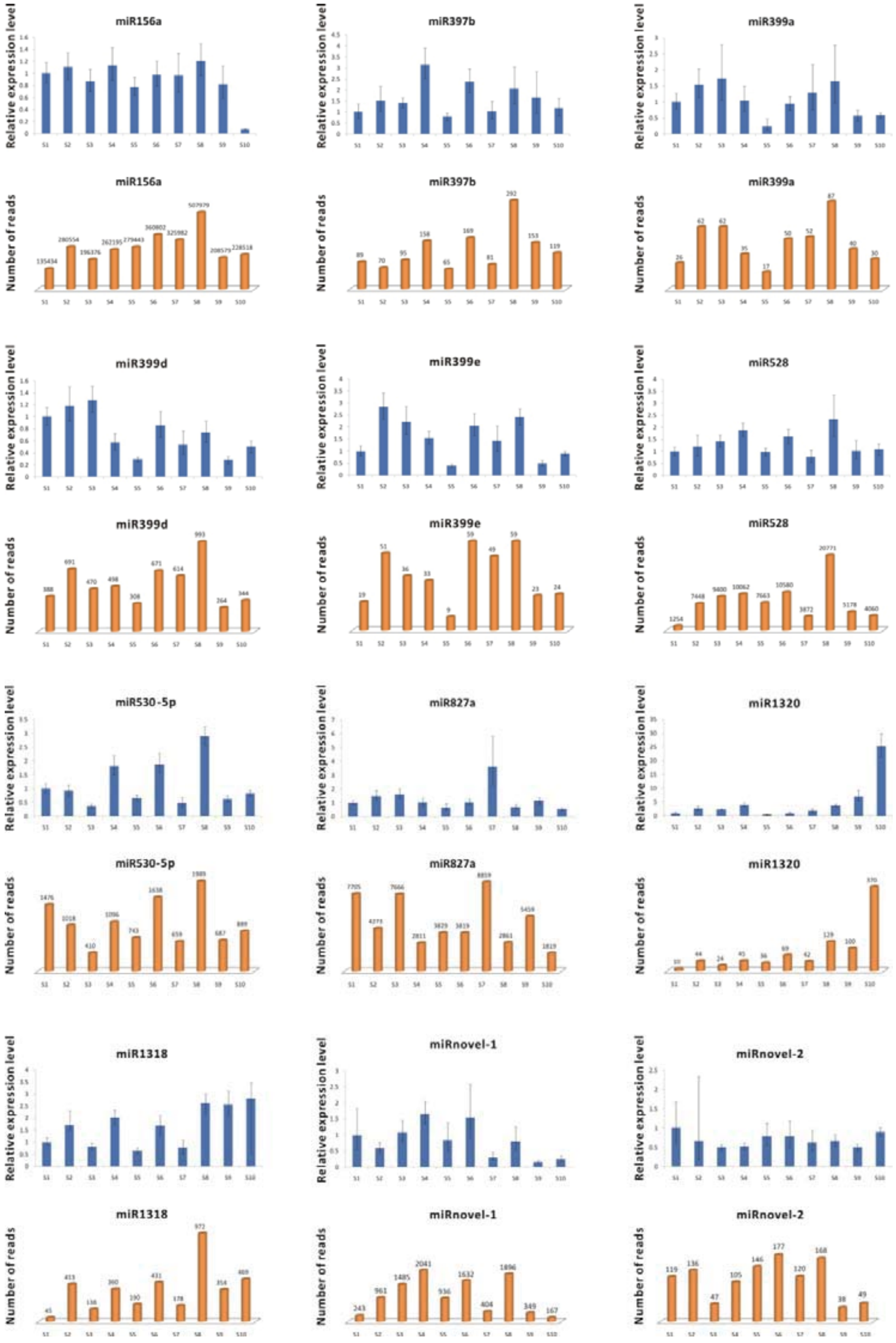


**Supplemental Figure S3**. Experimental validation of the differentially expressed miRNAs using real-time quantitative RT-PCR (qRT-PCR). The number of reads of each miRNA was used to visualize the miRNA expression patterns in the sequencing data.

Table S1. Primers used for qRT-PCR.

| Primer Name | Primer sequences |
|---|---|
| qRT-156a-F | gcggcggTGACAGAAGAGAGT |
| qRT-159a.2-F | gcgcTTGCATGCCCCAGGA |
| qRT-160a-F | cgccTGCCTGGCTCCCTGT |
| qRT-1846a-5p-F | ttcctAGTGAGGAGGCCGGG |
| qRT-2876-F | gcggcggTTCCTATATGAACAC |
| qRT-394-F | gcggcggTTGGCATTCTGTCC |
| qRT-397b-F | cggcggTTATTGAGTGCAGCG |
| qRT-399a-F | gcggcggTGCCAAAGGAGAATT |
| qRT-399d-F | gcggcggTGCCAAAGGAGAGTT |
| qRT-399e-F | gcggcggTGCCAAAGGAGATTT |
| qRT-528-F | gcgcgTGGAAGGGGCATGCA |
| qRT-529b-F | gcggcggAGAAGAGAGAGAGTA |
| qRT-530-5p-F | gcggcgTGCATTTGCACCTG |
| qRT-827a-F | gcggcggTTAGATGACCATCAG |
| qRT-172a-F | gcggcggAGAAtCttGAtGAtG |
| qRT-1318-RT | GTCGTATCCAGTGCAGGGTCCGAGGTATTCGCACTGGATACGACgtcggt |
| qRT-1320-RT | GTCGTATCCAGTGCAGGGTCCGAGGTATTCGCACTGGATACGACctataa |
| qRT-156a-RT | GTCGTATCCAGTGCAGGGTCCGAGGTATTCGCACTGGATACGACgtgctc |
| qRT-159a.2-RT | GTCGTATCCAGTGCAGGGTCCGAGGTATTCGCACTGGATACGACtgcagc |
| qRT-160a-RT | GTCGTATCCAGTGCAGGGTCCGAGGTATTCGCACTGGATACGACtggcat |
| qRT-1846a-5p-RT | GTCGTATCCAGTGCAGGGTCCGAGGTATTCGCACTGGATACGACagcggc |
| qRT-2876-RT | GTCGTATCCAGTGCAGGGTCCGAGGTATTCGCACTGGATACGACgcaaca |
| qRT-394-RT | GTCGTATCCAGTGCAGGGTCCGAGGTATTCGCACTGGATACGACggaggt |
| qRT-397b-RT | GTCGTATCCAGTGCAGGGTCCGAGGTATTCGCACTGGATACGACcatcaa |
| qRT-399a-RT | GTCGTATCCAGTGCAGGGTCCGAGGTATTCGCACTGGATACGACcagggc |
| qRT-399d-RT | GTCGTATCCAGTGCAGGGTCCGAGGTATTCGCACTGGATACGACcagggc |
| qRT-399e-RT | GTCGTATCCAGTGCAGGGTCCGAGGTATTCGCACTGGATACGACctgggc |
| qRT-528-RT | GTCGTATCCAGTGCAGGGTCCGAGGTATTCGCACTGGATACGACctcctc |
| qRT-529b-RT | GTCGTATCCAGTGCAGGGTCCGAGGTATTCGCACTGGATACGACaagctg |
| qRT-530-5p-RT | GTCGTATCCAGTGCAGGGTCCGAGGTATTCGCACTGGATACGACtaggtg |
| qRT-827a-RT | GTCGTATCCAGTGCAGGGTCCGAGGTATTCGCACTGGATACGACtgtttg |
| qRT-172a-RT | GTCGTATCCAGTGCAGGGTCCGAGGTATTCGCACTGGATACGACatgcag |
| Universal Rverse primer | CCAGTGCAGGGTCCGAGGT |

Primers used for RLM-PCR.

| Primer Name | Primer sequences |
|---|---|
| 5' RACE outer primer | GCTGATGGCGATGAATGAACACTG |
| BGIOSGA017588 outer primer | TTTCCAAGTCAACAAGCA |
| BGIOSGA013804 outer primer | CCTCCGCATCACGTTCCACAG |
| BGIOSGA004670 outer primer | TGCTCCACGAGGAAGAATTGAACA |
| BGIOSGA005785 outer primer | CCACTCATCAAAGAAGGGACGC |
| BGIOSGA014417 outer primer | GTCTGGTTCTCCTGCTTCCCG |
| BGIOSGA027518 outer primer | GGAAGTCGGCGTTATCAACCAAA |
| 5' RACE inner primer | CGCGGATCCGAACACTGCGTTTGCTGGCTTTGATG |
| BGIOSGA017588 inner primer | TTCTTCTTGGTCTTCTTCAC |
| BGIOSGA013804 inner primer | GCTCGCATTCCCGCCTCA |
| BGIOSGA004670 inner primer | ATGAGGTGAAGTGTCCCCAGGTT |
| BGIOSGA005785 inner primer | GCGTCTGGTTCTCTTGCTTCACA |
| BGIOSGA014417 inner primer | GTCACATAGTCGCTCCCGAAGAA |
| BGIOSGA027518 inner primer | CACTTTCCAGCCATCAGGGTCAA |

Table S2. Statistics of sequencing data and alignment against Indica group and blast fungus.

Table S2A. RNA-Seq

| Library | Total Reads per library | Mapped Reads | Percentage | Reads mapped to multiple location | Percentage |
|---|---|---|---|---|---|
| CO39 control 0 hour (SC00) | 11,737,877 | 10,939,014 | 93.19% | 1,293,757 | 11.02% |
| C101LAC control 0 hour (RC00) | 11,872,207 | 11,193,079 | 94.28% | 1,310,117 | 11.04% |
| CO39 8 hpi (SI08) | 11,697,095 | 10,990,503 | 93.96% | 1,145,668 | 9.79% |
| C101LAC 8 hpi (RI08) | 12,300,002 | 11,615,102 | 94.43% | 1,040,818 | 8.46% |
| CO39 control 8 hour (SC08) | 12,375,338 | 11,778,050 | 95.17% | 1,263,339 | 10.21% |
| C101LAC control 8 hour (RC08) | 12,041,649 | 11,479,131 | 95.33% | 1,257,336 | 10.44% |
| CO39 24 hpi (SI24) | 11,995,402 | 11,307,197 | 94.26% | 1,384,227 | 11.54% |
| C101LAC 24 hpi (RI24) | 12,253,337 | 11,715,033 | 95.61% | 1,022,605 | 8.35% |
| CO39 48 hpi (SI48) | 12,339,628 | 11,758,300 | 95.29% | 1,137,125 | 9.22% |
| C101LAC 48 hpi (RI48) | 12,376,207 | 10,529,076 | 85.08% | 985,139 | 7.96% |
| Total Reads of all libraries | 120,988,742 | | | | |

Table S2B.Small RNA-seq

| Library | Total Reads per library | Mapped Reads | Percentage | Reads mappedtomultiple location | Percentage |
|---|---|---|---|---|---|
| CO39 control 0 hour (SC00) | 19,844,062 | 17,639,775 | 88.89% | 14,780,769 | 83.79% |
| C101LAC control 0 hour (RC00) | 18,041,629 | 16,364,984 | 90.71% | 13,295,218 | 81.24% |
| CO39 8 hpi (SI08) | 15,840,093 | 13,692,898 | 86.44% | 11,181,843 | 81.66% |
| C101LAC 8 hpi (RI08) | 17,512,544 | 15,015,109 | 85.74% | 13,154,851 | 87.61% |
| CO39 control 8 hour (SC08) | 19,564,471 | 17,725,524 | 90.60% | 15,347,583 | 86.58% |
| C101LAC control 8 hour (RC08) | 18,709,513 | 16,823,420 | 89.92% | 14,021,347 | 83.34% |
| CO39 24 hpi (SI24) | 15,760,954 | 12,598,797 | 79.94% | 9,811,148 | 77.87% |
| C101LAC 24 hpi (RI24) | 17,734,294 | 13,526,766 | 76.27% | 10,479,987 | 77.48% |
| CO39 48 hpi (SI48) | 20,650,577 | 11,429,751 | 55.35% | 8,109,109 | 70.95% |
| C101LAC 48 hpi (RI48) | 19,718,337 | 8,487,961 | 43.05% | 6,030,845 | 71.05% |
| Total Reads of all libraries | 183,376,474 | | | | |

Two kinds of RNA-Seq libraries were constructed including mRNA and small RNA. Library represent samples at different time points. Total Reads per library is all reads in each library. Mapped Reads represent reads which could map to the genome of Indica group and blast fungus. The first “Percentage” is ratio of mapped reads in total reads, Reads mapped to multiple location represent reads which could map to more than one sites on the genome of Indica group and blast fungus. And the second “Percentage” is the ratio of multiple location reads in total reads.

| **Table S3. Significantly enriched (FDR<0.01 or top-50) GO Annotations of DE rice genes in FOUR types of comparisons** Four groups of DE genes were derived from 4 comparisons including *SC vs RC*、*SI vs RI*、*SI vs SC* and *RI vs RC*. S represents susceptible rice plant CO39, R represent resistant rice plant C101LAC; C is control experiment and I represents infection experiment; GO ID is a list of code of GO-analysis. Genes is a list of genes involved in the GO-analysis. The orders of GO-analysis sorted from small to large display according to FDR. | | | | | | | | | |
|---|---|---|---|---|---|---|---|---|---|
| GO ID | GO Category | Term Name | GO Level | Num | List Total | Expected Num | Pop Total | p-val | FDR |
| GO:0020037 | molecular_function | heme binding | 4 | 19 | 122 | 664 | 22421 | 3.35E-09 | 4.54E-07 |
| GO:0046906 | molecular_function | tetrapyrrole binding | 3 | 19 | 122 | 681 | 22421 | 5.05E-09 | 4.54E-07 |
| GO:0005506 | molecular_function | iron ion binding | 6 | 17 | 122 | 576 | 22421 | 1.55E-08 | 9.28E-07 |
| GO:0016491 | molecular_function | oxidoreductase activity | 2 | 33 | 122 | 2139 | 22421 | 2.38E-08 | 1.07E-06 |
| GO:0055114 | biological_process | oxidation-reduction process | 3 | 33 | 99 | 2160 | 18640 | 8.94E-09 | 4.77E-06 |
| GO:0003824 | molecular_function | catalytic activity | 1 | 88 | 122 | 11089 | 22421 | 2.76E-07 | 9.94E-06 |
| GO:0016705 | molecular_function | oxidoreductase activity, acting on paired donors, with incorporation or reduction of molecular oxygen | 3 | 14 | 122 | 642 | 22421 | 1.07E-05 | 0.000322287 |
| GO:0016702 | molecular_function | oxidoreductase activity, acting on single donors with incorporation of molecular oxygen, incorporation of two atoms of oxygen | 4 | 4 | 122 | 41 | 22421 | 7.23E-05 | 0.001859462 |
| GO:0009698 | biological_process | phenylpropanoid metabolic process | 3 | 6 | 99 | 88 | 18640 | 7.34E-06 | 0.001961066 |
| GO:0016165 | molecular_function | linoleate 13S-lipoxygenase activity | 5 | 3 | 122 | 17 | 22421 | 0.000101091 | 0.002274539 |
| GO:0005618 | cellular_component | cell wall | 3 | 13 | 60 | 669 | 11387 | 3.51E-05 | 0.002387359 |
| GO:0030312 | cellular_component | external encapsulating structure | 2 | 13 | 60 | 700 | 11387 | 5.62E-05 | 0.002387359 |
| GO:0003993 | molecular_function | acid phosphatase activity | 6 | 4 | 122 | 49 | 22421 | 0.000146298 | 0.002925952 |
| GO:0046872 | molecular_function | metal ion binding | 4 | 37 | 122 | 3807 | 22421 | 0.000191186 | 0.00316661 |
| GO:0004601 | molecular_function | peroxidase activity | 2 | 7 | 122 | 221 | 22421 | 0.000211024 | 0.00316661 |
| GO:0016684 | molecular_function | oxidoreductase activity, acting on peroxide as acceptor | 3 | 7 | 122 | 222 | 22421 | 0.000216924 | 0.00316661 |
| GO:0043169 | molecular_function | cation binding | 3 | 37 | 122 | 3863 | 22421 | 0.000258034 | 0.00316661 |
| GO:0016701 | molecular_function | oxidoreductase activity, acting on single donors with incorporation of molecular oxygen | 3 | 4 | 122 | 57 | 22421 | 0.000263757 | 0.00316661 |
| GO:0009055 | molecular_function | electron carrier activity | 1 | 12 | 122 | 661 | 22421 | 0.000263884 | 0.00316661 |
| GO:0016757 | molecular_function | transferase activity, transferring glycosyl groups | 3 | 12 | 122 | 688 | 22421 | 0.00037869 | 0.00421822 |
| GO:0016209 | molecular_function | antioxidant activity | 1 | 7 | 122 | 247 | 22421 | 0.000413568 | 0.00421822 |

| | | | | | | | | | |
|---|---|---|---|---|---|---|---|---|---|
| GO:0051213 | molecular_function | dioxygenase activity | 3 | 6 | 122 | 177 | 22421 | | 0.000421822 |
| GO:0045491 | biological_process | xylan metabolic process | 7 | 5 | 99 | 70 | 18640 | | 3.51E-05 |
| GO:0006633 | biological_process | fatty acid biosynthetic process | 5 | 7 | 99 | 187 | 18640 | | 6.25E-05 |
| GO:0044710 | biological_process | single-organism metabolic process | 2 | 49 | 99 | 5713 | 18640 | | 6.53E-05 |
| GO:0010410 | biological_process | hemicellulose metabolic process | 6 | 5 | 99 | 81 | 18640 | | 7.11E-05 |
| GO:0009808 | biological_process | lignin metabolic process | 4 | 4 | 99 | 44 | 18640 | | 8.63E-05 |
| GO:0016758 | molecular_function | transferase activity, transferring hexosyl groups | 4 | 10 | 122 | 542 | 22421 | | 0.000769396 |
| GO:0046914 | molecular_function | transition metal ion binding | 5 | 26 | 122 | 2506 | 22421 | | 0.000869475 |
| GO:0004497 | molecular_function | monooxygenase activity | 3 | 8 | 122 | 375 | 22421 | | 0.001045514 |
| GO ID | GO Category | Term Name | GO Level | Num | List Total | Expected Num | Pop Total | | p-val |
| GO:0044436 | cellular_component | thylakoid part | 3 | 180 | 2618 | 344 | 11387 | | 3.16E-33 |
| GO:0034357 | cellular_component | photosynthetic membrane | 2 | 160 | 2618 | 298 | 11387 | | 2.57E-31 |
| GO:0042651 | cellular_component | thylakoid membrane | 3 | 160 | 2618 | 298 | 11387 | | 2.57E-31 |
| GO:0055035 | cellular_component | plastid thylakoid membrane | 4 | 155 | 2618 | 286 | 11387 | | 5.65E-31 |
| GO:0009535 | cellular_component | chloroplast thylakoid membrane | 5 | 154 | 2618 | 285 | 11387 | | 1.38E-30 |
| GO:0015979 | biological_process | photosynthesis | 3 | 135 | 3800 | 255 | 18640 | | 5.74E-31 |
| GO:0009579 | cellular_component | thylakoid | 3 | 207 | 2618 | 446 | 11387 | | 1.03E-28 |
| GO:0009628 | biological_process | response to abiotic stimulus | 2 | 544 | 3800 | 1779 | 18640 | | 5.31E-27 |
| GO:0042221 | biological_process | response to chemical | 2 | 583 | 3800 | 1976 | 18640 | | 1.09E-24 |
| GO:0051649 | biological_process | establishment of localization in cell | 2 | 281 | 3800 | 787 | 18640 | | 1.62E-24 |
| GO:0071840 | biological_process | cellular component organization or biogenesis | 1 | 588 | 3800 | 2015 | 18640 | | 1.25E-23 |
| GO:0044464 | cellular_component | cell part | 1 | 2260 | 2618 | 9092 | 11387 | | 1.39E-22 |
| GO:0044444 | cellular_component | cytoplasmic part | 3 | 1440 | 2618 | 5318 | 11387 | | 1.98E-22 |
| GO:0046907 | biological_process | intracellular transport | 3 | 235 | 3800 | 634 | 18640 | | 4.45E-23 |
| GO:0016043 | biological_process | cellular component organization | 2 | 540 | 3800 | 1851 | 18640 | | 1.32E-21 |
| GO:1901700 | biological_process | response to oxygen-containing compound | 3 | 372 | 3800 | 1170 | 18640 | | 1.44E-21 |
| GO:0010033 | biological_process | response to organic substance | 3 | 392 | 3800 | 1255 | 18640 | | 3.65E-21 |
| GO:0006996 | biological_process | organelle organization | 2 | 366 | 3800 | 1152 | 18640 | | 3.67E-21 |
| GO:0050896 | biological_process | response to stimulus | 1 | 1166 | 3800 | 4613 | 18640 | | 5.85E-21 |
| GO:0009416 | biological_process | response to light stimulus | 4 | 233 | 3800 | 650 | 18640 | | 1.02E-20 |
| GO:0048856 | biological_process | anatomical structure development | 2 | 505 | 3800 | 1726 | 18640 | | 1.85E-20 |

| | | | | | | | | | |
|---|---|---|---|---|---|---|---|---|---|
| GO:0032502 | biological_process | developmental process | 1 | 587 | 3800 | 2079 | 18640 | | 5.93E-20 |
| GO:0009637 | biological_process | response to blue light | 5 | 55 | 3800 | 83 | 18640 | | 1.61E-19 |
| GO:0044424 | cellular_component | intracellular part | 2 | 1968 | 2618 | 7773 | 11387 | | 9.03E-19 |
| GO:0009314 | biological_process | response to radiation | 3 | 241 | 3800 | 699 | 18640 | | 6.43E-19 |
| GO:0044435 | cellular_component | plastid part | 3 | 384 | 2618 | 1145 | 11387 | | 5.23E-18 |
| GO:0044434 | cellular_component | chloroplast part | 4 | 378 | 2618 | 1127 | 11387 | | 9.89E-18 |
| GO:0009657 | biological_process | plastid organization | 3 | 107 | 3800 | 240 | 18640 | | 1.57E-17 |
| GO:0006886 | biological_process | intracellular protein transport | 4 | 172 | 3800 | 461 | 18640 | | 1.67E-17 |
| GO:0031976 | cellular_component | plastid thylakoid | 4 | 87 | 2618 | 167 | 11387 | | 1.81E-16 |
| GO:0009534 | cellular_component | chloroplast thylakoid | 5 | 87 | 2618 | 167 | 11387 | | 1.81E-16 |
| GO:0031984 | cellular_component | organelle subcompartment | 3 | 88 | 2618 | 170 | 11387 | | 2.03E-16 |
| GO:0016482 | biological_process | cytoplasmic transport | 4 | 143 | 3800 | 363 | 18640 | | 4.45E-17 |
| GO:0044767 | biological_process | single-organism developmental process | 2 | 517 | 3800 | 1846 | 18640 | | 8.66E-17 |
| GO:0005622 | cellular_component | intracellular | 2 | 1752 | 2618 | 6862 | 11387 | | 7.59E-16 |
| GO:0009719 | biological_process | response to endogenous stimulus | 2 | 286 | 3800 | 907 | 18640 | | 3.36E-16 |
| GO:0048518 | biological_process | positive regulation of biological process | 3 | 187 | 3800 | 532 | 18640 | | 7.30E-16 |
| GO:0009725 | biological_process | response to hormone | 3 | 269 | 3800 | 847 | 18640 | | 1.02E-15 |
| GO:0009536 | cellular_component | plastid | 4 | 594 | 2618 | 1994 | 11387 | | 5.26E-15 |
| GO:0071310 | biological_process | cellular response to organic substance | 4 | 168 | 3800 | 465 | 18640 | | 1.30E-15 |
| GO:0044723 | biological_process | single-organism carbohydrate metabolic process | 3 | 274 | 3800 | 869 | 18640 | | 1.48E-15 |
| GO:0005623 | cellular_component | cell | 1 | 1967 | 2618 | 7870 | 11387 | | 7.89E-15 |
| GO:0019637 | biological_process | organophosphate metabolic process | 3 | 345 | 3800 | 1156 | 18640 | | 2.06E-15 |
| GO:0009639 | biological_process | response to red or far red light | 5 | 97 | 3800 | 223 | 18640 | | 3.54E-15 |
| GO:0043933 | biological_process | macromolecular complex subunit organization | 3 | 188 | 3800 | 545 | 18640 | | 5.01E-15 |
| GO:0005737 | cellular_component | cytoplasm | 3 | 1270 | 2618 | 4795 | 11387 | | 2.97E-14 |
| GO:0071822 | biological_process | protein complex subunit organization | 4 | 176 | 3800 | 505 | 18640 | | 1.31E-14 |
| GO:0043231 | cellular_component | intracellular membrane-bounded organelle | 3 | 1535 | 2618 | 5957 | 11387 | | 8.44E-14 |
| GO:0043227 | cellular_component | membrane-bounded organelle | 2 | 1535 | 2618 | 5958 | 11387 | | 9.12E-14 |
| | | | | | | | | | |
| GO ID | GO Category | Term Name | GO Level | Num | List Total | Expected Num | Pop Total | | p-val |
| GO:0044435 | cellular_component | plastid part | 3 | 564 | 2041 | 1145 | 11387 | | 4.28E-148 |

| | | | | | | | | | |
|---|---|---|---|---|---|---|---|---|---|
| GO:0044434 | cellular_component | chloroplast part | 4 | 553 | 2041 | 1127 | 11387 | | 7.91E-144 |
| GO:0009536 | cellular_component | plastid | 4 | 772 | 2041 | 1994 | 11387 | | 2.00E-134 |
| GO:0009507 | cellular_component | chloroplast | 5 | 635 | 2041 | 1646 | 11387 | | 7.73E-105 |
| GO:0009579 | cellular_component | thylakoid | 3 | 275 | 2041 | 446 | 11387 | | 2.33E-98 |
| GO:0015979 | biological_process | photosynthesis | 3 | 178 | 2936 | 255 | 18640 | | 1.95E-84 |
| GO:0009532 | cellular_component | plastid stroma | 4 | 284 | 2041 | 538 | 11387 | | 3.24E-79 |
| GO:0009570 | cellular_component | chloroplast stroma | 5 | 282 | 2041 | 533 | 11387 | | 6.16E-79 |
| GO:0044436 | cellular_component | thylakoid part | 3 | 213 | 2041 | 344 | 11387 | | 4.07E-76 |
| GO:0034357 | cellular_component | photosynthetic membrane | 2 | 190 | 2041 | 298 | 11387 | | 7.14E-71 |
| GO:0042651 | cellular_component | thylakoid membrane | 3 | 190 | 2041 | 298 | 11387 | | 7.14E-71 |
| GO:0009535 | cellular_component | chloroplast thylakoid membrane | 5 | 184 | 2041 | 285 | 11387 | | 6.39E-70 |
| GO:0055035 | cellular_component | plastid thylakoid membrane | 4 | 184 | 2041 | 286 | 11387 | | 1.49E-69 |
| GO:0044444 | cellular_component | cytoplasmic part | 3 | 1303 | 2041 | 5318 | 11387 | | 3.40E-66 |
| GO:0009526 | cellular_component | plastid envelope | 4 | 250 | 2041 | 523 | 11387 | | 2.76E-58 |
| GO:0044711 | biological_process | single-organism biosynthetic process | 3 | 571 | 2936 | 1934 | 18640 | | 1.84E-59 |
| GO:0009941 | cellular_component | chloroplast envelope | 5 | 247 | 2041 | 519 | 11387 | | 4.20E-57 |
| GO:0031984 | cellular_component | organelle subcompartment | 3 | 122 | 2041 | 170 | 11387 | | 3.20E-54 |
| GO:0031976 | cellular_component | plastid thylakoid | 4 | 120 | 2041 | 167 | 11387 | | 1.93E-53 |
| GO:0009534 | cellular_component | chloroplast thylakoid | 5 | 120 | 2041 | 167 | 11387 | | 1.93E-53 |
| GO:0009657 | biological_process | plastid organization | 3 | 142 | 2936 | 240 | 18640 | | 1.04E-53 |
| GO:0031975 | cellular_component | envelope | 2 | 260 | 2041 | 591 | 11387 | | 8.03E-52 |
| GO:0031967 | cellular_component | organelle envelope | 3 | 260 | 2041 | 591 | 11387 | | 8.03E-52 |
| GO:0044281 | biological_process | small molecule metabolic process | 3 | 626 | 2936 | 2316 | 18640 | | 1.60E-50 |
| GO:0009240 | biological_process | isopentenyl diphosphate biosynthetic process | 5 | 107 | 2936 | 156 | 18640 | | 8.00E-50 |
| GO:0046490 | biological_process | isopentenyl diphosphate metabolic process | 5 | 107 | 2936 | 156 | 18640 | | 8.00E-50 |
| GO:0019288 | biological_process | isopentenyl diphosphate biosynthetic process, methylerythritol 4-phosphate pathway | 5 | 105 | 2936 | 153 | 18640 | | 6.01E-49 |
| GO:0019682 | biological_process | glyceraldehyde-3-phosphate metabolic process | 4 | 105 | 2936 | 153 | 18640 | | 6.01E-49 |
| GO:0006091 | biological_process | generation of precursor metabolites and energy | 3 | 184 | 2936 | 392 | 18640 | | 7.34E-49 |
| GO:0005737 | cellular_component | cytoplasm | 3 | 1153 | 2041 | 4795 | 11387 | | 2.14E-47 |
| GO:0006090 | biological_process | pyruvate metabolic process | 7 | 107 | 2936 | 163 | 18640 | | 5.81E-47 |
| GO:0019752 | biological_process | carboxylic acid metabolic process | 5 | 408 | 2936 | 1338 | 18640 | | 3.16E-45 |
| GO:0044710 | biological_process | single-organism | 2 | 1230 | 2936 | 5713 | 18640 | | 3.20E-45 |

| | | | | | | | | | |
|---|---|---|---|---|---|---|---|---|---|
| | | metabolic process | | | | | | | |
| GO:0043436 | biological_process | oxoacid metabolic process | 4 | 409 | 2936 | 1346 | 18640 | | 6.38E-45 |
| GO:0006082 | biological_process | organic acid metabolic process | 3 | 409 | 2936 | 1348 | 18640 | | 9.61E-45 |
| GO:0006740 | biological_process | NADPH regeneration | 4 | 91 | 2936 | 131 | 18640 | | 2.91E-43 |
| GO:0006098 | biological_process | pentose-phosphate shunt | 5 | 90 | 2936 | 130 | 18640 | | 1.32E-42 |
| GO:0006081 | biological_process | cellular aldehyde metabolic process | 3 | 122 | 2936 | 225 | 18640 | | 8.36E-41 |
| GO:0006739 | biological_process | NADP metabolic process | 7 | 91 | 2936 | 137 | 18640 | | 9.50E-41 |
| GO:0051186 | biological_process | cofactor metabolic process | 3 | 176 | 2936 | 415 | 18640 | | 2.50E-39 |
| GO:0006733 | biological_process | oxidoreduction coenzyme metabolic process | 5 | 106 | 2936 | 184 | 18640 | | 7.62E-39 |
| GO:0009658 | biological_process | chloroplast organization | 4 | 106 | 2936 | 184 | 18640 | | 7.62E-39 |
| GO:0010027 | biological_process | thylakoid membrane organization | 4 | 88 | 2936 | 138 | 18640 | | 2.46E-37 |
| GO:0009668 | biological_process | plastid membrane organization | 3 | 88 | 2936 | 138 | 18640 | | 2.46E-37 |
| GO:0019362 | biological_process | pyridine nucleotide metabolic process | 5 | 93 | 2936 | 152 | 18640 | | 3.19E-37 |
| GO:0046496 | biological_process | nicotinamide nucleotide metabolic process | 6 | 92 | 2936 | 150 | 18640 | | 5.86E-37 |
| GO:0042440 | biological_process | pigment metabolic process | 3 | 108 | 2936 | 199 | 18640 | | 2.70E-36 |
| GO:0006364 | biological_process | rRNA processing | 7 | 101 | 2936 | 178 | 18640 | | 2.82E-36 |
| GO:0008610 | biological_process | lipid biosynthetic process | 4 | 230 | 2936 | 650 | 18640 | | 5.61E-36 |
| GO:0042254 | biological_process | ribosome biogenesis | 4 | 116 | 2936 | 226 | 18640 | | 8.55E-36 |
| | | | | | | | | | |
| GO ID | GO Category | Term Name | GO Level | Num | List Total | Expected Num | Pop Total | | p-val |
| GO:0044435 | cellular_component | plastid part | 3 | 680 | 3351 | 1145 | 11387 | | 2.97E-110 |
| GO:0044434 | cellular_component | chloroplast part | 4 | 669 | 3351 | 1127 | 11387 | | 3.82E-108 |
| GO:0009536 | cellular_component | plastid | 4 | 1008 | 3351 | 1994 | 11387 | | 3.60E-107 |
| GO:0009507 | cellular_component | chloroplast | 5 | 826 | 3351 | 1646 | 11387 | | 1.81E-82 |
| GO:0044444 | cellular_component | cytoplasmic part | 3 | 2018 | 3351 | 5318 | 11387 | | 4.14E-78 |
| GO:0015979 | biological_process | photosynthesis | 3 | 204 | 4827 | 255 | 18640 | | 5.48E-74 |
| GO:0009579 | cellular_component | thylakoid | 3 | 311 | 3351 | 446 | 11387 | | 4.98E-72 |
| GO:0044436 | cellular_component | thylakoid part | 3 | 244 | 3351 | 344 | 11387 | | 1.29E-58 |
| GO:0005737 | cellular_component | cytoplasm | 3 | 1794 | 3351 | 4795 | 11387 | | 5.21E-57 |
| GO:0009570 | cellular_component | chloroplast stroma | 5 | 328 | 3351 | 533 | 11387 | | 6.14E-56 |
| GO:0009532 | cellular_component | plastid stroma | 4 | 330 | 3351 | 538 | 11387 | | 8.25E-56 |
| GO:0034357 | cellular_component | photosynthetic membrane | 2 | 217 | 3351 | 298 | 11387 | | 2.42E-55 |
| GO:0042651 | cellular_component | thylakoid membrane | 3 | 217 | 3351 | 298 | 11387 | | 2.42E-55 |
| GO:0055035 | cellular_component | plastid thylakoid | 4 | 210 | 3351 | 286 | 11387 | | 1.41E-54 |

| | | | | | | | | | |
|---|---|---|---|---|---|---|---|---|---|
| | | membrane | | | | | | | |
| GO:0009535 | cellular_component | chloroplast thylakoid membrane | 5 | 209 | 3351 | 285 | 11387 | | 3.67E-54 |
| GO:0009526 | cellular_component | plastid envelope | 4 | 311 | 3351 | 523 | 11387 | | 2.20E-48 |
| GO:0009657 | biological_process | plastid organization | 3 | 171 | 4827 | 240 | 18640 | | 2.00E-49 |
| GO:0009941 | cellular_component | chloroplast envelope | 5 | 308 | 3351 | 519 | 11387 | | 1.19E-47 |
| GO:0031975 | cellular_component | envelope | 2 | 333 | 3351 | 591 | 11387 | | 1.25E-44 |
| GO:0031967 | cellular_component | organelle envelope | 3 | 333 | 3351 | 591 | 11387 | | 1.25E-44 |
| GO:0044711 | biological_process | single-organism biosynthetic process | 3 | 770 | 4827 | 1934 | 18640 | | 8.61E-46 |
| GO:0031984 | cellular_component | organelle subcompartment | 3 | 133 | 3351 | 170 | 11387 | | 5.10E-40 |
| GO:0031976 | cellular_component | plastid thylakoid | 4 | 131 | 3351 | 167 | 11387 | | 1.17E-39 |
| GO:0009534 | cellular_component | chloroplast thylakoid | 5 | 131 | 3351 | 167 | 11387 | | 1.17E-39 |
| GO:0044281 | biological_process | small molecule metabolic process | 3 | 871 | 4827 | 2316 | 18640 | | 1.74E-40 |
| GO:0006364 | biological_process | rRNA processing | 7 | 127 | 4827 | 178 | 18640 | | 4.29E-37 |
| GO:0016072 | biological_process | rRNA metabolic process | 6 | 129 | 4827 | 184 | 18640 | | 2.29E-36 |
| GO:0009240 | biological_process | isopentenyl diphosphate biosynthetic process | 5 | 115 | 4827 | 156 | 18640 | | 6.11E-36 |
| GO:0046490 | biological_process | isopentenyl diphosphate metabolic process | 5 | 115 | 4827 | 156 | 18640 | | 6.11E-36 |
| GO:0009658 | biological_process | chloroplast organization | 4 | 128 | 4827 | 184 | 18640 | | 1.55E-35 |
| GO:0019288 | biological_process | isopentenyl diphosphate biosynthetic process, methylerythritol 4-phosphate pathway | 5 | 113 | 4827 | 153 | 18640 | | 1.82E-35 |
| GO:0019682 | biological_process | glyceraldehyde-3-phospha te metabolic process | 4 | 113 | 4827 | 153 | 18640 | | 1.82E-35 |
| GO:0042254 | biological_process | ribosome biogenesis | 4 | 147 | 4827 | 226 | 18640 | | 2.17E-35 |
| GO:0022613 | biological_process | ribonucleoprotein complex biogenesis | 3 | 147 | 4827 | 226 | 18640 | | 2.17E-35 |
| GO:0044446 | cellular_component | intracellular organelle part | 2 | 1158 | 3351 | 3042 | 11387 | | 1.51E-33 |
| GO:0043231 | cellular_component | intracellular membrane-bounded organelle | 3 | 2044 | 3351 | 5957 | 11387 | | 1.77E-33 |
| GO:0043227 | cellular_component | membrane-bounded organelle | 2 | 2044 | 3351 | 5958 | 11387 | | 2.05E-33 |
| GO:0034660 | biological_process | ncRNA metabolic process | 5 | 214 | 4827 | 391 | 18640 | | 1.99E-34 |
| GO:0044422 | cellular_component | organelle part | 1 | 1160 | 3351 | 3057 | 11387 | | 6.89E-33 |
| GO:0006090 | biological_process | pyruvate metabolic process | 7 | 116 | 4827 | 163 | 18640 | | 8.12E-34 |
| GO:0010207 | biological_process | photosystem II assembly | 7 | 84 | 4827 | 101 | 18640 | | 1.70E-33 |
| GO:0044710 | biological_process | single-organism metabolic process | 2 | 1813 | 4827 | 5713 | 18640 | | 3.59E-33 |
| GO:0006091 | biological_process | generation of precursor metabolites and energy | 3 | 212 | 4827 | 392 | 18640 | | 4.09E-33 |

| | | | | | | | | | |
|---|---|---|---|---|---|---|---|---|---|
| GO:0010027 | biological_process | thylakoid membrane organization | 4 | 101 | 4827 | 138 | 18640 | | 2.80E-31 |
| GO:0009668 | biological_process | plastid membrane organization | 3 | 101 | 4827 | 138 | 18640 | | 2.80E-31 |
| GO:0043436 | biological_process | oxoacid metabolic process | 4 | 534 | 4827 | 1346 | 18640 | | 1.01E-30 |
| GO:0019752 | biological_process | carboxylic acid metabolic process | 5 | 531 | 4827 | 1338 | 18640 | | 1.38E-30 |
| GO:0006082 | biological_process | organic acid metabolic process | 3 | 534 | 4827 | 1348 | 18640 | | 1.57E-30 |
| GO:0044802 | biological_process | single-organism membrane organization | 2 | 106 | 4827 | 152 | 18640 | | 8.57E-30 |
| GO:0044085 | biological_process | cellular component biogenesis | 2 | 178 | 4827 | 323 | 18640 | | 2.66E-29 |

**Table S4. Candidate disease resistant R genes**

| Gene annotations | | | | | | Normalized expression levels at different time points | | | | | | | | | |
|---|---|---|---|---|---|---|---|---|---|---|---|---|---|---|---|
| Gene stable ID | Chromosome/scaffold name | Gene start (bp) | Gene end (bp) | Gene name | Gene description | SC00 | RC00 | SI08 | RI08 | SC08 | RC08 | SI24 | RI24 | SI48 | RI48 |
| BGIOSGA001054 | 1 | 30650516 | 30651259 | | Putative uncharacterized protein [Source:UniProtKB/TrEMBL;Acc:A2WTL3] | 96.91207 | 65.45105 | 277.2364 | 59.14279 | 91.7374 | 3.732427 | 251.5092 | 5.12183 | 214.9724 | 35.18242 |
| BGIOSGA001872 | 1 | 11201847 | 11203348 | | Putative uncharacterized protein [Source:UniProtKB/TrEMBL;Acc:A2WNL4] | 8.810189 | 7.700124 | 21.015 | 12.56784 | 285.9562 | 39.56373 | 11.91987 | 0.640229 | 271.3159 | 68.80118 |
| BGIOSGA001992 | 1 | 8959019 | 8961448 | | Putative uncharacterized protein [Source:UniProtKB/TrEMBL;Acc:A2WMV6] | 162.9885 | 53.90086 | 18.59019 | 13.30713 | 1004.153 | 77.63449 | 33.37563 | 4.481601 | 523.5617 | 141.5115 |
| BGIOSGA002993 | 1 | 6714072 | 6717048 | | Putative uncharacterized protein [Source:UniProtKB/TrEMBL;Acc:B8AAJ1] | 57.26623 | 40.42565 | 7.274423 | 5.914279 | 352.8997 | 12.69025 | 15.49583 | 1.280458 | 109.2198 | 39.09158 |
| BGIOSGA003051 | 1 | 7532808 | 7534522 | | Putative uncharacterized protein [Source:UniProtKB/TrEMBL;Acc:A2WMB5] | 8.810189 | 737.2868 | 4.041346 | 107.1963 | 6.611704 | 32.09887 | 0 | 31.37121 | 6.067769 | 14.8548 |
| BGIOSGA003503 | 1 | 16072868 | 16077053 | | Putative uncharacterized protein [Source:UniProtKB/TrEMBL;Acc:B8A7X1] | 0 | 159.7776 | 0 | 2.217855 | 0 | 5.225398 | 0 | 42.2551 | 0 | 25.80044 |
| BGIOSGA005655 | 2 | 32902134 | 32903237 | | Putative uncharacterized protein [Source:UniProtKB/TrEMBL;Acc:A2X9F4] | 2698.12 | 1128.068 | 34.75558 | 31.04996 | 391.7435 | 54.49344 | 446.9951 | 50.57807 | 535.6973 | 222.822 |
| BGIOSGA008255 | 2 | 18910371 | 18913246 | | Putative uncharacterized protein [Source:UniProtKB/TrEMBL;Acc:A2X540] | 63.87387 | 34.65056 | 11.31577 | 5.174994 | 28.92621 | 0 | 11.91987 | 0.640229 | 25.1379 | 3.909158 |
| BGIOSGA009561 | 3 | 38661094 | 38664277 | | Putative uncharacterized protein [Source:UniProtKB/TrEMBL;Acc:A2XND8] | 594.6877 | 132.8271 | 42.03 | 27.35354 | 348.7674 | 35.8313 | 175.2221 | 3.201144 | 217.5728 | 59.4192 |
| BGIOSGA010560 | 3 | 18291941 | 18292315 | | Putative uncharacterized protein [Source:UniProtKB/TrEMBL;Acc:A2XHS6] | 0 | 956.7403 | 0 | 53.22851 | 0 | 54.49344 | 0 | 33.93213 | 0 | 68.80118 |
| BGIOSGA012763 | 3 | 16929251 | 16930654 | | Putative uncharacterized protein [Source:UniProtKB/TrEMBL;Acc:A2XHE8] | 118.9375 | 105.8767 | 27.48115 | 24.3964 | 66.11704 | 4.478913 | 71.51921 | 8.963203 | 78.01417 | 23.45495 |
| BGIOSGA012819 | 3 | 17987574 | 17988149 | | Putative uncharacterized protein [Source:UniProtKB/TrEMBL;Acc:A2XHP8] | 1277.477 | 346.5056 | 3.233077 | 1.47857 | 566.9537 | 76.888 | 110.8548 | 2.560915 | 199.3695 | 57.85553 |
| BGIOSGA013779 | 3 | 38078390 | 38078754 | | Putative uncharacterized protein [Source:UniProtKB/TrEMBL;Acc:A2XN54] | 2422.802 | 702.6363 | 130.9396 | 135.2891 | 986.7969 | 104.508 | 348.0602 | 25.60915 | 461.1504 | 100.8563 |
| BGIOSGA013987 | 3 | 40603153 | 40603374 | | Putative uncharacterized protein [Source:UniProtKB/TrEMBL;Acc:B8ANS1] | 0 | 103.9517 | 0 | 24.3964 | 0 | 12.69025 | 0 | 7.042517 | 3.467296 | 3.127326 |
| BGIOSGA014724 | 4 | 24345073 | 24360237 | | Putative uncharacterized protein [Source:UniProtKB/TrEMBL;Acc:B8ARR7] | 6.607641 | 442.7571 | 7.274423 | 363.7282 | 0 | 9.704311 | 42.91153 | 763.7929 | 110.0867 | 801.3773 |
| BGIOSGA015129 | 4 | 18330993 | 18331901 | | Putative uncharacterized protein [Source:UniProtKB/TrEMBL;Acc:A2XT94] | 533.0164 | 167.4777 | 16.16539 | 21.43926 | 23.14097 | 0 | 138.2705 | 5.12183 | 60.67769 | 10.16381 |
| BGIOSGA015249 | 4 | 15486569 | 15493506 | | Putative uncharacterized protein [Source:UniProtKB/TrEMBL;Acc:B | 0 | 13.47522 | 1.616539 | 177.4284 | 0.826463 | 50.76101 | 0 | 360.4488 | 40.74073 | 590.2828 |

| | | | | | | | | | | | | | | | |
|---|---|---|---|---|---|---|---|---|---|---|---|---|---|---|---|
| | | | | | 8AT19] | | | | | | | | | | |
| BGIOSGA015397 | 4 | 11029803 | 11030328 | | Putative uncharacterized protein [Source:UniProtKB/TrEMBL;Acc:A2XRL4] | 0 | 136.6772 | 0 | 34.74639 | 0 | 300.0871 | 0 | 35.21258 | 0.866824 | 86.00147 |
| BGIOSGA015743 | 4 | 89164 | 90761 | | Putative uncharacterized protein [Source:UniProtKB/TrEMBL;Acc:A2XPI4] | 387.6483 | 80.8513 | 16.97365 | 21.43926 | 371.9084 | 35.8313 | 176.4141 | 7.682745 | 285.1851 | 123.5294 |
| BGIOSGA015897 | 4 | 4119632 | 4121925 | | Putative uncharacterized protein [Source:UniProtKB/TrEMBL;Acc:B8AVD7] | 0 | 19.25031 | 0 | 215.1319 | 0.826463 | 38.07076 | 0 | 139.5699 | 1.733648 | 147.7662 |
| BGIOSGA016141 | 4 | 13184835 | 13189761 | | Probable aldo-keto reductase 1 [Source:UniProtKB/Swiss-Prot;Acc:B8ASB2] | 77.08915 | 48.12577 | 20.20673 | 25.87497 | 383.4789 | 50.76101 | 45.2955 | 7.682745 | 86.68241 | 30.49143 |
| BGIOSGA016729 | 4 | 23875756 | 23877120 | | Putative uncharacterized protein [Source:UniProtKB/TrEMBL;Acc:A2XVB4] | 68.27896 | 19.25031 | 46.87962 | 14.7857 | 277.6916 | 13.43674 | 29.79967 | 1.280458 | 182.0331 | 22.67311 |
| BGIOSGA016974 | 4 | 27946878 | 27947379 | | Putative uncharacterized protein [Source:UniProtKB/TrEMBL;Acc:A2XWP7] | 68.27896 | 25.0254 | 0.808269 | 0 | 188.4336 | 16.42268 | 1.191987 | 0 | 130.8904 | 10.94564 |
| BGIOSGA017694 | 5 | 27624827 | 27626266 | | Putative uncharacterized protein [Source:UniProtKB/TrEMBL;Acc:A2Y6R1] | 154.1783 | 28.87546 | 6.466154 | 2.957139 | 90.91094 | 8.957825 | 46.48749 | 0.640229 | 72.81322 | 16.41846 |
| BGIOSGA017696 | 5 | 27610938 | 27612395 | | Putative uncharacterized protein [Source:UniProtKB/TrEMBL;Acc:A2Y6Q8] | 79.2917 | 46.20074 | 7.274423 | 8.132133 | 57.02595 | 2.985942 | 38.14358 | 3.201144 | 61.54451 | 42.2189 |
| BGIOSGA017730 | 5 | 26952902 | 26954532 | | Putative uncharacterized protein [Source:UniProtKB/TrEMBL;Acc:B8AZZ0] | 68.27896 | 25.0254 | 12.93231 | 5.914279 | 165.2926 | 11.94377 | 56.02338 | 1.920686 | 139.5587 | 35.18242 |
| BGIOSGA017928 | 5 | 23777262 | 23780195 | | Putative uncharacterized protein [Source:UniProtKB/TrEMBL;Acc:B8AYU7] | 165.191 | 26.95043 | 44.45481 | 28.09282 | 431.4137 | 9.704311 | 72.7112 | 0 | 185.5004 | 19.54579 |
| BGIOSGA018662 | 5 | 5875303 | 5876502 | | Putative uncharacterized protein [Source:UniProtKB/TrEMBL;Acc:A2Y180] | 2779.614 | 635.2602 | 505.9766 | 207.739 | 1934.75 | 91.81771 | 1220.595 | 16.64595 | 2062.174 | 500.3722 |
| BGIOSGA019138 | 5 | 2277186 | 2278863 | | Putative uncharacterized protein [Source:UniProtKB/TrEMBL;Acc:A2Y052] | 614.5107 | 90.47645 | 30.71423 | 12.56784 | 1638.05 | 115.7052 | 38.14358 | 0.640229 | 449.0149 | 57.85553 |
| BGIOSGA019220 | 5 | 3598394 | 3601585 | | Putative uncharacterized protein [Source:UniProtKB/TrEMBL;Acc:B8AYD4] | 24.22802 | 446.6072 | 14.54885 | 261.7068 | 24.79389 | 89.57825 | 30.99166 | 198.4709 | 35.53979 | 38.30975 |
| BGIOSGA020119 | 5 | 26051905 | 26053238 | | Putative uncharacterized protein [Source:UniProtKB/TrEMBL;Acc:A2Y668] | 174.0012 | 80.8513 | 5.657885 | 2.217855 | 76.0346 | 7.464854 | 146.6144 | 13.4448 | 106.6194 | 41.43707 |
| BGIOSGA020335 | 5 | 28939234 | 28941927 | | Putative uncharacterized protein [Source:UniProtKB/TrEMBL;Acc:A2Y7B0] | 145.3681 | 38.50062 | 13.74058 | 3.696424 | 200.0041 | 11.19728 | 63.1753 | 0 | 197.6359 | 56.29187 |
| BGIOSGA020564 | 6 | 31728746 | 31730240 | | Putative uncharacterized protein [Source:UniProtKB/TrEMBL;Acc:B8B278] | 330.3821 | 257.9541 | 44.45481 | 19.96069 | 277.6916 | 21.64808 | 197.8698 | 24.32869 | 405.6737 | 120.4021 |
| BGIOSGA021894 | 6 | 3262689 | 3262886 | | Putative uncharacterized protein [Source:UniProtKB/TrEMBL;Acc:B8B2Y9] | 121.1401 | 48.12577 | 28.28942 | 12.56784 | 260.3359 | 17.91565 | 133.5025 | 0.640229 | 220.1733 | 43.78257 |
| BGIOSGA021931 | 6 | 2690869 | 2691351 | | Putative uncharacterized protein [Source:UniProtKB/TrEMBL;Acc:A2Y992] | 1317.123 | 359.9808 | 149.5298 | 147.1177 | 2900.885 | 88.08528 | 755.7197 | 6.402288 | 387.4704 | 63.32835 |

| | | | | | | | | | | | | | | | |
|---|---|---|---|---|---|---|---|---|---|---|---|---|---|---|---|
| BGIOSGA021934 | 6 | 2661792 | 2664013 | | Putative uncharacterized protein [Source:UniProtKB/TrEMBL;Acc:A2Y986] | 180.6089 | 32.72552 | 39.60519 | 36.96424 | 1588.462 | 16.42268 | 72.7112 | 0 | 170.7643 | 16.41846 |
| BGIOSGA022217 | 6 | 1577063 | 1578370 | | Putative uncharacterized protein [Source:UniProtKB/TrEMBL;Acc:B8B1X8] | 127.7477 | 107.8017 | 15.35712 | 14.7857 | 337.1969 | 35.8313 | 146.6144 | 4.481601 | 350.1969 | 71.9285 |
| BGIOSGA022314 | 6 | 3010783 | 3012448 | | Putative uncharacterized protein [Source:UniProtKB/TrEMBL;Acc:A2Y9D2] | 107.9248 | 94.32651 | 359.6798 | 28.83211 | 109.0931 | 2.239456 | 46.48749 | 2.560915 | 81.48146 | 33.61876 |
| BGIOSGA022371 | 6 | 3805405 | 3808042 | | Putative uncharacterized protein [Source:UniProtKB/TrEMBL;Acc:B8B378] | 629.9285 | 361.9058 | 81.63519 | 170.7748 | 301.659 | 38.07076 | 851.0786 | 21.76778 | 558.2347 | 139.9478 |
| BGIOSGA022555 | 6 | 7031597 | 7032577 | | Putative uncharacterized protein [Source:UniProtKB/TrEMBL;Acc:A2YAR2] | 240.0776 | 30.80049 | 51.72923 | 11.08927 | 515.7129 | 40.31021 | 178.798 | 1.920686 | 613.7114 | 143.857 |
| BGIOSGA022782 | 6 | 12830188 | 12831708 | | Putative uncharacterized protein [Source:UniProtKB/TrEMBL;Acc:B8B134] | 0 | 148.2274 | 0 | 132.332 | 0 | 13.43674 | 1.191987 | 35.85281 | 1.733648 | 7.818315 |
| BGIOSGA023145 | 6 | 23342915 | 23343911 | | Putative uncharacterized protein [Source:UniProtKB/TrEMBL;Acc:B8B429] | 1332.541 | 0 | 360.4881 | 14.7857 | 376.0407 | 0 | 2330.334 | 21.12755 | 341.5287 | 0 |
| BGIOSGA023146 | 6 | 23350874 | 23352881 | | Putative uncharacterized protein [Source:UniProtKB/TrEMBL;Acc:A2YED6] | 3464.607 | 0 | 1285.956 | 50.27137 | 1650.447 | 1.492971 | 6530.896 | 104.3573 | 1606.225 | 0.781832 |
| BGIOSGA023820 | 7 | 24928457 | 24930290 | | Putative uncharacterized protein [Source:UniProtKB/TrEMBL;Acc:A2YP90] | 158.5834 | 71.22614 | 33.13904 | 3.696424 | 375.2142 | 41.80318 | 33.37563 | 4.481601 | 334.5941 | 85.21964 |
| BGIOSGA023955 | 7 | 22590584 | 22591375 | | Putative uncharacterized protein [Source:UniProtKB/TrEMBL;Acc:A2YNE1] | 222.4573 | 51.97583 | 58.19539 | 24.3964 | 245.4595 | 32.84536 | 26.22371 | 3.201144 | 60.67769 | 12.5093 |
| BGIOSGA024098 | 7 | 20357418 | 20361571 | | Putative uncharacterized protein [Source:UniProtKB/TrEMBL;Acc:B8B7F6] | 19.82292 | 184.803 | 23.43981 | 38.44281 | 10.74402 | 181.396 | 20.26378 | 51.85853 | 22.53743 | 161.8391 |
| BGIOSGA024239 | 7 | 17619997 | 17621445 | | Putative uncharacterized protein [Source:UniProtKB/TrEMBL;Acc:A2YLQ6] | 343.5974 | 111.6518 | 11.31577 | 14.7857 | 61.98473 | 5.971884 | 188.3339 | 1.280458 | 76.28052 | 13.29114 |
| BGIOSGA025191 | 7 | 3149741 | 3153534 | | Putative uncharacterized protein [Source:UniProtKB/TrEMBL;Acc:B8B7F3] | 59.46877 | 19.25031 | 26.67289 | 25.87497 | 185.1277 | 24.63402 | 39.33557 | 0 | 97.0843 | 46.12806 |
| BGIOSGA025363 | 7 | 5679507 | 5681015 | | Putative uncharacterized protein [Source:UniProtKB/TrEMBL;Acc:A2YJA1] | 50.65858 | 15.40025 | 12.12404 | 17.00355 | 27.27328 | 0.746485 | 22.64775 | 0 | 8.668241 | 6.254652 |
| BGIOSGA026540 | 8 | 29159490 | 29160484 | | Putative uncharacterized protein [Source:UniProtKB/TrEMBL;Acc:A2YXR5] | 70.48151 | 34.65056 | 21.82327 | 9.610703 | 295.0473 | 35.08482 | 40.52755 | 1.920686 | 138.6919 | 34.40059 |
| BGIOSGA028112 | 8 | 5529308 | 5530162 | | Putative uncharacterized protein [Source:UniProtKB/TrEMBL;Acc:A2YRZ0] | 22.02547 | 0 | 98.60885 | 107.1963 | 38.84376 | 1.492971 | 38.14358 | 56.34013 | 24.27107 | 261.1317 |
| BGIOSGA028887 | 8 | 25834855 | 25837012 | | Putative uncharacterized protein [Source:UniProtKB/TrEMBL;Acc:B8BC10] | 162.9885 | 51.97583 | 52.5375 | 55.44636 | 337.1969 | 26.87348 | 61.98332 | 1.920686 | 221.0401 | 68.01934 |
| BGIOSGA029048 | 8 | 28215916 | 28221387 | | Putative uncharacterized protein [Source:UniProtKB/TrEMBL;Acc:B8B930] | 328.1795 | 57.75093 | 16.16539 | 10.34999 | 513.2336 | 70.91612 | 90.591 | 3.841373 | 546.0992 | 17.20029 |
| BGIOSGA030354 | 9 | 3288251 | 3291536 | | Putative uncharacterized protein [Source:UniProtKB/TrEMBL;Acc:A2YYW2] | 140.963 | 61.60099 | 18.59019 | 19.96069 | 420.6697 | 52.25398 | 57.21537 | 2.560915 | 240.9771 | 72.71033 |

**Table S5. #. reads (normalized) mapped to miRNA hairpins under each condition.**
(The first row is the name of 10 small RNA-Seq libraries; the second row is total reads mapped to rice genome. And the first column is a list of miRNAs expressed in our samples. Other columns are the amount of miRNAs detected in each library).

| **library name** | **SC00** | **RC00** | **SI08** | **RI08** | **SC08** | **RC08** | **SI24** | **RI24** | **SI48** | **RI48** |
|---|---|---|---|---|---|---|---|---|---|---|
| **# reads to rice genome** | **17639775** | **16364984** | **13692898** | **15015109** | **17725524** | **16823420** | **12598797** | **13526766** | **11429751** | **8487961** |
| osa-MIR156a | 515778.60 | 713423.09 | 608518.55 | 744475.21 | 694034.83 | 841766.93 | 1070153.67 | 1299680.15 | 714648.57 | 974132.27 |
| osa-MIR156b | 515770.48 | 713508.03 | 608567.73 | 744573.51 | 694097.08 | 841858.08 | 1070199.17 | 1299794.57 | 714736.33 | 974272.40 |
| osa-MIR156c | 515894.78 | 713533.43 | 608605.41 | 744602.14 | 694184.40 | 841927.92 | 1070335.67 | 1299907.93 | 714768.93 | 974306.17 |
| osa-MIR156d | 525521.68 | 723782.39 | 619032.33 | 758118.43 | 705103.55 | 855862.82 | 1085096.35 | 1315317.16 | 723888.99 | 986438.52 |
| osa-MIR156e | 515742.05 | 713408.20 | 608495.52 | 744466.62 | 694004.11 | 841750.75 | 1070061.54 | 1299597.52 | 714647.31 | 974088.37 |
| osa-MIR156f | 525243.84 | 723679.94 | 618920.35 | 757990.54 | 705007.34 | 855748.68 | 1084858.62 | 1315246.18 | 723746.06 | 986323.71 |
| osa-MIR156g | 515898.03 | 713540.43 | 608608.55 | 744605.96 | 694195.72 | 841940.70 | 1070345.90 | 1299915.34 | 714776.45 | 974316.30 |
| osa-MIR156h | 525485.94 | 723853.32 | 619071.05 | 758209.10 | 705211.89 | 855935.23 | 1085106.59 | 1315518.45 | 723961.71 | 986556.70 |
| osa-MIR156i | 515738.80 | 713405.58 | 608494.47 | 744461.85 | 693996.83 | 841743.93 | 1070060.40 | 1299593.28 | 714641.04 | 974083.31 |
| osa-MIR156j | 525485.94 | 723855.95 | 619073.15 | 758210.05 | 705212.70 | 855936.08 | 1085106.59 | 1315519.51 | 723961.71 | 986556.70 |
| osa-MIR156k | 111135.90 | 247756.30 | 208001.52 | 252775.18 | 228246.36 | 310769.75 | 373787.60 | 542291.67 | 263293.23 | 388704.74 |
| osa-MIR156l | 1004.93 | 1574.47 | 1335.42 | 1741.79 | 1739.82 | 2259.87 | 2530.83 | 3900.78 | 1875.67 | 2814.45 |
| osa-MIR159a | 60814.41 | 40881.15 | 21136.41 | 18561.27 | 86716.15 | 47850.05 | 38272.91 | 60933.48 | 142579.66 | 72606.73 |
| osa-MIR159b | 60560.13 | 40749.80 | 21049.55 | 18499.24 | 86358.00 | 47659.24 | 38049.97 | 60859.32 | 142264.95 | 72365.30 |
| osa-MIR159c | 57808.54 | 39149.05 | 19972.63 | 17137.30 | 84132.29 | 45791.20 | 35909.29 | 58661.03 | 140288.98 | 70558.78 |
| osa-MIR159d | 57819.91 | 39155.18 | 19974.73 | 17142.07 | 84141.19 | 45800.57 | 35922.94 | 58665.27 | 140291.49 | 70558.78 |
| osa-MIR159e | 57807.73 | 39149.05 | 19972.63 | 17135.39 | 84131.49 | 45791.20 | 35909.29 | 58659.97 | 140287.73 | 70555.41 |
| osa-MIR159f | 58123.75 | 39314.55 | 20058.45 | 17232.74 | 84406.36 | 46005.01 | 36091.28 | 58891.98 | 140619.98 | 70785.02 |
| osa-MIR160a | 945.63 | 415.07 | 375.72 | 474.34 | 793.91 | 607.35 | 960.01 | 206.59 | 935.33 | 378.19 |
| osa-MIR160b | 944.01 | 415.07 | 374.67 | 473.38 | 795.53 | 605.64 | 956.60 | 206.59 | 936.58 | 378.19 |
| osa-MIR160c | 938.32 | 387.05 | 345.37 | 429.48 | 777.75 | 584.35 | 934.98 | 152.56 | 922.79 | 349.48 |
| osa-MIR160d | 952.94 | 414.20 | 379.90 | 475.29 | 797.15 | 611.61 | 963.42 | 206.59 | 936.58 | 384.94 |
| osa-MIR160e | 745.78 | 312.62 | 287.81 | 331.18 | 602.31 | 427.61 | 693.84 | 112.30 | 772.33 | 285.33 |
| osa-MIR160f | 775.84 | 338.89 | 284.67 | 380.81 | 663.75 | 511.94 | 799.63 | 164.21 | 801.17 | 352.86 |
| osa-MIR162a | 1439.57 | 861.67 | 636.31 | 858.01 | 751.07 | 1119.29 | 1622.00 | 1394.19 | 535.37 | 1033.26 |
| osa-MIR162b | 1380.26 | 832.77 | 624.80 | 832.24 | 734.09 | 1090.33 | 1591.29 | 1361.35 | 526.59 | 1007.93 |
| osa-MIR164a | 5881.75 | 5376.68 | 5317.59 | 4890.37 | 6189.62 | 5358.79 | 7449.16 | 7812.15 | 4942.44 | 5731.89 |
| osa-MIR164b | 5937.00 | 5423.97 | 5356.32 | 4935.23 | 6242.98 | 5409.05 | 7500.34 | 7853.47 | 4986.32 | 5782.54 |
| osa-MIR164c | 5313.08 | 4894.18 | 4817.34 | 4459.94 | 5629.35 | 4804.26 | 6891.81 | 7174.38 | 4522.42 | 5059.93 |
| osa-MIR164d | 5949.18 | 5469.50 | 5303.99 | 4936.18 | 6231.66 | 5527.45 | 7636.84 | 8122.56 | 4968.77 | 5836.56 |
| osa-MIR164e | 40.62 | 45.54 | 58.61 | 45.81 | 66.29 | 46.00 | 63.70 | 65.68 | 57.67 | 75.97 |
| osa-MIR164f | 5923.19 | 5434.47 | 5368.87 | 4920.91 | 6249.45 | 5409.90 | 7492.38 | 7870.42 | 4991.33 | 5782.54 |
| osa-MIR166a | 246309.80 | 113867.37 | 85326.39 | 110429.46 | 117959.36 | 146964.23 | 227959.72 | 136412.77 | 65170.71 | 111575.13 |

| | | | | | | | | | | |
|---|---|---|---|---|---|---|---|---|---|---|
| osa-MIR166b | 246096.14 | 113836.72 | 85259.41 | 110384.60 | 117952.08 | 146905.45 | 227831.19 | 136361.92 | 65179.49 | 111576.82 |
| osa-MIR166c | 244286.94 | 112830.57 | 84391.81 | 109478.87 | 116786.27 | 145587.69 | 225361.78 | 135023.88 | 64532.53 | 110411.87 |
| osa-MIR166d | 246550.27 | 114046.01 | 85445.70 | 110598.39 | 118212.41 | 147204.44 | 228190.62 | 136569.57 | 65326.18 | 111814.87 |
| osa-MIR166e | 166378.88 | 74104.47 | 57337.07 | 74921.76 | 77813.32 | 94603.80 | 152688.98 | 86955.96 | 44767.80 | 74281.56 |
| osa-MIR166f | 246241.56 | 113821.84 | 85271.97 | 110381.74 | 117911.66 | 146906.30 | 227871.00 | 136362.98 | 65156.92 | 111544.74 |
| osa-MIR166g | 213346.80 | 95082.27 | 72562.49 | 96293.76 | 101564.46 | 122333.90 | 201433.25 | 114265.64 | 57735.74 | 96928.84 |
| osa-MIR166h | 190862.90 | 82595.94 | 63907.40 | 85105.27 | 89508.60 | 106881.06 | 177405.76 | 98903.02 | 52496.15 | 85071.69 |
| osa-MIR166i | 65059.18 | 31232.90 | 22091.93 | 30338.63 | 33604.73 | 40243.31 | 67805.65 | 39896.62 | 17521.70 | 32648.97 |
| osa-MIR166j | 65059.18 | 31232.90 | 22090.88 | 30338.63 | 33604.73 | 40243.31 | 67805.65 | 39896.62 | 17521.70 | 32648.97 |
| osa-MIR166k | 23596.88 | 12470.57 | 9802.12 | 12202.07 | 14558.87 | 14126.56 | 21223.67 | 13629.41 | 7806.09 | 10575.71 |
| osa-MIR166l | 43615.15 | 23521.66 | 17319.59 | 22033.40 | 25264.59 | 27744.58 | 42366.59 | 27087.20 | 12285.88 | 21132.86 |
| osa-MIR166m | 211379.17 | 100877.53 | 75840.33 | 97379.87 | 103030.21 | 127079.38 | 191007.39 | 117995.85 | 57059.95 | 95657.53 |
| osa-MIR166n | 224566.00 | 101988.76 | 76950.73 | 99757.30 | 106314.20 | 132237.99 | 204357.63 | 121665.67 | 60119.19 | 99972.91 |
| osa-MIR167a | 71600.60 | 73836.51 | 53130.93 | 55832.71 | 62567.25 | 71377.27 | 96198.68 | 96252.36 | 33212.88 | 46841.09 |
| osa-MIR167b | 191234.17 | 208531.94 | 148921.92 | 166607.66 | 189367.19 | 223649.18 | 283806.22 | 331306.47 | 91445.12 | 139334.69 |
| osa-MIR167c | 70857.26 | 73318.98 | 52741.61 | 55472.89 | 62152.51 | 70923.25 | 95774.41 | 95943.01 | 32845.52 | 46446.02 |
| osa-MIR167d | 194681.98 | 212143.25 | 151434.72 | 170051.15 | 193318.98 | 228670.65 | 289039.63 | 337951.14 | 93167.82 | 142061.34 |
| osa-MIR167e | 192896.33 | 210075.77 | 149911.97 | 168035.45 | 191287.30 | 226013.82 | 286487.19 | 334699.79 | 92469.46 | 140904.84 |
| osa-MIR167f | 194609.68 | 212085.46 | 151394.95 | 170002.48 | 193251.06 | 228619.54 | 288981.62 | 337867.45 | 93144.00 | 142037.71 |
| osa-MIR167g | 194691.73 | 212160.76 | 151476.58 | 170054.01 | 193302.81 | 228742.20 | 289160.20 | 338049.67 | 93221.74 | 142142.38 |
| osa-MIR167h | 194652.73 | 212095.09 | 151393.90 | 169985.30 | 193306.85 | 228651.91 | 288966.83 | 337855.80 | 93159.05 | 142046.15 |
| osa-MIR167i | 192947.51 | 210158.96 | 149965.34 | 168110.85 | 191355.21 | 226050.45 | 286575.91 | 334812.09 | 92494.54 | 140936.91 |
| osa-MIR167j | 194660.86 | 212141.50 | 151430.53 | 170034.93 | 193302.00 | 228673.20 | 289033.94 | 337945.85 | 93154.03 | 142049.52 |
| osa-MIR168a | 612626.06 | 649275.11 | 524772.47 | 624198.20 | 551873.21 | 662535.82 | 1080350.91 | 1285640.74 | 573915.74 | 891219.96 |
| osa-MIR168b | 0.81 | 0.88 | 1.05 | 1.91 | 2.43 | 0.00 | 5.69 | 2.12 | 2.51 | 1.69 |
| osa-MIR169a | 3555.05 | 3775.06 | 2453.15 | 2899.48 | 2719.68 | 3321.24 | 3601.17 | 4922.06 | 2469.96 | 4298.49 |
| osa-MIR169b | 3218.72 | 3541.25 | 2050.22 | 2420.37 | 2594.37 | 2976.25 | 2820.88 | 3988.71 | 1739.01 | 3246.66 |
| osa-MIR169c | 3447.81 | 3846.86 | 2231.28 | 2616.02 | 2751.21 | 3242.02 | 2964.19 | 4286.40 | 1864.38 | 3518.48 |
| osa-MIR169d | 184.41 | 189.15 | 106.75 | 108.80 | 139.86 | 165.25 | 150.14 | 261.68 | 100.30 | 199.22 |
| osa-MIR169e | 3608.67 | 4180.50 | 2321.28 | 2679.02 | 2924.22 | 3585.30 | 2827.70 | 4772.68 | 1760.32 | 3557.32 |
| osa-MIR169f | 1003.31 | 1263.61 | 711.66 | 784.52 | 761.58 | 1232.58 | 745.03 | 1737.45 | 633.16 | 1115.99 |
| osa-MIR169g | 2241.40 | 3250.53 | 1506.01 | 1706.48 | 1437.45 | 2475.38 | 2196.42 | 3746.10 | 867.62 | 1928.08 |
| osa-MIR169h | 2344.58 | 3280.30 | 1516.47 | 1779.97 | 1455.24 | 2462.61 | 2223.71 | 3821.32 | 896.46 | 1973.66 |
| osa-MIR169i | 2651.66 | 3533.37 | 1639.97 | 1922.17 | 1615.32 | 2702.82 | 2418.22 | 4192.12 | 954.13 | 2167.82 |
| osa-MIR169j | 2341.33 | 3270.67 | 1501.82 | 1774.24 | 1439.07 | 2443.87 | 2229.40 | 3816.02 | 893.95 | 1963.53 |
| osa-MIR169k | 2351.89 | 3301.32 | 1510.19 | 1787.60 | 1448.77 | 2466.01 | 2255.56 | 3872.17 | 903.98 | 1978.73 |
| osa-MIR169l | 2342.95 | 3282.93 | 1517.52 | 1779.97 | 1455.24 | 2461.76 | 2222.58 | 3823.44 | 895.21 | 1973.66 |

| | | | | | | | | | | |
|---|---|---|---|---|---|---|---|---|---|---|
| osa-MIR169m | 2351.89 | 3285.55 | 1510.19 | 1786.65 | 1442.30 | 2454.94 | 2231.68 | 3838.27 | 891.44 | 1970.28 |
| osa-MIR169n | 200.66 | 282.84 | 193.61 | 176.56 | 176.25 | 278.54 | 161.52 | 329.48 | 104.06 | 207.66 |
| osa-MIR169o | 197.41 | 274.96 | 188.38 | 172.75 | 173.01 | 276.84 | 158.11 | 327.36 | 102.81 | 207.66 |
| osa-MIR169p | 3475.43 | 3915.17 | 2257.44 | 2638.93 | 2777.89 | 3294.83 | 2988.08 | 4355.27 | 1884.45 | 3557.32 |
| osa-MIR169q | 2342.95 | 3282.93 | 1517.52 | 1779.97 | 1455.24 | 2461.76 | 2222.58 | 3823.44 | 895.21 | 1973.66 |
| osa-MIR171a | 1824.64 | 1528.06 | 1202.50 | 1155.78 | 1501.32 | 1661.05 | 1916.60 | 2083.88 | 1084.53 | 1489.11 |
| osa-MIR171b | 1428.19 | 1199.68 | 962.84 | 980.17 | 1338.82 | 1352.69 | 1444.56 | 1652.69 | 949.12 | 1261.18 |
| osa-MIR171c | 1423.32 | 1195.30 | 957.61 | 975.40 | 1333.16 | 1345.87 | 1435.46 | 1649.51 | 946.61 | 1261.18 |
| osa-MIR171d | 1346.95 | 1127.88 | 893.77 | 891.41 | 1225.64 | 1254.73 | 1303.52 | 1574.30 | 905.24 | 1200.40 |
| osa-MIR171e | 1382.70 | 1147.14 | 901.09 | 903.82 | 1245.85 | 1271.76 | 1345.60 | 1587.01 | 914.01 | 1220.66 |
| osa-MIR171f | 1332.33 | 1109.49 | 880.16 | 870.42 | 1212.70 | 1241.95 | 1270.53 | 1557.34 | 895.21 | 1198.72 |
| osa-MIR171g | 0.81 | 0.88 | 1.05 | 1.91 | 0.81 | 0.85 | 1.14 | 1.06 | 1.25 | 16.88 |
| osa-MIR171h | 164.92 | 208.41 | 117.22 | 151.75 | 165.74 | 304.95 | 195.64 | 218.24 | 104.06 | 113.12 |
| osa-MIR171i | 602.80 | 541.17 | 413.39 | 392.26 | 386.45 | 557.94 | 843.99 | 720.40 | 240.73 | 383.25 |
| osa-MIR172a | 23470.15 | 17703.63 | 12978.44 | 14083.20 | 19910.11 | 18961.48 | 22410.03 | 20788.96 | 18934.72 | 16874.88 |
| osa-MIR172b | 23128.13 | 17479.46 | 12768.08 | 13852.24 | 19608.55 | 18660.79 | 22114.30 | 20529.40 | 18704.03 | 16630.07 |
| osa-MIR172c | 144.61 | 136.61 | 91.05 | 87.81 | 111.57 | 114.14 | 142.18 | 117.60 | 106.57 | 116.49 |
| osa-MIR172d | 23522.95 | 17780.69 | 13010.89 | 14112.79 | 19953.77 | 19002.36 | 22460.08 | 20840.87 | 18959.80 | 16927.22 |
| osa-MIR319a | 15.44 | 11.38 | 13.61 | 20.04 | 11.32 | 13.63 | 23.89 | 33.90 | 13.79 | 23.64 |
| osa-MIR319b | 30.87 | 49.04 | 42.91 | 58.22 | 21.02 | 35.78 | 52.32 | 151.50 | 45.14 | 69.22 |
| osa-MIR390 | 192.54 | 248.69 | 209.31 | 265.32 | 256.28 | 259.80 | 362.85 | 428.00 | 183.05 | 288.70 |
| osa-MIR393a | 256.72 | 152.37 | 152.80 | 149.84 | 177.86 | 163.55 | 272.99 | 177.98 | 234.46 | 281.95 |
| osa-MIR393b | 489.06 | 287.22 | 323.39 | 271.05 | 385.64 | 367.99 | 478.87 | 420.59 | 588.03 | 660.14 |
| osa-MIR394 | 225.03 | 81.44 | 86.86 | 83.99 | 206.97 | 51.96 | 371.95 | 101.70 | 114.09 | 52.34 |
| osa-MIR395a | 14.62 | 11.38 | 9.42 | 2.86 | 12.13 | 7.67 | 14.79 | 4.24 | 6.27 | 3.38 |
| osa-MIR395b | 19.50 | 20.14 | 11.51 | 5.73 | 18.59 | 9.37 | 20.47 | 9.53 | 11.28 | 6.75 |
| osa-MIR395c | 16.25 | 12.26 | 11.51 | 4.77 | 14.55 | 11.07 | 19.34 | 5.30 | 7.52 | 11.82 |
| osa-MIR395d | 27.62 | 21.02 | 18.84 | 6.68 | 21.02 | 20.44 | 23.89 | 12.71 | 10.03 | 5.06 |
| osa-MIR395e | 21.12 | 15.76 | 15.70 | 6.68 | 19.40 | 15.33 | 21.61 | 7.42 | 7.52 | 5.06 |
| osa-MIR395f | 20.31 | 14.89 | 14.65 | 3.82 | 14.55 | 11.93 | 17.06 | 6.36 | 11.28 | 5.06 |
| osa-MIR395g | 21.12 | 16.64 | 11.51 | 5.73 | 14.55 | 14.48 | 20.47 | 9.53 | 7.52 | 8.44 |
| osa-MIR395h | 16.25 | 14.01 | 10.47 | 2.86 | 13.74 | 7.67 | 15.92 | 5.30 | 6.27 | 3.38 |
| osa-MIR395i | 19.50 | 12.26 | 13.61 | 2.86 | 16.17 | 12.78 | 17.06 | 5.30 | 7.52 | 5.06 |
| osa-MIR395j | 17.06 | 13.14 | 13.61 | 4.77 | 17.79 | 8.52 | 17.06 | 7.42 | 7.52 | 10.13 |
| osa-MIR395k | 19.50 | 14.01 | 12.56 | 2.86 | 16.98 | 16.18 | 17.06 | 6.36 | 7.52 | 5.06 |
| osa-MIR395l | 17.87 | 14.01 | 13.61 | 3.82 | 13.74 | 8.52 | 14.79 | 6.36 | 8.78 | 6.75 |
| osa-MIR395m | 16.25 | 11.38 | 9.42 | 2.86 | 12.13 | 7.67 | 14.79 | 5.30 | 6.27 | 3.38 |

| | | | | | | | | | | |
|---|---|---|---|---|---|---|---|---|---|---|
| osa-MIR395n | 27.62 | 19.26 | 15.70 | 6.68 | 16.98 | 21.30 | 21.61 | 7.42 | 12.54 | 5.06 |
| osa-MIR395o | 14.62 | 11.38 | 9.42 | 2.86 | 12.13 | 9.37 | 15.92 | 5.30 | 6.27 | 3.38 |
| osa-MIR395p | 25.18 | 20.14 | 14.65 | 6.68 | 21.02 | 19.59 | 21.61 | 11.65 | 10.03 | 3.38 |
| osa-MIR395q | 21.12 | 14.89 | 15.70 | 5.73 | 16.17 | 16.18 | 19.34 | 8.48 | 10.03 | 3.38 |
| osa-MIR395r | 18.69 | 13.14 | 12.56 | 3.82 | 18.59 | 13.63 | 20.47 | 7.42 | 7.52 | 3.38 |
| osa-MIR395s | 28.43 | 29.77 | 19.88 | 9.54 | 26.68 | 23.85 | 29.57 | 18.01 | 11.28 | 11.82 |
| osa-MIR395t | 15.44 | 10.51 | 9.42 | 2.86 | 11.32 | 7.67 | 14.79 | 4.24 | 6.27 | 3.38 |
| osa-MIR395u | 0.00 | 1.75 | 1.05 | 0.95 | 0.81 | 1.70 | 1.14 | 0.00 | 1.25 | 1.69 |
| osa-MIR395v | 0.81 | 0.00 | 0.00 | 0.00 | 0.81 | 0.85 | 0.00 | 0.00 | 0.00 | 1.69 |
| osa-MIR395w | 1.62 | 0.88 | 3.14 | 2.86 | 3.23 | 3.41 | 2.27 | 1.06 | 1.25 | 0.00 |
| osa-MIR395x | 0.00 | 0.00 | 0.00 | 0.00 | 0.00 | 0.85 | 0.00 | 0.00 | 0.00 | 0.00 |
| osa-MIR395y | 17.06 | 14.89 | 15.70 | 5.73 | 16.98 | 11.93 | 19.34 | 7.42 | 8.78 | 13.51 |
| osa-MIR396a | 1100.80 | 693.54 | 463.63 | 564.05 | 610.39 | 730.01 | 964.56 | 1157.94 | 546.65 | 771.57 |
| osa-MIR396b | 1100.80 | 693.54 | 463.63 | 564.05 | 610.39 | 729.16 | 964.56 | 1157.94 | 546.65 | 771.57 |
| osa-MIR396c | 1502.93 | 1302.14 | 947.14 | 1099.47 | 1186.83 | 1336.50 | 1649.30 | 1848.68 | 898.97 | 1276.38 |
| osa-MIR396d | 25722.92 | 16059.11 | 11427.44 | 16098.91 | 18889.02 | 19040.70 | 29908.10 | 23921.66 | 12394.96 | 19152.44 |
| osa-MIR396e | 25945.52 | 16208.85 | 11536.28 | 16221.07 | 19030.50 | 19222.13 | 30184.50 | 24073.15 | 12492.76 | 19267.25 |
| osa-MIR396f | 26029.20 | 16248.25 | 11595.93 | 16257.34 | 19064.46 | 19386.53 | 30181.09 | 24208.76 | 12610.61 | 19442.83 |
| osa-MIR396g | 23333.66 | 14479.38 | 10318.08 | 14429.65 | 16996.40 | 17017.63 | 27426.19 | 21815.53 | 11418.26 | 17644.76 |
| osa-MIR396h | 23333.66 | 14480.25 | 10318.08 | 14431.56 | 16994.78 | 17018.48 | 27427.33 | 21816.59 | 11414.50 | 17644.76 |
| osa-MIR396i | 23330.41 | 14477.63 | 10317.03 | 14429.65 | 16993.16 | 17016.78 | 27418.23 | 21813.42 | 11406.97 | 17644.76 |
| osa-MIR397a | 1074.80 | 1006.16 | 1891.14 | 2375.51 | 920.84 | 2517.12 | 2274.90 | 6011.14 | 2161.53 | 2235.35 |
| osa-MIR397b | 1138.98 | 1082.34 | 2024.06 | 2522.49 | 976.63 | 2657.67 | 2395.47 | 6284.47 | 2260.58 | 2360.29 |
| osa-MIR398a | 0.81 | 0.00 | 1.05 | 1.91 | 0.81 | 0.85 | 1.14 | 0.00 | 5.02 | 5.06 |
| osa-MIR398b | 95.05 | 118.22 | 160.12 | 271.05 | 59.02 | 209.55 | 199.05 | 402.58 | 164.25 | 197.53 |
| osa-MIR399a | 333.90 | 648.00 | 550.49 | 498.20 | 261.94 | 610.75 | 746.17 | 1143.11 | 377.39 | 629.75 |
| osa-MIR399b | 358.27 | 677.78 | 575.61 | 528.74 | 296.71 | 774.30 | 797.35 | 1196.08 | 378.64 | 646.63 |
| osa-MIR399c | 335.52 | 651.51 | 551.54 | 502.97 | 262.75 | 615.01 | 751.85 | 1154.77 | 381.15 | 633.12 |
| osa-MIR399d | 893.64 | 1698.82 | 1378.33 | 1140.51 | 507.72 | 1752.19 | 2215.75 | 2864.67 | 773.59 | 1328.72 |
| osa-MIR399e | 34.12 | 80.56 | 61.75 | 50.58 | 33.15 | 80.92 | 98.96 | 141.96 | 55.17 | 77.66 |
| osa-MIR399f | 36.56 | 91.07 | 71.17 | 50.58 | 33.96 | 88.59 | 100.10 | 149.38 | 57.67 | 77.66 |
| osa-MIR399g | 35.75 | 84.94 | 69.07 | 51.54 | 34.76 | 86.03 | 100.10 | 148.32 | 56.42 | 77.66 |
| osa-MIR399h | 30.87 | 78.81 | 60.70 | 52.49 | 32.34 | 78.37 | 97.82 | 139.84 | 53.91 | 75.97 |
| osa-MIR399i | 370.45 | 683.03 | 546.31 | 517.29 | 279.73 | 663.57 | 775.74 | 1142.05 | 356.08 | 617.93 |
| osa-MIR399j | 483.38 | 873.93 | 758.76 | 677.63 | 343.60 | 833.08 | 1036.22 | 1449.28 | 492.74 | 807.02 |
| osa-MIR399k | 0.00 | 3.50 | 12.56 | 0.00 | 0.81 | 5.11 | 4.55 | 1.06 | 2.51 | 3.38 |
| osa-MIR408 | 356.64 | 956.24 | 1710.09 | 2171.27 | 873.95 | 1589.49 | 596.02 | 2238.55 | 581.76 | 405.20 |

| | | | | | | | | | | |
|---|---|---|---|---|---|---|---|---|---|---|
| osa-MIR413 | 0.00 | 0.00 | 0.00 | 0.00 | 0.00 | 0.85 | 0.00 | 0.00 | 2.51 | 0.00 |
| osa-MIR414 | 0.00 | 6.13 | 3.14 | 0.95 | 1.62 | 3.41 | 2.27 | 4.24 | 2.51 | 5.06 |
| osa-MIR415 | 0.00 | 0.00 | 0.00 | 0.00 | 0.00 | 0.00 | 0.00 | 0.00 | 1.25 | 5.06 |
| osa-MIR416 | 0.00 | 0.00 | 0.00 | 0.00 | 1.62 | 0.00 | 0.00 | 3.18 | 0.00 | 3.38 |
| osa-MIR417 | 1.62 | 0.00 | 0.00 | 1.91 | 0.00 | 2.56 | 0.00 | 1.06 | 0.00 | 0.00 |
| osa-MIR418 | 0.00 | 0.00 | 0.00 | 0.00 | 0.00 | 0.00 | 0.00 | 0.00 | 0.00 | 0.00 |
| osa-MIR419 | 0.81 | 0.00 | 0.00 | 2.86 | 0.81 | 0.85 | 7.96 | 1.06 | 1.25 | 0.00 |
| osa-MIR426 | 0.00 | 0.00 | 0.00 | 0.00 | 0.00 | 0.00 | 0.00 | 0.00 | 0.00 | 0.00 |
| osa-MIR435 | 268.90 | 292.48 | 220.83 | 227.15 | 220.71 | 258.95 | 329.86 | 304.05 | 190.58 | 236.37 |
| osa-MIR437 | 45.49 | 51.67 | 41.86 | 56.31 | 54.17 | 69.85 | 46.64 | 91.11 | 26.33 | 75.97 |
| osa-MIR438 | 0.00 | 0.88 | 0.00 | 0.00 | 0.00 | 0.00 | 0.00 | 0.00 | 2.51 | 0.00 |
| osa-MIR439a | 657.23 | 584.08 | 330.71 | 538.28 | 471.34 | 719.79 | 859.91 | 959.83 | 388.67 | 653.38 |
| osa-MIR439b | 335.52 | 299.48 | 170.59 | 290.14 | 234.46 | 392.69 | 469.77 | 546.66 | 203.11 | 342.73 |
| osa-MIR439c | 657.23 | 584.08 | 330.71 | 538.28 | 471.34 | 719.79 | 859.91 | 959.83 | 388.67 | 653.38 |
| osa-MIR439d | 606.86 | 551.68 | 303.50 | 511.56 | 440.61 | 677.20 | 807.59 | 920.63 | 376.14 | 616.24 |
| osa-MIR439e | 661.29 | 589.33 | 335.95 | 541.15 | 480.23 | 727.45 | 866.74 | 965.13 | 389.93 | 658.45 |
| osa-MIR439f | 660.48 | 587.58 | 333.85 | 541.15 | 477.00 | 725.75 | 862.19 | 963.01 | 389.93 | 658.45 |
| osa-MIR439g | 570.30 | 494.76 | 279.43 | 477.20 | 404.23 | 645.68 | 727.97 | 821.05 | 329.75 | 568.97 |
| osa-MIR439h | 647.48 | 578.82 | 329.67 | 535.42 | 468.91 | 719.79 | 862.19 | 952.42 | 387.42 | 653.38 |
| osa-MIR439i | 756.34 | 683.03 | 389.32 | 608.91 | 571.59 | 829.67 | 982.76 | 1089.08 | 448.86 | 759.75 |
| osa-MIR439j | 17.87 | 26.27 | 16.75 | 21.00 | 32.34 | 23.00 | 43.22 | 29.66 | 11.28 | 20.26 |
| osa-MIR440 | 45.49 | 32.40 | 35.58 | 36.27 | 62.25 | 53.66 | 50.05 | 37.08 | 27.58 | 16.88 |
| osa-MIR443 | 17.87 | 12.26 | 12.56 | 21.00 | 16.17 | 16.18 | 22.75 | 16.95 | 16.30 | 6.75 |
| osa-MIR444a | 4104.23 | 3627.07 | 2455.24 | 2578.80 | 2982.43 | 3239.47 | 4087.99 | 3289.49 | 2253.06 | 3434.07 |
| osa-MIR444b | 12708.32 | 10308.51 | 6421.72 | 7651.47 | 9303.03 | 10715.88 | 12957.83 | 15934.71 | 7106.48 | 10916.76 |
| osa-MIR444c | 16744.31 | 14181.65 | 10895.78 | 12121.90 | 11328.24 | 13684.46 | 19444.70 | 22883.43 | 9530.05 | 14344.07 |
| osa-MIR444d | 3654.97 | 2789.04 | 1970.68 | 2055.79 | 2706.75 | 2878.29 | 3357.75 | 2772.50 | 1937.10 | 2947.83 |
| osa-MIR444e | 3093.61 | 2435.27 | 1644.15 | 1674.98 | 2298.47 | 2402.98 | 2945.99 | 2298.94 | 1662.52 | 2562.89 |
| osa-MIR444f | 11986.10 | 9746.32 | 6014.61 | 7052.10 | 8782.38 | 10058.27 | 12334.51 | 15172.98 | 6581.14 | 10280.26 |
| osa-MIR528 | 1070.74 | 6752.37 | 10296.10 | 10056.57 | 6453.99 | 9475.63 | 4591.88 | 22758.42 | 6692.73 | 7087.62 |
| osa-MIR529a | 33.31 | 129.60 | 85.82 | 114.53 | 111.57 | 50.26 | 88.72 | 140.90 | 120.36 | 94.55 |
| osa-MIR529b | 10.56 | 71.81 | 41.86 | 58.22 | 52.55 | 23.85 | 38.67 | 80.52 | 63.94 | 57.40 |
| osa-MIR530 | 1705.22 | 1601.62 | 760.85 | 1560.45 | 1332.35 | 2281.17 | 902.00 | 2936.71 | 1503.29 | 2196.52 |
| osa-MIR531a | 0.00 | 0.00 | 0.00 | 0.00 | 1.62 | 0.00 | 1.14 | 1.06 | 0.00 | 0.00 |
| osa-MIR531b | 0.00 | 0.00 | 0.00 | 0.00 | 0.81 | 0.00 | 1.14 | 0.00 | 0.00 | 0.00 |
| osa-MIR535 | 1548.43 | 2704.98 | 2346.40 | 3611.47 | 2478.76 | 3389.39 | 3954.91 | 6677.51 | 3026.65 | 4031.74 |
| osa-MIR810a | 1203.16 | 789.86 | 728.41 | 759.71 | 804.42 | 904.63 | 1457.07 | 991.62 | 564.21 | 729.36 |

| | | | | | | | | | | |
|---|---|---|---|---|---|---|---|---|---|---|
| osa-MIR810b | 1532.99 | 977.26 | 891.67 | 977.31 | 1073.64 | 1097.99 | 1760.77 | 1216.21 | 722.18 | 937.02 |
| osa-MIR812a | 7949.30 | 6124.51 | 5180.49 | 5668.21 | 7014.26 | 6851.18 | 9948.14 | 7056.78 | 3471.74 | 4674.99 |
| osa-MIR812b | 6437.43 | 5053.55 | 4330.68 | 4755.80 | 5657.65 | 5675.67 | 8256.75 | 6042.92 | 2804.73 | 3942.26 |
| osa-MIR812c | 4962.12 | 3748.79 | 3273.65 | 3545.62 | 4508.01 | 4114.28 | 5745.26 | 3968.58 | 2110.13 | 2718.21 |
| osa-MIR812d | 2351.08 | 1629.64 | 1368.91 | 1601.49 | 1994.49 | 1675.53 | 2305.61 | 1470.47 | 682.06 | 925.21 |
| osa-MIR812e | 9229.64 | 7441.53 | 6419.63 | 7063.55 | 8332.06 | 8441.52 | 11934.12 | 8767.74 | 4355.66 | 5915.92 |
| osa-MIR812f | 1994.43 | 1721.59 | 1271.58 | 1561.41 | 1642.80 | 2017.11 | 2194.14 | 2042.56 | 1260.06 | 1424.95 |
| osa-MIR812g | 3567.23 | 3195.36 | 2423.84 | 2935.75 | 2979.20 | 3805.07 | 4216.53 | 3995.07 | 1993.52 | 2567.95 |
| osa-MIR812h | 2155.29 | 2004.43 | 1485.07 | 1872.54 | 1828.75 | 2339.09 | 2465.99 | 2420.77 | 1332.78 | 1683.27 |
| osa-MIR812i | 2175.60 | 1932.63 | 1428.56 | 1758.97 | 1789.14 | 2356.13 | 2395.47 | 2378.39 | 1261.31 | 1661.32 |
| osa-MIR812j | 2572.86 | 2276.77 | 1665.08 | 2108.28 | 2103.63 | 2722.41 | 2861.82 | 2691.98 | 1464.43 | 1879.11 |
| osa-MIR812k | 17452.72 | 16572.25 | 10745.08 | 13981.08 | 13469.87 | 19527.94 | 20838.08 | 22178.91 | 8292.56 | 14980.57 |
| osa-MIR812l | 13506.10 | 13210.52 | 8083.66 | 10896.44 | 10440.54 | 15549.09 | 15936.81 | 17870.26 | 6286.50 | 12068.20 |
| osa-MIR812m | 20022.33 | 18701.91 | 12495.98 | 16034.96 | 15940.54 | 21922.40 | 24170.81 | 24439.71 | 9591.49 | 16451.11 |
| osa-MIR812n | 2252.78 | 1973.78 | 1601.24 | 1875.41 | 1990.45 | 2405.54 | 2951.68 | 2648.54 | 1061.96 | 1853.79 |
| osa-MIR812o | 1091.86 | 795.12 | 627.94 | 768.30 | 921.65 | 937.85 | 1116.98 | 878.26 | 516.56 | 683.77 |
| osa-MIR812p | 3616.79 | 3282.05 | 2565.13 | 3002.56 | 3056.00 | 3833.18 | 4585.06 | 4109.48 | 1688.85 | 2701.33 |
| osa-MIR812q | 4814.26 | 3658.59 | 3169.00 | 3657.28 | 4254.96 | 4180.72 | 5727.06 | 4442.14 | 2169.06 | 2861.72 |
| osa-MIR812r | 7764.89 | 6196.32 | 5086.30 | 5854.32 | 6828.31 | 7087.13 | 9783.21 | 7199.80 | 3311.26 | 4737.46 |
| osa-MIR812s | 1735.28 | 1237.34 | 917.84 | 1314.22 | 1487.58 | 1373.98 | 1618.59 | 1290.37 | 646.96 | 859.36 |
| osa-MIR812t | 1256.78 | 1017.54 | 872.83 | 982.08 | 1056.67 | 1316.91 | 1552.62 | 1475.77 | 576.74 | 1029.88 |
| osa-MIR812u | 802.65 | 650.63 | 536.89 | 624.18 | 724.39 | 829.67 | 978.21 | 988.44 | 392.44 | 722.61 |
| osa-MIR812v | 346.89 | 304.74 | 223.96 | 289.18 | 290.24 | 347.54 | 399.24 | 354.91 | 181.80 | 258.31 |
| osa-MIR814a | 350.96 | 251.32 | 168.50 | 255.78 | 260.33 | 279.40 | 392.42 | 321.00 | 135.41 | 232.99 |
| osa-MIR814b | 226.66 | 164.63 | 97.33 | 176.56 | 163.31 | 198.47 | 258.20 | 212.94 | 87.77 | 165.46 |
| osa-MIR814c | 277.84 | 218.92 | 178.96 | 244.33 | 255.48 | 236.81 | 299.15 | 276.51 | 134.16 | 194.16 |
| osa-MIR815a | 142.17 | 532.41 | 462.58 | 587.91 | 582.90 | 349.25 | 480.00 | 600.69 | 336.02 | 324.16 |
| osa-MIR815b | 740.09 | 1509.67 | 1420.19 | 1752.29 | 1794.80 | 1310.10 | 1947.31 | 1715.20 | 934.07 | 1104.17 |
| osa-MIR815c | 307.90 | 755.71 | 632.12 | 804.56 | 822.21 | 511.09 | 624.46 | 917.46 | 473.93 | 486.24 |
| osa-MIR816 | 305.46 | 274.96 | 201.99 | 259.60 | 310.45 | 359.47 | 416.31 | 389.87 | 164.25 | 238.05 |
| osa-MIR817 | 67.43 | 49.04 | 38.72 | 56.31 | 69.53 | 69.85 | 89.86 | 63.57 | 31.34 | 32.08 |
| osa-MIR818a | 466.32 | 345.89 | 293.04 | 365.54 | 519.84 | 449.76 | 538.01 | 471.44 | 307.18 | 347.80 |
| osa-MIR818b | 1290.09 | 1058.70 | 871.79 | 1070.84 | 1313.76 | 1414.02 | 1636.79 | 1459.88 | 714.66 | 1060.27 |
| osa-MIR818c | 422.45 | 407.19 | 372.58 | 397.99 | 606.35 | 518.76 | 675.65 | 583.74 | 356.08 | 433.90 |
| osa-MIR818d | 1528.12 | 1993.05 | 1867.07 | 2058.65 | 2632.37 | 2247.95 | 3048.37 | 2663.38 | 1243.76 | 1273.00 |
| osa-MIR818e | 2538.74 | 2113.02 | 1625.31 | 2041.47 | 2316.26 | 2516.27 | 2909.60 | 2824.41 | 1356.60 | 2046.26 |
| osa-MIR818f | 608.49 | 548.18 | 464.67 | 504.88 | 600.69 | 667.83 | 731.38 | 733.12 | 369.87 | 467.67 |

| | | | | | | | | | | |
|---|---|---|---|---|---|---|---|---|---|---|
| osa-MIR820a | 2945.75 | 5544.81 | 3767.63 | 5340.85 | 5507.28 | 5633.93 | 4635.11 | 7247.48 | 3257.34 | 4404.86 |
| osa-MIR820b | 3219.53 | 5749.72 | 3940.31 | 5596.63 | 5785.39 | 5901.40 | 4975.21 | 7522.93 | 3445.41 | 4588.89 |
| osa-MIR820c | 3600.54 | 5968.64 | 4207.19 | 5774.15 | 6038.44 | 6270.24 | 5392.65 | 7871.48 | 3618.44 | 4835.38 |
| osa-MIR821a | 385.89 | 319.62 | 240.71 | 266.28 | 322.58 | 373.10 | 414.03 | 414.23 | 194.34 | 320.78 |
| osa-MIR821b | 195.79 | 162.00 | 126.63 | 145.07 | 165.74 | 182.29 | 205.88 | 215.06 | 97.80 | 135.07 |
| osa-MIR821c | 259.97 | 235.56 | 175.82 | 187.06 | 214.24 | 269.17 | 286.64 | 314.65 | 127.89 | 209.35 |
| osa-MIR827a | 6400.88 | 3817.97 | 8149.60 | 2766.82 | 3172.42 | 3303.35 | 10326.91 | 3050.06 | 6915.90 | 3077.83 |
| osa-MIR827b | 6525.98 | 3919.55 | 8374.61 | 2830.77 | 3241.95 | 3407.27 | 10518.00 | 3165.54 | 7096.45 | 3221.34 |
| osa-MIR827c | 8.12 | 3.50 | 3.14 | 7.64 | 5.66 | 4.26 | 10.24 | 5.30 | 1.25 | 1.69 |
| osa-MIR1317 | 9.75 | 6.13 | 3.14 | 3.82 | 3.23 | 4.26 | 4.55 | 5.30 | 5.02 | 5.06 |
| osa-MIR1318 | 47.93 | 398.43 | 171.64 | 394.17 | 178.67 | 423.35 | 236.59 | 1148.41 | 505.28 | 893.13 |
| osa-MIR1319a | 146.23 | 126.97 | 80.59 | 110.71 | 101.06 | 178.03 | 149.01 | 189.64 | 82.75 | 111.43 |
| osa-MIR1319b | 138.11 | 156.75 | 57.56 | 120.26 | 90.55 | 191.66 | 147.87 | 189.64 | 55.17 | 91.17 |
| osa-MIR1320 | 11.37 | 50.79 | 38.72 | 64.90 | 37.19 | 80.07 | 64.83 | 166.33 | 175.53 | 903.26 |
| osa-MIR1423a | 1252.72 | 1120.00 | 808.99 | 1057.48 | 1093.05 | 1374.83 | 1488.92 | 1414.32 | 672.03 | 1036.64 |
| osa-MIR1423b | 1252.72 | 1120.00 | 808.99 | 1057.48 | 1093.05 | 1374.83 | 1488.92 | 1414.32 | 672.03 | 1036.64 |
| osa-MIR1424 | 0.00 | 0.00 | 0.00 | 0.00 | 0.00 | 0.00 | 0.00 | 0.00 | 0.00 | 0.00 |
| osa-MIR1425 | 16740.25 | 17683.49 | 10402.85 | 14819.05 | 14117.45 | 16328.51 | 11434.78 | 7195.57 | 12313.46 | 12004.04 |
| osa-MIR1426 | 17.87 | 10.51 | 11.51 | 8.59 | 15.36 | 6.81 | 12.51 | 9.53 | 5.02 | 6.75 |
| osa-MIR1427 | 262.40 | 227.68 | 124.54 | 149.84 | 219.90 | 214.66 | 310.52 | 129.25 | 165.50 | 143.51 |
| osa-MIR1428a | 3.25 | 0.88 | 2.09 | 0.00 | 2.43 | 3.41 | 2.27 | 1.06 | 0.00 | 1.69 |
| osa-MIR1428b | 0.00 | 0.88 | 1.05 | 1.91 | 1.62 | 1.70 | 0.00 | 0.00 | 0.00 | 1.69 |
| osa-MIR1428c | 1.62 | 0.88 | 1.05 | 1.91 | 3.23 | 1.70 | 0.00 | 1.06 | 0.00 | 1.69 |
| osa-MIR1428d | 3.25 | 1.75 | 3.14 | 1.91 | 3.23 | 2.56 | 2.27 | 1.06 | 0.00 | 1.69 |
| osa-MIR1428e | 2.44 | 1.75 | 2.09 | 2.86 | 3.23 | 5.11 | 1.14 | 2.12 | 2.51 | 1.69 |
| osa-MIR1428f | 2.44 | 0.88 | 1.05 | 0.95 | 1.62 | 1.70 | 0.00 | 2.12 | 1.25 | 1.69 |
| osa-MIR1428g | 4.06 | 0.88 | 3.14 | 0.95 | 2.43 | 2.56 | 2.27 | 1.06 | 0.00 | 1.69 |
| osa-MIR1429 | 186.85 | 161.13 | 104.66 | 140.30 | 156.03 | 208.70 | 244.55 | 204.47 | 89.02 | 140.13 |
| osa-MIR1430 | 284.34 | 399.31 | 303.50 | 293.96 | 333.90 | 366.28 | 229.76 | 368.68 | 195.59 | 378.19 |
| osa-MIR1431 | 75.55 | 67.43 | 46.05 | 29.59 | 42.85 | 78.37 | 85.31 | 79.46 | 41.38 | 75.97 |
| osa-MIR1432 | 47.93 | 397.56 | 171.64 | 394.17 | 178.67 | 422.50 | 236.59 | 1147.35 | 504.02 | 891.44 |
| osa-MIR1433 | 1083.74 | 1315.27 | 732.60 | 822.70 | 714.68 | 1099.70 | 800.76 | 1816.90 | 504.02 | 1058.58 |
| osa-MIR1435 | 4.06 | 0.00 | 0.00 | 0.95 | 1.62 | 0.85 | 1.14 | 0.00 | 2.51 | 0.00 |
| osa-MIR1436 | 584.93 | 515.78 | 637.36 | 528.74 | 742.98 | 621.83 | 1031.67 | 808.34 | 476.44 | 406.89 |
| osa-MIR1437 | 180.35 | 199.66 | 168.50 | 107.85 | 211.01 | 169.51 | 257.06 | 209.76 | 131.65 | 92.86 |
| osa-MIR1438 | 0.81 | 0.00 | 0.00 | 0.00 | 0.00 | 0.00 | 0.00 | 0.00 | 0.00 | 0.00 |
| osa-MIR1439 | 286.78 | 309.12 | 235.48 | 266.28 | 301.56 | 352.65 | 432.23 | 406.82 | 164.25 | 248.18 |

| | | | | | | | | | | |
|---|---|---|---|---|---|---|---|---|---|---|
| osa-MIR1440a | 79.61 | 103.33 | 68.03 | 97.35 | 106.72 | 97.11 | 111.47 | 125.01 | 77.73 | 89.48 |
| osa-MIR1440b | 79.61 | 103.33 | 68.03 | 97.35 | 106.72 | 97.11 | 111.47 | 125.01 | 77.73 | 89.48 |
| osa-MIR1441 | 4039.24 | 2715.49 | 2148.60 | 2787.82 | 3309.86 | 3219.87 | 4062.97 | 3222.75 | 1841.82 | 2448.08 |
| osa-MIR1442 | 540.24 | 646.25 | 586.08 | 655.68 | 861.02 | 775.15 | 1030.53 | 838.00 | 433.81 | 422.08 |
| osa-MIR1846a | 2.44 | 7.01 | 0.00 | 18.13 | 3.23 | 12.78 | 4.55 | 8.48 | 7.52 | 8.44 |
| osa-MIR1846b | 2.44 | 7.01 | 0.00 | 18.13 | 1.62 | 11.93 | 4.55 | 7.42 | 3.76 | 3.38 |
| osa-MIR1846c | 0.00 | 4.38 | 1.05 | 13.36 | 1.62 | 12.78 | 1.14 | 7.42 | 3.76 | 8.44 |
| osa-MIR1846d | 20.31 | 21.89 | 18.84 | 19.09 | 25.06 | 29.81 | 32.99 | 28.60 | 28.84 | 50.65 |
| osa-MIR1846e | 326.58 | 239.94 | 354.79 | 249.10 | 327.43 | 318.58 | 342.37 | 252.14 | 240.73 | 197.53 |
| osa-MIR1847 | 50.37 | 55.17 | 23.02 | 62.04 | 41.23 | 65.59 | 48.91 | 90.05 | 37.61 | 70.91 |
| osa-MIR1848 | 0.81 | 0.88 | 0.00 | 1.91 | 1.62 | 0.00 | 3.41 | 1.06 | 2.51 | 0.00 |
| osa-MIR1849 | 87.74 | 61.30 | 56.51 | 62.99 | 76.80 | 88.59 | 135.36 | 102.76 | 60.18 | 72.60 |
| osa-MIR1850 | 140.54 | 178.64 | 152.80 | 197.56 | 176.25 | 220.62 | 144.46 | 422.71 | 481.46 | 884.69 |
| osa-MIR1851 | 3805.27 | 2909.01 | 2326.51 | 2282.94 | 2766.57 | 2876.59 | 6511.90 | 3091.38 | 2047.44 | 1809.89 |
| osa-MIR1852 | 4.87 | 0.00 | 2.09 | 0.95 | 0.81 | 0.85 | 1.14 | 4.24 | 0.00 | 1.69 |
| osa-MIR1853 | 0.00 | 0.00 | 0.00 | 1.91 | 0.00 | 0.00 | 0.00 | 0.00 | 0.00 | 0.00 |
| osa-MIR1854 | 3.25 | 3.50 | 1.05 | 4.77 | 0.00 | 3.41 | 5.69 | 1.06 | 11.28 | 5.06 |
| osa-MIR1855 | 0.81 | 1.75 | 0.00 | 0.00 | 1.62 | 0.00 | 0.00 | 1.06 | 0.00 | 1.69 |
| osa-MIR1856 | 7.31 | 4.38 | 1.05 | 5.73 | 6.47 | 2.56 | 6.82 | 7.42 | 8.78 | 6.75 |
| osa-MIR1857 | 117.80 | 106.83 | 127.68 | 85.90 | 105.10 | 119.25 | 172.89 | 161.03 | 95.29 | 126.62 |
| osa-MIR1858a | 0.00 | 1.75 | 1.05 | 0.95 | 3.23 | 2.56 | 4.55 | 0.00 | 1.25 | 0.00 |
| osa-MIR1858b | 0.00 | 0.88 | 0.00 | 0.00 | 1.62 | 0.85 | 1.14 | 0.00 | 2.51 | 0.00 |
| osa-MIR1859 | 0.00 | 0.00 | 0.00 | 1.91 | 0.00 | 0.00 | 0.00 | 0.00 | 1.25 | 0.00 |
| osa-MIR1860 | 164.10 | 148.87 | 97.33 | 127.89 | 144.72 | 224.88 | 230.90 | 224.60 | 71.47 | 157.01 |
| osa-MIR1861a | 142.17 | 114.71 | 86.86 | 152.70 | 104.29 | 123.51 | 89.86 | 237.31 | 91.53 | 155.33 |
| osa-MIR1861b | 75.55 | 51.67 | 42.91 | 52.49 | 34.76 | 51.96 | 43.22 | 99.59 | 27.58 | 40.52 |
| osa-MIR1861c | 1.62 | 0.88 | 2.09 | 2.86 | 1.62 | 0.85 | 0.00 | 2.12 | 0.00 | 0.00 |
| osa-MIR1861d | 72.30 | 48.16 | 39.77 | 50.58 | 33.15 | 44.29 | 36.40 | 95.35 | 26.33 | 40.52 |
| osa-MIR1861e | 0.81 | 1.75 | 2.09 | 2.86 | 2.43 | 0.85 | 0.00 | 2.12 | 0.00 | 0.00 |
| osa-MIR1861f | 73.93 | 49.91 | 42.91 | 52.49 | 34.76 | 50.26 | 40.95 | 97.47 | 27.58 | 38.83 |
| osa-MIR1861g | 1.62 | 0.00 | 1.05 | 0.95 | 0.00 | 0.00 | 0.00 | 0.00 | 0.00 | 0.00 |
| osa-MIR1861h | 73.93 | 49.91 | 45.00 | 51.54 | 32.34 | 45.15 | 36.40 | 95.35 | 26.33 | 43.90 |
| osa-MIR1861i | 73.12 | 52.54 | 41.86 | 54.40 | 33.15 | 48.55 | 42.09 | 97.47 | 26.33 | 40.52 |
| osa-MIR1861j | 72.30 | 49.04 | 39.77 | 49.63 | 31.53 | 44.29 | 35.26 | 94.29 | 25.08 | 42.21 |
| osa-MIR1861k | 0.81 | 1.75 | 2.09 | 2.86 | 2.43 | 0.85 | 0.00 | 2.12 | 0.00 | 0.00 |
| osa-MIR1861l | 73.93 | 52.54 | 42.91 | 52.49 | 35.57 | 50.26 | 40.95 | 98.53 | 26.33 | 42.21 |
| osa-MIR1861m | 0.81 | 1.75 | 3.14 | 2.86 | 1.62 | 0.85 | 0.00 | 2.12 | 0.00 | 0.00 |

| | | | | | | | | | | |
|---|---|---|---|---|---|---|---|---|---|---|
| osa-MIR1861n | 2.44 | 1.75 | 3.14 | 0.95 | 2.43 | 1.70 | 6.82 | 6.36 | 3.76 | 1.69 |
| osa-MIR1862a | 1354.27 | 1570.10 | 1040.28 | 1319.94 | 1722.03 | 2074.18 | 1859.73 | 2903.86 | 1524.61 | 1931.45 |
| osa-MIR1862b | 1420.88 | 1567.47 | 996.33 | 1304.67 | 1713.95 | 2082.70 | 1884.75 | 2904.92 | 1535.89 | 1944.96 |
| osa-MIR1862c | 1308.77 | 1376.57 | 843.53 | 1140.51 | 1538.51 | 1867.19 | 1697.08 | 2605.11 | 1391.71 | 1700.15 |
| osa-MIR1862d | 5975.99 | 5236.57 | 4186.26 | 5485.92 | 6462.08 | 7455.97 | 8162.34 | 8368.34 | 4402.05 | 5618.77 |
| osa-MIR1862e | 5327.70 | 4702.40 | 3799.03 | 5044.99 | 5322.14 | 6639.07 | 7459.40 | 7391.56 | 3429.11 | 4852.27 |
| osa-MIR1862f | 662.92 | 673.40 | 273.15 | 373.17 | 898.21 | 960.00 | 644.93 | 1316.86 | 1030.61 | 1109.23 |
| osa-MIR1862g | 1145.48 | 937.85 | 529.56 | 757.80 | 1291.93 | 1349.28 | 1151.10 | 1680.24 | 1174.80 | 1289.89 |
| osa-MIR1863a | 585.74 | 670.77 | 409.21 | 623.23 | 612.82 | 810.08 | 880.39 | 1017.04 | 373.63 | 661.83 |
| osa-MIR1863b | 295.71 | 314.37 | 204.08 | 232.87 | 265.99 | 458.28 | 481.14 | 604.93 | 150.45 | 307.28 |
| osa-MIR1863c | 322.52 | 339.76 | 227.10 | 257.69 | 286.20 | 478.72 | 515.26 | 629.29 | 175.53 | 325.85 |
| osa-MIR1864 | 32.50 | 36.78 | 37.68 | 35.31 | 38.81 | 48.55 | 52.32 | 42.38 | 43.88 | 54.03 |
| osa-MIR1865 | 6.50 | 10.51 | 6.28 | 13.36 | 4.04 | 17.89 | 9.10 | 4.24 | 5.02 | 10.13 |
| osa-MIR1866 | 0.00 | 1.75 | 1.05 | 1.91 | 0.00 | 0.85 | 1.14 | 1.06 | 1.25 | 1.69 |
| osa-MIR1867 | 554.05 | 761.84 | 536.89 | 744.44 | 726.00 | 818.60 | 895.17 | 826.35 | 511.55 | 734.42 |
| osa-MIR1868 | 275.40 | 459.73 | 299.32 | 434.25 | 423.64 | 445.50 | 474.32 | 590.10 | 312.19 | 471.04 |
| osa-MIR1869 | 1.62 | 2.63 | 1.05 | 2.86 | 3.23 | 2.56 | 3.41 | 4.24 | 2.51 | 0.00 |
| osa-MIR1870 | 3059.49 | 2475.55 | 2047.08 | 2661.84 | 2328.38 | 3253.09 | 3611.40 | 3666.65 | 1627.42 | 2699.64 |
| osa-MIR1871 | 432.20 | 347.65 | 237.57 | 277.73 | 291.86 | 487.24 | 445.88 | 568.91 | 194.34 | 521.69 |
| osa-MIR1872 | 44.68 | 42.03 | 19.88 | 34.36 | 35.57 | 43.44 | 58.01 | 38.14 | 13.79 | 32.08 |
| osa-MIR1873 | 380.20 | 481.62 | 290.94 | 591.73 | 378.36 | 553.68 | 482.28 | 670.61 | 278.34 | 467.67 |
| osa-MIR1874 | 0.00 | 0.00 | 1.05 | 0.95 | 0.00 | 0.00 | 1.14 | 0.00 | 1.25 | 0.00 |
| osa-MIR1875 | 55.24 | 151.49 | 127.68 | 167.02 | 127.74 | 155.03 | 158.11 | 244.73 | 96.54 | 123.25 |
| osa-MIR1876 | 90.18 | 82.31 | 50.24 | 110.71 | 57.40 | 103.07 | 119.43 | 102.76 | 43.88 | 70.91 |
| osa-MIR1877 | 43.87 | 25.39 | 25.12 | 31.50 | 33.15 | 42.59 | 43.22 | 30.72 | 15.05 | 21.95 |
| osa-MIR1878 | 31.68 | 27.15 | 15.70 | 39.13 | 16.17 | 47.70 | 34.12 | 41.32 | 17.55 | 55.71 |
| osa-MIR1879 | 31.68 | 49.91 | 41.86 | 56.31 | 54.17 | 65.59 | 61.42 | 144.08 | 60.18 | 59.09 |
| osa-MIR1880 | 40.62 | 35.03 | 33.49 | 50.58 | 29.91 | 38.33 | 76.21 | 50.85 | 16.30 | 16.88 |
| osa-MIR1881 | 35.75 | 40.28 | 23.02 | 55.36 | 38.81 | 42.59 | 25.02 | 57.21 | 16.30 | 47.27 |
| osa-MIR1882a | 8.12 | 8.76 | 2.09 | 2.86 | 0.00 | 2.56 | 3.41 | 6.36 | 2.51 | 5.06 |
| osa-MIR1882b | 10.56 | 7.01 | 3.14 | 6.68 | 4.04 | 5.11 | 5.69 | 6.36 | 3.76 | 6.75 |
| osa-MIR1882c | 12.19 | 6.13 | 4.19 | 3.82 | 1.62 | 2.56 | 3.41 | 7.42 | 2.51 | 6.75 |
| osa-MIR1882d | 13.81 | 7.88 | 3.14 | 5.73 | 2.43 | 4.26 | 3.41 | 8.48 | 5.02 | 5.06 |
| osa-MIR1882e | 7.31 | 5.25 | 3.14 | 1.91 | 2.43 | 1.70 | 4.55 | 5.30 | 3.76 | 5.06 |
| osa-MIR1882f | 6.50 | 4.38 | 1.05 | 3.82 | 2.43 | 3.41 | 1.14 | 2.12 | 2.51 | 5.06 |
| osa-MIR1882g | 10.56 | 7.88 | 5.23 | 6.68 | 1.62 | 4.26 | 4.55 | 8.48 | 5.02 | 5.06 |
| osa-MIR1882h | 12.19 | 10.51 | 3.14 | 4.77 | 3.23 | 8.52 | 4.55 | 9.53 | 6.27 | 6.75 |

| | | | | | | | | | | |
|---|---|---|---|---|---|---|---|---|---|---|
| osa-MIR1883a | 20.31 | 12.26 | 15.70 | 17.18 | 19.40 | 15.33 | 20.47 | 16.95 | 28.84 | 13.51 |
| osa-MIR1883b | 129.98 | 115.59 | 148.61 | 107.85 | 223.14 | 113.29 | 283.22 | 125.01 | 131.65 | 52.34 |
| osa-MIR2055 | 362.33 | 258.33 | 161.17 | 227.15 | 259.52 | 323.69 | 324.17 | 334.78 | 213.14 | 398.45 |
| osa-MIR2090 | 0.00 | 1.75 | 0.00 | 0.00 | 0.00 | 0.00 | 0.00 | 0.00 | 0.00 | 1.69 |
| osa-MIR2091 | 0.00 | 0.00 | 0.00 | 0.00 | 0.81 | 0.00 | 0.00 | 0.00 | 0.00 | 0.00 |
| osa-MIR2092 | 4.06 | 3.50 | 4.19 | 3.82 | 2.43 | 3.41 | 5.69 | 1.06 | 0.00 | 5.06 |
| osa-MIR2093 | 0.00 | 0.00 | 0.00 | 0.00 | 0.00 | 0.85 | 0.00 | 0.00 | 0.00 | 0.00 |
| osa-MIR2094 | 0.81 | 0.00 | 0.00 | 0.00 | 0.00 | 0.00 | 0.00 | 0.00 | 0.00 | 0.00 |
| osa-MIR2095 | 0.00 | 0.00 | 0.00 | 0.00 | 0.00 | 0.00 | 0.00 | 1.06 | 0.00 | 0.00 |
| osa-MIR2096 | 10.56 | 9.63 | 11.51 | 9.54 | 9.70 | 14.48 | 13.65 | 14.83 | 10.03 | 6.75 |
| osa-MIR2097 | 5.69 | 4.38 | 6.28 | 5.73 | 7.28 | 6.81 | 4.55 | 2.12 | 1.25 | 10.13 |
| osa-MIR2098 | 5.69 | 4.38 | 2.09 | 2.86 | 3.23 | 4.26 | 3.41 | 7.42 | 1.25 | 1.69 |
| osa-MIR2099 | 0.00 | 0.00 | 0.00 | 0.00 | 0.00 | 0.00 | 0.00 | 0.00 | 0.00 | 0.00 |
| osa-MIR2100 | 6.50 | 4.38 | 6.28 | 3.82 | 8.08 | 11.07 | 7.96 | 8.48 | 6.27 | 6.75 |
| osa-MIR2101 | 0.00 | 0.00 | 1.05 | 0.00 | 0.00 | 0.00 | 2.27 | 0.00 | 0.00 | 0.00 |
| osa-MIR2102 | 2.44 | 3.50 | 0.00 | 4.77 | 0.00 | 6.81 | 3.41 | 10.59 | 1.25 | 5.06 |
| osa-MIR2103 | 53.62 | 132.23 | 68.03 | 91.62 | 69.53 | 115.85 | 87.58 | 191.75 | 52.66 | 119.87 |
| osa-MIR2104 | 0.00 | 0.00 | 0.00 | 0.95 | 0.00 | 0.00 | 0.00 | 0.00 | 0.00 | 0.00 |
| osa-MIR2105 | 0.81 | 0.00 | 0.00 | 0.00 | 0.81 | 1.70 | 0.00 | 1.06 | 1.25 | 0.00 |
| osa-MIR2106 | 20.31 | 45.54 | 13.61 | 10.50 | 12.13 | 15.33 | 6.82 | 7.42 | 15.05 | 10.13 |
| osa-MIR2118a | 6.50 | 0.00 | 3.14 | 0.95 | 1.62 | 1.70 | 1.14 | 1.06 | 3.76 | 0.00 |
| osa-MIR2118b | 7.31 | 4.38 | 4.19 | 5.73 | 5.66 | 5.96 | 5.69 | 8.48 | 8.78 | 6.75 |
| osa-MIR2118c | 5.69 | 1.75 | 4.19 | 0.95 | 2.43 | 3.41 | 2.27 | 1.06 | 3.76 | 0.00 |
| osa-MIR2118d | 8.12 | 5.25 | 7.33 | 6.68 | 7.28 | 8.52 | 7.96 | 9.53 | 7.52 | 8.44 |
| osa-MIR2118e | 7.31 | 23.64 | 18.84 | 14.32 | 21.83 | 11.07 | 19.34 | 27.54 | 26.33 | 18.57 |
| osa-MIR2118f | 7.31 | 7.01 | 7.33 | 9.54 | 6.47 | 2.56 | 4.55 | 13.77 | 7.52 | 6.75 |
| osa-MIR2118g | 4.87 | 8.76 | 4.19 | 10.50 | 4.85 | 2.56 | 3.41 | 13.77 | 3.76 | 10.13 |
| osa-MIR2118h | 0.81 | 2.63 | 0.00 | 3.82 | 1.62 | 0.85 | 4.55 | 6.36 | 3.76 | 3.38 |
| osa-MIR2118i | 7.31 | 7.88 | 7.33 | 8.59 | 12.13 | 5.96 | 6.82 | 15.89 | 3.76 | 8.44 |
| osa-MIR2118j | 26.00 | 40.28 | 27.21 | 53.45 | 40.42 | 54.52 | 52.32 | 70.98 | 27.58 | 50.65 |
| osa-MIR2118k | 1.62 | 2.63 | 0.00 | 3.82 | 3.23 | 1.70 | 4.55 | 7.42 | 5.02 | 3.38 |
| osa-MIR2118l | 0.81 | 4.38 | 6.28 | 4.77 | 3.23 | 0.00 | 2.27 | 6.36 | 2.51 | 5.06 |
| osa-MIR2118m | 26.00 | 42.91 | 25.12 | 53.45 | 41.23 | 57.92 | 55.74 | 74.16 | 30.09 | 48.96 |
| osa-MIR2118n | 8.12 | 5.25 | 5.23 | 5.73 | 5.66 | 5.11 | 5.69 | 8.48 | 7.52 | 6.75 |
| osa-MIR2118o | 6.50 | 3.50 | 10.47 | 4.77 | 8.08 | 7.67 | 4.55 | 4.24 | 2.51 | 6.75 |
| osa-MIR2118p | 10.56 | 7.88 | 5.23 | 10.50 | 11.32 | 6.81 | 6.82 | 10.59 | 6.27 | 1.69 |
| osa-MIR2118q | 14.62 | 9.63 | 15.70 | 11.45 | 8.89 | 11.93 | 15.92 | 18.01 | 10.03 | 8.44 |

| | | | | | | | | | | |
|---|---|---|---|---|---|---|---|---|---|---|
| osa-MIR2118r | 22.75 | 42.91 | 25.12 | 49.63 | 38.00 | 57.07 | 53.46 | 73.10 | 23.82 | 48.96 |
| osa-MIR2120 | 56.06 | 21.02 | 21.98 | 36.27 | 46.89 | 29.81 | 48.91 | 42.38 | 20.06 | 28.70 |
| osa-MIR2121a | 298.96 | 211.04 | 176.87 | 189.93 | 223.14 | 184.84 | 315.07 | 225.66 | 126.63 | 182.34 |
| osa-MIR2121b | 296.52 | 211.04 | 168.50 | 194.70 | 223.95 | 189.96 | 310.52 | 224.60 | 120.36 | 173.90 |
| osa-MIR2122 | 204.72 | 124.35 | 95.24 | 117.39 | 169.78 | 152.48 | 226.35 | 174.80 | 101.56 | 123.25 |
| osa-MIR2275a | 0.81 | 0.00 | 0.00 | 0.00 | 0.00 | 0.00 | 0.00 | 0.00 | 0.00 | 0.00 |
| osa-MIR2275b | 0.81 | 0.00 | 0.00 | 0.00 | 0.00 | 0.00 | 0.00 | 0.00 | 0.00 | 0.00 |
| osa-MIR2275c | 5.69 | 2.63 | 2.09 | 1.91 | 3.23 | 5.11 | 4.55 | 9.53 | 1.25 | 5.06 |
| osa-MIR2275d | 7.31 | 2.63 | 2.09 | 1.91 | 3.23 | 5.11 | 4.55 | 9.53 | 3.76 | 6.75 |
| osa-MIR2863a | 6.50 | 7.01 | 3.14 | 2.86 | 4.04 | 7.67 | 13.65 | 3.18 | 5.02 | 1.69 |
| osa-MIR2863b | 13.81 | 10.51 | 7.33 | 13.36 | 11.32 | 10.22 | 9.10 | 25.43 | 8.78 | 20.26 |
| osa-MIR2863c | 59.30 | 71.81 | 49.19 | 65.85 | 73.57 | 81.77 | 70.52 | 64.62 | 31.34 | 38.83 |
| osa-MIR2864 | 72.30 | 6806.67 | 40.82 | 5735.98 | 54.98 | 7083.72 | 54.60 | 7912.79 | 37.61 | 5556.30 |
| osa-MIR2865 | 28.43 | 104.21 | 12.56 | 122.16 | 19.40 | 124.37 | 18.20 | 120.77 | 10.03 | 89.48 |
| osa-MIR2866 | 43.06 | 70.05 | 65.93 | 62.99 | 59.02 | 85.18 | 60.28 | 146.20 | 55.17 | 101.30 |
| osa-MIR2867 | 638.54 | 495.64 | 351.65 | 339.77 | 434.96 | 580.09 | 603.99 | 642.01 | 282.10 | 440.65 |
| osa-MIR2868 | 0.00 | 0.00 | 0.00 | 0.00 | 0.00 | 0.00 | 0.00 | 0.00 | 0.00 | 1.69 |
| osa-MIR2869 | 32.50 | 103.33 | 173.73 | 166.07 | 78.42 | 105.63 | 219.53 | 583.74 | 156.72 | 357.93 |
| osa-MIR2870 | 13.00 | 17.51 | 15.70 | 20.04 | 7.28 | 4.26 | 18.20 | 22.25 | 16.30 | 18.57 |
| osa-MIR2871a | 228.28 | 197.90 | 132.91 | 146.98 | 118.04 | 187.40 | 229.76 | 183.28 | 87.77 | 177.27 |
| osa-MIR2871b | 220.16 | 194.40 | 127.68 | 144.12 | 116.42 | 179.73 | 221.80 | 180.10 | 85.26 | 175.59 |
| osa-MIR2872 | 73.93 | 96.32 | 41.86 | 102.12 | 67.91 | 126.92 | 79.62 | 208.71 | 68.96 | 94.55 |
| osa-MIR2873a | 1412.76 | 1105.11 | 700.15 | 880.92 | 721.15 | 1249.62 | 944.08 | 1375.12 | 778.60 | 808.71 |
| osa-MIR2873b | 419.20 | 243.44 | 180.01 | 214.74 | 231.22 | 264.06 | 385.60 | 311.47 | 176.78 | 253.25 |
| osa-MIR2873c | 4.06 | 5.25 | 2.09 | 4.77 | 7.28 | 5.11 | 9.10 | 1.06 | 2.51 | 1.69 |
| osa-MIR2874 | 38.18 | 30.65 | 25.12 | 42.95 | 40.42 | 59.63 | 40.95 | 46.61 | 22.57 | 18.57 |
| osa-MIR2875 | 109.67 | 91.95 | 56.51 | 91.62 | 72.76 | 143.96 | 89.86 | 107.00 | 33.85 | 69.22 |
| osa-MIR2876 | 0.00 | 38.53 | 0.00 | 30.54 | 0.81 | 76.66 | 0.00 | 61.45 | 1.25 | 60.78 |
| osa-MIR2877 | 23.56 | 21.89 | 15.70 | 15.27 | 13.74 | 20.44 | 14.79 | 22.25 | 12.54 | 20.26 |
| osa-MIR2878 | 238.84 | 190.90 | 166.40 | 204.24 | 148.76 | 232.55 | 261.61 | 289.22 | 150.45 | 177.27 |
| osa-MIR2879 | 3.25 | 3.50 | 1.05 | 0.00 | 3.23 | 4.26 | 4.55 | 4.24 | 0.00 | 3.38 |
| osa-MIR2880 | 168.98 | 112.09 | 77.45 | 125.03 | 146.33 | 146.51 | 163.79 | 116.54 | 70.21 | 91.17 |
| osa-MIR2905 | 34.12 | 7.88 | 16.75 | 16.22 | 20.21 | 16.18 | 21.61 | 13.77 | 13.79 | 21.95 |
| osa-MIR2907a | 0.81 | 0.00 | 0.00 | 0.00 | 0.81 | 0.00 | 2.27 | 1.06 | 0.00 | 0.00 |
| osa-MIR2907b | 2.44 | 0.00 | 0.00 | 0.95 | 1.62 | 0.85 | 3.41 | 1.06 | 1.25 | 0.00 |
| osa-MIR2907c | 4.87 | 5.25 | 0.00 | 3.82 | 5.66 | 3.41 | 4.55 | 2.12 | 1.25 | 1.69 |
| osa-MIR2907d | 0.00 | 0.00 | 0.00 | 0.00 | 0.00 | 0.00 | 1.14 | 1.06 | 0.00 | 0.00 |

| | | | | | | | | | | |
|---|---|---|---|---|---|---|---|---|---|---|
| osa-MIR2918 | 3.25 | 3.50 | 0.00 | 0.95 | 7.28 | 7.67 | 1.14 | 2.12 | 1.25 | 1.69 |
| osa-MIR2919 | 3.25 | 6.13 | 4.19 | 2.86 | 4.04 | 4.26 | 7.96 | 9.53 | 2.51 | 1.69 |
| osa-MIR2920 | 0.00 | 0.00 | 0.00 | 0.00 | 0.00 | 0.00 | 0.00 | 0.00 | 0.00 | 0.00 |
| osa-MIR2921 | 99.92 | 74.43 | 63.84 | 71.58 | 75.19 | 83.48 | 110.33 | 84.75 | 36.36 | 70.91 |
| osa-MIR2922 | 0.00 | 0.88 | 0.00 | 0.00 | 0.00 | 0.00 | 0.00 | 0.00 | 0.00 | 0.00 |
| osa-MIR2923 | 0.81 | 0.00 | 0.00 | 0.00 | 0.81 | 0.00 | 1.14 | 0.00 | 0.00 | 0.00 |
| osa-MIR2924 | 0.00 | 0.88 | 1.05 | 0.95 | 0.00 | 0.85 | 1.14 | 1.06 | 0.00 | 0.00 |
| osa-MIR2925 | 18.69 | 13.14 | 1.05 | 2.86 | 10.51 | 5.96 | 4.55 | 1.06 | 7.52 | 8.44 |
| osa-MIR2926 | 0.00 | 0.00 | 1.05 | 0.00 | 0.00 | 0.00 | 0.00 | 0.00 | 1.25 | 0.00 |
| osa-MIR2927 | 0.81 | 0.00 | 0.00 | 0.95 | 0.00 | 0.00 | 0.00 | 2.12 | 1.25 | 0.00 |
| osa-MIR2928 | 0.00 | 0.00 | 0.00 | 0.00 | 0.00 | 0.00 | 1.14 | 0.00 | 1.25 | 1.69 |
| osa-MIR2929 | 0.00 | 0.00 | 0.00 | 0.00 | 0.00 | 0.00 | 0.00 | 0.00 | 0.00 | 0.00 |
| osa-MIR2930 | 0.00 | 1.75 | 0.00 | 0.95 | 0.81 | 0.85 | 0.00 | 3.18 | 0.00 | 0.00 |
| osa-MIR2931 | 11.37 | 3.50 | 3.14 | 6.68 | 4.04 | 4.26 | 5.69 | 10.59 | 5.02 | 5.06 |
| osa-MIR2932 | 8.12 | 7.01 | 5.23 | 2.86 | 0.81 | 7.67 | 6.82 | 6.36 | 1.25 | 5.06 |
| osa-MIR3979 | 10.56 | 14.89 | 10.47 | 25.77 | 8.89 | 13.63 | 10.24 | 7.42 | 6.27 | 20.26 |
| osa-MIR3980a | 386.70 | 434.34 | 229.20 | 274.87 | 285.39 | 485.54 | 379.91 | 563.61 | 303.42 | 401.82 |
| osa-MIR3980b | 386.70 | 431.71 | 229.20 | 273.91 | 286.20 | 483.83 | 379.91 | 563.61 | 303.42 | 401.82 |
| osa-MIR3981 | 368.83 | 263.58 | 226.06 | 263.42 | 328.24 | 417.39 | 456.12 | 375.03 | 161.74 | 229.61 |
| osa-MIR3982 | 48.74 | 32.40 | 37.68 | 27.68 | 48.51 | 41.74 | 48.91 | 41.32 | 57.67 | 40.52 |
| osa-MIR5071 | 0.00 | 0.00 | 0.00 | 0.00 | 0.00 | 0.00 | 1.14 | 0.00 | 0.00 | 0.00 |
| osa-MIR5072 | 203.10 | 148.87 | 249.08 | 230.97 | 287.01 | 204.44 | 135.36 | 242.61 | 272.07 | 163.77 |
| osa-MIR5073 | 36.56 | 21.89 | 12.56 | 12.41 | 21.83 | 22.15 | 12.51 | 18.01 | 22.57 | 28.70 |
| osa-MIR5074 | 38.18 | 21.02 | 25.12 | 30.54 | 44.47 | 40.89 | 68.25 | 37.08 | 21.31 | 27.01 |
| osa-MIR5075 | 19.50 | 28.02 | 28.26 | 29.59 | 54.98 | 61.33 | 27.30 | 44.50 | 36.36 | 25.32 |
| osa-MIR5076 | 26.81 | 55.17 | 25.12 | 21.95 | 39.61 | 24.70 | 13.65 | 14.83 | 32.60 | 13.51 |
| osa-MIR5077 | 16583.46 | 13253.43 | 17342.62 | 25564.70 | 20743.64 | 19711.08 | 23283.60 | 14666.58 | 16261.65 | 7762.95 |
| osa-MIR5078 | 173.85 | 227.68 | 149.66 | 166.07 | 171.39 | 207.84 | 230.90 | 287.10 | 149.20 | 217.79 |
| osa-MIR5079 | 159.23 | 145.36 | 87.91 | 82.08 | 71.95 | 106.48 | 65.97 | 54.03 | 40.12 | 38.83 |
| osa-MIR5080 | 4.06 | 3.50 | 1.05 | 4.77 | 4.85 | 1.70 | 2.27 | 5.30 | 1.25 | 5.06 |
| osa-MIR5081 | 173.04 | 108.58 | 93.14 | 120.26 | 122.08 | 143.96 | 170.62 | 155.73 | 71.47 | 96.23 |
| osa-MIR5082 | 13.81 | 11.38 | 7.33 | 8.59 | 13.74 | 13.63 | 5.69 | 9.53 | 7.52 | 16.88 |
| osa-MIR5083 | 99.92 | 100.70 | 82.68 | 83.03 | 115.61 | 68.15 | 86.45 | 58.27 | 77.73 | 89.48 |
| osa-MIR5143a | 22.75 | 24.52 | 18.84 | 25.77 | 18.59 | 30.67 | 40.95 | 23.31 | 26.33 | 33.77 |
| osa-MIR5143b | 14.62 | 22.77 | 15.70 | 21.00 | 14.55 | 27.26 | 35.26 | 19.07 | 18.81 | 30.39 |
| osa-MIR5144 | 2496.50 | 2227.73 | 1532.17 | 2089.19 | 1980.74 | 2569.08 | 3711.50 | 3729.15 | 1374.15 | 2390.68 |
| osa-MIR5145 | 353.39 | 183.89 | 201.99 | 243.37 | 289.43 | 234.25 | 367.40 | 221.42 | 136.66 | 177.27 |

| | | | | | | | | | | |
|---|---|---|---|---|---|---|---|---|---|---|
| osa-MIR5146 | 7.31 | 0.00 | 1.05 | 4.77 | 4.04 | 0.85 | 2.27 | 1.06 | 1.25 | 0.00 |
| osa-MIR5147 | 24.37 | 22.77 | 13.61 | 20.04 | 17.79 | 29.81 | 23.89 | 24.37 | 7.52 | 20.26 |
| osa-MIR5148a | 523.18 | 284.60 | 274.20 | 308.27 | 309.64 | 309.21 | 453.84 | 308.29 | 176.78 | 192.47 |
| osa-MIR5148b | 508.56 | 276.72 | 256.41 | 295.87 | 288.62 | 299.84 | 462.94 | 302.99 | 169.26 | 184.03 |
| osa-MIR5148c | 561.37 | 319.62 | 306.64 | 354.08 | 343.60 | 341.58 | 509.58 | 349.61 | 196.84 | 226.24 |
| osa-MIR5149 | 39.00 | 29.77 | 24.07 | 26.72 | 33.96 | 34.92 | 51.19 | 38.14 | 13.79 | 37.14 |
| osa-MIR5150 | 5144.10 | 4042.14 | 2889.56 | 4483.80 | 3678.52 | 4997.62 | 5649.71 | 6360.75 | 2090.07 | 4205.64 |
| osa-MIR5151 | 229.10 | 155.87 | 170.59 | 165.11 | 162.50 | 240.21 | 307.11 | 390.93 | 146.69 | 212.73 |
| osa-MIR5152 | 3.25 | 0.88 | 1.05 | 1.91 | 2.43 | 1.70 | 1.14 | 4.24 | 2.51 | 1.69 |
| osa-MIR5153 | 69.05 | 49.04 | 32.44 | 62.99 | 53.36 | 49.41 | 65.97 | 66.74 | 28.84 | 28.70 |
| osa-MIR5154 | 110.49 | 58.67 | 57.56 | 57.26 | 84.89 | 72.40 | 106.92 | 56.15 | 32.60 | 55.71 |
| osa-MIR5155 | 4.06 | 3.50 | 2.09 | 2.86 | 3.23 | 3.41 | 2.27 | 6.36 | 0.00 | 8.44 |
| osa-MIR5156 | 36.56 | 35.03 | 25.12 | 41.04 | 33.15 | 46.85 | 40.95 | 41.32 | 23.82 | 37.14 |
| osa-MIR5157a | 41.43 | 28.02 | 16.75 | 31.50 | 22.64 | 31.52 | 14.79 | 24.37 | 6.27 | 10.13 |
| osa-MIR5157b | 41.43 | 28.02 | 16.75 | 31.50 | 22.64 | 31.52 | 14.79 | 24.37 | 6.27 | 10.13 |
| osa-MIR5158 | 5.69 | 12.26 | 10.47 | 11.45 | 5.66 | 12.78 | 13.65 | 15.89 | 11.28 | 10.13 |
| osa-MIR5159 | 3.25 | 2.63 | 1.05 | 2.86 | 0.00 | 2.56 | 2.27 | 0.00 | 3.76 | 5.06 |
| osa-MIR5160 | 749.03 | 404.56 | 368.39 | 323.54 | 522.27 | 442.95 | 742.75 | 462.97 | 323.48 | 334.29 |
| osa-MIR5161 | 67.43 | 82.31 | 50.24 | 62.04 | 71.15 | 64.74 | 87.58 | 104.88 | 31.34 | 62.47 |
| osa-MIR5162 | 6.50 | 9.63 | 17.79 | 13.36 | 8.89 | 17.04 | 13.65 | 18.01 | 15.05 | 15.19 |
| osa-MIR5179 | 0.00 | 0.00 | 0.00 | 0.00 | 0.81 | 0.00 | 0.00 | 0.00 | 0.00 | 0.00 |
| osa-MIR5337a | 29.25 | 9.63 | 6.28 | 11.45 | 15.36 | 14.48 | 17.06 | 22.25 | 13.79 | 16.88 |
| osa-MIR5337b | 33.31 | 28.90 | 12.56 | 19.09 | 24.25 | 26.41 | 31.85 | 33.90 | 12.54 | 32.08 |
| osa-MIR5338 | 0.81 | 0.00 | 0.00 | 1.91 | 0.00 | 0.00 | 1.14 | 0.00 | 1.25 | 0.00 |
| osa-MIR5339 | 23.56 | 9.63 | 9.42 | 24.81 | 15.36 | 17.04 | 22.75 | 20.13 | 11.28 | 21.95 |
| osa-MIR5340 | 12.19 | 14.01 | 10.47 | 11.45 | 9.70 | 17.89 | 11.37 | 26.49 | 5.02 | 18.57 |
| osa-MIR5484 | 0.81 | 0.88 | 0.00 | 0.00 | 1.62 | 1.70 | 0.00 | 1.06 | 1.25 | 0.00 |
| osa-MIR5485 | 0.00 | 0.00 | 0.00 | 0.95 | 0.00 | 0.00 | 1.14 | 1.06 | 1.25 | 0.00 |
| osa-MIR5486 | 0.00 | 0.00 | 0.00 | 0.00 | 0.00 | 0.00 | 0.00 | 0.00 | 0.00 | 0.00 |
| osa-MIR5487 | 0.81 | 0.00 | 1.05 | 0.00 | 1.62 | 0.00 | 1.14 | 0.00 | 5.02 | 0.00 |
| osa-MIR5488 | 0.00 | 0.00 | 0.00 | 0.00 | 0.00 | 0.00 | 0.00 | 1.06 | 0.00 | 0.00 |
| osa-MIR5489 | 0.00 | 0.00 | 0.00 | 0.00 | 0.00 | 0.00 | 0.00 | 0.00 | 0.00 | 0.00 |
| osa-MIR5490 | 8.12 | 3.50 | 3.14 | 14.32 | 9.70 | 5.11 | 11.37 | 1.06 | 7.52 | 5.06 |
| osa-MIR5491 | 0.00 | 0.00 | 0.00 | 0.00 | 0.00 | 0.00 | 0.00 | 0.00 | 0.00 | 0.00 |
| osa-MIR5492 | 0.00 | 0.00 | 0.00 | 0.00 | 0.00 | 0.00 | 0.00 | 1.06 | 0.00 | 0.00 |
| osa-MIR5493 | 13.81 | 19.26 | 46.05 | 29.59 | 28.30 | 18.74 | 14.79 | 13.77 | 36.36 | 20.26 |
| osa-MIR5494 | 0.00 | 0.00 | 0.00 | 0.00 | 0.00 | 0.00 | 0.00 | 0.00 | 0.00 | 0.00 |

| | | | | | | | | | | |
|---|---|---|---|---|---|---|---|---|---|---|
| osa-MIR5495 | 0.81 | 0.88 | 0.00 | 0.00 | 0.00 | 0.00 | 0.00 | 0.00 | 0.00 | 0.00 |
| osa-MIR5496 | 0.00 | 0.00 | 0.00 | 0.00 | 0.00 | 0.00 | 0.00 | 0.00 | 0.00 | 0.00 |
| osa-MIR5497 | 0.00 | 0.00 | 0.00 | 0.00 | 0.00 | 0.00 | 0.00 | 0.00 | 0.00 | 0.00 |
| osa-MIR5498 | 131.61 | 80.56 | 79.54 | 95.44 | 104.29 | 103.92 | 161.52 | 87.93 | 57.67 | 84.42 |
| osa-MIR5499 | 0.00 | 2.63 | 0.00 | 0.95 | 0.00 | 1.70 | 0.00 | 0.00 | 0.00 | 0.00 |
| osa-MIR5500 | 2.44 | 0.88 | 1.05 | 0.95 | 1.62 | 0.85 | 0.00 | 4.24 | 0.00 | 1.69 |
| osa-MIR5501 | 0.00 | 0.88 | 1.05 | 0.00 | 0.00 | 0.00 | 0.00 | 0.00 | 0.00 | 0.00 |
| osa-MIR5502 | 21.12 | 14.01 | 16.75 | 17.18 | 22.64 | 23.00 | 29.57 | 19.07 | 6.27 | 11.82 |
| osa-MIR5503 | 17.06 | 13.14 | 14.65 | 11.45 | 15.36 | 10.22 | 12.51 | 15.89 | 15.05 | 10.13 |
| osa-MIR5504 | 5.69 | 9.63 | 7.33 | 13.36 | 10.51 | 5.96 | 7.96 | 5.30 | 12.54 | 11.82 |
| osa-MIR5505 | 0.81 | 2.63 | 2.09 | 0.00 | 0.81 | 2.56 | 0.00 | 3.18 | 1.25 | 1.69 |
| osa-MIR5506 | 0.00 | 0.00 | 0.00 | 0.00 | 0.00 | 0.00 | 0.00 | 0.00 | 0.00 | 0.00 |
| osa-MIR5507 | 0.00 | 0.88 | 0.00 | 0.00 | 0.00 | 0.85 | 0.00 | 0.00 | 0.00 | 1.69 |
| osa-MIR5508 | 47.93 | 42.91 | 31.40 | 26.72 | 54.17 | 50.26 | 86.45 | 64.62 | 31.34 | 27.01 |
| osa-MIR5509 | 0.00 | 0.00 | 0.00 | 0.00 | 0.00 | 0.85 | 0.00 | 0.00 | 0.00 | 0.00 |
| osa-MIR5510 | 8.94 | 1.75 | 5.23 | 4.77 | 9.70 | 11.07 | 9.10 | 4.24 | 8.78 | 3.38 |
| osa-MIR5511 | 0.00 | 0.00 | 0.00 | 0.00 | 0.00 | 0.00 | 0.00 | 0.00 | 0.00 | 0.00 |
| osa-MIR5512a | 183.60 | 192.65 | 150.71 | 202.33 | 155.23 | 257.25 | 254.79 | 387.75 | 122.87 | 239.74 |
| osa-MIR5512b | 183.60 | 192.65 | 150.71 | 202.33 | 155.23 | 257.25 | 254.79 | 387.75 | 122.87 | 239.74 |
| osa-MIR5513 | 67.43 | 58.67 | 60.70 | 61.08 | 75.19 | 59.63 | 84.17 | 65.68 | 45.14 | 48.96 |
| osa-MIR5514 | 1.62 | 2.63 | 2.09 | 1.91 | 3.23 | 1.70 | 3.41 | 0.00 | 2.51 | 0.00 |
| osa-MIR5515 | 0.00 | 0.88 | 0.00 | 0.00 | 0.00 | 0.00 | 0.00 | 0.00 | 1.25 | 0.00 |
| osa-MIR5516a | 0.00 | 0.00 | 0.00 | 0.00 | 0.00 | 0.00 | 0.00 | 0.00 | 0.00 | 0.00 |
| osa-MIR5516b | 0.00 | 0.00 | 0.00 | 0.00 | 0.00 | 0.00 | 0.00 | 0.00 | 0.00 | 0.00 |
| osa-MIR5517 | 0.00 | 0.00 | 0.00 | 0.00 | 0.00 | 0.00 | 0.00 | 0.00 | 0.00 | 0.00 |
| osa-MIR5518 | 0.00 | 0.00 | 0.00 | 0.00 | 0.00 | 0.00 | 0.00 | 0.00 | 0.00 | 0.00 |
| osa-MIR5519 | 0.00 | 0.00 | 0.00 | 0.00 | 0.00 | 0.00 | 0.00 | 0.00 | 0.00 | 0.00 |
| osa-MIR5521 | 0.00 | 0.00 | 0.00 | 0.00 | 0.81 | 0.00 | 0.00 | 0.00 | 0.00 | 0.00 |
| osa-MIR5522 | 4.06 | 8.76 | 4.19 | 10.50 | 4.04 | 1.70 | 3.41 | 12.71 | 2.51 | 10.13 |
| osa-MIR5523 | 13.81 | 16.64 | 6.28 | 6.68 | 13.74 | 15.33 | 14.79 | 10.59 | 27.58 | 11.82 |
| osa-MIR5524 | 0.81 | 1.75 | 0.00 | 1.91 | 0.00 | 0.00 | 1.14 | 0.00 | 2.51 | 0.00 |
| osa-MIR5525 | 0.81 | 0.88 | 1.05 | 0.95 | 0.81 | 0.85 | 0.00 | 0.00 | 1.25 | 0.00 |
| osa-MIR5526 | 125.11 | 98.08 | 90.00 | 100.21 | 113.99 | 111.59 | 129.67 | 103.82 | 61.44 | 70.91 |
| osa-MIR5527 | 0.00 | 0.00 | 0.00 | 0.00 | 0.00 | 0.00 | 0.00 | 1.06 | 0.00 | 0.00 |
| osa-MIR5528 | 0.00 | 0.00 | 0.00 | 0.00 | 0.81 | 0.00 | 0.00 | 0.00 | 0.00 | 0.00 |
| osa-MIR5529 | 3.25 | 2.63 | 0.00 | 0.00 | 7.28 | 1.70 | 0.00 | 2.12 | 3.76 | 0.00 |
| osa-MIR5530 | 0.00 | 0.00 | 0.00 | 0.00 | 0.00 | 0.00 | 0.00 | 1.06 | 0.00 | 0.00 |

| | | | | | | | | | | |
|---|---|---|---|---|---|---|---|---|---|---|
| osa-MIR5531 | 0.00 | 0.00 | 0.00 | 0.95 | 0.00 | 0.00 | 0.00 | 0.00 | 0.00 | 0.00 |
| osa-MIR5532 | 22.75 | 22.77 | 14.65 | 21.00 | 24.25 | 23.85 | 21.61 | 27.54 | 23.82 | 16.88 |
| osa-MIR5533 | 0.00 | 0.00 | 0.00 | 0.00 | 0.00 | 0.00 | 0.00 | 0.00 | 0.00 | 0.00 |
| osa-MIR5534a | 13.81 | 14.01 | 12.56 | 19.09 | 19.40 | 17.89 | 29.57 | 14.83 | 2.51 | 20.26 |
| osa-MIR5534b | 2.44 | 3.50 | 3.14 | 6.68 | 2.43 | 8.52 | 2.27 | 3.18 | 0.00 | 3.38 |
| osa-MIR5535 | 1.62 | 3.50 | 0.00 | 0.95 | 0.81 | 1.70 | 1.14 | 1.06 | 2.51 | 0.00 |
| osa-MIR5536 | 49.56 | 43.78 | 51.28 | 47.72 | 65.49 | 60.48 | 72.80 | 54.03 | 36.36 | 38.83 |
| osa-MIR5537 | 6.50 | 8.76 | 4.19 | 0.95 | 3.23 | 5.11 | 3.41 | 3.18 | 5.02 | 3.38 |
| osa-MIR5538 | 23.56 | 14.89 | 8.37 | 6.68 | 16.17 | 24.70 | 20.47 | 26.49 | 8.78 | 8.44 |
| osa-MIR5539 | 9.75 | 7.88 | 3.14 | 5.73 | 7.28 | 11.07 | 6.82 | 4.24 | 6.27 | 13.51 |
| osa-MIR5540 | 411.07 | 287.22 | 198.85 | 234.78 | 279.73 | 293.03 | 350.33 | 329.48 | 184.31 | 231.30 |
| osa-MIR5541 | 0.81 | 0.00 | 0.00 | 0.00 | 0.00 | 0.00 | 0.00 | 0.00 | 2.51 | 0.00 |
| osa-MIR5542 | 41.43 | 35.03 | 25.12 | 28.63 | 38.81 | 34.07 | 54.60 | 46.61 | 32.60 | 40.52 |
| osa-MIR5543 | 17.87 | 10.51 | 8.37 | 7.64 | 7.28 | 8.52 | 6.82 | 3.18 | 0.00 | 6.75 |
| osa-MIR5544 | 0.00 | 0.00 | 1.05 | 0.00 | 0.81 | 0.00 | 0.00 | 0.00 | 0.00 | 0.00 |
| osa-MIR5788 | 3065.99 | 1969.41 | 1540.54 | 1647.30 | 2061.59 | 2215.58 | 2972.16 | 2241.73 | 1335.29 | 1522.88 |
| osa-MIR5789 | 0.00 | 0.00 | 0.00 | 0.00 | 0.00 | 0.00 | 0.00 | 0.00 | 0.00 | 0.00 |
| osa-MIR5790 | 47.12 | 37.65 | 34.54 | 38.18 | 59.83 | 44.29 | 80.76 | 50.85 | 27.58 | 37.14 |
| osa-MIR5791 | 0.00 | 0.00 | 0.00 | 0.00 | 0.00 | 0.00 | 0.00 | 0.00 | 0.00 | 0.00 |
| osa-MIR5792 | 2.44 | 1.75 | 3.14 | 0.95 | 1.62 | 1.70 | 3.41 | 0.00 | 0.00 | 0.00 |
| osa-MIR5793 | 0.00 | 0.00 | 0.00 | 0.00 | 0.00 | 0.00 | 0.00 | 0.00 | 0.00 | 0.00 |
| osa-MIR5794 | 26203.05 | 28398.32 | 19407.49 | 20316.42 | 22970.16 | 29897.97 | 43564.33 | 45768.97 | 15392.77 | 27621.12 |
| osa-MIR5795 | 2.44 | 5.25 | 2.09 | 3.82 | 6.47 | 8.52 | 9.10 | 9.53 | 2.51 | 5.06 |
| osa-MIR5796 | 0.00 | 0.00 | 0.00 | 0.00 | 0.00 | 0.00 | 0.00 | 0.00 | 0.00 | 0.00 |
| osa-MIR5797 | 0.00 | 0.00 | 0.00 | 0.00 | 0.00 | 0.00 | 0.00 | 0.00 | 0.00 | 0.00 |
| osa-MIR5798 | 0.00 | 0.00 | 0.00 | 0.00 | 0.00 | 0.00 | 0.00 | 0.00 | 0.00 | 0.00 |
| osa-MIR5799 | 226.66 | 157.62 | 113.03 | 147.93 | 175.44 | 178.03 | 197.92 | 183.28 | 101.56 | 121.56 |
| osa-MIR5800 | 0.00 | 0.00 | 0.00 | 0.00 | 0.00 | 0.00 | 0.00 | 0.00 | 0.00 | 1.69 |
| osa-MIR5801 | 780.71 | 520.15 | 410.25 | 538.28 | 815.74 | 735.12 | 896.31 | 707.69 | 511.55 | 557.15 |
| osa-MIR5802 | 372.08 | 323.13 | 212.45 | 318.77 | 314.49 | 329.65 | 433.37 | 366.56 | 206.88 | 310.65 |
| osa-MIR5803 | 9.75 | 3.50 | 3.14 | 5.73 | 8.89 | 5.11 | 9.10 | 3.18 | 2.51 | 3.38 |
| osa-MIR5804 | 0.00 | 0.00 | 0.00 | 0.00 | 0.00 | 0.00 | 0.00 | 0.00 | 0.00 | 0.00 |
| osa-MIR5805 | 0.00 | 11.38 | 4.19 | 8.59 | 0.81 | 11.93 | 2.27 | 15.89 | 2.51 | 3.38 |
| osa-MIR5806 | 0.00 | 0.00 | 0.00 | 0.00 | 0.00 | 0.00 | 0.00 | 0.00 | 0.00 | 0.00 |
| osa-MIR5807 | 0.00 | 0.00 | 0.00 | 2.86 | 0.81 | 0.00 | 0.00 | 2.12 | 3.76 | 0.00 |
| osa-MIR5808 | 281.09 | 207.54 | 169.54 | 218.56 | 287.81 | 248.73 | 308.25 | 296.64 | 188.07 | 278.57 |
| osa-MIR5809 | 59.30 | 27.15 | 23.02 | 26.72 | 122.08 | 63.89 | 114.88 | 23.31 | 67.70 | 30.39 |

| | | | | | | | | | | |
|---|---|---|---|---|---|---|---|---|---|---|
| osa-MIR5810 | 671.04 | 590.21 | 406.07 | 401.80 | 557.84 | 611.61 | 783.70 | 758.54 | 378.64 | 496.37 |
| osa-MIR5811 | 8.12 | 7.01 | 7.33 | 13.36 | 6.47 | 14.48 | 14.79 | 15.89 | 8.78 | 10.13 |
| osa-MIR5812 | 4.06 | 2.63 | 2.09 | 6.68 | 2.43 | 3.41 | 9.10 | 4.24 | 0.00 | 0.00 |
| osa-MIR5813 | 81.24 | 77.94 | 51.28 | 64.90 | 69.53 | 74.96 | 121.71 | 118.65 | 48.90 | 74.29 |
| osa-MIR5814 | 26.81 | 28.90 | 32.44 | 41.99 | 22.64 | 54.52 | 48.91 | 72.04 | 15.05 | 54.03 |
| osa-MIR5815 | 26.00 | 11.38 | 13.61 | 14.32 | 21.83 | 28.11 | 36.40 | 25.43 | 7.52 | 18.57 |
| osa-MIR5816 | 591.42 | 422.95 | 308.74 | 383.67 | 383.21 | 510.24 | 559.63 | 535.01 | 151.71 | 315.72 |
| osa-MIR5817 | 86.93 | 127.85 | 133.96 | 122.16 | 143.91 | 133.74 | 171.75 | 202.35 | 92.78 | 126.62 |
| osa-MIR5818 | 0.00 | 0.00 | 0.00 | 0.00 | 0.00 | 0.00 | 0.00 | 0.00 | 0.00 | 0.00 |
| osa-MIR5819 | 5.69 | 3.50 | 1.05 | 2.86 | 2.43 | 2.56 | 2.27 | 3.18 | 0.00 | 1.69 |
| osa-MIR5820 | 0.00 | 0.00 | 0.00 | 0.00 | 0.00 | 0.00 | 0.00 | 0.00 | 0.00 | 0.00 |
| osa-MIR5821 | 13.81 | 14.89 | 11.51 | 4.77 | 10.51 | 15.33 | 39.81 | 15.89 | 5.02 | 5.06 |
| osa-MIR5822 | 0.00 | 0.00 | 0.00 | 0.00 | 0.00 | 0.00 | 0.00 | 0.00 | 0.00 | 0.00 |
| osa-MIR5823 | 0.00 | 0.00 | 1.05 | 0.95 | 2.43 | 1.70 | 1.14 | 1.06 | 2.51 | 3.38 |
| osa-MIR5824 | 181.98 | 117.34 | 128.73 | 115.48 | 166.54 | 163.55 | 227.49 | 189.64 | 75.23 | 104.68 |
| osa-MIR5825 | 88.55 | 49.04 | 36.63 | 58.22 | 64.68 | 74.11 | 117.16 | 96.41 | 36.36 | 52.34 |
| osa-MIR5826 | 21.12 | 4.38 | 5.23 | 5.73 | 2.43 | 5.96 | 17.06 | 5.30 | 8.78 | 11.82 |
| osa-MIR5827 | 6.50 | 5.25 | 3.14 | 8.59 | 4.04 | 3.41 | 6.82 | 9.53 | 2.51 | 6.75 |
| osa-MIR5828 | 29.25 | 26.27 | 21.98 | 27.68 | 22.64 | 40.04 | 36.40 | 56.15 | 22.57 | 35.45 |
| osa-MIR5829 | 8.94 | 7.88 | 8.37 | 8.59 | 1.62 | 5.11 | 10.24 | 10.59 | 0.00 | 1.69 |
| osa-MIR5830 | 2.44 | 7.01 | 8.37 | 0.95 | 1.62 | 5.96 | 10.24 | 13.77 | 3.76 | 8.44 |
| osa-MIR5831 | 20.31 | 14.01 | 21.98 | 22.91 | 16.98 | 24.70 | 25.02 | 33.90 | 11.28 | 11.82 |
| osa-MIR5832 | 0.00 | 0.00 | 0.00 | 0.00 | 0.00 | 0.00 | 0.00 | 0.00 | 0.00 | 0.00 |
| osa-MIR5833 | 0.00 | 0.88 | 0.00 | 0.00 | 0.00 | 0.00 | 0.00 | 0.00 | 1.25 | 0.00 |
| osa-MIR5834 | 0.00 | 0.00 | 0.00 | 0.00 | 0.00 | 0.00 | 0.00 | 1.06 | 0.00 | 0.00 |
| osa-MIR5835 | 0.81 | 0.00 | 2.09 | 0.95 | 1.62 | 1.70 | 0.00 | 4.24 | 1.25 | 1.69 |
| osa-MIR5836 | 0.00 | 0.00 | 0.00 | 0.00 | 0.00 | 0.00 | 0.00 | 0.00 | 0.00 | 0.00 |
| osa-MIR5837 | 0.00 | 0.00 | 0.00 | 0.00 | 0.00 | 0.00 | 0.00 | 0.00 | 0.00 | 0.00 |
| osa-MIR6245 | 177.10 | 113.84 | 63.84 | 72.53 | 112.38 | 160.14 | 147.87 | 131.37 | 63.94 | 123.25 |
| osa-MIR6246 | 6.50 | 3.50 | 3.14 | 3.82 | 0.81 | 2.56 | 5.69 | 4.24 | 5.02 | 10.13 |
| osa-MIR6247 | 0.00 | 0.00 | 0.00 | 1.91 | 0.00 | 0.00 | 0.00 | 0.00 | 0.00 | 0.00 |
| osa-MIR6248 | 7.31 | 14.01 | 6.28 | 6.68 | 14.55 | 7.67 | 12.51 | 18.01 | 7.52 | 13.51 |
| osa-MIR6249 | 1847.39 | 1206.69 | 973.30 | 914.32 | 1166.62 | 1259.84 | 3093.86 | 1243.76 | 886.43 | 710.79 |
| osa-MIR6250 | 5.69 | 7.88 | 5.23 | 3.82 | 6.47 | 5.96 | 5.69 | 2.12 | 3.76 | 5.06 |
| osa-MIR6251 | 61.74 | 79.69 | 50.24 | 49.63 | 74.38 | 121.81 | 78.48 | 113.36 | 67.70 | 60.78 |
| osa-MIR6252 | 4.87 | 2.63 | 4.19 | 0.95 | 1.62 | 5.11 | 6.82 | 4.24 | 0.00 | 0.00 |
| osa-MIR6253 | 8.94 | 4.38 | 4.19 | 7.64 | 8.08 | 4.26 | 0.00 | 10.59 | 13.79 | 27.01 |

| | | | | | | | | | | |
|---|---|---|---|---|---|---|---|---|---|---|
| osa-MIR6254 | 0.00 | 31.52 | 0.00 | 27.68 | 1.62 | 33.22 | 0.00 | 47.67 | 0.00 | 38.83 |
| osa-MIR6255 | 4.87 | 2.63 | 2.09 | 7.64 | 3.23 | 2.56 | 2.27 | 3.18 | 5.02 | 8.44 |
| osa-MIR6256 | 0.81 | 6.13 | 11.51 | 13.36 | 3.23 | 0.00 | 9.10 | 8.48 | 16.30 | 20.26 |
| novel-1 | 96.68 | 119.09 | 49.19 | 100.21 | 118.04 | 150.77 | 136.49 | 177.98 | 47.64 | 82.73 |
| novel-2 | 45.49 | 52.54 | 34.54 | 56.31 | 49.32 | 69.85 | 72.80 | 52.97 | 25.08 | 43.90 |
| novel-3 | 23.56 | 20.14 | 17.79 | 18.13 | 17.79 | 22.15 | 25.02 | 13.77 | 13.79 | 23.64 |
| novel-4 | 69.05 | 77.94 | 57.56 | 84.94 | 59.83 | 100.51 | 110.33 | 153.62 | 47.64 | 101.30 |
| novel-5 | 18.69 | 14.89 | 13.61 | 15.27 | 11.32 | 19.59 | 15.92 | 33.90 | 3.76 | 18.57 |
| novel-6 | 121.86 | 45.54 | 69.07 | 57.26 | 70.34 | 66.44 | 169.48 | 110.18 | 73.97 | 119.87 |
| novel-7 | 3.25 | 4.38 | 6.28 | 6.68 | 8.08 | 4.26 | 4.55 | 8.48 | 6.27 | 16.88 |
| novel-8 | 128.36 | 110.34 | 77.45 | 95.44 | 96.21 | 115.00 | 94.41 | 192.81 | 50.15 | 65.84 |
| novel-9 | 21.12 | 22.77 | 29.30 | 36.27 | 19.40 | 46.00 | 42.09 | 60.39 | 13.79 | 43.90 |
| novel-10 | 119.42 | 71.81 | 57.56 | 66.81 | 108.33 | 84.33 | 113.74 | 82.63 | 95.29 | 77.66 |
| novel-11 | 47.12 | 32.40 | 46.05 | 22.91 | 34.76 | 41.74 | 61.42 | 48.73 | 27.58 | 37.14 |
| novel-12 | 194.98 | 185.64 | 126.63 | 147.93 | 157.65 | 220.62 | 219.53 | 262.74 | 130.39 | 221.17 |
| novel-13 | 94.24 | 88.44 | 63.84 | 57.26 | 66.29 | 65.59 | 73.93 | 76.28 | 155.47 | 82.73 |

| Table S6. A list of novel miRNAs in rice genome. | | | | | | |
|---|---|---|---|---|---|---|
| **ID** | **miRNA sequence** | **chrom** | **start** | **end** | **strand** | **reads** |
| novel-1 | TGGACAAGATTTTTTACATGGTTG | Chr02 | 15998751 | 15998774 | - | 1051 |
| novel-2 | AGGGTATTATGGTCTTTTCTCCTC | Chr03 | 17403832 | 17403855 | + | 433 |
| novel-3 | AATATATTTTGTACAAGAGATGGG | Chr03 | 17403510 | 17403533 | + | 193 |
| novel-4 | TAGGATATGGTAATGCTAAAA | Chr04 | 15821101 | 15821121 | - | 533 |
| novel-5 | AAGATTGCTACTTCATGCATA | Chr09 | 16002329 | 16002349 | + | 150 |
| novel-6 | CAACGTGGCGGTAATTTTGTA | Chr10 | 3561303 | 3561323 | + | 626 |
| novel-7 | TTAATAATGTGCGCATGAAAT | Chr10 | 14071388 | 14071408 | - | 56 |
| novel-8 | AGACTGCTTAGTTAAACGGTG | Chr11 | 338845 | 338865 | + | 1015 |
| novel-9 | AATCAAGTTAGGAACCATGCAAGT | Chr12 | 4647320 | 4647340 | + | 284 |
| novel-10 | CAAAATTTTCCATGCACTTCGA | Chr06 | 28179212 | 28179235 | - | 754 |
| novel-11 | TGGAGCATGAGGTTATCTCTC | Chr08 | 23702075 | 23702096 | - | 380 |
| novel-12 | ATTTAGTTGAATTAGAGTGGGTCA | Chr11 | 2584831 | 2584851 | + | 1645 |
| novel-13 | GTGTCGAGGAAGATCGAGTCGGAG | Chr12 | 10962541 | 10962564 | - | 630 |

13 novel miRNAs were identified. ID is the genetic code of novel miRNAs, the second column shows the sequence of each novel miRNA. Chrom represents the chromosome in which the novel miRNAs locate. Start and end represent the start sites and end sites in the chromosomes in rice. "+" represents novel miRNAs in positive-sense strand of DNA "-" represent novel miRNAs in antisense strand of DNA. The last column are amount of miRNAs reads.

**Table S7. A list of anti-correlated pairs between rice DE miRNAs and DE fungus genes**

(Anti-correlated pairs are the opposite expression pairs of DE rice miRNAs and their putative fungus genes. miRNA FC means the fold change of the miRNAs; gene FC means the fold change of the DE fungus genes; positive number shows miRNAs or genes are up-regulated expression, ; negative number shows miRNAs or genes are down-regulated expression. The column "target score" shows the matching degree of miRNAs and their putative target fungus genes, lower numbers mean better matching).

| comparison | miRNA ID | miRNA FC | exp1 | exp2 | gene ID (fungus) | gene FC | exp1 | exp2 | gene description | target score | range | alignment info | | |
|---|---|---|---|---|---|---|---|---|---|---|---|---|---|---|
| RI08_RC00 | osa-miR1431 | -1.18148 | 15.270484 | 35.902903 | MGG_08834 | 3.30326 | 8.8714183 | 0 | conserved hypothetical protein (1222 nt) | 4 | 564-584 | target 5' ACAAGCCCGCCAGC UCGCAAA 3' | ::::: <br> ::::.::::::::: | query 3' CGUUCGCCCGGUUGAG CGUUU 5' |
| RI08_RC08 | osa-miR1431 | -1.72569 | 15.270484 | 52.81274 | MGG_08834 | 3.30326 | 8.8714183 | 0 | conserved hypothetical protein (1222 nt) | 4 | 564-584 | target 5' ACAAGCCCGCCAGC UCGCAAA 3' | ::::: <br> ::::.::::::::: | query 3' CGUUCGCCCGGUUGAG CGUUU 5' |
| RI08_RC08 | osa-miR1862f | -1.3978 | 298.72884 | 788.78383 | MGG_10118 | 1.30951 | 1.4785697 | 0 | ATPase family AAA domain-containing protein 1 | 2.5 | 213-233 | target 5' CCAGAAUCAAGCC AACCUCAU 3' | :::.:: <br> : ::.:::::::::: | query 3' GGUUUUA-UUUGGUUG GAGUA 5' |
| RI08_RC08 | osa-miR1862f | -1.3978 | 298.72884 | 788.78383 | MGG_07703 | 1.30951 | 1.4785697 | 0 | predicted protein (1424 nt) | 3.5 | 123-142 | target 5' CUGAAGGAAACCA AUCUCAU 3' | :..::. <br> ::::::::.::::: | query 3' GGUUUUAUUUGGUUGG AGUA 5' |
| RI08_RC08 | osa-miR1862g | -1.3978 | 298.72884 | 788.78383 | MGG_10118 | 1.30951 | 1.4785697 | 0 | ATPase family AAA domain-containing protein 1 | 2.5 | 213-233 | target 5' CCAGAAUCAAGCC AACCUCAU 3' | :::.:: <br> : ::.:::::::::: | query 3' GGUUUUA-UUUGGUUG GAGUA 5' |
| RI08_RC08 | osa-miR1862g | -1.3978 | 298.72884 | 788.78383 | MGG_07703 | 1.30951 | 1.4785697 | 0 | predicted protein (1424 nt) | 3.5 | 123-142 | target 5' CUGAAGGAAACCA AUCUCAU 3' | :..::. <br> ::::::::.::::: | query 3' GGUUUUAUUUGGUUGG AGUA 5' |
| RI08_SI08 | osa-miR5160 | -3.48819 | 0.9544052 | 20.931286 | MGG_01973 | 1.6861 | 2.2178546 | 0 | conserved hypothetical protein (3625 nt) | 4 | 1977-1999 | target 5' UAGAGACCCUACC AUCGAUCUCG 3' | .:::.: <br> ::::::::::::::: | query 3' GUCUUU-AUAUGGUAG CUAGAGC 5' |
| RI24_RC00 | osa-miR1425-5p | -1.78538 | 3769.409 | 12995.975 | MGG_06769 | 3.11815 | 7.6827454 | 0 | conserved hypothetical protein (1874 nt) | 3 | 209-229 | target 5' AGCAACAAGGAUU GCAUCCUA 3' | :::: : <br> :::::::: :::::: | query 3' UCGUCGUUCCUAACUU AGGAU 5' |
| RI24_RI08 | osa-miR1850.1 | 1.21758 | 158.91269 | 67.762771 | MGG_12791 | -1.3961 | 6.4022878 | 18.482121 | hypothetical protein (672 nt) | 4 | 38-57 | target 5' CCUCGA-CUCCCAA CUCUCCA 3' | ::.:.: <br> ::::::::: :::: | query 3' GGGGUUAGAGGGUUGA AAGGU 5' |
| RI24_RI08 | osa-miR1862f | 1.88773 | 1108.1512 | 298.72884 | MGG_07703 | -1.3095 | 0 | 1.4785697 | predicted protein (1424 nt) | 3.5 | 123-142 | target 5' CUGAAGGAAACCA AUCUCAU 3' | :..::. <br> ::::::::.::::: | query 3' GGUUUUAUUUGGUUGG AGUA 5' |
| RI24_RI08 | osa-miR1862g | 1.88773 | 1108.1512 | 298.72884 | MGG_07703 | -1.3095 | 0 | 1.4785697 | predicted protein (1424 nt) | 3.5 | 123-142 | target 5' CUGAAGGAAACCA AUCUCAU 3' | :..::. <br> ::::::::.::::: | query 3' GGUUUUAUUUGGUUGG AGUA 5' |
| RI24_RI08 | osa-miR397a | 1.2274 | 57.208568 | 23.860131 | MGG_03767 | -1.9845 | 0 | 2.9571394 | hypothetical protein (1683 nt) | 4 | 1086-1106 | target 5' UUUCAACGCUGCA CUCAAUCG 3' | . ::::: <br> :::::::::::: . | query 3' GUAGUUGCGACGUGAG UUACU 5' |
| RI24_RI08 | osa-miR397a | 1.2274 | 57.208568 | 23.860131 | MGG_04775 | -2.4311 | 9.6034318 | 56.185649 | fatty acid synthase S-acetyltransferase (6979 | 4 | 1356-1375 | target 5' CAUCAAGGCUGCA CUC-AUGC 3' | :::::: <br> ::::::::: ::: | query 3' GUAGUUGCGACGUGAG UUACU 5' |
| RI24_RI08 | osa-miR397b | 1.2274 | 57.208568 | 23.860131 | MGG_03767 | -1.9845 | 0 | 2.9571394 | hypothetical protein (1683 nt) | 4 | 1086-1106 | target 5' UUUCAACGCUGCA CUCAAUCG 3' | . ::::: <br> :::::::::::: . | query 3' GUAGUUGCGACGUGAG UUAUU 5' |
| RI24_RI08 | osa-miR444b.2 | 1.40794 | 11618.636 | 4377.8568 | MGG_06680 | -1.3095 | 0 | 1.4785697 | nudix/MutT family protein (1328 nt) | 3 | 978-997 | target 5' AAGAUUGAG-CAAC AACUGCA 3' | ::: :: <br> ::: ::::::::::: | query 3' UUCGAACUCUGUUGUU GACGU 5' |
| RI24_RI08 | osa-miR444c.2 | 1.40794 | 11618.636 | 4377.8568 | MGG_06680 | -1.3095 | 0 | 1.4785697 | nudix/MutT family protein (1328 nt) | 3 | 978-997 | target 5' AAGAUUGAG-CAAC AACUGCA 3' | ::: :: <br> ::: ::::::::::: | query 3' UUCGAACUCUGUUGUU GACGU 5' |

| | | | | | | | | | | | | | |
|---|---|---|---|---|---|---|---|---|---|---|---|---|---|
| RI24_RI08 | osa-miR444f | 1.24508 | 13282.982 | 5603.3131 | MGG_06680 | -1.3095 | 0 | 1.4785697 | nudix/MutT family protein (1328 nt) | 3 | 978-997 | target 5' AAGAUUGA-GCAACAACUGCA 3' | ::: ::<br>:: ::::::::::: | query 3' UUCGAACUCCGUUGUUGACGU 5' |
| RI24_SI24 | osa-miR1425-5p | -1.10212 | 3769.409 | 8092.955 | MGG_06769 | 1.98591 | 7.6827454 | 1.1919868 | conserved hypothetical protein (1874 nt) | 3 | 209-229 | target 5' AGCAACAAGGAUUGCAUCCUA 3' | :::: :<br>::::::: :::::: | query 3' UCGUCGUUCCUAACUUAGGAU 5' |
| RI24_SI24 | osa-miR159a.2 | -1.58837 | 46.614389 | 142.18122 | MGG_14003 | 1.18932 | 1.2804576 | 0 | ankyrin repeat protein (4561 nt) | 3 | 2089-2110 | target 5' CGCAGUCGCCUGGGGCAUGCAA 3' | ::::<br>: ::::::::::::: | query 3' ACGUC-GAGGACCCCGUACGUU 5' |
| RI24_SI24 | osa-miR169n | 1.06485 | 319.94422 | 152.41827 | MGG_01974 | -1.1322 | 0 | 1.1919868 | predicted protein (529 nt) | 3.5 | 416-436 | target 5' UGGGUAGGUCAAUCUUGGCUA 3' | :.::.:.<br>:::: ::::::::: | query 3' AUCCGUUCAGUAAGAACCGAU 5' |
| RI24_SI24 | osa-miR169n | 1.06485 | 319.94422 | 152.41827 | MGG_10639 | -2.1941 | 0 | 3.5759605 | predicted protein (2313 nt) | 4 | 1590-1610 | target 5' UAGACCAGUCAUUCUUGGCAA 3' | ::: : :<br>::::::::::: : | query 3' AUCCGUUCAGUAAGAACCGAU 5' |
| RI24_SI24 | osa-miR169n | 1.06485 | 319.94422 | 152.41827 | MGG_04899 | -1.1322 | 0 | 1.1919868 | lipid A export ATP-binding/permease protein m | 4 | 3032-3051 | target 5' UGGGCCA-UCGUUCUUGGCUG 3' | :.::: :<br>::.:::::::::. | query 3' AUCCGUUCAGUAAGAACCGAU 5' |
| RI24_SI24 | osa-miR169o | 1.06485 | 319.94422 | 152.41827 | MGG_01974 | -1.1322 | 0 | 1.1919868 | predicted protein (529 nt) | 3.5 | 416-436 | target 5' UGGGUAGGUCAAUCUUGGCUA 3' | :.::.:.<br>:::: ::::::::: | query 3' AUCCGUUCAGUAAGAACCGAU 5' |
| RI24_SI24 | osa-miR169o | 1.06485 | 319.94422 | 152.41827 | MGG_10639 | -2.1941 | 0 | 3.5759605 | predicted protein (2313 nt) | 4 | 1590-1610 | target 5' UAGACCAGUCAUUCUUGGCAA 3' | ::: : :<br>::::::::::: : | query 3' AUCCGUUCAGUAAGAACCGAU 5' |
| RI24_SI24 | osa-miR169o | 1.06485 | 319.94422 | 152.41827 | MGG_04899 | -1.1322 | 0 | 1.1919868 | lipid A export ATP-binding/permease protein m | 4 | 3032-3051 | target 5' UGGGCCA-UCGUUCUUGGCUG 3' | :.::: :<br>::.:::::::::. | query 3' AUCCGUUCAGUAAGAACCGAU 5' |
| RI24_SI24 | osa-miR1850.1 | 2.97513 | 158.91269 | 19.336646 | MGG_08821 | -1.7587 | 0 | 2.3839737 | conserved hypothetical protein (2400 nt) | 4 | 539-558 | target 5' CCACAAU-UCCCAACUUUGCA 3' | :: :::<br>: ::::::::: :: | query 3' GGGGUUAGAGGGUUGAAAGGU 5' |
| RI48_RC00 | osa-miR1433 | -2.032 | 37.14331 | 154.99546 | MGG_00843 | 3.00656 | 7.0364838 | 0 | regulatory protein suaprga1 (1281 nt) | 4 | 296-315 | target 5' GGUUGACGAGGAG-CUUGCCA 3' | ::: : :<br>:::::: ::::::: | query 3' CCAUCGGCUCCUCUGAACGGU 5' |
| RI48_RI24 | osa-miR1433 | -2.20447 | 37.14331 | 174.80396 | MGG_00843 | 2.29267 | 7.0364838 | 0.6402288 | regulatory protein suaprga1 (1281 nt) | 4 | 296-315 | target 5' GGUUGACGAGGAG-CUUGCCA 3' | ::: : :<br>:::::: ::::::: | query 3' CCAUCGGCUCCUCUGAACGGU 5' |
| RI48_RI24 | osa-miR5508 | -1.41954 | 21.94832 | 60.386822 | MGG_14752 | 1.17432 | 7.0364838 | 2.5609151 | conserved hypothetical protein (3554 nt) | 4 | 846-866 | target 5' CCACGGAGGAUCAGCCAUCUU 3' | ::::.<br>.:::::::::::: | query 3' GGUGUGGUCUAGUCGGUAGAU 5' |
| RI48_SI48 | osa-miR1431 | 1.51262 | 59.09163 | 20.060627 | MGG_08834 | -2.8902 | 88.346964 | 661.38677 | conserved hypothetical protein (1222 nt) | 4 | 564-584 | target 5' ACAAGCCCGCCAGCUCGCAAA 3' | :::::<br>::::.::::::::: | query 3' CGUUCGCCCGGUUGAGCGUUU 5' |
| RI48_SI48 | osa-miR169e | 1.01023 | 3380.0412 | 1677.57 | MGG_06752 | -1.2989 | 4.6909892 | 13.002361 | peptidase family M28 family (3293 nt) | 4 | 1787-1807 | target 5' CUGGCUCUUCAUCCUUGGCUG 3' | :.:::<br>:::::::::::::. | query 3' GGCCGUUCAGUAGGAACCGAU 5' |
| RI48_SI48 | osa-miR169n | 1.01304 | 205.97654 | 101.55693 | MGG_01974 | -3.4425 | 1.5636631 | 26.871546 | predicted protein (529 nt) | 3.5 | 416-436 | target 5' UGGGUAGGUCAAUCUUGGCUA 3' | :.::.:.<br>:::: ::::::::: | query 3' AUCCGUUCAGUAAGAACCGAU 5' |
| RI48_SI48 | osa-miR169n | 1.01304 | 205.97654 | 101.55693 | MGG_10639 | -1.9879 | 0.7818315 | 6.0677686 | predicted protein (2313 nt) | 4 | 1590-1610 | target 5' UAGACCAGUCAUUCUUGGCAA 3' | ::: : :<br>::::::::::: : | query 3' AUCCGUUCAGUAAGAACCGAU 5' |
| RI48_SI48 | osa-miR169n | 1.01304 | 205.97654 | 101.55693 | MGG_04899 | -3.6169 | 0 | 11.268713 | lipid A export ATP-binding/permease protein m | 4 | 3032-3051 | target 5' UGGGCCA-UCGUUCUUGGCUG 3' | :.::: :<br>::.:::::::::. | query 3' AUCCGUUCAGUAAGAACCGAU 5' |
| RI48_SI48 | osa-miR169o | 1.01304 | 205.97654 | 101.55693 | MGG_01974 | -3.4425 | 1.5636631 | 26.871546 | predicted protein (529 nt) | 3.5 | 416-436 | target 5' UGGGUAGGUCAAUCUUGGCUA 3' | :.::.:.<br>:::: ::::::::: | query 3' AUCCGUUCAGUAAGAACCGAU 5' |

| | | | | | | | | | | | | | | |
|---|---|---|---|---|---|---|---|---|---|---|---|---|---|---|
| RI48_SI48 | osa-miR169o | 1.01304 | 205.97654 | 101.55693 | MGG_10639 | -1.9879 | 0.7818315 | 6.0677686 | predicted protein (2313 nt) | 4 | 1590-1610 | target 5' UAGACCAGUCAUUCUUGGCAA 3' | ::: : :<br>:::::::::::: : | query 3' AUCCGUUCAGUAAGAACCGAU 5' |
| RI48_SI48 | osa-miR169o | 1.01304 | 205.97654 | 101.55693 | MGG_04899 | -3.6169 | 0 | 11.268713 | lipid A export ATP-binding/permease protein m | 4 | 3032-3051 | target 5' UGGGCCA-UCGUUCUUGGCUG 3' | :.::: :<br>::.:::::::::. | query 3' AUCCGUUCAGUAAGAACCGAU 5' |
| RI48_SI48 | osa-miR1850.1 | 1.0015 | 683.77457 | 341.03067 | MGG_08821 | -3.3971 | 0 | 9.5350649 | conserved hypothetical protein (2400 nt) | 4 | 539-558 | target 5' CCACAAU-UCCCAACUUUGCA 3' | :: :::<br>: ::::::::: :: | query 3' GGGGUUAGAGGGUUGAAAGGU 5' |
| RI48_SI48 | osa-miR1850.1 | 1.0015 | 683.77457 | 341.03067 | MGG_12791 | -1.8154 | 9.3819785 | 35.539787 | hypothetical protein (672 nt) | 4 | 38-57 | target 5' CCUCGA-CUCCCAACUCUCCA 3' | ::.:.:<br>::::::::: :::: | query 3' GGGGUUAGAGGGUUGAAAGGU 5' |
| RI48_SI48 | osa-miR2864.2 | 6.84841 | 2714.8383 | 22.568206 | MGG_02981 | -3.5112 | 0 | 10.401889 | anaphase-promoting complex protein (2728 nt) | 4 | 1123-1143 | target 5' CGACUACACAAUGCAAAACAG 3' | . :::<br>:::::::::::::. | query 3' AUGGAUAUGUUACGUUUUGUU 5' |
| RI48_SI48 | osa-miR5161 | 2.04817 | 13.506658 | 2.5075784 | MGG_02949 | -1.7889 | 7.8183154 | 29.472019 | conserved hypothetical protein (3524 nt) | 4 | 461-479 | target 5' UAUGGU-CCUUUGAUCCAGG 3' | :::. :<br>:::.::::::::. | query 3' AUAUGAGGGAGACUAGGUCU 5' |
| RI48_SI48 | osa-miR5161 | 2.04817 | 13.506658 | 2.5075784 | MGG_02732 | -2.9882 | 0 | 6.9345926 | conserved hypothetical protein (1700 nt) | 4 | 1168-1186 | target 5' AAGACU-CCUCUGAUCCAGC 3' | : :::<br>:::::::::::: | query 3' AUAUGAGGGAGACUAGGUCU 5' |
| SI08_SC00 | osa-miR1431 | -1.28912 | 15.698465 | 39.807448 | MGG_08834 | 3.41944 | 9.6992311 | 0 | conserved hypothetical protein (1222 nt) | 4 | 564-584 | target 5' ACAAGCCCGCCAGCUCGCAAA 3' | :::::<br>::::.:::::::: | query 3' CGUUCGCCCGGUUGAGCGUUU 5' |
| SI08_SC00 | osa-miR160a | -1.45851 | 332.80745 | 916.3837 | MGG_13163 | 2.08171 | 3.233077 | 0 | conserved hypothetical protein (1031 nt) | 2.5 | 413-434 | target 5' UGGCACUACAGGGAGCUAGGCG 3' | :::::<br>:::::::::::.::::. | query 3' ACCGU-AUGUCCCUCGGUCCGU 5' |
| SI08_SC00 | osa-miR160a | -1.45851 | 332.80745 | 916.3837 | MGG_13171 | 2.08171 | 3.233077 | 0 | E3 ubiquitin-protein ligase ubr1 (6712 nt) | 4 | 1075-1095 | target 5' UGGCAUCGAGGAAGCCAGGCA 3' | ::::::<br>::: ::::::::: | query 3' ACCGUAUGUCCCUCGGUCCGU 5' |
| SI08_SC00 | osa-miR160b | -1.45851 | 332.80745 | 916.3837 | MGG_13163 | 2.08171 | 3.233077 | 0 | conserved hypothetical protein (1031 nt) | 2.5 | 413-434 | target 5' UGGCACUACAGGGAGCUAGGCG 3' | :::::<br>:::::::::::.::::. | query 3' ACCGU-AUGUCCCUCGGUCCGU 5' |
| SI08_SC00 | osa-miR160b | -1.45851 | 332.80745 | 916.3837 | MGG_13171 | 2.08171 | 3.233077 | 0 | E3 ubiquitin-protein ligase ubr1 (6712 nt) | 4 | 1075-1095 | target 5' UGGCAUCGAGGAAGCCAGGCA 3' | ::::::<br>::: ::::::::: | query 3' ACCGUAUGUCCCUCGGUCCGU 5' |
| SI08_SC00 | osa-miR160c | -1.45851 | 332.80745 | 916.3837 | MGG_13163 | 2.08171 | 3.233077 | 0 | conserved hypothetical protein (1031 nt) | 2.5 | 413-434 | target 5' UGGCACUACAGGGAGCUAGGCG 3' | :::::<br>:::::::::::.::::. | query 3' ACCGU-AUGUCCCUCGGUCCGU 5' |
| SI08_SC00 | osa-miR160c | -1.45851 | 332.80745 | 916.3837 | MGG_13171 | 2.08171 | 3.233077 | 0 | E3 ubiquitin-protein ligase ubr1 (6712 nt) | 4 | 1075-1095 | target 5' UGGCAUCGAGGAAGCCAGGCA 3' | ::::::<br>::: ::::::::: | query 3' ACCGUAUGUCCCUCGGUCCGU 5' |
| SI08_SC00 | osa-miR160d | -1.45851 | 332.80745 | 916.3837 | MGG_13163 | 2.08171 | 3.233077 | 0 | conserved hypothetical protein (1031 nt) | 2.5 | 413-434 | target 5' UGGCACUACAGGGAGCUAGGCG 3' | :::::<br>:::::::::::.::::. | query 3' ACCGU-AUGUCCCUCGGUCCGU 5' |
| SI08_SC00 | osa-miR160d | -1.45851 | 332.80745 | 916.3837 | MGG_13171 | 2.08171 | 3.233077 | 0 | E3 ubiquitin-protein ligase ubr1 (6712 nt) | 4 | 1075-1095 | target 5' UGGCAUCGAGGAAGCCAGGCA 3' | ::::::<br>::: ::::::::: | query 3' ACCGUAUGUCCCUCGGUCCGU 5' |
| SI08_SC00 | osa-miR160e | -1.40872 | 276.29298 | 735.21919 | MGG_13163 | 2.08171 | 3.233077 | 0 | conserved hypothetical protein (1031 nt) | 3 | 413-434 | target 5' UGGCACUACAGGGAGCUAGGCG 3' | .:::: :<br>::::::::::.::::. | query 3' GCCGU-AUGUCCCUCGGUCCGU 5' |
| SI08_SC00 | osa-miR160f | -1.53015 | 252.222 | 730.34481 | MGG_13163 | 2.08171 | 3.233077 | 0 | conserved hypothetical protein (1031 nt) | 3.5 | 413-434 | target 5' UGGCACUACAGGGAGCUAGGCG 3' | :::::<br>: :::::::::.::::. | query 3' ACCGU-AAGUCCCUCGGUCCGU 5' |
| SI08_SC00 | osa-miR160f | -1.53015 | 252.222 | 730.34481 | MGG_13171 | 2.08171 | 3.233077 | 0 | E3 ubiquitin-protein ligase ubr1 (6712 nt) | 4 | 1075-1095 | target 5' UGGCAUCGAGGAAGCCAGGCA 3' | ::::::<br>::: ::::::::: | query 3' ACCGUAAGUCCCUCGGUCCGU 5' |

| | | | | | | | | | | | | | | |
|---|---|---|---|---|---|---|---|---|---|---|---|---|---|---|
| SI08_SC00 | osa-miR166i | -1.65604 | 13792.671 | 43469.733 | MGG_08115 | 2.08171 | 3.233077 | 0 | NAD-binding Rossmann fold oxidoreductase fami | 3.5 | 1143-1164 | target 5'<br>GAGGCUGGGAAGC<br>CUGAUCCGA 3' | ::::<br>. ::::::::::::: | query 3'<br>CUCC-UUACUUCGGACU<br>AGGCU 5' |
| SI08_SC00 | osa-miR166j | -1.65604 | 13792.671 | 43469.733 | MGG_08115 | 2.08171 | 3.233077 | 0 | NAD-binding Rossmann fold oxidoreductase fami | 3.5 | 1143-1164 | target 5'<br>GAGGCUGGGAAGC<br>CUGAUCCGA 3' | ::::<br>. ::::::::::::: | query 3'<br>CUCC-UUACUUCGGACU<br>AGGCU 5' |
| SI08_SC00 | osa-miR1862f | -1.31898 | 220.82507 | 552.42989 | MGG_10118 | 1.38766 | 1.6165385 | 0 | ATPase family AAA domain-containing protein 1 | 2.5 | 213-233 | target 5'<br>CCAGAAUCAAGCC<br>AACCUCAU 3' | :::.::<br>: ::.:::::::::: | query 3'<br>GGUUUUA-UUUGGUUG<br>GAGUA 5' |
| SI08_SC00 | osa-miR1862f | -1.31898 | 220.82507 | 552.42989 | MGG_06360 | 2.33381 | 4.0413463 | 0 | conserved hypothetical protein (4721 nt) | 4 | 1324-1343 | target 5'<br>CCAUACUCAACCA<br>ACCUCAU 3' | ::: : :<br>::::::::::: | query 3'<br>GGUUUUAUUUGGUUGG<br>AGUA 5' |
| SI08_SC00 | osa-miR1862g | -1.31898 | 220.82507 | 552.42989 | MGG_10118 | 1.38766 | 1.6165385 | 0 | ATPase family AAA domain-containing protein 1 | 2.5 | 213-233 | target 5'<br>CCAGAAUCAAGCC<br>AACCUCAU 3' | :::.::<br>: ::.:::::::::: | query 3'<br>GGUUUUA-UUUGGUUG<br>GAGUA 5' |
| SI08_SC00 | osa-miR1862g | -1.31898 | 220.82507 | 552.42989 | MGG_06360 | 2.33381 | 4.0413463 | 0 | conserved hypothetical protein (4721 nt) | 4 | 1324-1343 | target 5'<br>CCAUACUCAACCA<br>ACCUCAU 3' | ::: : :<br>::::::::::: | query 3'<br>GGUUUUAUUUGGUUGG<br>AGUA 5' |
| SI08_SC00 | osa-miR2055 | -1.36934 | 57.561038 | 150.29343 | MGG_06905 | 2.54834 | 4.8496155 | 0 | protein transport protein sec73 (4680 nt) | 3 | 1735-1756 | target 5'<br>GAAGCUGAGCUUC<br>CCAAGGAAA 3' | :::.:.<br>: ::::::::::::: | query 3'<br>CUUUGG-UGGAAGGGU<br>UCCUUU 5' |
| SI08_SC00 | osa-miR2055 | -1.36934 | 57.561038 | 150.29343 | MGG_04685 | 3.18312 | 8.0826926 | 0 | conserved hypothetical protein (4541 nt) | 4 | 1767-1788 | target 5'<br>GACGAUAUCCUUC<br>CCAAGGAAA 3' | :: .:.<br>::::::::::::: | query 3'<br>CU-UUGGUGGAAGGGU<br>UCCUUU 5' |
| SI08_SC00 | osa-miR396a | -1.25267 | 455.25548 | 1086.1747 | MGG_01046 | 1.77602 | 2.4248078 | 0 | methionyl-tRNA synthetase (2588 nt) | 3.5 | 2556-2576 | target 5'<br>CAGCUUGAGAAGG<br>CUGUGGAG 3' | ::: :..<br>::::.::::::::. | query 3'<br>GUCAAGUUCUUUCGAC<br>ACCUU 5' |
| SI08_SC00 | osa-miR396b | -1.25267 | 455.25548 | 1086.1747 | MGG_01046 | 1.77602 | 2.4248078 | 0 | methionyl-tRNA synthetase (2588 nt) | 3.5 | 2556-2576 | target 5'<br>CAGCUUGAGAAGG<br>CUGUGGAG 3' | ::: :..<br>::::.::::::::. | query 3'<br>GUCAAGUUCUUUCGAC<br>ACCUU 5' |
| SI08_SC00 | osa-miR396c | -1.27383 | 457.34861 | 1107.297 | MGG_08526 | 8.83562 | 455.86386 | 0 | hypothetical protein (2024 nt) | 4 | 1874-1894 | target 5'<br>GGGUUCAAAAAAG<br>UUGUGGAA 3' | ..::::<br>: ::::.:::::: | query 3'<br>UUCAAGUUCUUUCGAC<br>ACCUU 5' |
| SI08_SC00 | osa-miR396c | -1.27383 | 457.34861 | 1107.297 | MGG_06547 | 2.54834 | 4.8496155 | 0 | tartrate dehydrogenase/decarboxylase (1258 nt | 4 | 1108-1128 | target 5'<br>AAGCUCCUGGAAG<br>CUGUGGAA 3' | ::: ::<br>:.:::::::::: | query 3'<br>UUCAAGUUCUUUCGAC<br>ACCUU 5' |
| SI08_SC00 | osa-miR444b.2 | -1.00775 | 3830.4254 | 7703.1474 | MGG_06680 | 1.38766 | 1.6165385 | 0 | nudix/MutT family protein (1328 nt) | 3 | 978-997 | target 5'<br>AAGAUUGAG-CAAC<br>AACUGCA 3' | ::: ::<br>::: :::::::::: | query 3'<br>UUCGAACUCUGUUGUU<br>GACGU 5' |
| SI08_SC00 | osa-miR444c.2 | -1.00775 | 3830.4254 | 7703.1474 | MGG_06680 | 1.38766 | 1.6165385 | 0 | nudix/MutT family protein (1328 nt) | 3 | 978-997 | target 5'<br>AAGAUUGAG-CAAC<br>AACUGCA 3' | ::: ::<br>::: :::::::::: | query 3'<br>UUCGAACUCUGUUGUU<br>GACGU 5' |
| SI08_SC00 | osa-miR444d.3 | -1.10466 | 42.909137 | 93.425643 | MGG_14775 | 2.73506 | 5.6578848 | 0 | conserved hypothetical protein (3712 nt) | 4 | 3018-3038 | target 5'<br>CCACUAGCAAGAA<br>AGCCAUGA 3' | : ::: :<br>:::::::::::..: | query 3'<br>GUUGAACGUUCUUUCG<br>GUGUU 5' |
| SI08_SC08 | osa-miR159a.2 | -2.45633 | 34.536623 | 194.03204 | MGG_14003 | 2.08171 | 3.233077 | 0 | ankyrin repeat protein (4561 nt) | 3 | 2089-2110 | target 5'<br>CGCAGUCGCCUGG<br>GGCAUGCAA 3' | ::::<br>: ::::::::::::: | query 3'<br>ACGUC-GAGGACCCCGU<br>ACGUU 5' |
| SI08_SC08 | osa-miR160a | -1.19797 | 332.80745 | 764.80964 | MGG_13163 | 2.08171 | 3.233077 | 0 | conserved hypothetical protein (1031 nt) | 2.5 | 413-434 | target 5'<br>UGGCACUACAGGG<br>AGCUAGGCG 3' | :::::<br>::::::::::.::::. | query 3'<br>ACCGU-AUGUCCCUCGG<br>UCCGU 5' |
| SI08_SC08 | osa-miR160a | -1.19797 | 332.80745 | 764.80964 | MGG_13171 | 2.08171 | 3.233077 | 0 | E3 ubiquitin-protein ligase ubr1 (6712 nt) | 4 | 1075-1095 | target 5'<br>UGGCAUCGAGGAA<br>GCCAGGCA 3' | ::::::<br>::: ::::::::: | query 3'<br>ACCGUAUGUCCCUCGG<br>UCCGU 5' |
| SI08_SC08 | osa-miR160b | -1.19797 | 332.80745 | 764.80964 | MGG_13163 | 2.08171 | 3.233077 | 0 | conserved hypothetical protein (1031 nt) | 2.5 | 413-434 | target 5'<br>UGGCACUACAGGG<br>AGCUAGGCG 3' | :::::<br>::::::::::.::::. | query 3'<br>ACCGU-AUGUCCCUCGG<br>UCCGU 5' |

| | | | | | | | | | | | | | | |
|---|---|---|---|---|---|---|---|---|---|---|---|---|---|---|
| SI08_SC08 | osa-miR160b | -1.19797 | 332.80745 | 764.80964 | MGG_13171 | 2.08171 | 3.233077 | 0 | E3 ubiquitin-protein ligase ubr1 (6712 nt) | 4 | 1075-1095 | target 5' UGGCAUCGAGGAA GCCAGGCA 3' | :::::: ::: ::::::::: | query 3' ACCGUAUGUCCCUCGG UCCGU 5' |
| SI08_SC08 | osa-miR160c | -1.19797 | 332.80745 | 764.80964 | MGG_13163 | 2.08171 | 3.233077 | 0 | conserved hypothetical protein (1031 nt) | 2.5 | 413-434 | target 5' UGGCACUACAGGG AGCUAGGCG 3' | ::::: ::::::::::.::::. | query 3' ACCGU-AUGUCCCUCGG UCCGU 5' |
| SI08_SC08 | osa-miR160c | -1.19797 | 332.80745 | 764.80964 | MGG_13171 | 2.08171 | 3.233077 | 0 | E3 ubiquitin-protein ligase ubr1 (6712 nt) | 4 | 1075-1095 | target 5' UGGCAUCGAGGAA GCCAGGCA 3' | :::::: ::: ::::::::: | query 3' ACCGUAUGUCCCUCGG UCCGU 5' |
| SI08_SC08 | osa-miR160d | -1.19797 | 332.80745 | 764.80964 | MGG_13163 | 2.08171 | 3.233077 | 0 | conserved hypothetical protein (1031 nt) | 2.5 | 413-434 | target 5' UGGCACUACAGGG AGCUAGGCG 3' | ::::: ::::::::::.::::. | query 3' ACCGU-AUGUCCCUCGG UCCGU 5' |
| SI08_SC08 | osa-miR160d | -1.19797 | 332.80745 | 764.80964 | MGG_13171 | 2.08171 | 3.233077 | 0 | E3 ubiquitin-protein ligase ubr1 (6712 nt) | 4 | 1075-1095 | target 5' UGGCAUCGAGGAA GCCAGGCA 3' | :::::: ::: ::::::::: | query 3' ACCGUAUGUCCCUCGG UCCGU 5' |
| SI08_SC08 | osa-miR160e | -1.10006 | 276.29298 | 593.41467 | MGG_13163 | 2.08171 | 3.233077 | 0 | conserved hypothetical protein (1031 nt) | 3 | 413-434 | target 5' UGGCACUACAGGG AGCUAGGCG 3' | .:::: : :::::::::.::::. | query 3' GCCGU-AUGUCCCUCGG UCCGU 5' |
| SI08_SC08 | osa-miR160f | -1.26213 | 252.222 | 606.35014 | MGG_13163 | 2.08171 | 3.233077 | 0 | conserved hypothetical protein (1031 nt) | 3.5 | 413-434 | target 5' UGGCACUACAGGG AGCUAGGCG 3' | ::::: : :::::::::.::::. | query 3' ACCGU-AAGUCCCUCGG UCCGU 5' |
| SI08_SC08 | osa-miR160f | -1.26213 | 252.222 | 606.35014 | MGG_13171 | 2.08171 | 3.233077 | 0 | E3 ubiquitin-protein ligase ubr1 (6712 nt) | 4 | 1075-1095 | target 5' UGGCAUCGAGGAA GCCAGGCA 3' | :::::: ::: ::::::::: | query 3' ACCGUAAGUCCCUCGG UCCGU 5' |
| SI08_SC08 | osa-miR1862f | -1.78909 | 220.82507 | 765.6181 | MGG_10118 | 1.38766 | 1.6165385 | 0 | ATPase family AAA domain-containing protein 1 | 2.5 | 213-233 | target 5' CCAGAAUCAAGCC AACCUCAU 3' | :::.:: : ::.:::::::::: | query 3' GGUUUUA-UUUGGUUG GAGUA 5' |
| SI08_SC08 | osa-miR1862f | -1.78909 | 220.82507 | 765.6181 | MGG_06360 | 2.33381 | 4.0413463 | 0 | conserved hypothetical protein (4721 nt) | 4 | 1324-1343 | target 5' CCAUACUCAACCA ACCUCAU 3' | ::: : : ::::::::::: | query 3' GGUUUUAUUUGGUUGG AGUA 5' |
| SI08_SC08 | osa-miR1862g | -1.78909 | 220.82507 | 765.6181 | MGG_10118 | 1.38766 | 1.6165385 | 0 | ATPase family AAA domain-containing protein 1 | 2.5 | 213-233 | target 5' CCAGAAUCAAGCC AACCUCAU 3' | :::.:: : ::.:::::::::: | query 3' GGUUUUA-UUUGGUUG GAGUA 5' |
| SI08_SC08 | osa-miR1862g | -1.78909 | 220.82507 | 765.6181 | MGG_06360 | 2.33381 | 4.0413463 | 0 | conserved hypothetical protein (4721 nt) | 4 | 1324-1343 | target 5' CCAUACUCAACCA ACCUCAU 3' | ::: : : ::::::::::: | query 3' GGUUUUAUUUGGUUGG AGUA 5' |
| SI08_SC08 | osa-miR530-3p | -1.23358 | 293.03801 | 690.43069 | MGG_10153 | 2.54834 | 4.8496155 | 0 | conserved hypothetical protein (1505 nt) | 4 | 1182-1202 | target 5' GAUGGUUCUGCCU CUGCAUCU 3' | : :: ::::::::::::.:: | query 3' CAACGUAGACGGAGAC GUGGA 5' |
| SI08_SC08 | osa-miR530-3p | -1.23358 | 293.03801 | 690.43069 | MGG_01537 | 1.38766 | 1.6165385 | 0 | conserved hypothetical protein (6864 nt) | 4 | 3304-3323 | target 5' AUCGCA-CUGCCUC UGCACCG 3' | : ::: ::::::::::::: | query 3' CAACGUAGACGGAGAC GUGGA 5' |
| SI08_SC08 | osa-miR811d.1 | -2.62951 | 57.561038 | 361.38468 | MGG_03290 | 1.38766 | 1.6165385 | 0 | N-(5-amino-5-carboxypentanoyl)-L-cysteinyl-D- | 3.5 | 2114-2134 | target 5' UGGGCAUAUGGGA UCCGUCCA 3' | :::.:: : .:.:::::::::: | query 3' ACCUGUAGGCUCUAGG CAGGU 5' |
| SI24_SC00 | osa-miR530-3p | -1.75301 | 117.15732 | 397.26208 | MGG_10153 | 2.52806 | 4.7679473 | 0 | conserved hypothetical protein (1505 nt) | 4 | 1182-1202 | target 5' GAUGGUUCUGCCU CUGCAUCU 3' | : :: ::::::::::::.:: | query 3' CAACGUAGACGGAGAC GUGGA 5' |
| SI24_SC00 | osa-miR530-3p | -1.75301 | 117.15732 | 397.26208 | MGG_01537 | 3.39724 | 9.5358947 | 0 | conserved hypothetical protein (6864 nt) | 4 | 3304-3323 | target 5' AUCGCA-CUGCCUC UGCACCG 3' | : ::: ::::::::::::: | query 3' CAACGUAGACGGAGAC GUGGA 5' |
| SI24_SI08 | osa-miR159a.2 | 2.01046 | 142.18122 | 34.536623 | MGG_14003 | -2.0817 | 0 | 3.233077 | ankyrin repeat protein (4561 nt) | 3 | 2089-2110 | target 5' CGCAGUCGCCUGG GGCAUGCAA 3' | :::: : ::::::::::::: | query 3' ACGUC-GAGGACCCCGU ACGUU 5' |
| SI24_SI08 | osa-miR160a | 1.44657 | 908.82235 | 332.80745 | MGG_13163 | -2.0817 | 0 | 3.233077 | conserved hypothetical protein (1031 nt) | 2.5 | 413-434 | target 5' UGGCACUACAGGG AGCUAGGCG 3' | ::::: ::::::::::.::::. | query 3' ACCGU-AUGUCCCUCGG UCCGU 5' |

| | | | | | | | | | | | | | | |
|---|---|---|---|---|---|---|---|---|---|---|---|---|---|---|
| SI24_SI08 | osa-miR160a | 1.44657 | 908.82235 | 332.80745 | MGG_13171 | -2.0817 | 0 | 3.233077 | E3 ubiquitin-protein ligase ubr1 (6712 nt) | 4 | 1075-1095 | target 5'<br>UGGCAUCGAGGAAGCCAGGCA 3' | ::::::<br>::: ::::::::: | query 3'<br>ACCGUAUGUCCCUCGGUCCGU 5' |
| SI24_SI08 | osa-miR160b | 1.44657 | 908.82235 | 332.80745 | MGG_13163 | -2.0817 | 0 | 3.233077 | conserved hypothetical protein (1031 nt) | 2.5 | 413-434 | target 5'<br>UGGCACUACAGGGAGCUAGGCG 3' | :::::<br>:::::::::.::::. | query 3'<br>ACCGU-AUGUCCCUCGGUCCGU 5' |
| SI24_SI08 | osa-miR160b | 1.44657 | 908.82235 | 332.80745 | MGG_13171 | -2.0817 | 0 | 3.233077 | E3 ubiquitin-protein ligase ubr1 (6712 nt) | 4 | 1075-1095 | target 5'<br>UGGCAUCGAGGAAGCCAGGCA 3' | ::::::<br>::: ::::::::: | query 3'<br>ACCGUAUGUCCCUCGGUCCGU 5' |
| SI24_SI08 | osa-miR160c | 1.44657 | 908.82235 | 332.80745 | MGG_13163 | -2.0817 | 0 | 3.233077 | conserved hypothetical protein (1031 nt) | 2.5 | 413-434 | target 5'<br>UGGCACUACAGGGAGCUAGGCG 3' | :::::<br>:::::::::.::::. | query 3'<br>ACCGU-AUGUCCCUCGGUCCGU 5' |
| SI24_SI08 | osa-miR160c | 1.44657 | 908.82235 | 332.80745 | MGG_13171 | -2.0817 | 0 | 3.233077 | E3 ubiquitin-protein ligase ubr1 (6712 nt) | 4 | 1075-1095 | target 5'<br>UGGCAUCGAGGAAGCCAGGCA 3' | ::::::<br>::: ::::::::: | query 3'<br>ACCGUAUGUCCCUCGGUCCGU 5' |
| SI24_SI08 | osa-miR160d | 1.44657 | 908.82235 | 332.80745 | MGG_13163 | -2.0817 | 0 | 3.233077 | conserved hypothetical protein (1031 nt) | 2.5 | 413-434 | target 5'<br>UGGCACUACAGGGAGCUAGGCG 3' | :::::<br>:::::::::.::::. | query 3'<br>ACCGU-AUGUCCCUCGGUCCGU 5' |
| SI24_SI08 | osa-miR160d | 1.44657 | 908.82235 | 332.80745 | MGG_13171 | -2.0817 | 0 | 3.233077 | E3 ubiquitin-protein ligase ubr1 (6712 nt) | 4 | 1075-1095 | target 5'<br>UGGCAUCGAGGAAGCCAGGCA 3' | ::::::<br>::: ::::::::: | query 3'<br>ACCGUAUGUCCCUCGGUCCGU 5' |
| SI24_SI08 | osa-miR160e | 1.30147 | 682.46985 | 276.29298 | MGG_13163 | -2.0817 | 0 | 3.233077 | conserved hypothetical protein (1031 nt) | 3 | 413-434 | target 5'<br>UGGCACUACAGGGAGCUAGGCG 3' | .:::: :<br>::::::::.::::. | query 3'<br>GCCGU-AUGUCCCUCGGUCCGU 5' |
| SI24_SI08 | osa-miR160f | 1.52545 | 727.96784 | 252.222 | MGG_13163 | -2.0817 | 0 | 3.233077 | conserved hypothetical protein (1031 nt) | 3.5 | 413-434 | target 5'<br>UGGCACUACAGGGAGCUAGGCG 3' | :::::<br>: ::::::::.::::. | query 3'<br>ACCGU-AAGUCCCUCGGUCCGU 5' |
| SI24_SI08 | osa-miR160f | 1.52545 | 727.96784 | 252.222 | MGG_13171 | -2.0817 | 0 | 3.233077 | E3 ubiquitin-protein ligase ubr1 (6712 nt) | 4 | 1075-1095 | target 5'<br>UGGCAUCGAGGAAGCCAGGCA 3' | ::::::<br>::: ::::::::: | query 3'<br>ACCGUAAGUCCCUCGGUCCGU 5' |
| SI24_SI08 | osa-miR1850.1 | -1.14442 | 19.336646 | 43.955701 | MGG_08821 | 1.75872 | 2.3839737 | 0 | conserved hypothetical protein (2400 nt) | 4 | 539-558 | target 5'<br>CCACAAU-UCCCAACUUUGCA 3' | :: :::<br>: ::::::::: :: | query 3'<br>GGGGUUAGAGGGUUGAAAGGU 5' |
| SI24_SI08 | osa-miR1860-5p | 1.28895 | 29.573694 | 11.512208 | MGG_12127 | -1.3932 | 9.5358947 | 26.672885 | conserved hypothetical protein (1344 nt) | 3 | 1316-1337 | target 5'<br>GGAGUUUGGAAGCUGGUUUUCG 3' | .:: :.:<br>::::::::::::: | query 3'<br>UCU-AGACCUUCGACCAAAAGA 5' |
| SI24_SI08 | osa-miR1862f | 1.22186 | 516.40219 | 220.82507 | MGG_10118 | -1.3877 | 0 | 1.6165385 | ATPase family AAA domain-containing protein 1 | 2.5 | 213-233 | target 5'<br>CCAGAAUCAAGCCAACCUCAU 3' | :::.::<br>: ::.:::::::::: | query 3'<br>GGUUUUA-UUUGGUUGGAGUA 5' |
| SI24_SI08 | osa-miR1862f | 1.22186 | 516.40219 | 220.82507 | MGG_06360 | -2.3338 | 0 | 4.0413463 | conserved hypothetical protein (4721 nt) | 4 | 1324-1343 | target 5'<br>CCAUACUCAACCAACCUCAU 3' | ::: : :<br>:::::::::::: | query 3'<br>GGUUUUAUUUGGUUGGAGUA 5' |
| SI24_SI08 | osa-miR1862g | 1.22186 | 516.40219 | 220.82507 | MGG_10118 | -1.3877 | 0 | 1.6165385 | ATPase family AAA domain-containing protein 1 | 2.5 | 213-233 | target 5'<br>CCAGAAUCAAGCCAACCUCAU 3' | :::.::<br>: ::.:::::::::: | query 3'<br>GGUUUUA-UUUGGUUGGAGUA 5' |
| SI24_SI08 | osa-miR1862g | 1.22186 | 516.40219 | 220.82507 | MGG_06360 | -2.3338 | 0 | 4.0413463 | conserved hypothetical protein (4721 nt) | 4 | 1324-1343 | target 5'<br>CCAUACUCAACCAACCUCAU 3' | ::: : :<br>:::::::::::: | query 3'<br>GGUUUUAUUUGGUUGGAGUA 5' |
| SI24_SI08 | osa-miR2055 | 1.11973 | 126.25692 | 57.561038 | MGG_06905 | -2.5483 | 0 | 4.8496155 | protein transport protein sec73 (4680 nt) | 3 | 1735-1756 | target 5'<br>GAAGCUGAGCUUCCCAAGGAAA 3' | :::.:.<br>: ::::::::::::: | query 3'<br>CUUUGG-UGGAAGGGUUCCUUU 5' |
| SI24_SI08 | osa-miR2055 | 1.11973 | 126.25692 | 57.561038 | MGG_04685 | -3.1831 | 0 | 8.0826926 | conserved hypothetical protein (4541 nt) | 4 | 1767-1788 | target 5'<br>GACGAUAUCCUUCCCAAGGAAA 3' | :: .:.<br>:::::::::::::: | query 3'<br>CU-UUGGUGGAAGGGUUCCUUU 5' |
| SI24_SI08 | osa-miR396c | 1.06981 | 961.14504 | 457.34861 | MGG_08526 | -2.7791 | 65.559276 | 455.86386 | hypothetical protein (2024 nt) | 4 | 1874-1894 | target 5'<br>GGGUUCAAAAAAGUUGUGGAA 3' | ..:::::<br>: ::::.::::::: | query 3'<br>UUCAAGUUCUUUCGACACCUU 5' |

| | | | | | | | | | | | | | | |
|---|---|---|---|---|---|---|---|---|---|---|---|---|---|---|
| SI24_S<br>I08 | osa-miR4<br>44d.3 | 1.04<br>91 | 89.85<br>853 | 42.90<br>9137 | MGG_<br>14775 | -1.6<br>028 | 1.191<br>9868 | 5.657<br>8848 | conserved hypothetical<br>protein (3712 nt) | 4 | 3018-<br>3038 | target 5'<br>CCACUAGCAAGAA<br>AGCCAUGA 3' | : ::: :<br>:::::::::::..: | query 3'<br>GUUGAACGUUCUUUCG<br>GUGUU 5' |
| SI24_S<br>I08 | osa-miR5<br>30-3p | -1.31<br>529 | 117.1<br>5732 | 293.0<br>3801 | MGG_<br>01537 | 2.00<br>958 | 9.535<br>8947 | 1.616<br>5385 | conserved hypothetical<br>protein (6864 nt) | 4 | 3304-<br>3323 | target 5'<br>AUCGCA-CUGCCUC<br>UGCACCG 3' | : :::<br>::::::::::::: | query 3'<br>CAACGUAGACGGAGAC<br>GUGGA 5' |
| SI24_S<br>I08 | osa-miR5<br>508 | 1.32<br>139 | 77.34<br>6583 | 30.35<br>0365 | MGG_<br>14752 | -1.7<br>76 | 0 | 2.424<br>8078 | conserved hypothetical<br>protein (3554 nt) | 4 | 846-8<br>66 | target 5'<br>CCACGGAGGAUCA<br>GCCAUCUU 3' | ::::.<br>.:::::::::::: | query 3'<br>GGUGUGGUCUAGUCGG<br>UAGAU 5' |
| SI48_S<br>C00 | osa-miR1<br>433 | -2.99<br>396 | 18.80<br>6838 | 156.7<br>926 | MGG_<br>03910 | 3.27<br>325 | 8.668<br>2408 | 0 | conserved hypothetical<br>protein (1339 nt) | 4 | 132-1<br>51 | target 5'<br>GGCAGACGAGGAG<br>A-UUGCCA 3' | :: :: :<br>::::::: :::::: | query 3'<br>CCAUCGGCUCCUCUGA<br>ACGGU 5' |
| SI48_S<br>C00 | osa-miR1<br>433 | -2.99<br>396 | 18.80<br>6838 | 156.7<br>926 | MGG_<br>00843 | 5.60<br>512 | 47.67<br>5324 | 0 | regulatory protein suaprga1<br>(1281 nt) | 4 | 296-3<br>15 | target 5'<br>GGUUGACGAGGAG-<br>CUUGCCA 3' | ::: : :<br>:::::: ::::::: | query 3'<br>CCAUCGGCUCCUCUGA<br>ACGGU 5' |
| SI48_S<br>C00 | osa-miR1<br>62b | -1.41<br>755 | 510.2<br>9221 | 1364.<br>8268 | MGG_<br>08092 | 4.38<br>798 | 19.93<br>6954 | 0 | conserved hypothetical<br>protein (4337 nt) | 4 | 4120-<br>4141 | target 5'<br>CUGUGAAGCAGAA<br>GCUUAUCGA 3' | ::: ::<br>::::: ::::::::: | query 3'<br>GAC-CUACGUCUCCGAA<br>UAGCU 5' |
| SI48_S<br>C00 | osa-miR1<br>66a | -1.88<br>318 | 54169<br>.963 | 19982<br>8.51 | MGG_<br>14929 | 3.61<br>691 | 11.26<br>8713 | 0 | conserved hypothetical<br>protein (833 nt) | 4 | 541-5<br>60 | target 5'<br>GGGGAA-GAGGCCU<br>GGUUCGC 3' | ::::::<br>::.::::::::.:: | query 3'<br>CCCCUUACUUCGGACC<br>AGGCU 5' |
| SI48_S<br>C00 | osa-miR1<br>66b | -1.88<br>318 | 54169<br>.963 | 19982<br>8.51 | MGG_<br>14929 | 3.61<br>691 | 11.26<br>8713 | 0 | conserved hypothetical<br>protein (833 nt) | 4 | 541-5<br>60 | target 5'<br>GGGGAA-GAGGCCU<br>GGUUCGC 3' | ::::::<br>::.::::::::.:: | query 3'<br>CCCCUUACUUCGGACC<br>AGGCU 5' |
| SI48_S<br>C00 | osa-miR1<br>66c | -1.88<br>318 | 54169<br>.963 | 19982<br>8.51 | MGG_<br>14929 | 3.61<br>691 | 11.26<br>8713 | 0 | conserved hypothetical<br>protein (833 nt) | 4 | 541-5<br>60 | target 5'<br>GGGGAA-GAGGCCU<br>GGUUCGC 3' | ::::::<br>::.::::::::.:: | query 3'<br>CCCCUUACUUCGGACC<br>AGGCU 5' |
| SI48_S<br>C00 | osa-miR1<br>66d | -1.88<br>318 | 54169<br>.963 | 19982<br>8.51 | MGG_<br>14929 | 3.61<br>691 | 11.26<br>8713 | 0 | conserved hypothetical<br>protein (833 nt) | 4 | 541-5<br>60 | target 5'<br>GGGGAA-GAGGCCU<br>GGUUCGC 3' | ::::::<br>::.::::::::.:: | query 3'<br>CCCCUUACUUCGGACC<br>AGGCU 5' |
| SI48_S<br>C00 | osa-miR1<br>66e | -1.86<br>057 | 40182<br>.69 | 14592<br>6.79 | MGG_<br>14929 | 3.61<br>691 | 11.26<br>8713 | 0 | conserved hypothetical<br>protein (833 nt) | 3 | 541-5<br>60 | target 5'<br>GGGGAA-GAGGCCU<br>GGUUCGC 3' | ::::::<br>::.:::::::::: | query 3'<br>CCCCUUACUUCGGACC<br>AAGCU 5' |
| SI48_S<br>C00 | osa-miR1<br>66f | -1.88<br>318 | 54169<br>.963 | 19982<br>8.51 | MGG_<br>14929 | 3.61<br>691 | 11.26<br>8713 | 0 | conserved hypothetical<br>protein (833 nt) | 4 | 541-5<br>60 | target 5'<br>GGGGAA-GAGGCCU<br>GGUUCGC 3' | ::::::<br>::.::::::::.:: | query 3'<br>CCCCUUACUUCGGACC<br>AGGCU 5' |
| SI48_S<br>C00 | osa-miR1<br>66i | -1.79<br>826 | 12497<br>.771 | 43469<br>.733 | MGG_<br>08115 | 2.41<br>525 | 4.334<br>1204 | 0 | NAD-binding Rossmann<br>fold oxidoreductase fami | 3.5 | 1143-<br>1164 | target 5'<br>GAGGCUGGGAAGC<br>CUGAUCCGA 3' | ::::<br>. ::::::::::::: | query 3'<br>CUCC-UUACUUCGGACU<br>AGGCU 5' |
| SI48_S<br>C00 | osa-miR1<br>66j | -1.79<br>826 | 12497<br>.771 | 43469<br>.733 | MGG_<br>08115 | 2.41<br>525 | 4.334<br>1204 | 0 | NAD-binding Rossmann<br>fold oxidoreductase fami | 3.5 | 1143-<br>1164 | target 5'<br>GAGGCUGGGAAGC<br>CUGAUCCGA 3' | ::::<br>. ::::::::::::: | query 3'<br>CUCC-UUACUUCGGACU<br>AGGCU 5' |
| SI48_S<br>C00 | osa-miR1<br>66n | -1.88<br>318 | 54169<br>.963 | 19982<br>8.51 | MGG_<br>14929 | 3.61<br>691 | 11.26<br>8713 | 0 | conserved hypothetical<br>protein (833 nt) | 4 | 541-5<br>60 | target 5'<br>GGGGAA-GAGGCCU<br>GGUUCGC 3' | ::::::<br>::.::::::::.:: | query 3'<br>CCCCUUACUUCGGACC<br>AGGCU 5' |
| SI48_S<br>C00 | osa-miR1<br>67d | -1.10<br>609 | 32163<br>.455 | 69236<br>.526 | MGG_<br>02705 | 3.97<br>6 | 14.73<br>6009 | 0 | conserved hypothetical<br>protein (2862 nt) | 4 | 1421-<br>1440 | target 5'<br>CAGA-CAUGCUGGC<br>GGCUUUC 3' | :::: :<br>:::::::::.::::. | query 3'<br>GUCUAGUACGACCGUC<br>GAAGU 5' |
| SI48_S<br>C00 | osa-miR1<br>67e | -1.10<br>609 | 32163<br>.455 | 69236<br>.526 | MGG_<br>02705 | 3.97<br>6 | 14.73<br>6009 | 0 | conserved hypothetical<br>protein (2862 nt) | 4 | 1421-<br>1440 | target 5'<br>CAGA-CAUGCUGGC<br>GGCUUUC 3' | :::: :<br>:::::::::.::::. | query 3'<br>GUCUAGUACGACCGUC<br>GAAGU 5' |
| SI48_S<br>C00 | osa-miR1<br>67f | -1.10<br>609 | 32163<br>.455 | 69236<br>.526 | MGG_<br>02705 | 3.97<br>6 | 14.73<br>6009 | 0 | conserved hypothetical<br>protein (2862 nt) | 4 | 1421-<br>1440 | target 5'<br>CAGA-CAUGCUGGC<br>GGCUUUC 3' | :::: :<br>:::::::::.::::. | query 3'<br>GUCUAGUACGACCGUC<br>GAAGU 5' |
| SI48_S<br>C00 | osa-miR1<br>67g | -1.10<br>609 | 32163<br>.455 | 69236<br>.526 | MGG_<br>02705 | 3.97<br>6 | 14.73<br>6009 | 0 | conserved hypothetical<br>protein (2862 nt) | 4 | 1421-<br>1440 | target 5'<br>CAGA-CAUGCUGGC<br>GGCUUUC 3' | :::: :<br>:::::::::.::::. | query 3'<br>GUCUAGUACGACCGUC<br>GAAGU 5' |

| | | | | | | | | | | | | | | |
|---|---|---|---|---|---|---|---|---|---|---|---|---|---|---|
| SI48_SC00 | osa-miR167h | -1.10609 | 32163.455 | 69236.526 | MGG_02705 | 3.976 | 14.736009 | 0 | conserved hypothetical protein (2862 nt) | 4 | 1421-1440 | target 5' CAGA-CAUGCUGGC GGCUUUC 3' | :::: : :::::::::.::::. | query 3' GUCUAGUACGACCGUC GAAGU 5' |
| SI48_SC00 | osa-miR167i | -1.10609 | 32163.455 | 69236.526 | MGG_02705 | 3.976 | 14.736009 | 0 | conserved hypothetical protein (2862 nt) | 4 | 1421-1440 | target 5' CAGA-CAUGCUGGC GGCUUUC 3' | :::: : :::::::::.::::. | query 3' GUCUAGUACGACCGUC GAAGU 5' |
| SI48_SC00 | osa-miR167j | -1.10609 | 32163.455 | 69236.526 | MGG_02705 | 3.976 | 14.736009 | 0 | conserved hypothetical protein (2862 nt) | 4 | 1421-1440 | target 5' CAGA-CAUGCUGGC GGCUUUC 3' | :::: : :::::::::.::::. | query 3' GUCUAGUACGACCGUC GAAGU 5' |
| SI48_SC00 | osa-miR169e | -1.04226 | 1677.57 | 3455.9364 | MGG_06752 | 3.8076 | 13.002361 | 0 | peptidase family M28 family (3293 nt) | 4 | 1787-1807 | target 5' CUGGCUCUUCAUC CUUGGCUG 3' | :.::: ::::::::::::. | query 3' GGCCGUUCAGUAGGAA CCGAU 5' |
| SI48_SC00 | osa-miR2867-3p | -1.39234 | 48.897779 | 129.9835 | MGG_14778 | 1.45083 | 1.7336482 | 0 | leptomycin B resistance protein pmd1 (4387 nt | 3.5 | 1328-1348 | target 5' UGCGUAUCCCACU CGUCCUGG 3' | ::: .:: ::::: :::::::: | query 3' ACG-GUAGGGUGUGCA GGACC 5' |
| SI48_SC00 | osa-miR2867-3p | -1.39234 | 48.897779 | 129.9835 | MGG_06107 | 5.5528 | 45.941676 | 0 | transcription initiation factor IIA gamma cha | 4 | 553-572 | target 5' UGCAAUGCCAAAC GUCCUGG 3' | ::: :: ::: ::::::::: | query 3' ACGGUAGGGUGUGCAG GACC 5' |
| SI48_SC00 | osa-miR2867-3p | -1.39234 | 48.897779 | 129.9835 | MGG_14970 | 2.1594 | 3.4672963 | 0 | carnitinyl-CoA dehydratase (1101 nt) | 4 | 104-124 | target 5' UUCCAUCACCGCAC GUCCUGC 3' | : :::: : ::.::::::::: | query 3' ACGGUAG-GGUGUGCA GGACC 5' |
| SI48_SC00 | osa-miR396a | -1.02944 | 531.60663 | 1086.1747 | MGG_01046 | 5.98667 | 62.411334 | 0 | methionyl-tRNA synthetase (2588 nt) | 3.5 | 2556-2576 | target 5' CAGCUUGAGAAGG CUGUGGAG 3' | ::: :.. ::::.::::::::. | query 3' GUCAAGUUCUUUCGAC ACCUU 5' |
| SI48_SC00 | osa-miR396b | -1.02944 | 531.60663 | 1086.1747 | MGG_01046 | 5.98667 | 62.411334 | 0 | methionyl-tRNA synthetase (2588 nt) | 3.5 | 2556-2576 | target 5' CAGCUUGAGAAGG CUGUGGAG 3' | ::: :.. ::::.::::::::. | query 3' GUCAAGUUCUUUCGAC ACCUU 5' |
| SI48_SC00 | osa-miR396c | -1.04705 | 535.36799 | 1107.297 | MGG_03303 | 2.41525 | 4.3341204 | 0 | conserved hypothetical protein (2454 nt) | 3.5 | 822-841 | target 5' AAGUUCGAG-AAGU UGUGGAA 3' | :::::: .:: :::.::::::: | query 3' UUCAAGUUCUUUCGAC ACCUU 5' |
| SI48_SC00 | osa-miR396c | -1.04705 | 535.36799 | 1107.297 | MGG_06547 | 6.00626 | 63.278158 | 0 | tartrate dehydrogenase/decarboxylase (1258 nt | 4 | 1108-1128 | target 5' AAGCUCCUGGAAG CUGUGGAA 3' | ::: :: :.:::::::::: | query 3' UUCAAGUUCUUUCGAC ACCUU 5' |
| SI48_SC00 | osa-miR5161 | -1.37919 | 2.5075784 | 8.123969 | MGG_02949 | 4.92941 | 29.472019 | 0 | conserved hypothetical protein (3524 nt) | 4 | 461-479 | target 5' UAUGGU-CCUUUGA UCCAGG 3' | :::. : :::.::::::::. | query 3' AUAUGAGGGAGACUAG GUCU 5' |
| SI48_SC00 | osa-miR5161 | -1.37919 | 2.5075784 | 8.123969 | MGG_02732 | 2.98816 | 6.9345926 | 0 | conserved hypothetical protein (1700 nt) | 4 | 1168-1186 | target 5' AAGACU-CCUCUGA UCCAGC 3' | : ::: :::::::::::: | query 3' AUAUGAGGGAGACUAG GUCU 5' |
| SI48_SC00 | osa-miR820a | -1.19579 | 50.151568 | 116.17276 | MGG_11519 | 4.32698 | 19.07013 | 0 | alcohol dehydrogenase (1729 nt) | 3 | 745-765 | target 5' CUGGUCCUUCCGC GAGGCCGC 3' | :::::: : :::.::::::::: | query 3' GACCAGGUAGGUGCUC CGGCU 5' |
| SI48_SC00 | osa-miR820a | -1.19579 | 50.151568 | 116.17276 | MGG_09559 | 3.61691 | 11.268713 | 0 | autophagy-related protein 9 (3015 nt) | 3 | 410-430 | target 5' CUGGACCAUCCAC GAGGCAGA 3' | :::: : :::::::::::: :: | query 3' GACCAGGUAGGUGCUC CGGCU 5' |
| SI48_SC00 | osa-miR820a | -1.19579 | 50.151568 | 116.17276 | MGG_04090 | 5.44209 | 42.47438 | 0 | SNARE protein [Neurospora crassa OR74A]) (234 | 4 | 654-674 | target 5' CAGGUCCCUCAAC GAGGCCGA 3' | : :::: : :: :::::::::: | query 3' GACCAGGUAGGUGCUC CGGCU 5' |
| SI48_SC00 | osa-miR820a | -1.19579 | 50.151568 | 116.17276 | MGG_08117 | 4.12678 | 16.469658 | 0 | DRAP deaminase (1625 nt) | 4 | 618-637 | target 5' CAGGUCC-UCCACG AGGACGA 3' | : :::: : ::::::::: ::: | query 3' GACCAGGUAGGUGCUC CGGCU 5' |
| SI48_SC00 | osa-miR820a | -1.19579 | 50.151568 | 116.17276 | MGG_02417 | 4.80072 | 26.871546 | 0 | ATP-dependent rRNA helicase spb-4 (2165 nt) | 4 | 714-733 | target 5' CUGGUCC-UCGACG AGGCUGA 3' | :::::: : :: ::::::::.:: | query 3' GACCAGGUAGGUGCUC CGGCU 5' |
| SI48_SC00 | osa-miR820a | -1.19579 | 50.151568 | 116.17276 | MGG_01706 | 7.43155 | 171.63117 | 0 | hypothetical protein (773 nt) | 4 | 352-373 | target 5' CUGCUUCGUCCGA CGAGGCCGA 3' | ::: :.: .::: :::::::::: | query 3' GACCAGGUAGG-UGCU CCGGCU 5' |

| | | | | | | | | | | | | | | |
|---|---|---|---|---|---|---|---|---|---|---|---|---|---|---|
| SI48_SC00 | osa-miR820b | -1.19579 | 50.151568 | 116.17276 | MGG_11519 | 4.32698 | 19.07013 | 0 | alcohol dehydrogenase (1729 nt) | 3 | 745-765 | target 5'<br>CUGGUCCUUCCGC<br>GAGGCCGC 3' | ::::::<br>: :::.:::::::: | query 3'<br>GACCAGGUAGGUGCUC<br>CGGCU 5' |
| SI48_SC00 | osa-miR820b | -1.19579 | 50.151568 | 116.17276 | MGG_09559 | 3.61691 | 11.268713 | 0 | autophagy-related protein 9 (3015 nt) | 3 | 410-430 | target 5'<br>CUGGACCAUCCAC<br>GAGGCAGA 3' | ::::  :<br>::::::::::: :: | query 3'<br>GACCAGGUAGGUGCUC<br>CGGCU 5' |
| SI48_SC00 | osa-miR820b | -1.19579 | 50.151568 | 116.17276 | MGG_04090 | 5.44209 | 42.47438 | 0 | SNARE protein [Neurospora crassa OR74A]) (234 | 4 | 654-674 | target 5'<br>CAGGUCCCUCAAC<br>GAGGCCGA 3' | :  ::::<br>: :: ::::::::: | query 3'<br>GACCAGGUAGGUGCUC<br>CGGCU 5' |
| SI48_SC00 | osa-miR820b | -1.19579 | 50.151568 | 116.17276 | MGG_08117 | 4.12678 | 16.469658 | 0 | DRAP deaminase (1625 nt) | 4 | 618-637 | target 5'<br>CAGGUCC-UCCACG<br>AGGACGA 3' | :  ::::<br>: :::::::: ::: | query 3'<br>GACCAGGUAGGUGCUC<br>CGGCU 5' |
| SI48_SC00 | osa-miR820b | -1.19579 | 50.151568 | 116.17276 | MGG_02417 | 4.80072 | 26.871546 | 0 | ATP-dependent rRNA helicase spb-4 (2165 nt) | 4 | 714-733 | target 5'<br>CUGGUCC-UCGACG<br>AGGCUGA 3' | ::::::<br>: :: :::::::.:: | query 3'<br>GACCAGGUAGGUGCUC<br>CGGCU 5' |
| SI48_SC00 | osa-miR820b | -1.19579 | 50.151568 | 116.17276 | MGG_01706 | 7.43155 | 171.63117 | 0 | hypothetical protein (773 nt) | 4 | 352-373 | target 5'<br>CUGCUUCGUCCGA<br>CGAGGCCGA 3' | ::: :.:<br>.::: ::::::::: | query 3'<br>GACCAGGUAGG-UGCU<br>CCGGCU 5' |
| SI48_SC00 | osa-miR820c | -1.19579 | 50.151568 | 116.17276 | MGG_11519 | 4.32698 | 19.07013 | 0 | alcohol dehydrogenase (1729 nt) | 3 | 745-765 | target 5'<br>CUGGUCCUUCCGC<br>GAGGCCGC 3' | ::::::<br>: :::.:::::::: | query 3'<br>GACCAGGUAGGUGCUC<br>CGGCU 5' |
| SI48_SC00 | osa-miR820c | -1.19579 | 50.151568 | 116.17276 | MGG_09559 | 3.61691 | 11.268713 | 0 | autophagy-related protein 9 (3015 nt) | 3 | 410-430 | target 5'<br>CUGGACCAUCCAC<br>GAGGCAGA 3' | ::::  :<br>::::::::::: :: | query 3'<br>GACCAGGUAGGUGCUC<br>CGGCU 5' |
| SI48_SC00 | osa-miR820c | -1.19579 | 50.151568 | 116.17276 | MGG_04090 | 5.44209 | 42.47438 | 0 | SNARE protein [Neurospora crassa OR74A]) (234 | 4 | 654-674 | target 5'<br>CAGGUCCCUCAAC<br>GAGGCCGA 3' | :  ::::<br>: :: ::::::::: | query 3'<br>GACCAGGUAGGUGCUC<br>CGGCU 5' |
| SI48_SC00 | osa-miR820c | -1.19579 | 50.151568 | 116.17276 | MGG_08117 | 4.12678 | 16.469658 | 0 | DRAP deaminase (1625 nt) | 4 | 618-637 | target 5'<br>CAGGUCC-UCCACG<br>AGGACGA 3' | :  ::::<br>: :::::::: ::: | query 3'<br>GACCAGGUAGGUGCUC<br>CGGCU 5' |
| SI48_SC00 | osa-miR820c | -1.19579 | 50.151568 | 116.17276 | MGG_02417 | 4.80072 | 26.871546 | 0 | ATP-dependent rRNA helicase spb-4 (2165 nt) | 4 | 714-733 | target 5'<br>CUGGUCC-UCGACG<br>AGGCUGA 3' | ::::::<br>: :: :::::::.:: | query 3'<br>GACCAGGUAGGUGCUC<br>CGGCU 5' |
| SI48_SC00 | osa-miR820c | -1.19579 | 50.151568 | 116.17276 | MGG_01706 | 7.43155 | 171.63117 | 0 | hypothetical protein (773 nt) | 4 | 352-373 | target 5'<br>CUGCUUCGUCCGA<br>CGAGGCCGA 3' | ::: :.:<br>.::: ::::::::: | query 3'<br>GACCAGGUAGG-UGCU<br>CCGGCU 5' |
| SI48_SI24 | osa-miR1433 | -2.69651 | 18.806838 | 127.39437 | MGG_03910 | 3.27325 | 8.6682408 | 0 | conserved hypothetical protein (1339 nt) | 4 | 132-151 | target 5'<br>GGCAGACGAGGAG<br>A-UUGCCA 3' | :: :: :<br>::::::: :::::: | query 3'<br>CCAUCGGCUCCUCUGA<br>ACGGU 5' |
| SI48_SI24 | osa-miR1433 | -2.69651 | 18.806838 | 127.39437 | MGG_00843 | 3.8464 | 47.675324 | 2.3839737 | regulatory protein suaprga1 (1281 nt) | 4 | 296-315 | target 5'<br>GGUUGACGAGGAG-<br>CUUGCCA 3' | ::: : :<br>:::::: ::::::: | query 3'<br>CCAUCGGCUCCUCUGA<br>ACGGU 5' |
| SI48_SI24 | osa-miR162b | -1.62126 | 510.29221 | 1571.9556 | MGG_08092 | 4.38798 | 19.936954 | 0 | conserved hypothetical protein (4337 nt) | 4 | 4120-4141 | target 5'<br>CUGUGAAGCAGAA<br>GCUUAUCGA 3' | :::  ::<br>::::: ::::::::: | query 3'<br>GAC-CUACGUCUCCGAA<br>UAGCU 5' |
| SI48_SI24 | osa-miR166a | -1.76845 | 54169.963 | 184552.36 | MGG_14929 | 3.61691 | 11.268713 | 0 | conserved hypothetical protein (833 nt) | 4 | 541-560 | target 5'<br>GGGGAA-GAGGCCU<br>GGUUCGC 3' | ::::::<br>::.:::::::.:: | query 3'<br>CCCCUUACUUCGGACC<br>AGGCU 5' |
| SI48_SI24 | osa-miR166b | -1.76845 | 54169.963 | 184552.36 | MGG_14929 | 3.61691 | 11.268713 | 0 | conserved hypothetical protein (833 nt) | 4 | 541-560 | target 5'<br>GGGGAA-GAGGCCU<br>GGUUCGC 3' | ::::::<br>::.:::::::.:: | query 3'<br>CCCCUUACUUCGGACC<br>AGGCU 5' |
| SI48_SI24 | osa-miR166c | -1.76845 | 54169.963 | 184552.36 | MGG_14929 | 3.61691 | 11.268713 | 0 | conserved hypothetical protein (833 nt) | 4 | 541-560 | target 5'<br>GGGGAA-GAGGCCU<br>GGUUCGC 3' | ::::::<br>::.:::::::.:: | query 3'<br>CCCCUUACUUCGGACC<br>AGGCU 5' |
| SI48_SI24 | osa-miR166d | -1.76845 | 54169.963 | 184552.36 | MGG_14929 | 3.61691 | 11.268713 | 0 | conserved hypothetical protein (833 nt) | 4 | 541-560 | target 5'<br>GGGGAA-GAGGCCU<br>GGUUCGC 3' | ::::::<br>::.:::::::.:: | query 3'<br>CCCCUUACUUCGGACC<br>AGGCU 5' |

| | | | | | | | | | | | | | | |
|---|---|---|---|---|---|---|---|---|---|---|---|---|---|---|
| SI48_SI24 | osa-miR166e | -1.70778 | 40182.69 | 131261.7 | MGG_14929 | 3.61691 | 11.268713 | 0 | conserved hypothetical protein (833 nt) | 3 | 541-560 | target 5' GGGGAA-GAGGCCUGGUUCGC 3' | ::::::<br>::.::::::::: | query 3' CCCCUUACUUCGGACCAAGCU 5' |
| SI48_SI24 | osa-miR166f | -1.76845 | 54169.963 | 184552.36 | MGG_14929 | 3.61691 | 11.268713 | 0 | conserved hypothetical protein (833 nt) | 4 | 541-560 | target 5' GGGGAA-GAGGCCUGGUUCGC 3' | ::::::<br>::.:::::::.:: | query 3' CCCCUUACUUCGGACCAGGCU 5' |
| SI48_SI24 | osa-miR166i | -1.83817 | 12497.771 | 44689.263 | MGG_08115 | 1.28301 | 4.3341204 | 1.1919868 | NAD-binding Rossmann fold oxidoreductase fami | 3.5 | 1143-1164 | target 5' GAGGCUGGGAAGCCUGAUCCGA 3' | ::::<br>. ::::::::::::: | query 3' CUCC-UUACUUCGGACUAGGCU 5' |
| SI48_SI24 | osa-miR166j | -1.83817 | 12497.771 | 44689.263 | MGG_08115 | 1.28301 | 4.3341204 | 1.1919868 | NAD-binding Rossmann fold oxidoreductase fami | 3.5 | 1143-1164 | target 5' GAGGCUGGGAAGCCUGAUCCGA 3' | ::::<br>. ::::::::::::: | query 3' CUCC-UUACUUCGGACUAGGCU 5' |
| SI48_SI24 | osa-miR166n | -1.76845 | 54169.963 | 184552.36 | MGG_14929 | 3.61691 | 11.268713 | 0 | conserved hypothetical protein (833 nt) | 4 | 541-560 | target 5' GGGGAA-GAGGCCUGGUUCGC 3' | ::::::<br>::.:::::::.:: | query 3' CCCCUUACUUCGGACCAGGCU 5' |
| SI48_SI24 | osa-miR167d | -1.54805 | 32163.455 | 94054.582 | MGG_02705 | 3.976 | 14.736009 | 0 | conserved hypothetical protein (2862 nt) | 4 | 1421-1440 | target 5' CAGA-CAUGCUGGCGGCUUUC 3' | :::: :<br>:::::::::.::::. | query 3' GUCUAGUACGACCGUCGAAGU 5' |
| SI48_SI24 | osa-miR167e | -1.54805 | 32163.455 | 94054.582 | MGG_02705 | 3.976 | 14.736009 | 0 | conserved hypothetical protein (2862 nt) | 4 | 1421-1440 | target 5' CAGA-CAUGCUGGCGGCUUUC 3' | :::: :<br>:::::::::.::::. | query 3' GUCUAGUACGACCGUCGAAGU 5' |
| SI48_SI24 | osa-miR167f | -1.54805 | 32163.455 | 94054.582 | MGG_02705 | 3.976 | 14.736009 | 0 | conserved hypothetical protein (2862 nt) | 4 | 1421-1440 | target 5' CAGA-CAUGCUGGCGGCUUUC 3' | :::: :<br>:::::::::.::::. | query 3' GUCUAGUACGACCGUCGAAGU 5' |
| SI48_SI24 | osa-miR167g | -1.54805 | 32163.455 | 94054.582 | MGG_02705 | 3.976 | 14.736009 | 0 | conserved hypothetical protein (2862 nt) | 4 | 1421-1440 | target 5' CAGA-CAUGCUGGCGGCUUUC 3' | :::: :<br>:::::::::.::::. | query 3' GUCUAGUACGACCGUCGAAGU 5' |
| SI48_SI24 | osa-miR167h | -1.54805 | 32163.455 | 94054.582 | MGG_02705 | 3.976 | 14.736009 | 0 | conserved hypothetical protein (2862 nt) | 4 | 1421-1440 | target 5' CAGA-CAUGCUGGCGGCUUUC 3' | :::: :<br>:::::::::.::::. | query 3' GUCUAGUACGACCGUCGAAGU 5' |
| SI48_SI24 | osa-miR167i | -1.54805 | 32163.455 | 94054.582 | MGG_02705 | 3.976 | 14.736009 | 0 | conserved hypothetical protein (2862 nt) | 4 | 1421-1440 | target 5' CAGA-CAUGCUGGCGGCUUUC 3' | :::: :<br>:::::::::.::::. | query 3' GUCUAGUACGACCGUCGAAGU 5' |
| SI48_SI24 | osa-miR167j | -1.54805 | 32163.455 | 94054.582 | MGG_02705 | 3.976 | 14.736009 | 0 | conserved hypothetical protein (2862 nt) | 4 | 1421-1440 | target 5' CAGA-CAUGCUGGCGGCUUUC 3' | :::: :<br>:::::::::.::::. | query 3' GUCUAGUACGACCGUCGAAGU 5' |
| SI48_SI24 | osa-miR1860-5p | -1.6449 | 8.7765245 | 29.573694 | MGG_12127 | 3.43168 | 112.68713 | 9.5358947 | conserved hypothetical protein (1344 nt) | 3 | 1316-1337 | target 5' GGAGUUUGGAAGCUGGUUUUCG 3' | .:: :.:<br>::::::::::::: | query 3' UCU-AGACCUUCGACCAAAAGA 5' |
| SI48_SI24 | osa-miR1876 | -1.33671 | 42.628833 | 109.19518 | MGG_01537 | 4.39179 | 220.17332 | 9.5358947 | conserved hypothetical protein (6864 nt) | 4 | 4963-4985 | target 5' UGGCCAGCCCACAAGCCCA-UUAU 3' | :::::<br>::::::::.:::: :::: | query 3' CCCGGUCGGGUGUUUGGGUGAAUA 5' |
| SI48_SI24 | osa-miR2867-3p | -1.3507 | 48.897779 | 126.25692 | MGG_14778 | 1.45083 | 1.7336482 | 0 | leptomycin B resistance protein pmd1 (4387 nt | 3.5 | 1328-1348 | target 5' UGCGUAUCCCACUCGUCCUGG 3' | ::: .::<br>::::: :::::::: | query 3' ACG-GUAGGGUGUGCAGGACC 5' |
| SI48_SI24 | osa-miR2867-3p | -1.3507 | 48.897779 | 126.25692 | MGG_06107 | 1.73396 | 45.941676 | 13.111855 | transcription initiation factor IIA gamma cha | 4 | 553-572 | target 5' UGCAAUGCCAAACGUCCUGG 3' | ::: ::<br>::: ::::::::: | query 3' ACGGUAGGGUGUGCAGGACC 5' |
| SI48_SI24 | osa-miR390 | -1.00617 | 135.40923 | 272.98794 | MGG_00351 | 1.84819 | 2.6004722 | 0 | coiled-coil domain-containing protein MTMR15 | 3 | 860-880 | target 5' GGCCUUGUCUCUCCUGAGCUU 3' | ::: .:.<br>::.::::::::::: | query 3' CCGCGAUAGGGAGGACUCGAA 5' |
| SI48_SI24 | osa-miR399d | -1.05293 | 493.99295 | 1025.9797 | MGG_10741 | 2.63249 | 5.2009445 | 0 | conserved hypothetical protein (1692 nt) | 3.5 | 489-508 | target 5' CA-GUCGGCUCUCCUUUGGCG 3' | :: : :.<br>.:::::::::::. | query 3' GUCCCGUUGAGAGGAAACCGU 5' |
| SI48_SI24 | osa-miR399d | -1.05293 | 493.99295 | 1025.9797 | MGG_03433 | 2.63249 | 5.2009445 | 0 | averantin oxidoreductase (1833 nt) | 4 | 1506-1526 | target 5' CAUGGCAACCCUCUUUUGGCA 3' | :: :::<br>::: :::.::::::: | query 3' GUCCCGUUGAGAGGAAACCGU 5' |

| | | | | | | | | | | | | | | |
|---|---|---|---|---|---|---|---|---|---|---|---|---|---|---|
| SI48_SI24 | osa-miR399i | -1.09439 | 329.74656 | 705.21885 | MGG_10741 | 2.63249 | 5.2009445 | 0 | conserved hypothetical protein (1692 nt) | 3 | 489-508 | target 5' CA-GUCGGCUCUCCUUUGGCG 3' | :: : :. ::::::::::::. | query 3' GUCCCGUCGAGAGGAAACCGU 5' |
| SI48_SI24 | osa-miR444a-5p | -1.06903 | 401.21255 | 842.85026 | MGG_06794 | 1.84819 | 2.6004722 | 0 | puromycin resistance protein pur8 (2276 nt) | 4 | 2162-2181 | target 5' UAUGCAG-UGGCACCUCUGGC 3' | :::::: : :: :::::::.:: | query 3' AUACGUCAACGGUGGAGAUCG 5' |
| SI48_SI24 | osa-miR5160 | -1.40298 | 21.314417 | 58.009937 | MGG_01973 | 4.12678 | 16.469658 | 0 | conserved hypothetical protein (3625 nt) | 4 | 1977-1999 | target 5' UAGAGACCCUACCAUCGAUCUCG 3' | .:::.: ::::::::::::: | query 3' GUCUUU-AUAUGGUAGCUAGAGC 5' |
| SI48_SI24 | osa-miR5161 | -2.27055 | 2.5075784 | 15.924297 | MGG_02949 | 4.92941 | 29.472019 | 0 | conserved hypothetical protein (3524 nt) | 4 | 461-479 | target 5' UAUGGU-CCUUUGAUCCAGG 3' | :::. : :::.::::::::. | query 3' AUAUGAGGGAGACUAGGUCU 5' |
| SI48_SI24 | osa-miR5161 | -2.27055 | 2.5075784 | 15.924297 | MGG_02732 | 1.85592 | 6.9345926 | 1.1919868 | conserved hypothetical protein (1700 nt) | 4 | 1168-1186 | target 5' AAGACU-CCUCUGAUCCAGC 3' | : ::: ::::::::::: | query 3' AUAUGAGGGAGACUAGGUCU 5' |
| SI48_SI24 | osa-miR5508 | -1.81189 | 21.314417 | 77.346583 | MGG_14752 | 4.19664 | 17.336482 | 0 | conserved hypothetical protein (3554 nt) | 4 | 846-866 | target 5' CCACGGAGGAUCAGCCAUCUU 3' | ::::. .::::::::::: | query 3' GGUGUGGUCUAGUCGGUAGAU 5' |
| SI48_SI24 | osa-miR5512 | -1.07512 | 35.106098 | 75.071684 | MGG_03627 | 4.55688 | 22.537426 | 0 | conserved hypothetical protein (1204 nt) | 4 | 1088-1109 | target 5' UUGUAUCAUUUACUAUAUCCUA 3' | :: :: : : ::::.:::::::: | query 3' AAAAUCGU-AAUGGUAUAGGAU 5' |
| SI48_SI24 | osa-miR811d.1 | -1.70872 | 85.257666 | 280.95009 | MGG_03290 | 2.19391 | 19.936954 | 3.5759605 | N-(5-amino-5-carboxypentanoyl)-L-cysteinyl-D- | 3.5 | 2114-2134 | target 5' UGGGCAUAUGGGAUCCGUCCA 3' | :::.:: : .:.:::::::::: | query 3' ACCUGUAGGCUCUAGGCAGGU 5' |
| SI48_SI24 | osa-miR820a | -1.64741 | 50.151568 | 159.24297 | MGG_11519 | 1.5279 | 19.07013 | 5.9599342 | alcohol dehydrogenase (1729 nt) | 3 | 745-765 | target 5' CUGGUCCUUCCGCGAGGCCGC 3' | :::::: : :::.:::::::: | query 3' GACCAGGUAGGUGCUCCGGCU 5' |
| SI48_SI24 | osa-miR820a | -1.64741 | 50.151568 | 159.24297 | MGG_09559 | 3.61691 | 11.268713 | 0 | autophagy-related protein 9 (3015 nt) | 3 | 410-430 | target 5' CUGGACCAUCCACGAGGCAGA 3' | :::: : ::::::::::: :: | query 3' GACCAGGUAGGUGCUCCGGCU 5' |
| SI48_SI24 | osa-miR820a | -1.64741 | 50.151568 | 159.24297 | MGG_04090 | 5.44209 | 42.47438 | 0 | SNARE protein [Neurospora crassa OR74A]) (234 | 4 | 654-674 | target 5' CAGGUCCCUCAACGAGGCCGA 3' | : :::: : :: :::::::::: | query 3' GACCAGGUAGGUGCUCCGGCU 5' |
| SI48_SI24 | osa-miR820a | -1.64741 | 50.151568 | 159.24297 | MGG_08117 | 4.12678 | 16.469658 | 0 | DRAP deaminase (1625 nt) | 4 | 618-637 | target 5' CAGGUCC-UCCACGAGGACGA 3' | : :::: : ::::::::: ::: | query 3' GACCAGGUAGGUGCUCCGGCU 5' |
| SI48_SI24 | osa-miR820a | -1.64741 | 50.151568 | 159.24297 | MGG_02417 | 2.60665 | 26.871546 | 3.5759605 | ATP-dependent rRNA helicase spb-4 (2165 nt) | 4 | 714-733 | target 5' CUGGUCC-UCGACGAGGCUGA 3' | :::::: : :: :::::::.:: | query 3' GACCAGGUAGGUGCUCCGGCU 5' |
| SI48_SI24 | osa-miR820a | -1.64741 | 50.151568 | 159.24297 | MGG_01706 | 3.74003 | 171.63117 | 11.919868 | hypothetical protein (773 nt) | 4 | 352-373 | target 5' CUGCUUCGUCCGACGAGGCCGA 3' | ::: :.: .::: :::::::::: | query 3' GACCAGGUAGG-UGCUCCGGCU 5' |
| SI48_SI24 | osa-miR820b | -1.64741 | 50.151568 | 159.24297 | MGG_11519 | 1.5279 | 19.07013 | 5.9599342 | alcohol dehydrogenase (1729 nt) | 3 | 745-765 | target 5' CUGGUCCUUCCGCGAGGCCGC 3' | :::::: : :::.:::::::: | query 3' GACCAGGUAGGUGCUCCGGCU 5' |
| SI48_SI24 | osa-miR820b | -1.64741 | 50.151568 | 159.24297 | MGG_09559 | 3.61691 | 11.268713 | 0 | autophagy-related protein 9 (3015 nt) | 3 | 410-430 | target 5' CUGGACCAUCCACGAGGCAGA 3' | :::: : ::::::::::: :: | query 3' GACCAGGUAGGUGCUCCGGCU 5' |
| SI48_SI24 | osa-miR820b | -1.64741 | 50.151568 | 159.24297 | MGG_04090 | 5.44209 | 42.47438 | 0 | SNARE protein [Neurospora crassa OR74A]) (234 | 4 | 654-674 | target 5' CAGGUCCCUCAACGAGGCCGA 3' | : :::: : :: :::::::::: | query 3' GACCAGGUAGGUGCUCCGGCU 5' |
| SI48_SI24 | osa-miR820b | -1.64741 | 50.151568 | 159.24297 | MGG_08117 | 4.12678 | 16.469658 | 0 | DRAP deaminase (1625 nt) | 4 | 618-637 | target 5' CAGGUCC-UCCACGAGGACGA 3' | : :::: : ::::::::: ::: | query 3' GACCAGGUAGGUGCUCCGGCU 5' |
| SI48_SI24 | osa-miR820b | -1.64741 | 50.151568 | 159.24297 | MGG_02417 | 2.60665 | 26.871546 | 3.5759605 | ATP-dependent rRNA helicase spb-4 (2165 nt) | 4 | 714-733 | target 5' CUGGUCC-UCGACGAGGCUGA 3' | :::::: : :: :::::::.:: | query 3' GACCAGGUAGGUGCUCCGGCU 5' |

| | | | | | | | | | | | | | | |
|---|---|---|---|---|---|---|---|---|---|---|---|---|---|---|
| SI48_SI24 | osa-miR820b | -1.64741 | 50.151568 | 159.24297 | MGG_01706 | 3.74003 | 171.63117 | 11.919868 | hypothetical protein (773 nt) | 4 | 352-373 | target 5' CUGCUUCGUCCGACGAGGCCGA 3' | ::: :.: .::: ::::::::: | query 3' GACCAGGUAGG-UGCUCCGGCU 5' |
| SI48_SI24 | osa-miR820c | -1.64741 | 50.151568 | 159.24297 | MGG_11519 | 1.5279 | 19.07013 | 5.9599342 | alcohol dehydrogenase (1729 nt) | 3 | 745-765 | target 5' CUGGUCCUUCCGCGAGGCCGC 3' | :::::: : :::.:::::::: | query 3' GACCAGGUAGGUGCUCCGGCU 5' |
| SI48_SI24 | osa-miR820c | -1.64741 | 50.151568 | 159.24297 | MGG_09559 | 3.61691 | 11.268713 | 0 | autophagy-related protein 9 (3015 nt) | 3 | 410-430 | target 5' CUGGACCAUCCACGAGGCAGA 3' | :::: : ::::::::::: :: | query 3' GACCAGGUAGGUGCUCCGGCU 5' |
| SI48_SI24 | osa-miR820c | -1.64741 | 50.151568 | 159.24297 | MGG_04090 | 5.44209 | 42.47438 | 0 | SNARE protein [Neurospora crassa OR74A]) (234 | 4 | 654-674 | target 5' CAGGUCCCUCAACGAGGCCGA 3' | : :::: : :: ::::::::: | query 3' GACCAGGUAGGUGCUCCGGCU 5' |
| SI48_SI24 | osa-miR820c | -1.64741 | 50.151568 | 159.24297 | MGG_08117 | 4.12678 | 16.469658 | 0 | DRAP deaminase (1625 nt) | 4 | 618-637 | target 5' CAGGUCC-UCCACGAGGACGA 3' | : :::: : :::::::: ::: | query 3' GACCAGGUAGGUGCUCCGGCU 5' |
| SI48_SI24 | osa-miR820c | -1.64741 | 50.151568 | 159.24297 | MGG_02417 | 2.60665 | 26.871546 | 3.5759605 | ATP-dependent rRNA helicase spb-4 (2165 nt) | 4 | 714-733 | target 5' CUGGUCC-UCGACGAGGCUGA 3' | :::::: : :: :::::::.:: | query 3' GACCAGGUAGGUGCUCCGGCU 5' |
| SI48_SI24 | osa-miR820c | -1.64741 | 50.151568 | 159.24297 | MGG_01706 | 3.74003 | 171.63117 | 11.919868 | hypothetical protein (773 nt) | 4 | 352-373 | target 5' CUGCUUCGUCCGACGAGGCCGA 3' | ::: :.: .::: ::::::::: | query 3' GACCAGGUAGG-UGCUCCGGCU 5' |

| **Table S8. A list of anti-correlated pairs between rice DE miRNAs and DE rice genes** | | | | | | | | | | | | | | |
|---|---|---|---|---|---|---|---|---|---|---|---|---|---|---|
| **comparison** | **miRNA ID** | **miRNA FC (log2 of exp1 vs exp2)** | **exp1** | **exp2** | **gene ID (fungus)** | **gene FC** | **exp1** | **exp2** | **gene description** | **target score** | **range** | **alignment info** | | |
| RC00_SC00 | osa-miR156a | 1.15423 | 250602.3 | 112596.6 | BGIOSGA029541 | -1.24754 | 9.625154 | 24.22802 | Putative uncharacterized protein [Source:UniProtKB/TrEMBL;Acc:B8BD46] | 1 | 578-597 | target 5' GUGCUCUCUCUCUUCUGUCA 3' | :::::: ::::::::::::: | query 3' CACGAGUGAGAGAAGACAGU 5' |
| RC00_SC00 | osa-miR156a | 1.15423 | 250602.3 | 112596.6 | BGIOSGA007499 | -1.23357 | 13.47522 | 33.03821 | Squamosa promoter-binding-like protein 3 [Source:UniProtKB/Swiss-Prot;Acc:A2X0Q6] | 2 | 1148-1167 | target 5' AUGCUCUCUCUCUUCUGUCA 3' | :::: : ::::::::::::: | query 3' CACGAGUGAGAGAAGACAGU 5' |
| RC00_SC00 | osa-miR156a | 1.15423 | 250602.3 | 112596.6 | BGIOSGA036253 | -2.28092 | 1.925031 | 13.21528 | Putative uncharacterized protein [Source:UniProtKB/TrEMBL;Acc:A2ZK56] | 4 | 232-250 | target 5' GUGCUCACUCUC-UCUGGCA 3' | :::::: :::::: :::: :: | query 3' CACGAGUGAGAGAAGACAGU 5' |
| RC00_SC00 | osa-miR156b | 1.15423 | 250602.3 | 112596.6 | BGIOSGA029541 | -1.24754 | 9.625154 | 24.22802 | Putative uncharacterized protein [Source:UniProtKB/TrEMBL;Acc:B8BD46] | 1 | 578-597 | target 5' GUGCUCUCUCUCUUCUGUCA 3' | :::::: ::::::::::::: | query 3' CACGAGUGAGAGAAGACAGU 5' |
| RC00_SC00 | osa-miR156b | 1.15423 | 250602.3 | 112596.6 | BGIOSGA007499 | -1.23357 | 13.47522 | 33.03821 | Squamosa promoter-binding-like protein 3 [Source:UniProtKB/Swiss-Prot;Acc:A2X0Q6] | 2 | 1148-1167 | target 5' AUGCUCUCUCUCUUCUGUCA 3' | :::: : ::::::::::::: | query 3' CACGAGUGAGAGAAGACAGU 5' |
| RC00_SC00 | osa-miR156b | 1.15423 | 250602.3 | 112596.6 | BGIOSGA036253 | -2.28092 | 1.925031 | 13.21528 | Putative uncharacterized protein [Source:UniProtKB/TrEMBL;Acc:A2ZK56] | 4 | 232-250 | target 5' GUGCUCACUCUC-UCUGGCA 3' | :::::: :::::: :::: :: | query 3' CACGAGUGAGAGAAGACAGU 5' |
| RC00_SC00 | osa-miR156c | 1.15423 | 250602.3 | 112596.6 | BGIOSGA029541 | -1.24754 | 9.625154 | 24.22802 | Putative uncharacterized protein [Source:UniProtKB/TrEMBL;Acc:B8BD46] | 1 | 578-597 | target 5' GUGCUCUCUCUCUUCUGUCA 3' | :::::: ::::::::::::: | query 3' CACGAGUGAGAGAAGACAGU 5' |
| RC00_SC00 | osa-miR156c | 1.15423 | 250602.3 | 112596.6 | BGIOSGA007499 | -1.23357 | 13.47522 | 33.03821 | Squamosa promoter-binding-like protein 3 [Source:UniProtKB/Swiss-Prot;Acc:A2X0Q6] | 2 | 1148-1167 | target 5' AUGCUCUCUCUCUUCUGUCA 3' | :::: : ::::::::::::: | query 3' CACGAGUGAGAGAAGACAGU 5' |
| RC00_SC00 | osa-miR156c | 1.15423 | 250602.3 | 112596.6 | BGIOSGA036253 | -2.28092 | 1.925031 | 13.21528 | Putative uncharacterized protein [Source:UniProtKB/TrEMBL;Acc:A2ZK56] | 4 | 232-250 | target 5' GUGCUCACUCUC-UCUGGCA 3' | :::::: :::::: :::: :: | query 3' CACGAGUGAGAGAAGACAGU 5' |
| RC00_SC00 | osa-miR156d | 1.15423 | 250602.3 | 112596.6 | BGIOSGA029541 | -1.24754 | 9.625154 | 24.22802 | Putative uncharacterized protein [Source:UniProtKB/TrEMBL;Acc:B8BD46] | 1 | 578-597 | target 5' GUGCUCUCUCUCUUCUGUCA 3' | :::::: ::::::::::::: | query 3' CACGAGUGAGAGAAGACAGU 5' |
| RC00_SC00 | osa-miR156d | 1.15423 | 250602.3 | 112596.6 | BGIOSGA007499 | -1.23357 | 13.47522 | 33.03821 | Squamosa promoter-binding-like protein 3 [Source:UniProtKB/Swiss-Prot;Acc:A2X0Q6] | 2 | 1148-1167 | target 5' AUGCUCUCUCUCUUCUGUCA 3' | :::: : ::::::::::::: | query 3' CACGAGUGAGAGAAGACAGU 5' |
| RC00_SC00 | osa-miR156d | 1.15423 | 250602.3 | 112596.6 | BGIOSGA036253 | -2.28092 | 1.925031 | 13.21528 | Putative uncharacterized protein | 4 | 232-250 | target 5' GUGCUCACUCUC-U | :::::: :::::: :::: :: | query 3' CACGAGUGAGAGAAGA |

| | | | | | | | | | | | | | | |
|---|---|---|---|---|---|---|---|---|---|---|---|---|---|---|
| | | | | | | | | | [Source:UniProtKB/TrEMBL;Acc:A2ZK56] | | | CUGGCA 3' | | CAGU 5' |
| RC00_SC00 | osa-miR156e | 1.15423 | 250602.3 | 112596.6 | BGIOSGA029541 | -1.24754 | 9.625154 | 24.22802 | Putative uncharacterized protein [Source:UniProtKB/TrEMBL;Acc:B8BD46] | 1 | 578-597 | target 5' GUGCUCUCUCUCUCUGUCA 3' | :::::: :::::::::::: | query 3' CACGAGUGAGAGAAGACAGU 5' |
| RC00_SC00 | osa-miR156e | 1.15423 | 250602.3 | 112596.6 | BGIOSGA007499 | -1.23357 | 13.47522 | 33.03821 | Squamosa promoter-binding-like protein 3 [Source:UniProtKB/Swiss-Prot;Acc:A2X0Q6] | 2 | 1148-1167 | target 5' AUGCUCUCUCUCUCUGUCA 3' | :::: : :::::::::::: | query 3' CACGAGUGAGAGAAGACAGU 5' |
| RC00_SC00 | osa-miR156e | 1.15423 | 250602.3 | 112596.6 | BGIOSGA036253 | -2.28092 | 1.925031 | 13.21528 | Putative uncharacterized protein [Source:UniProtKB/TrEMBL;Acc:A2ZK56] | 4 | 232-250 | target 5' GUGCUCACUCUC-UCUGGCA 3' | :::::: :::::: :::: :: | query 3' CACGAGUGAGAGAAGACAGU 5' |
| RC00_SC00 | osa-miR156f | 1.15423 | 250602.3 | 112596.6 | BGIOSGA029541 | -1.24754 | 9.625154 | 24.22802 | Putative uncharacterized protein [Source:UniProtKB/TrEMBL;Acc:B8BD46] | 1 | 578-597 | target 5' GUGCUCUCUCUCUCUGUCA 3' | :::::: :::::::::::: | query 3' CACGAGUGAGAGAAGACAGU 5' |
| RC00_SC00 | osa-miR156f | 1.15423 | 250602.3 | 112596.6 | BGIOSGA007499 | -1.23357 | 13.47522 | 33.03821 | Squamosa promoter-binding-like protein 3 [Source:UniProtKB/Swiss-Prot;Acc:A2X0Q6] | 2 | 1148-1167 | target 5' AUGCUCUCUCUCUCUGUCA 3' | :::: : :::::::::::: | query 3' CACGAGUGAGAGAAGACAGU 5' |
| RC00_SC00 | osa-miR156f | 1.15423 | 250602.3 | 112596.6 | BGIOSGA036253 | -2.28092 | 1.925031 | 13.21528 | Putative uncharacterized protein [Source:UniProtKB/TrEMBL;Acc:A2ZK56] | 4 | 232-250 | target 5' GUGCUCACUCUC-UCUGGCA 3' | :::::: :::::: :::: :: | query 3' CACGAGUGAGAGAAGACAGU 5' |
| RC00_SC00 | osa-miR156g | 1.15423 | 250602.3 | 112596.6 | BGIOSGA029541 | -1.24754 | 9.625154 | 24.22802 | Putative uncharacterized protein [Source:UniProtKB/TrEMBL;Acc:B8BD46] | 1 | 578-597 | target 5' GUGCUCUCUCUCUCUGUCA 3' | :::::: :::::::::::: | query 3' CACGAGUGAGAGAAGACAGU 5' |
| RC00_SC00 | osa-miR156g | 1.15423 | 250602.3 | 112596.6 | BGIOSGA007499 | -1.23357 | 13.47522 | 33.03821 | Squamosa promoter-binding-like protein 3 [Source:UniProtKB/Swiss-Prot;Acc:A2X0Q6] | 2 | 1148-1167 | target 5' AUGCUCUCUCUCUCUGUCA 3' | :::: : :::::::::::: | query 3' CACGAGUGAGAGAAGACAGU 5' |
| RC00_SC00 | osa-miR156g | 1.15423 | 250602.3 | 112596.6 | BGIOSGA036253 | -2.28092 | 1.925031 | 13.21528 | Putative uncharacterized protein [Source:UniProtKB/TrEMBL;Acc:A2ZK56] | 4 | 232-250 | target 5' GUGCUCACUCUC-UCUGGCA 3' | :::::: :::::: :::: :: | query 3' CACGAGUGAGAGAAGACAGU 5' |
| RC00_SC00 | osa-miR156h | 1.15423 | 250602.3 | 112596.6 | BGIOSGA029541 | -1.24754 | 9.625154 | 24.22802 | Putative uncharacterized protein [Source:UniProtKB/TrEMBL;Acc:B8BD46] | 1 | 578-597 | target 5' GUGCUCUCUCUCUCUGUCA 3' | :::::: :::::::::::: | query 3' CACGAGUGAGAGAAGACAGU 5' |
| RC00_SC00 | osa-miR156h | 1.15423 | 250602.3 | 112596.6 | BGIOSGA007499 | -1.23357 | 13.47522 | 33.03821 | Squamosa promoter-binding-like protein 3 [Source:UniProtKB/Swiss-Prot;Acc:A2X0Q6] | 2 | 1148-1167 | target 5' AUGCUCUCUCUCUCUGUCA 3' | :::: : :::::::::::: | query 3' CACGAGUGAGAGAAGACAGU 5' |
| RC00_SC00 | osa-miR156h | 1.15423 | 250602.3 | 112596.6 | BGIOSGA036253 | -2.28092 | 1.925031 | 13.21528 | Putative uncharacterized protein [Source:UniProtKB/TrEMBL;Acc:A2ZK56] | 4 | 232-250 | target 5' GUGCUCACUCUC-UCUGGCA 3' | :::::: :::::: :::: :: | query 3' CACGAGUGAGAGAAGACAGU 5' |
| RC00_SC00 | osa-miR156i | 1.15423 | 250602.3 | 112596.6 | BGIOSGA029541 | -1.24754 | 9.625154 | 24.22802 | Putative uncharacterized protein [Source:UniProtKB/TrEM | 1 | 578-597 | target 5' GUGCUCUCUCUCUCUGUCA 3' | :::::: :::::::::::: | query 3' CACGAGUGAGAGAAGACAGU 5' |

| | | | | | | | | | | | | | |
|---|---|---|---|---|---|---|---|---|---|---|---|---|---|
| | | | | | | | | | BL;Acc:B8BD46] | | | | |
| RC00_ SC00 | osa-miR1 56i | 1.15423 | 2506 02.3 | 1125 96.6 | BGIOSG A007499 | -1.2 3357 | 13.4 7522 | 33.0 3821 | Squamosa promoter-binding-like protein 3 [Source:UniProtKB/Swiss-Prot;Acc:A2X0Q6] | 2 | 1148-1167 | target 5' AUGCUCUCUCUCU UCUGUCA 3' | :::: : ::::::::::::: | query 3' CACGAGUGAGAGAAGA CAGU 5' |
| RC00_ SC00 | osa-miR1 56i | 1.15423 | 2506 02.3 | 1125 96.6 | BGIOSG A036253 | -2.2 8092 | 1.92 5031 | 13.2 1528 | Putative uncharacterized protein [Source:UniProtKB/TrEMBL;Acc:A2ZK56] | 4 | 232-250 | target 5' GUGCUCACUCUC-U CUGGCA 3' | :::::: :::::: :::: :: | query 3' CACGAGUGAGAGAAGA CAGU 5' |
| RC00_ SC00 | osa-miR1 56j | 1.15423 | 2506 02.3 | 1125 96.6 | BGIOSG A029541 | -1.2 4754 | 9.62 5154 | 24.2 2802 | Putative uncharacterized protein [Source:UniProtKB/TrEMBL;Acc:B8BD46] | 1 | 578-597 | target 5' GUGCUCUCUCUCU UCUGUCA 3' | :::::: ::::::::::::: | query 3' CACGAGUGAGAGAAGA CAGU 5' |
| RC00_ SC00 | osa-miR1 56j | 1.15423 | 2506 02.3 | 1125 96.6 | BGIOSG A007499 | -1.2 3357 | 13.4 7522 | 33.0 3821 | Squamosa promoter-binding-like protein 3 [Source:UniProtKB/Swiss-Prot;Acc:A2X0Q6] | 2 | 1148-1167 | target 5' AUGCUCUCUCUCU UCUGUCA 3' | :::: : ::::::::::::: | query 3' CACGAGUGAGAGAAGA CAGU 5' |
| RC00_ SC00 | osa-miR1 56j | 1.15423 | 2506 02.3 | 1125 96.6 | BGIOSG A036253 | -2.2 8092 | 1.92 5031 | 13.2 1528 | Putative uncharacterized protein [Source:UniProtKB/TrEMBL;Acc:A2ZK56] | 4 | 232-250 | target 5' GUGCUCACUCUC-U CUGGCA 3' | :::::: :::::: :::: :: | query 3' CACGAGUGAGAGAAGA CAGU 5' |
| RC00_ SC00 | osa-miR1 56k | 1.15807 | 2474 84.8 | 1109 00.3 | BGIOSG A029541 | -1.2 4754 | 9.62 5154 | 24.2 2802 | Putative uncharacterized protein [Source:UniProtKB/TrEMBL;Acc:B8BD46] | 0 | 577-597 | target 5' UGUGCUCUCUCUC UUCUGUCA 3' | :::::: :::::::::::::: | query 3' ACACGAGAGAGAGAAG ACAGU 5' |
| RC00_ SC00 | osa-miR1 56k | 1.15807 | 2474 84.8 | 1109 00.3 | BGIOSG A007499 | -1.2 3357 | 13.4 7522 | 33.0 3821 | Squamosa promoter-binding-like protein 3 [Source:UniProtKB/Swiss-Prot;Acc:A2X0Q6] | 2 | 1147-1167 | target 5' CAUGCUCUCUCUC UUCUGUCA 3' | :: :::::::::::::::: | query 3' ACACGAGAGAGAGAAG ACAGU 5' |
| RC00_ SC00 | osa-miR1 59a.2 | -1.6156 7 | 15.7 6225 | 50.3 6861 | BGIOSG A011584 | 2.27 8 | 3.85 0062 | 0 | Putative uncharacterized protein [Source:UniProtKB/TrEMBL;Acc:A2XBP8] | 4 | 1087-1107 | target 5' AGCUGCUCCGGGG GCAUGCAA 3' | :: : :::: :::::::::: | query 3' ACGUCGAGGACCCCGU ACGUU 5' |
| RC00_ SC00 | osa-miR1 60a | -1.2708 8 | 379. 1697 | 916. 3837 | BGIOSG A014036 | 1.54 845 | 1.92 5031 | 0 | Putative uncharacterized protein [Source:UniProtKB/TrEMBL;Acc:A2XZ93] | 2 | 1185-1205 | target 5' UGACAUUCAGGGA GCCAGGCA 3' | :: ::: ::::::::::::: | query 3' ACCGUAUGUCCCUCGG UCCGU 5' |
| RC00_ SC00 | osa-miR1 60b | -1.2708 8 | 379. 1697 | 916. 3837 | BGIOSG A014036 | 1.54 845 | 1.92 5031 | 0 | Putative uncharacterized protein [Source:UniProtKB/TrEMBL;Acc:A2XZ93] | 2 | 1185-1205 | target 5' UGACAUUCAGGGA GCCAGGCA 3' | :: ::: ::::::::::::: | query 3' ACCGUAUGUCCCUCGG UCCGU 5' |
| RC00_ SC00 | osa-miR1 60c | -1.2708 8 | 379. 1697 | 916. 3837 | BGIOSG A014036 | 1.54 845 | 1.92 5031 | 0 | Putative uncharacterized protein [Source:UniProtKB/TrEMBL;Acc:A2XZ93] | 2 | 1185-1205 | target 5' UGACAUUCAGGGA GCCAGGCA 3' | :: ::: ::::::::::::: | query 3' ACCGUAUGUCCCUCGG UCCGU 5' |
| RC00_ SC00 | osa-miR1 60d | -1.2708 8 | 379. 1697 | 916. 3837 | BGIOSG A014036 | 1.54 845 | 1.92 5031 | 0 | Putative uncharacterized protein [Source:UniProtKB/TrEMBL;Acc:A2XZ93] | 2 | 1185-1205 | target 5' UGACAUUCAGGGA GCCAGGCA 3' | :: ::: ::::::::::::: | query 3' ACCGUAUGUCCCUCGG UCCGU 5' |
| RC00_ SC00 | osa-miR1 60e | -1.2473 3 | 309. 1152 | 735. 2192 | BGIOSG A014036 | 1.54 845 | 1.92 5031 | 0 | Putative uncharacterized protein [Source:UniProtKB/TrEMBL;Acc:A2XZ93] | 2.5 | 1185-1205 | target 5' UGACAUUCAGGGA GCCAGGCA 3' | .: ::: ::::::::::::: | query 3' GCCGUAUGUCCCUCGG UCCGU 5' |

| | | | | | | | | | | | | | | |
|---|---|---|---|---|---|---|---|---|---|---|---|---|---|---|
| RC00_SC00 | osa-miR160f | -1.3173 | 292.4773 | 730.3448 | BGIOSGA014036 | 1.54845 | 1.925031 | 0 | Putative uncharacterized protein [Source:UniProtKB/TrEMBL;Acc:A2XZ93] | 1 | 1185-1205 | target 5' UGACAUUCAGGGAGCCAGGCA 3' | :: ::: :::::::::::::: | query 3' ACCGUAAGUCCCUCGGUCCGU 5' |
| RC00_SC00 | osa-miR166a | -1.22 | 85782.54 | 199828.5 | BGIOSGA002254 | 1.54845 | 1.925031 | 0 | Putative uncharacterized protein [Source:UniProtKB/TrEMBL;Acc:B8ADK5] | 3.5 | 565-586 | target 5' GGGGAUCUGAAGUCUGGUCCGG 3' | ::::: :::::.::::::::. | query 3' CCCCU-UACUUCGGACCAGGCU 5' |
| RC00_SC00 | osa-miR166b | -1.22 | 85782.54 | 199828.5 | BGIOSGA002254 | 1.54845 | 1.925031 | 0 | Putative uncharacterized protein [Source:UniProtKB/TrEMBL;Acc:B8ADK5] | 3.5 | 565-586 | target 5' GGGGAUCUGAAGUCUGGUCCGG 3' | ::::: :::::.::::::::. | query 3' CCCCU-UACUUCGGACCAGGCU 5' |
| RC00_SC00 | osa-miR166c | -1.22 | 85782.54 | 199828.5 | BGIOSGA002254 | 1.54845 | 1.925031 | 0 | Putative uncharacterized protein [Source:UniProtKB/TrEMBL;Acc:B8ADK5] | 3.5 | 565-586 | target 5' GGGGAUCUGAAGUCUGGUCCGG 3' | ::::: :::::.::::::::. | query 3' CCCCU-UACUUCGGACCAGGCU 5' |
| RC00_SC00 | osa-miR166d | -1.22 | 85782.54 | 199828.5 | BGIOSGA002254 | 1.54845 | 1.925031 | 0 | Putative uncharacterized protein [Source:UniProtKB/TrEMBL;Acc:B8ADK5] | 3.5 | 565-586 | target 5' GGGGAUCUGAAGUCUGGUCCGG 3' | ::::: :::::.::::::::. | query 3' CCCCU-UACUUCGGACCAGGCU 5' |
| RC00_SC00 | osa-miR166f | -1.22 | 85782.54 | 199828.5 | BGIOSGA002254 | 1.54845 | 1.925031 | 0 | Putative uncharacterized protein [Source:UniProtKB/TrEMBL;Acc:B8ADK5] | 3.5 | 565-586 | target 5' GGGGAUCUGAAGUCUGGUCCGG 3' | ::::: :::::.::::::::. | query 3' CCCCU-UACUUCGGACCAGGCU 5' |
| RC00_SC00 | osa-miR166g | -1.2105 | 82255.3 | 190355.2 | BGIOSGA002254 | 1.54845 | 1.925031 | 0 | Putative uncharacterized protein [Source:UniProtKB/TrEMBL;Acc:B8ADK5] | 4 | 565-586 | target 5' GGGGAUCUGAAGUCUGGUCCGG 3' | :.::: :::::.::::::::. | query 3' CUCCU-UACUUCGGACCAGGCU 5' |
| RC00_SC00 | osa-miR166h | -1.2105 | 82255.3 | 190355.2 | BGIOSGA002254 | 1.54845 | 1.925031 | 0 | Putative uncharacterized protein [Source:UniProtKB/TrEMBL;Acc:B8ADK5] | 4 | 565-586 | target 5' GGGGAUCUGAAGUCUGGUCCGG 3' | :.::: :::::.::::::::. | query 3' CUCCU-UACUUCGGACCAGGCU 5' |
| RC00_SC00 | osa-miR166i | -1.17997 | 19185.29 | 43469.73 | BGIOSGA017414 | 3.12104 | 7.700124 | 0 | Putative uncharacterized protein [Source:UniProtKB/TrEMBL;Acc:A2XZA6] | 4 | 3637-3658 | target 5' AAGGAAGUGAAGCUUGAUCUGA 3' | :::: : ::::::.:::::.:: | query 3' CUCCUU-ACUUCGGACUAGGCU 5' |
| RC00_SC00 | osa-miR166j | -1.17997 | 19185.29 | 43469.73 | BGIOSGA017414 | 3.12104 | 7.700124 | 0 | Putative uncharacterized protein [Source:UniProtKB/TrEMBL;Acc:A2XZA6] | 4 | 3637-3658 | target 5' AAGGAAGUGAAGCUUGAUCUGA 3' | :::: : ::::::.:::::.:: | query 3' CUCCUU-ACUUCGGACUAGGCU 5' |
| RC00_SC00 | osa-miR166n | -1.22 | 85782.54 | 199828.5 | BGIOSGA002254 | 1.54845 | 1.925031 | 0 | Putative uncharacterized protein [Source:UniProtKB/TrEMBL;Acc:B8ADK5] | 3.5 | 565-586 | target 5' GGGGAUCUGAAGUCUGGUCCGG 3' | ::::: :::::.::::::::. | query 3' CCCCU-UACUUCGGACCAGGCU 5' |
| RC00_SC00 | osa-miR2864.2 | 6.26092 | 3315.327 | 42.24464 | BGIOSGA007922 | -1.67922 | 0 | 2.202547 | Putative uncharacterized protein [Source:UniProtKB/TrEMBL;Acc:A2X370] | 2 | 565-586 | target 5' UACCUAUACAAUGACAAAACAA 3' | :::::: ::::::: :::::::: | query 3' AUGGAUAUGUUAC-GUUUUGUU 5' |
| RC00_SC00 | osa-miR5161 | 1.32711 | 21.89201 | 8.123969 | BGIOSGA036828 | -1.14927 | 17.32528 | 39.64585 | Putative uncharacterized protein [Source:UniProtKB/TrEMBL;Acc:B8BLQ7] | 4 | 1690-1709 | target 5' CAUACUCCCUGUGGUCCAGA 3' | :::: ::::: ::.:::::: | query 3' AUAUGAGGGAGACUAGGUCU 5' |
| RC00_SC00 | osa-miR529b | 2.56529 | 67.4274 | 10.56116 | BGIOSGA029541 | -1.24754 | 9.625154 | 24.22802 | Putative uncharacterized protein [Source:UniProtKB/TrEMBL;Acc:B8BD46] | 1.5 | 573-593 | target 5' CAGCUGUGCUCUCUCUCUUCU 3' | :::: ::.:::::::::::: | query 3' UUCGACAUGAGAGAGAGAAGA 5' |

| | | | | | | | | | | | | | | |
|---|---|---|---|---|---|---|---|---|---|---|---|---|---|---|
| RC00_SC00 | osa-miR529b | 2.56529 | 67.4274 | 10.56116 | BGIOSGA007499 | -1.23357 | 13.47522 | 33.03821 | Squamosa promoter-binding-like protein 3 [Source:UniProtKB/Swiss-Prot;Acc:A2X0Q6] | 3 | 1144-1163 | target 5'<br>GAGC-AUGCUCUCUCUCUUCU 3' | .:::<br>:.:::::::::::: | query 3'<br>UUCGACAUGAGAGAGAGAAGA 5' |
| RC00_SC00 | osa-miR811d.1 | -1.08502 | 44.65971 | 95.86283 | BGIOSGA005433 | 3.12104 | 7.700124 | 0 | Putative uncharacterized protein [Source:UniProtKB/TrEMBL;Acc:A2XAK6] | 4 | 55-74 | target 5'<br>UGGGUCUCCGAGAUCCG-CCA 3' | :::..<br>:::::::::: ::: | query 3'<br>ACCUGUAGGCUCUAGGCAGGU 5' |
| RC08_RC00 | osa-miR1846d-5p | 1.26919 | 16.18455 | 6.129764 | BGIOSGA016941 | -3.53628 | 5.225398 | 71.22614 | Putative uncharacterized protein [Source:UniProtKB/TrEMBL;Acc:B8AT39] | 4 | 268-289 | target 5'<br>GAGAAGCACCUGCUCGGUGGGA 3' | ::::<br>: :::::::::::: | query 3'<br>CUCUAGGCCGACGAGCCACCCU 5' |
| RC08_RC00 | osa-miR1861d | -1.22107 | 11.07364 | 27.1461 | BGIOSGA029503 | 3.56922 | 102.2685 | 7.700124 | Putative uncharacterized protein [Source:UniProtKB/TrEMBL;Acc:B8BD43] | 3.5 | 209-231 | target 5'<br>CUCCAGCUUCUGCCUCAAGGCCA 3' | :: :::<br>:.::::::::::.::: | query 3'<br>GA-GUCAAGGACGGAGUUCUGGU 5' |
| RC08_RC00 | osa-miR1861d | -1.22107 | 11.07364 | 27.1461 | BGIOSGA017432 | 4.8761 | 28.36645 | 0 | Putative uncharacterized protein [Source:UniProtKB/TrEMBL;Acc:B8AXI3] | 4 | 491-512 | target 5'<br>CCCAGUUUCUUCCUCAAGACCG 3' | : ::::<br>:.:: ::::::::::. | query 3'<br>GAGUCAAGGACGGAGUUCUGGU 5' |
| RC08_RC00 | osa-miR1861h | -1.07485 | 12.77728 | 28.02178 | BGIOSGA017432 | 4.8761 | 28.36645 | 0 | Putative uncharacterized protein [Source:UniProtKB/TrEMBL;Acc:B8AXI3] | 3.5 | 491-512 | target 5'<br>CCCAGUUUCUUCCUCAAGACCG 3' | : ::::<br>:.:: :::::::::: | query 3'<br>GAGUCAAGGACGGAGUUCUGGC 5' |
| RC08_RC00 | osa-miR1861j | -1.07485 | 12.77728 | 28.02178 | BGIOSGA017432 | 4.8761 | 28.36645 | 0 | Putative uncharacterized protein [Source:UniProtKB/TrEMBL;Acc:B8AXI3] | 3.5 | 491-512 | target 5'<br>CCCAGUUUCUUCCUCAAGACCG 3' | : ::::<br>:.:: :::::::::: | query 3'<br>GAGUCAAGGACGGAGUUCUGGC 5' |
| RC08_RC00 | osa-miR529b | -1.51159 | 22.9991 | 67.4274 | BGIOSGA020577 | 2.80304 | 59.71884 | 7.700124 | Squamosa promoter-binding-like protein 12 [Source:UniProtKB/Swiss-Prot;Acc:A2YGR5] | 3 | 1141-1160 | target 5'<br>CGGC-GUGCUCUCUCUCUUCU 3' | .::<br>::.::::::::::::: | query 3'<br>UUCGACAUGAGAGAGAGAAGA 5' |
| RC08_RC00 | osa-miR529b | -1.51159 | 22.9991 | 67.4274 | BGIOSGA007499 | 3.20922 | 132.8744 | 13.47522 | Squamosa promoter-binding-like protein 3 [Source:UniProtKB/Swiss-Prot;Acc:A2X0Q6] | 3 | 1144-1163 | target 5'<br>GAGC-AUGCUCUCUCUCUUCU 3' | .:::<br>:.:::::::::::: | query 3'<br>UUCGACAUGAGAGAGAGAAGA 5' |
| RC08_SC08 | osa-miR1320 | 1.94884 | 34.07274 | 8.084668 | BGIOSGA014361 | -1.49037 | 35.8313 | 102.4814 | Putative uncharacterized protein [Source:UniProtKB/TrEMBL;Acc:B8AU51] | 3.5 | 175-195 | target 5'<br>CCGCAUAAUUCCUCCGUUCCA 3' | : . :<br>::::::::::::::: | query 3'<br>GAUAUUUUAAGGAGGCAAGGU 5' |
| RC08_SC08 | osa-miR394 | -2.24877 | 40.03546 | 194.032 | BGIOSGA020470 | 1.1322 | 1030.15 | 469.431 | Putative uncharacterized protein [Source:UniProtKB/TrEMBL;Acc:B8AX92] | 3 | 1366-1385 | target 5'<br>GGAGUUGGACAGAAUGCAAA 3' | :::: :<br>:::::::::: :: | query 3'<br>CCUCCACCUGUCUUACGGUU 5' |
| RC08_SC08 | osa-miR394 | -2.24877 | 40.03546 | 194.032 | BGIOSGA025510 | 1.36506 | 46.2821 | 17.35572 | Putative uncharacterized protein [Source:UniProtKB/TrEMBL;Acc:B8B516] | 4 | 20-38 | target 5'<br>GGCGGCGGA-AGAAUGCCAA 3' | :: ::<br>::: :::::::::: | query 3'<br>CCUCCACCUGUCUUACGGUU 5' |
| RC08_SC08 | osa-miR529b | -1.09105 | 22.9991 | 50.12494 | BGIOSGA024017 | 1.12587 | 2.985942 | 0.826463 | Putative uncharacterized protein [Source:UniProtKB/TrEMBL;Acc:A2YN09] | 3.5 | 2994-3014 | target 5'<br>UAGCUUUGCUCUCUCUCUUCA 3' | ::::<br>:.:::::::::::: | query 3'<br>UUCGACAUGAGAGAGAGAAGA 5' |
| RI08_RC00 | osa-miR159a.1 | -1.15455 | 18151.83 | 40410.03 | BGIOSGA037152 | 2.28469 | 2714.654 | 556.3339 | Putative uncharacterized protein | 3.5 | 966-985 | target 5'<br>UAG-UCUCCCUUCA | .::<br>:::::::::::.:::: | query 3'<br>GUCUCGAGGGAAGUUA |

| | | | | | | | | | | | | | | |
|---|---|---|---|---|---|---|---|---|---|---|---|---|---|---|
| | | | | | | | | | [Source:UniProtKB/TrEMBL;Acc:B8BNM4] | | | AUUCAAA 3' | | GGUUU 5' |
| RI08_RC00 | osa-miR159a.1 | -1.15455 | 18151.83 | 40410.03 | BGIOSGA021944 | 6.24161 | 74.66777 | 0 | Putative uncharacterized protein [Source:UniProtKB/TrEMBL;Acc:A2Y963] | 3.5 | 424-443 | target 5' CA-AGUUACUUUCAAUCCAAA 3' | :: ::.<br>: :.::::::::::: | query 3' GUCUCGAGGGAAGUUAGGUUU 5' |
| RI08_RC00 | osa-miR159a.1 | -1.15455 | 18151.83 | 40410.03 | BGIOSGA004162 | 1.30675 | 368.1639 | 148.2274 | Putative uncharacterized protein [Source:UniProtKB/TrEMBL;Acc:B8A768] | 4 | 1653-1674 | target 5' CAGAGCUGCCUUACAAUCCAAC 3' | ::::::<br>: :::: :::::::: | query 3' GUCUCGAGGGAA-GUUAGGUUU 5' |
| RI08_RC00 | osa-miR159b | -1.15455 | 18151.83 | 40410.03 | BGIOSGA037152 | 2.28469 | 2714.654 | 556.3339 | Putative uncharacterized protein [Source:UniProtKB/TrEMBL;Acc:B8BNM4] | 3.5 | 966-985 | target 5' UAG-UCUCCCUUCAAUUCAAA 3' | .::<br>:::::::::::.:::: | query 3' GUCUCGAGGGAAGUUAGGUUU 5' |
| RI08_RC00 | osa-miR159b | -1.15455 | 18151.83 | 40410.03 | BGIOSGA021944 | 6.24161 | 74.66777 | 0 | Putative uncharacterized protein [Source:UniProtKB/TrEMBL;Acc:A2Y963] | 3.5 | 424-443 | target 5' CA-AGUUACUUUCAAUCCAAA 3' | :: ::.<br>: :.::::::::::: | query 3' GUCUCGAGGGAAGUUAGGUUU 5' |
| RI08_RC00 | osa-miR159b | -1.15455 | 18151.83 | 40410.03 | BGIOSGA004162 | 1.30675 | 368.1639 | 148.2274 | Putative uncharacterized protein [Source:UniProtKB/TrEMBL;Acc:B8A768] | 4 | 1653-1674 | target 5' CAGAGCUGCCUUACAAUCCAAC 3' | ::::::<br>: :::: :::::::: | query 3' GUCUCGAGGGAA-GUUAGGUUU 5' |
| RI08_RC00 | osa-miR159f | -1.19212 | 17146.84 | 39179.7 | BGIOSGA037152 | 2.28469 | 2714.654 | 556.3339 | Putative uncharacterized protein [Source:UniProtKB/TrEMBL;Acc:B8BNM4] | 4 | 966-985 | target 5' UAG-UCUCCCUUCAAUUCAAA 3' | :::<br>:::::::::::.::: | query 3' AUCUCGAGGGAAGUUAGGUUC 5' |
| RI08_RC08 | osa-miR1862f | -1.3978 | 298.7288 | 788.7838 | BGIOSGA005796 | 1.97973 | 338.5925 | 85.09934 | Putative uncharacterized protein [Source:UniProtKB/TrEMBL;Acc:B8AHB3] | 3 | 1138-1156 | target 5' UCAAAG-AAACCAACUUCAU 3' | .::::.<br>::::::::.:::: | query 3' GGUUUUAUUUGGUUGGAGUA 5' |
| RI08_RC08 | osa-miR1862g | -1.3978 | 298.7288 | 788.7838 | BGIOSGA005796 | 1.97973 | 338.5925 | 85.09934 | Putative uncharacterized protein [Source:UniProtKB/TrEMBL;Acc:B8AHB3] | 3 | 1138-1156 | target 5' UCAAAG-AAACCAACUUCAU 3' | .::::.<br>::::::::.:::: | query 3' GGUUUUAUUUGGUUGGAGUA 5' |
| RI08_RC08 | osa-miR396f | -1.02084 | 662.3572 | 1345.021 | BGIOSGA014417 | 1.21876 | 56.92493 | 23.88753 | Putative uncharacterized protein [Source:UniProtKB/TrEMBL;Acc:B8ATM9] | 3.5 | 451-472 | target 5' CGUUCAAGAAAGCCUGUGGAAG 3' | ::::<br>:::::::::::::: . | query 3' UCAAGUUCUUUCGGACACCUCU 5' |
| RI08_RC08 | osa-miR396f | -1.02084 | 662.3572 | 1345.021 | BGIOSGA008811 | 1.30951 | 1.47857 | 0 | Putative uncharacterized protein [Source:UniProtKB/TrEMBL;Acc:A2X8B1] | 3.5 | 508-529 | target 5' CGUUCAAGAAAGCCUGUGGAAG 3' | ::::<br>:::::::::::::: . | query 3' UCAAGUUCUUUCGGACACCUCU 5' |
| RI08_RC08 | osa-miR5083 | 1.05802 | 15.27048 | 6.814547 | BGIOSGA007106 | -1.15628 | 15.52498 | 35.8313 | Putative uncharacterized protein [Source:UniProtKB/TrEMBL;Acc:A2X127] | 3 | 1947-1965 | target 5' UGAUCAGAUAGUUGUA-UCU 3' | ::::::<br>::::.::::: ::: | query 3' ACUAGUCUAUUAACAUCAGA 5' |
| RI08_RC08 | osa-miR529b | 1.2558 | 56.30991 | 22.9991 | BGIOSGA020577 | -2.32842 | 11.08927 | 59.71884 | Squamosa promoter-binding-like protein 12 [Source:UniProtKB/Swiss-Prot;Acc:A2YGR5] | 3 | 1141-1160 | target 5' CGGC-GUGCUCUCUCUCUUCU 3' | .::<br>::.:::::::::::: | query 3' UUCGACAUGAGAGAGAGAAGA 5' |
| RI08_RC08 | osa-miR529b | 1.2558 | 56.30991 | 22.9991 | BGIOSGA031055 | -2.24258 | 0 | 3.732427 | Putative uncharacterized protein [Source:UniProtKB/TrEMBL;Acc:B8BDB6] | 3.5 | 1071-1091 | target 5' CGAUUGUGCUCUCUCUCUUCU 3' | . .:<br>::.:::::::::::: | query 3' UUCGACAUGAGAGAGAGAAGA 5' |
| RI24_RC00 | osa-miR1318 | 1.41378 | 237.3096 | 88.44374 | BGIOSGA013804 | -1.57716 | 2.560915 | 9.625154 | Putative uncharacterized protein | 1 | 45-64 | target 5' GUCGGUGUCGUCU | ::::::<br>:::.:::::::::: | query 3' CAGCCACAGUAGAGAG |

| | | | | | | | | | | | | | | |
|---|---|---|---|---|---|---|---|---|---|---|---|---|---|
| | | | | | | | | | [Source:UniProtKB/TrEMBL;Acc:A2XNA2] | | | CUCCUGA 3' | | GACU 5' |
| RI24_RC00 | osa-miR1318 | 1.41378 | 237.3096 | 88.44374 | BGIOSGA018749 | -1.0603 | 185.0261 | 386.9312 | Putative uncharacterized protein [Source:UniProtKB/TrEMBL;Acc:A2Y0R2] | 4 | 1060-1079 | target 5' GUCGAUCUCAUUUCUCUUGA 3' | :::: : ::::.::::.::: | query 3' CAGCCACAGUAGAGAGGACU 5' |
| RI24_RC00 | osa-miR1320 | 1.87004 | 76.27809 | 20.14065 | BGIOSGA003173 | -1.38058 | 209.3548 | 546.7088 | Putative uncharacterized protein [Source:UniProtKB/TrEMBL;Acc:A2WN00] | 4 | 277-297 | target 5' CUAGAUACUUCCUCCGUUUCA 3' | ::: : : ::::::::::.:: | query 3' GAUAUUUUAAGGAGGCAAGGU 5' |
| RI24_RC00 | osa-miR1427 | -1.02897 | 40.25788 | 83.18965 | BGIOSGA015180 | 2.97929 | 4044.965 | 512.0582 | Putative uncharacterized protein [Source:UniProtKB/TrEMBL;Acc:B8ATF3] | 2.5 | 53-73 | target 5' GCGGCACCGCGCGGUUCCGCG 3' | ::: :: ::::.:::::::::. | query 3' CGCGGUGGCGUGCCAAGGCGU 5' |
| RI24_RC00 | osa-miR1432 | 1.42616 | 357.0238 | 132.2278 | BGIOSGA013804 | -1.57716 | 2.560915 | 9.625154 | Putative uncharacterized protein [Source:UniProtKB/TrEMBL;Acc:A2XNA2] | 1 | 45-65 | target 5' GUCGGUGUCGUCUCUCCUGAU 3' | :::::: :::.:::::::::: | query 3' CAGCCACAGUAGAGAGGACUA 5' |
| RI24_RC00 | osa-miR1432 | 1.42616 | 357.0238 | 132.2278 | BGIOSGA018749 | -1.0603 | 185.0261 | 386.9312 | Putative uncharacterized protein [Source:UniProtKB/TrEMBL;Acc:A2Y0R2] | 4 | 1060-1080 | target 5' GUCGAUCUCAUUUCUCUUGAU 3' | :::: : ::::.::::.:::: | query 3' CAGCCACAGUAGAGAGGACUA 5' |
| RI24_RC00 | osa-miR156a | 1.13193 | 549201.2 | 250602.3 | BGIOSGA033954 | -1.39744 | 14.72526 | 40.42565 | Putative uncharacterized protein [Source:UniProtKB/TrEMBL;Acc:A2ZEH1] | 1 | 467-486 | target 5' GUGCUCUCUCUCUUCUGUCA 3' | :::::: :::::::::::: | query 3' CACGAGUGAGAGAAGACAGU 5' |
| RI24_RC00 | osa-miR156a | 1.13193 | 549201.2 | 250602.3 | BGIOSGA029541 | -1.8631 | 1.920686 | 9.625154 | Putative uncharacterized protein [Source:UniProtKB/TrEMBL;Acc:B8BD46] | 1 | 578-597 | target 5' GUGCUCUCUCUCUUCUGUCA 3' | :::::: :::::::::::: | query 3' CACGAGUGAGAGAAGACAGU 5' |
| RI24_RC00 | osa-miR156a | 1.13193 | 549201.2 | 250602.3 | BGIOSGA007643 | -1.27796 | 25.60915 | 63.52602 | Putative uncharacterized protein [Source:UniProtKB/TrEMBL;Acc:A2X1G6] | 1 | 716-735 | target 5' GUGCUCUCUCUCUUCUGUCA 3' | :::::: :::::::::::: | query 3' CACGAGUGAGAGAAGACAGU 5' |
| RI24_RC00 | osa-miR156a | 1.13193 | 549201.2 | 250602.3 | BGIOSGA036252 | -3.60955 | 1.920686 | 34.65056 | Putative uncharacterized protein [Source:UniProtKB/TrEMBL;Acc:A2ZK57] | 4 | 232-250 | target 5' GUGCUCACUCUC-UCUGGCA 3' | :::::: :::::: :::: :: | query 3' CACGAGUGAGAGAAGACAGU 5' |
| RI24_RC00 | osa-miR156a | 1.13193 | 549201.2 | 250602.3 | BGIOSGA036253 | -1.54845 | 0 | 1.925031 | Putative uncharacterized protein [Source:UniProtKB/TrEMBL;Acc:A2ZK56] | 4 | 232-250 | target 5' GUGCUCACUCUC-UCUGGCA 3' | :::::: :::::: :::: :: | query 3' CACGAGUGAGAGAAGACAGU 5' |
| RI24_RC00 | osa-miR156b | 1.13193 | 549201.2 | 250602.3 | BGIOSGA033954 | -1.39744 | 14.72526 | 40.42565 | Putative uncharacterized protein [Source:UniProtKB/TrEMBL;Acc:A2ZEH1] | 1 | 467-486 | target 5' GUGCUCUCUCUCUUCUGUCA 3' | :::::: :::::::::::: | query 3' CACGAGUGAGAGAAGACAGU 5' |
| RI24_RC00 | osa-miR156b | 1.13193 | 549201.2 | 250602.3 | BGIOSGA029541 | -1.8631 | 1.920686 | 9.625154 | Putative uncharacterized protein [Source:UniProtKB/TrEMBL;Acc:B8BD46] | 1 | 578-597 | target 5' GUGCUCUCUCUCUUCUGUCA 3' | :::::: :::::::::::: | query 3' CACGAGUGAGAGAAGACAGU 5' |
| RI24_RC00 | osa-miR156b | 1.13193 | 549201.2 | 250602.3 | BGIOSGA007643 | -1.27796 | 25.60915 | 63.52602 | Putative uncharacterized protein [Source:UniProtKB/TrEMBL;Acc:A2X1G6] | 1 | 716-735 | target 5' GUGCUCUCUCUCUUCUGUCA 3' | :::::: :::::::::::: | query 3' CACGAGUGAGAGAAGACAGU 5' |
| RI24_RC00 | osa-miR156b | 1.13193 | 549201.2 | 250602.3 | BGIOSGA036252 | -3.60955 | 1.920686 | 34.65056 | Putative uncharacterized protein [Source:UniProtKB/TrEMBL | 4 | 232-250 | target 5' GUGCUCACUCUC-UCUGGCA 3' | :::::: :::::: :::: :: | query 3' CACGAGUGAGAGAAGACAGU 5' |

| | | | | | | | | | | | | | | |
|---|---|---|---|---|---|---|---|---|---|---|---|---|---|---|
| | | | | | | | | | BL;Acc:A2ZK57] | | | | | |
| RI24_ RC00 | osa-miR1 56b | 1.13193 | 5492 01.2 | 2506 02.3 | BGIOSG A036253 | -1.5 4845 | 0 | 1.92 5031 | Putative uncharacterized protein [Source:UniProtKB/TrEM BL;Acc:A2ZK56] | 4 | 232-2 50 | target 5' GUGCUCACUCUC-U CUGGCA 3' | :::::: :::::: :::: :: | query 3' CACGAGUGAGAGAAGA CAGU 5' |
| RI24_ RC00 | osa-miR1 56c | 1.13193 | 5492 01.2 | 2506 02.3 | BGIOSG A033954 | -1.3 9744 | 14.7 2526 | 40.4 2565 | Putative uncharacterized protein [Source:UniProtKB/TrEM BL;Acc:A2ZEH1] | 1 | 467-4 86 | target 5' GUGCUCUCUCUCU UCUGUCA 3' | :::::: :::::::::::: | query 3' CACGAGUGAGAGAAGA CAGU 5' |
| RI24_ RC00 | osa-miR1 56c | 1.13193 | 5492 01.2 | 2506 02.3 | BGIOSG A029541 | -1.8 631 | 1.92 0686 | 9.62 5154 | Putative uncharacterized protein [Source:UniProtKB/TrEM BL;Acc:B8BD46] | 1 | 578-5 97 | target 5' GUGCUCUCUCUCU UCUGUCA 3' | :::::: :::::::::::: | query 3' CACGAGUGAGAGAAGA CAGU 5' |
| RI24_ RC00 | osa-miR1 56c | 1.13193 | 5492 01.2 | 2506 02.3 | BGIOSG A007643 | -1.2 7796 | 25.6 0915 | 63.5 2602 | Putative uncharacterized protein [Source:UniProtKB/TrEM BL;Acc:A2X1G6] | 1 | 716-7 35 | target 5' GUGCUCUCUCUCU UCUGUCA 3' | :::::: :::::::::::: | query 3' CACGAGUGAGAGAAGA CAGU 5' |
| RI24_ RC00 | osa-miR1 56c | 1.13193 | 5492 01.2 | 2506 02.3 | BGIOSG A036252 | -3.6 0955 | 1.92 0686 | 34.6 5056 | Putative uncharacterized protein [Source:UniProtKB/TrEM BL;Acc:A2ZK57] | 4 | 232-2 50 | target 5' GUGCUCACUCUC-U CUGGCA 3' | :::::: :::::: :::: :: | query 3' CACGAGUGAGAGAAGA CAGU 5' |
| RI24_ RC00 | osa-miR1 56c | 1.13193 | 5492 01.2 | 2506 02.3 | BGIOSG A036253 | -1.5 4845 | 0 | 1.92 5031 | Putative uncharacterized protein [Source:UniProtKB/TrEM BL;Acc:A2ZK56] | 4 | 232-2 50 | target 5' GUGCUCACUCUC-U CUGGCA 3' | :::::: :::::: :::: :: | query 3' CACGAGUGAGAGAAGA CAGU 5' |
| RI24_ RC00 | osa-miR1 56d | 1.13193 | 5492 01.2 | 2506 02.3 | BGIOSG A033954 | -1.3 9744 | 14.7 2526 | 40.4 2565 | Putative uncharacterized protein [Source:UniProtKB/TrEM BL;Acc:A2ZEH1] | 1 | 467-4 86 | target 5' GUGCUCUCUCUCU UCUGUCA 3' | :::::: :::::::::::: | query 3' CACGAGUGAGAGAAGA CAGU 5' |
| RI24_ RC00 | osa-miR1 56d | 1.13193 | 5492 01.2 | 2506 02.3 | BGIOSG A029541 | -1.8 631 | 1.92 0686 | 9.62 5154 | Putative uncharacterized protein [Source:UniProtKB/TrEM BL;Acc:B8BD46] | 1 | 578-5 97 | target 5' GUGCUCUCUCUCU UCUGUCA 3' | :::::: :::::::::::: | query 3' CACGAGUGAGAGAAGA CAGU 5' |
| RI24_ RC00 | osa-miR1 56d | 1.13193 | 5492 01.2 | 2506 02.3 | BGIOSG A007643 | -1.2 7796 | 25.6 0915 | 63.5 2602 | Putative uncharacterized protein [Source:UniProtKB/TrEM BL;Acc:A2X1G6] | 1 | 716-7 35 | target 5' GUGCUCUCUCUCU UCUGUCA 3' | :::::: :::::::::::: | query 3' CACGAGUGAGAGAAGA CAGU 5' |
| RI24_ RC00 | osa-miR1 56d | 1.13193 | 5492 01.2 | 2506 02.3 | BGIOSG A036252 | -3.6 0955 | 1.92 0686 | 34.6 5056 | Putative uncharacterized protein [Source:UniProtKB/TrEM BL;Acc:A2ZK57] | 4 | 232-2 50 | target 5' GUGCUCACUCUC-U CUGGCA 3' | :::::: :::::: :::: :: | query 3' CACGAGUGAGAGAAGA CAGU 5' |
| RI24_ RC00 | osa-miR1 56d | 1.13193 | 5492 01.2 | 2506 02.3 | BGIOSG A036253 | -1.5 4845 | 0 | 1.92 5031 | Putative uncharacterized protein [Source:UniProtKB/TrEM BL;Acc:A2ZK56] | 4 | 232-2 50 | target 5' GUGCUCACUCUC-U CUGGCA 3' | :::::: :::::: :::: :: | query 3' CACGAGUGAGAGAAGA CAGU 5' |
| RI24_ RC00 | osa-miR1 56e | 1.13193 | 5492 01.2 | 2506 02.3 | BGIOSG A033954 | -1.3 9744 | 14.7 2526 | 40.4 2565 | Putative uncharacterized protein [Source:UniProtKB/TrEM BL;Acc:A2ZEH1] | 1 | 467-4 86 | target 5' GUGCUCUCUCUCU UCUGUCA 3' | :::::: :::::::::::: | query 3' CACGAGUGAGAGAAGA CAGU 5' |
| RI24_ RC00 | osa-miR1 56e | 1.13193 | 5492 01.2 | 2506 02.3 | BGIOSG A029541 | -1.8 631 | 1.92 0686 | 9.62 5154 | Putative uncharacterized protein [Source:UniProtKB/TrEM BL;Acc:B8BD46] | 1 | 578-5 97 | target 5' GUGCUCUCUCUCU UCUGUCA 3' | :::::: :::::::::::: | query 3' CACGAGUGAGAGAAGA CAGU 5' |
| RI24_ RC00 | osa-miR1 56e | 1.13193 | 5492 01.2 | 2506 02.3 | BGIOSG A007643 | -1.2 7796 | 25.6 0915 | 63.5 2602 | Putative uncharacterized protein [Source:UniProtKB/TrEM | 1 | 716-7 35 | target 5' GUGCUCUCUCUCU UCUGUCA 3' | :::::: :::::::::::: | query 3' CACGAGUGAGAGAAGA CAGU 5' |

| | | | | | | | | | | | | | |
|---|---|---|---|---|---|---|---|---|---|---|---|---|---|
| | | | | | | | | | BL;Acc:A2X1G6] | | | | | |
| RI24_ RC00 | osa-miR1 56e | 1.13193 | 5492 01.2 | 2506 02.3 | BGIOSG A036252 | -3.6 0955 | 1.92 0686 | 34.6 5056 | Putative uncharacterized protein [Source:UniProtKB/TrEM BL;Acc:A2ZK57] | 4 | 232-2 50 | target 5' GUGCUCACUCUC-U CUGGCA 3' | :::::: :::::: :::: :: | query 3' CACGAGUGAGAGAAGA CAGU 5' |
| RI24_ RC00 | osa-miR1 56e | 1.13193 | 5492 01.2 | 2506 02.3 | BGIOSG A036253 | -1.5 4845 | 0 | 1.92 5031 | Putative uncharacterized protein [Source:UniProtKB/TrEM BL;Acc:A2ZK56] | 4 | 232-2 50 | target 5' GUGCUCACUCUC-U CUGGCA 3' | :::::: :::::: :::: :: | query 3' CACGAGUGAGAGAAGA CAGU 5' |
| RI24_ RC00 | osa-miR1 56f | 1.13193 | 5492 01.2 | 2506 02.3 | BGIOSG A033954 | -1.3 9744 | 14.7 2526 | 40.4 2565 | Putative uncharacterized protein [Source:UniProtKB/TrEM BL;Acc:A2ZEH1] | 1 | 467-4 86 | target 5' GUGCUCUCUCUCU UCUGUCA 3' | :::::: :::::::::::: | query 3' CACGAGUGAGAGAAGA CAGU 5' |
| RI24_ RC00 | osa-miR1 56f | 1.13193 | 5492 01.2 | 2506 02.3 | BGIOSG A029541 | -1.8 631 | 1.92 0686 | 9.62 5154 | Putative uncharacterized protein [Source:UniProtKB/TrEM BL;Acc:B8BD46] | 1 | 578-5 97 | target 5' GUGCUCUCUCUCU UCUGUCA 3' | :::::: :::::::::::: | query 3' CACGAGUGAGAGAAGA CAGU 5' |
| RI24_ RC00 | osa-miR1 56f | 1.13193 | 5492 01.2 | 2506 02.3 | BGIOSG A007643 | -1.2 7796 | 25.6 0915 | 63.5 2602 | Putative uncharacterized protein [Source:UniProtKB/TrEM BL;Acc:A2X1G6] | 1 | 716-7 35 | target 5' GUGCUCUCUCUCU UCUGUCA 3' | :::::: :::::::::::: | query 3' CACGAGUGAGAGAAGA CAGU 5' |
| RI24_ RC00 | osa-miR1 56f | 1.13193 | 5492 01.2 | 2506 02.3 | BGIOSG A036252 | -3.6 0955 | 1.92 0686 | 34.6 5056 | Putative uncharacterized protein [Source:UniProtKB/TrEM BL;Acc:A2ZK57] | 4 | 232-2 50 | target 5' GUGCUCACUCUC-U CUGGCA 3' | :::::: :::::: :::: :: | query 3' CACGAGUGAGAGAAGA CAGU 5' |
| RI24_ RC00 | osa-miR1 56f | 1.13193 | 5492 01.2 | 2506 02.3 | BGIOSG A036253 | -1.5 4845 | 0 | 1.92 5031 | Putative uncharacterized protein [Source:UniProtKB/TrEM BL;Acc:A2ZK56] | 4 | 232-2 50 | target 5' GUGCUCACUCUC-U CUGGCA 3' | :::::: :::::: :::: :: | query 3' CACGAGUGAGAGAAGA CAGU 5' |
| RI24_ RC00 | osa-miR1 56g | 1.13193 | 5492 01.2 | 2506 02.3 | BGIOSG A033954 | -1.3 9744 | 14.7 2526 | 40.4 2565 | Putative uncharacterized protein [Source:UniProtKB/TrEM BL;Acc:A2ZEH1] | 1 | 467-4 86 | target 5' GUGCUCUCUCUCU UCUGUCA 3' | :::::: :::::::::::: | query 3' CACGAGUGAGAGAAGA CAGU 5' |
| RI24_ RC00 | osa-miR1 56g | 1.13193 | 5492 01.2 | 2506 02.3 | BGIOSG A029541 | -1.8 631 | 1.92 0686 | 9.62 5154 | Putative uncharacterized protein [Source:UniProtKB/TrEM BL;Acc:B8BD46] | 1 | 578-5 97 | target 5' GUGCUCUCUCUCU UCUGUCA 3' | :::::: :::::::::::: | query 3' CACGAGUGAGAGAAGA CAGU 5' |
| RI24_ RC00 | osa-miR1 56g | 1.13193 | 5492 01.2 | 2506 02.3 | BGIOSG A007643 | -1.2 7796 | 25.6 0915 | 63.5 2602 | Putative uncharacterized protein [Source:UniProtKB/TrEM BL;Acc:A2X1G6] | 1 | 716-7 35 | target 5' GUGCUCUCUCUCU UCUGUCA 3' | :::::: :::::::::::: | query 3' CACGAGUGAGAGAAGA CAGU 5' |
| RI24_ RC00 | osa-miR1 56g | 1.13193 | 5492 01.2 | 2506 02.3 | BGIOSG A036252 | -3.6 0955 | 1.92 0686 | 34.6 5056 | Putative uncharacterized protein [Source:UniProtKB/TrEM BL;Acc:A2ZK57] | 4 | 232-2 50 | target 5' GUGCUCACUCUC-U CUGGCA 3' | :::::: :::::: :::: :: | query 3' CACGAGUGAGAGAAGA CAGU 5' |
| RI24_ RC00 | osa-miR1 56g | 1.13193 | 5492 01.2 | 2506 02.3 | BGIOSG A036253 | -1.5 4845 | 0 | 1.92 5031 | Putative uncharacterized protein [Source:UniProtKB/TrEM BL;Acc:A2ZK56] | 4 | 232-2 50 | target 5' GUGCUCACUCUC-U CUGGCA 3' | :::::: :::::: :::: :: | query 3' CACGAGUGAGAGAAGA CAGU 5' |
| RI24_ RC00 | osa-miR1 56h | 1.13193 | 5492 01.2 | 2506 02.3 | BGIOSG A033954 | -1.3 9744 | 14.7 2526 | 40.4 2565 | Putative uncharacterized protein [Source:UniProtKB/TrEM BL;Acc:A2ZEH1] | 1 | 467-4 86 | target 5' GUGCUCUCUCUCU UCUGUCA 3' | :::::: :::::::::::: | query 3' CACGAGUGAGAGAAGA CAGU 5' |
| RI24_ RC00 | osa-miR1 56h | 1.13193 | 5492 01.2 | 2506 02.3 | BGIOSG A029541 | -1.8 631 | 1.92 0686 | 9.62 5154 | Putative uncharacterized protein [Source:UniProtKB/TrEM | 1 | 578-5 97 | target 5' GUGCUCUCUCUCU UCUGUCA 3' | :::::: :::::::::::: | query 3' CACGAGUGAGAGAAGA CAGU 5' |

| | | | | | | | | | | | | | | |
|---|---|---|---|---|---|---|---|---|---|---|---|---|---|---|
| | | | | | | | | | BL;Acc:B8BD46] | | | | | |
| RI24_ RC00 | osa-miR1 56h | 1.13193 | 5492 01.2 | 2506 02.3 | BGIOSG A007643 | -1.2 7796 | 25.6 0915 | 63.5 2602 | Putative uncharacterized protein [Source:UniProtKB/TrEM BL;Acc:A2X1G6] | 1 | 716-7 35 | target 5' GUGCUCUCUCUCU UCUGUCA 3' | :::::: ::::::::::::: | query 3' CACGAGUGAGAGAAGA CAGU 5' |
| RI24_ RC00 | osa-miR1 56h | 1.13193 | 5492 01.2 | 2506 02.3 | BGIOSG A036252 | -3.6 0955 | 1.92 0686 | 34.6 5056 | Putative uncharacterized protein [Source:UniProtKB/TrEM BL;Acc:A2ZK57] | 4 | 232-2 50 | target 5' GUGCUCACUCUC-U CUGGCA 3' | :::::: :::::: :::: :: | query 3' CACGAGUGAGAGAAGA CAGU 5' |
| RI24_ RC00 | osa-miR1 56h | 1.13193 | 5492 01.2 | 2506 02.3 | BGIOSG A036253 | -1.5 4845 | 0 | 1.92 5031 | Putative uncharacterized protein [Source:UniProtKB/TrEM BL;Acc:A2ZK56] | 4 | 232-2 50 | target 5' GUGCUCACUCUC-U CUGGCA 3' | :::::: :::::: :::: :: | query 3' CACGAGUGAGAGAAGA CAGU 5' |
| RI24_ RC00 | osa-miR1 56i | 1.13193 | 5492 01.2 | 2506 02.3 | BGIOSG A033954 | -1.3 9744 | 14.7 2526 | 40.4 2565 | Putative uncharacterized protein [Source:UniProtKB/TrEM BL;Acc:A2ZEH1] | 1 | 467-4 86 | target 5' GUGCUCUCUCUCU UCUGUCA 3' | :::::: ::::::::::::: | query 3' CACGAGUGAGAGAAGA CAGU 5' |
| RI24_ RC00 | osa-miR1 56i | 1.13193 | 5492 01.2 | 2506 02.3 | BGIOSG A029541 | -1.8 631 | 1.92 0686 | 9.62 5154 | Putative uncharacterized protein [Source:UniProtKB/TrEM BL;Acc:B8BD46] | 1 | 578-5 97 | target 5' GUGCUCUCUCUCU UCUGUCA 3' | :::::: ::::::::::::: | query 3' CACGAGUGAGAGAAGA CAGU 5' |
| RI24_ RC00 | osa-miR1 56i | 1.13193 | 5492 01.2 | 2506 02.3 | BGIOSG A007643 | -1.2 7796 | 25.6 0915 | 63.5 2602 | Putative uncharacterized protein [Source:UniProtKB/TrEM BL;Acc:A2X1G6] | 1 | 716-7 35 | target 5' GUGCUCUCUCUCU UCUGUCA 3' | :::::: ::::::::::::: | query 3' CACGAGUGAGAGAAGA CAGU 5' |
| RI24_ RC00 | osa-miR1 56i | 1.13193 | 5492 01.2 | 2506 02.3 | BGIOSG A036252 | -3.6 0955 | 1.92 0686 | 34.6 5056 | Putative uncharacterized protein [Source:UniProtKB/TrEM BL;Acc:A2ZK57] | 4 | 232-2 50 | target 5' GUGCUCACUCUC-U CUGGCA 3' | :::::: :::::: :::: :: | query 3' CACGAGUGAGAGAAGA CAGU 5' |
| RI24_ RC00 | osa-miR1 56i | 1.13193 | 5492 01.2 | 2506 02.3 | BGIOSG A036253 | -1.5 4845 | 0 | 1.92 5031 | Putative uncharacterized protein [Source:UniProtKB/TrEM BL;Acc:A2ZK56] | 4 | 232-2 50 | target 5' GUGCUCACUCUC-U CUGGCA 3' | :::::: :::::: :::: :: | query 3' CACGAGUGAGAGAAGA CAGU 5' |
| RI24_ RC00 | osa-miR1 56j | 1.13193 | 5492 01.2 | 2506 02.3 | BGIOSG A033954 | -1.3 9744 | 14.7 2526 | 40.4 2565 | Putative uncharacterized protein [Source:UniProtKB/TrEM BL;Acc:A2ZEH1] | 1 | 467-4 86 | target 5' GUGCUCUCUCUCU UCUGUCA 3' | :::::: ::::::::::::: | query 3' CACGAGUGAGAGAAGA CAGU 5' |
| RI24_ RC00 | osa-miR1 56j | 1.13193 | 5492 01.2 | 2506 02.3 | BGIOSG A029541 | -1.8 631 | 1.92 0686 | 9.62 5154 | Putative uncharacterized protein [Source:UniProtKB/TrEM BL;Acc:B8BD46] | 1 | 578-5 97 | target 5' GUGCUCUCUCUCU UCUGUCA 3' | :::::: ::::::::::::: | query 3' CACGAGUGAGAGAAGA CAGU 5' |
| RI24_ RC00 | osa-miR1 56j | 1.13193 | 5492 01.2 | 2506 02.3 | BGIOSG A007643 | -1.2 7796 | 25.6 0915 | 63.5 2602 | Putative uncharacterized protein [Source:UniProtKB/TrEM BL;Acc:A2X1G6] | 1 | 716-7 35 | target 5' GUGCUCUCUCUCU UCUGUCA 3' | :::::: ::::::::::::: | query 3' CACGAGUGAGAGAAGA CAGU 5' |
| RI24_ RC00 | osa-miR1 56j | 1.13193 | 5492 01.2 | 2506 02.3 | BGIOSG A036252 | -3.6 0955 | 1.92 0686 | 34.6 5056 | Putative uncharacterized protein [Source:UniProtKB/TrEM BL;Acc:A2ZK57] | 4 | 232-2 50 | target 5' GUGCUCACUCUC-U CUGGCA 3' | :::::: :::::: :::: :: | query 3' CACGAGUGAGAGAAGA CAGU 5' |
| RI24_ RC00 | osa-miR1 56j | 1.13193 | 5492 01.2 | 2506 02.3 | BGIOSG A036253 | -1.5 4845 | 0 | 1.92 5031 | Putative uncharacterized protein [Source:UniProtKB/TrEM BL;Acc:A2ZK56] | 4 | 232-2 50 | target 5' GUGCUCACUCUC-U CUGGCA 3' | :::::: :::::: :::: :: | query 3' CACGAGUGAGAGAAGA CAGU 5' |
| RI24_ RC00 | osa-miR1 56k | 1.13076 | 5419 29.4 | 2474 84.8 | BGIOSG A029541 | -1.8 631 | 1.92 0686 | 9.62 5154 | Putative uncharacterized protein [Source:UniProtKB/TrEM | 0 | 577-5 97 | target 5' UGUGCUCUCUCUC UUCUGUCA 3' | :::::: ::::::::::::::: | query 3' ACACGAGAGAGAGAAG ACAGU 5' |

| | | | | | | | | | | | | | | |
|---|---|---|---|---|---|---|---|---|---|---|---|---|---|---|
| | | | | | | | | | BL;Acc:B8BD46] | | | | | |
| RI24_ RC00 | osa-miR1 56k | 1.13076 | 5419 29.4 | 2474 84.8 | BGIOSG A033954 | -1.3 9744 | 14.7 2526 | 40.4 2565 | Putative uncharacterized protein [Source:UniProtKB/TrEM BL;Acc:A2ZEH1] | 0 | 466-4 86 | target 5' UGUGCUCUCUCUC UUCUGUCA 3' | :::::: :::::::::::::: | query 3' ACACGAGAGAGAGAAG ACAGU 5' |
| RI24_ RC00 | osa-miR1 56k | 1.13076 | 5419 29.4 | 2474 84.8 | BGIOSG A007643 | -1.2 7796 | 25.6 0915 | 63.5 2602 | Putative uncharacterized protein [Source:UniProtKB/TrEM BL;Acc:A2X1G6] | 1 | 715-7 35 | target 5' CGUGCUCUCUCUC UUCUGUCA 3' | :::: ::::::::::::::: | query 3' ACACGAGAGAGAGAAG ACAGU 5' |
| RI24_ RC00 | osa-miR1 56l | 1.29835 | 3705 .844 | 1506 .171 | BGIOSG A029541 | -1.8 631 | 1.92 0686 | 9.62 5154 | Putative uncharacterized protein [Source:UniProtKB/TrEM BL;Acc:B8BD46] | 2.5 | 577-5 97 | target 5' UGUGCUCUCUCUC UUCUGUCA 3' | :.:::: : ::::::::::: | query 3' AUACGAGUGAGAGAA GACAGC 5' |
| RI24_ RC00 | osa-miR1 56l | 1.29835 | 3705 .844 | 1506 .171 | BGIOSG A033954 | -1.3 9744 | 14.7 2526 | 40.4 2565 | Putative uncharacterized protein [Source:UniProtKB/TrEM BL;Acc:A2ZEH1] | 2.5 | 466-4 86 | target 5' UGUGCUCUCUCUC UUCUGUCA 3' | :.:::: : ::::::::::: | query 3' AUACGAGUGAGAGAA GACAGC 5' |
| RI24_ RC00 | osa-miR1 56l | 1.29835 | 3705 .844 | 1506 .171 | BGIOSG A007643 | -1.2 7796 | 25.6 0915 | 63.5 2602 | Putative uncharacterized protein [Source:UniProtKB/TrEM BL;Acc:A2X1G6] | 3.5 | 715-7 35 | target 5' CGUGCUCUCUCUC UUCUGUCA 3' | .::: :: ::::::::::: | query 3' AUACGAGUGAGAGAA GACAGC 5' |
| RI24_ RC00 | osa-miR1 56l | 1.29835 | 3705 .844 | 1506 .171 | BGIOSG A004831 | -1.4 3729 | 12.1 6435 | 34.6 5056 | Putative uncharacterized protein [Source:UniProtKB/TrEM BL;Acc:B8ACH2] | 4 | 1515- 1534 | target 5' UAUACU-ACUCUCU UUUGUUG 3' | ::: :: :::::::.:::.: | query 3' AUACGAGUGAGAGAA GACAGC 5' |
| RI24_ RC00 | osa-miR1 59a.2 | 1.50618 | 46.6 1439 | 15.7 6225 | BGIOSG A033431 | -1.5 6411 | 0.64 0229 | 3.85 0062 | Putative uncharacterized protein [Source:UniProtKB/TrEM BL;Acc:B8BI71] | 4 | 1782- 1802 | target 5' UGCAGUUCUUAGG GCAUGCAG 3' | :::::. ::.: :::::::::. | query 3' ACGUCGAGGACCCCGU ACGUU 5' |
| RI24_ RC00 | osa-miR1 846d-5p | 1.24435 | 15.8 9127 | 6.12 9764 | BGIOSG A016941 | -1.51 175 | 24.3 2869 | 71.2 2614 | Putative uncharacterized protein [Source:UniProtKB/TrEM BL;Acc:B8AT39] | 4 | 268-2 89 | target 5' GAGAAGCACCUGC UCGGUGGGA 3' | :::: : :::::::::::: | query 3' CUCUAGGCCGACGAGC CACCCU 5' |
| RI24_ RC00 | osa-miR1 850.1 | 1.62655 | 158. 9127 | 50.7 8947 | BGIOSG A012292 | -1.7 4265 | 191. 4284 | 642. 9603 | Putative uncharacterized protein [Source:UniProtKB/TrEM BL;Acc:A2XEV9] | 4 | 472-4 93 | target 5' CACCAUUUCUCCCA ACUUUUCA 3' | : ::: ::::::::::::.:: | query 3' GGGGU-UAGAGGGUUG AAAGGU 5' |
| RI24_ RC00 | osa-miR1 879 | 2.1704 | 78.3 9693 | 16.6 3793 | BGIOSG A001273 | -1.0 3567 | 5.12 183 | 11.5 5019 | Putative uncharacterized protein [Source:UniProtKB/TrEM BL;Acc:A2WS92] | 2.5 | 640-6 62 | target 5' CCACC-CACCCCUA AACCAAACAU 3' | ::::: :: ::::::::::::::. | query 3' GGUGGAGUAGGGAUU UGGUUUGUG 5' |
| RI24_ RC00 | osa-miR5 28 | 1.75327 | 2247 9.79 | 6667 .432 | BGIOSG A024054 | -3.4 4565 | 7.04 2517 | 86.6 2639 | Putative uncharacterized protein [Source:UniProtKB/TrEM BL;Acc:B8B7J3] | 2 | 378-3 97 | target 5' CUCCUCUGC-UGCC CCUUCCA 3' | :::::: ::: ::::::::::: | query 3' GAGGAGACGUACGGGG AAGGU 5' |
| RI24_ RC00 | osa-miR5 28 | 1.75327 | 2247 9.79 | 6667 .432 | BGIOSG A009946 | -1.7 9589 | 15.3 6549 | 55.8 259 | Putative uncharacterized protein [Source:UniProtKB/TrEM BL;Acc:A2XL83] | 4 | 61-80 | target 5' CUCCUCUGCGUGC UCC-UCCA 3' | :::::: :::.:::.:: :::: | query 3' GAGGAGACGUACGGGG AAGGU 5' |
| RI24_ RC00 | osa-miR5 28 | 1.75327 | 2247 9.79 | 6667 .432 | BGIOSG A013952 | -1.5 4845 | 0 | 1.92 5031 | Putative uncharacterized protein [Source:UniProtKB/TrEM BL;Acc:A2XP44] | 4 | 46-65 | target 5' CUCCUCUGCGUGC UCC-UCCA 3' | :::::: :::.:::.:: :::: | query 3' GAGGAGACGUACGGGG AAGGU 5' |
| RI24_ RC00 | osa-miR5 35 | 1.32822 | 6516 .48 | 2594 .642 | BGIOSG A007643 | -1.2 7796 | 25.6 0915 | 63.5 2602 | Putative uncharacterized protein [Source:UniProtKB/TrEM | 4 | 714-7 35 | target 5' GCGUGCUCUCUCU CUUCUGUCA 3' | :::::: :::::::: : ::::: | query 3' CGCACGAGAGAGAGCA -ACAGU 5' |

| | | | | | | | | | | | | | | |
|---|---|---|---|---|---|---|---|---|---|---|---|---|---|---|
| | | | | | | | | | BL;Acc:A2X1G6] | | | | | |
| RI24_ RI08 | osa-miR159e | 1.7756 | 58631.37 | 17123.94 | BGIOSGA013684 | -1.30951 | 0 | 1.47857 | Transcription factor PCF6 [Source:UniProtKB/Swiss-Prot;Acc:A2XMN1] | 3.5 | 850-870 | target 5' AGGGGGACCCUUC AGUCCAAU 3' | :::.:<br>:::::::.:::::: | query 3' UCCUCGAGGGAAGUUA GGUUA 5' |
| RI24_ RI08 | osa-miR160a | -1.53748 | 143.0214 | 417.0751 | BGIOSGA014036 | 1.18932 | 1.280458 | 0 | Putative uncharacterized protein [Source:UniProtKB/TrEMBL;Acc:A2XZ93] | 2 | 1185-1205 | target 5' UGACAUUCAGGGA GCCAGGCA 3' | :: :::<br>::::::::::::: | query 3' ACCGUAUGUCCCUCGG UCCGU 5' |
| RI24_ RI08 | osa-miR160b | -1.53748 | 143.0214 | 417.0751 | BGIOSGA014036 | 1.18932 | 1.280458 | 0 | Putative uncharacterized protein [Source:UniProtKB/TrEMBL;Acc:A2XZ93] | 2 | 1185-1205 | target 5' UGACAUUCAGGGA GCCAGGCA 3' | :: :::<br>::::::::::::: | query 3' ACCGUAUGUCCCUCGG UCCGU 5' |
| RI24_ RI08 | osa-miR160c | -1.53748 | 143.0214 | 417.0751 | BGIOSGA014036 | 1.18932 | 1.280458 | 0 | Putative uncharacterized protein [Source:UniProtKB/TrEMBL;Acc:A2XZ93] | 2 | 1185-1205 | target 5' UGACAUUCAGGGA GCCAGGCA 3' | :: :::<br>::::::::::::: | query 3' ACCGUAUGUCCCUCGG UCCGU 5' |
| RI24_ RI08 | osa-miR160d | -1.53748 | 143.0214 | 417.0751 | BGIOSGA014036 | 1.18932 | 1.280458 | 0 | Putative uncharacterized protein [Source:UniProtKB/TrEMBL;Acc:A2XZ93] | 2 | 1185-1205 | target 5' UGACAUUCAGGGA GCCAGGCA 3' | :: :::<br>::::::::::::: | query 3' ACCGUAUGUCCCUCGG UCCGU 5' |
| RI24_ RI08 | osa-miR160e | -1.57752 | 108.0606 | 324.4978 | BGIOSGA014036 | 1.18932 | 1.280458 | 0 | Putative uncharacterized protein [Source:UniProtKB/TrEMBL;Acc:A2XZ93] | 2.5 | 1185-1205 | target 5' UGACAUUCAGGGA GCCAGGCA 3' | .: :::<br>::::::::::::: | query 3' GCCGUAUGUCCCUCGG UCCGU 5' |
| RI24_ RI08 | osa-miR160f | -1.57075 | 110.1795 | 329.2698 | BGIOSGA014036 | 1.18932 | 1.280458 | 0 | Putative uncharacterized protein [Source:UniProtKB/TrEMBL;Acc:A2XZ93] | 1 | 1185-1205 | target 5' UGACAUUCAGGGA GCCAGGCA 3' | :: :::<br>:::::::::::::: | query 3' ACCGUAAGUCCCUCGG UCCGU 5' |
| RI24_ RI08 | osa-miR169d | 1.16355 | 99.58529 | 43.90264 | BGIOSGA016519 | -1.27056 | 0.640229 | 2.957139 | Putative uncharacterized protein [Source:UniProtKB/TrEMBL;Acc:A2XU39] | 4 | 101-122 | target 5' CCGGCGGCCUCAUC UUUGGCUA 3' | :::::.<br>. :::::.::::::: | query 3' GGCCGUU-AAGUAGGA ACCGAU 5' |
| RI24_ RI08 | osa-miR396a | 1.02777 | 1145.231 | 561.1903 | BGIOSGA013499 | -1.27056 | 0.640229 | 2.957139 | Growth regulating factor 1 [Source:UniProtKB/TrEMBL;Acc:A6MZS4] | 3 | 777-798 | target 5' CCGUUCAAGAAAG CAUGUGGAA 3' | : ::::<br>:::::::: :::::: | query 3' GUCAAGUUCUUUCG-A CACCUU 5' |
| RI24_ RI08 | osa-miR396a | 1.02777 | 1145.231 | 561.1903 | BGIOSGA005785 | -1.98446 | 0 | 2.957139 | Putative uncharacterized protein [Source:UniProtKB/TrEMBL;Acc:B8AH82] | 3 | 483-504 | target 5' CCGUUCAAGAAAG CCUGUGGAA 3' | : ::::<br>::::::: :::::::: | query 3' GUCAAGUUCUUUC-GA CACCUU 5' |
| RI24_ RI08 | osa-miR396a | 1.02777 | 1145.231 | 561.1903 | BGIOSGA014417 | -2.36507 | 10.24366 | 56.92493 | Putative uncharacterized protein [Source:UniProtKB/TrEMBL;Acc:B8ATM9] | 3 | 450-471 | target 5' CCGUUCAAGAAAG CCUGUGGAA 3' | : ::::<br>::::::: :::::::: | query 3' GUCAAGUUCUUUC-GA CACCUU 5' |
| RI24_ RI08 | osa-miR396a | 1.02777 | 1145.231 | 561.1903 | BGIOSGA008811 | -1.30951 | 0 | 1.47857 | Putative uncharacterized protein [Source:UniProtKB/TrEMBL;Acc:A2X8B1] | 3 | 507-528 | target 5' CCGUUCAAGAAAG CCUGUGGAA 3' | : ::::<br>::::::: :::::::: | query 3' GUCAAGUUCUUUC-GA CACCUU 5' |
| RI24_ RI08 | osa-miR396b | 1.02777 | 1145.231 | 561.1903 | BGIOSGA013499 | -1.27056 | 0.640229 | 2.957139 | Growth regulating factor 1 [Source:UniProtKB/TrEMBL;Acc:A6MZS4] | 3 | 777-798 | target 5' CCGUUCAAGAAAG CAUGUGGAA 3' | : ::::<br>:::::::: :::::: | query 3' GUCAAGUUCUUUCG-A CACCUU 5' |
| RI24_ RI08 | osa-miR396b | 1.02777 | 1145.231 | 561.1903 | BGIOSGA005785 | -1.98446 | 0 | 2.957139 | Putative uncharacterized protein [Source:UniProtKB/TrEMBL;Acc:B8AH82] | 3 | 483-504 | target 5' CCGUUCAAGAAAG CCUGUGGAA 3' | : ::::<br>::::::: :::::::: | query 3' GUCAAGUUCUUUC-GA CACCUU 5' |
| RI24_ RI08 | osa-miR396b | 1.02777 | 1145.231 | 561.1903 | BGIOSGA014417 | -2.36507 | 10.24366 | 56.92493 | Putative uncharacterized protein | 3 | 450-471 | target 5' CCGUUCAAGAAAG | : ::::<br>::::::: :::::::: | query 3' GUCAAGUUCUUUC-GA |

| | | | | | | | | | | | | | |
|---|---|---|---|---|---|---|---|---|---|---|---|---|---|
| | | | | | | | | | [Source:UniProtKB/TrEMBL;Acc:B8ATM9] | | | CCUGUGGAA 3' | | CACCUU 5' |
| RI24_RI08 | osa-miR396b | 1.02777 | 1145.231 | 561.1903 | BGIOSGA008811 | -1.30951 | 0 | 1.47857 | Putative uncharacterized protein [Source:UniProtKB/TrEMBL;Acc:A2X8B1] | 3 | 507-528 | target 5' CCGUUCAAGAAAG CCUGUGGAA 3' | : :::: ::::::: ::::::: | query 3' GUCAAGUUCUUUC-GA CACCUU 5' |
| RI24_RI08 | osa-miR396f | 1.45189 | 1813.724 | 662.3572 | BGIOSGA005785 | -1.98446 | 0 | 2.957139 | Putative uncharacterized protein [Source:UniProtKB/TrEMBL;Acc:B8AH82] | 3 | 484-505 | target 5' CGUUCAAGAAAGC CUGUGGAAA 3' | :::: ::::::::::::: : | query 3' UCAAGUUCUUUCGGAC ACCUCU 5' |
| RI24_RI08 | osa-miR396f | 1.45189 | 1813.724 | 662.3572 | BGIOSGA014417 | -2.36507 | 10.24366 | 56.92493 | Putative uncharacterized protein [Source:UniProtKB/TrEMBL;Acc:B8ATM9] | 3.5 | 451-472 | target 5' CGUUCAAGAAAGC CUGUGGAAG 3' | :::: ::::::::::::: . | query 3' UCAAGUUCUUUCGGAC ACCUCU 5' |
| RI24_RI08 | osa-miR396f | 1.45189 | 1813.724 | 662.3572 | BGIOSGA008811 | -1.30951 | 0 | 1.47857 | Putative uncharacterized protein [Source:UniProtKB/TrEMBL;Acc:A2X8B1] | 3.5 | 508-529 | target 5' CGUUCAAGAAAGC CUGUGGAAG 3' | :::: ::::::::::::: . | query 3' UCAAGUUCUUUCGGAC ACCUCU 5' |
| RI24_RI08 | osa-miR399a | 1.19134 | 1134.637 | 496.2907 | BGIOSGA031778 | -1.40587 | 3.841373 | 11.82856 | Putative uncharacterized protein [Source:UniProtKB/TrEMBL;Acc:B8BH91] | 2 | 4213-4232 | target 5' CA-GGCAAUUUUCC UUUGGCA 3' | :: ::: ::::.:::::::::: | query 3' GUCCCGUUAAGAGGAA ACCGU 5' |
| RI24_RI08 | osa-miR399b | 1.19134 | 1134.637 | 496.2907 | BGIOSGA031778 | -1.40587 | 3.841373 | 11.82856 | Putative uncharacterized protein [Source:UniProtKB/TrEMBL;Acc:B8BH91] | 2 | 4213-4232 | target 5' CA-GGCAAUUUUCC UUUGGCA 3' | :: ::: ::::.:::::::::: | query 3' GUCCCGUUAAGAGGAA ACCGU 5' |
| RI24_RI08 | osa-miR399c | 1.19134 | 1134.637 | 496.2907 | BGIOSGA031778 | -1.40587 | 3.841373 | 11.82856 | Putative uncharacterized protein [Source:UniProtKB/TrEMBL;Acc:B8BH91] | 2 | 4213-4232 | target 5' CA-GGCAAUUUUCC UUUGGCA 3' | :: ::: ::::.:::::::::: | query 3' GUCCCGUUAAGAGGAA ACCGU 5' |
| RI24_RI08 | osa-miR399d | 1.16688 | 1494.839 | 665.2204 | BGIOSGA031778 | -1.40587 | 3.841373 | 11.82856 | Putative uncharacterized protein [Source:UniProtKB/TrEMBL;Acc:B8BH91] | 3 | 4213-4232 | target 5' CA-GGCAAUUUUCC UUUGGCA 3' | :: ::: ::.:.:::::::::: | query 3' GUCCCGUUGAGAGGAA ACCGU 5' |
| RI24_RI08 | osa-miR399d | 1.16688 | 1494.839 | 665.2204 | BGIOSGA017239 | -1.352 | 30.09075 | 78.36419 | Putative uncharacterized protein [Source:UniProtKB/TrEMBL;Acc:B8AV88] | 3 | 3888-3907 | target 5' UA-GCCAGCUCUCC UUUGGCA 3' | .: : : :.::::::::::::: | query 3' GUCCCGUUGAGAGGAA ACCGU 5' |
| RI24_RI08 | osa-miR399e | 1.52505 | 141.962 | 48.67467 | BGIOSGA029256 | -1.32965 | 253.5306 | 638.7421 | Putative uncharacterized protein [Source:UniProtKB/TrEMBL;Acc:B8BEL6] | 4 | 721-740 | target 5' GUGGGC-AAUCUCC CUUGGCA 3' | :::: : ::::::: :::::: | query 3' GACCCGUUUAGAGGAA ACCGU 5' |
| RI24_RI08 | osa-miR399f | 1.52505 | 141.962 | 48.67467 | BGIOSGA029256 | -1.32965 | 253.5306 | 638.7421 | Putative uncharacterized protein [Source:UniProtKB/TrEMBL;Acc:B8BEL6] | 4 | 721-740 | target 5' GUGGGC-AAUCUCC CUUGGCA 3' | :::: : ::::::: :::::: | query 3' GACCCGUUUAGAGGAA ACCGU 5' |
| RI24_RI08 | osa-miR399g | 1.52505 | 141.962 | 48.67467 | BGIOSGA029256 | -1.32965 | 253.5306 | 638.7421 | Putative uncharacterized protein [Source:UniProtKB/TrEMBL;Acc:B8BEL6] | 4 | 721-740 | target 5' GUGGGC-AAUCUCC CUUGGCA 3' | :::: : ::::::: :::::: | query 3' GACCCGUUUAGAGGAA ACCGU 5' |
| RI24_RI08 | osa-miR399i | 1.18371 | 1065.774 | 468.613 | BGIOSGA017239 | -1.352 | 30.09075 | 78.36419 | Putative uncharacterized protein [Source:UniProtKB/TrEMBL;Acc:B8AV88] | 2.5 | 3888-3907 | target 5' UA-GCCAGCUCUCC UUUGGCA 3' | .: : : :::::::::::::: | query 3' GUCCCGUCGAGAGGAA ACCGU 5' |
| RI24_RI08 | osa-miR399i | 1.18371 | 1065.774 | 468.613 | BGIOSGA031778 | -1.40587 | 3.841373 | 11.82856 | Putative uncharacterized protein [Source:UniProtKB/TrEMBL | 4 | 4213-4232 | target 5' CA-GGCAAUUUUCC UUUGGCA 3' | :: ::: : .:.:::::::::: | query 3' GUCCCGUCGAGAGGAA ACCGU 5' |

| | | | | | | | | | | | | | | |
|---|---|---|---|---|---|---|---|---|---|---|---|---|---|---|
| | | | | | | | | | BL;Acc:B8BH91] | | | | | |
| RI24_ RI08 | osa-miR3 99j | 1.13041 | 1363 .471 | 622. 2722 | BGIOSG A017239 | -1.3 52 | 30.0 9075 | 78.3 6419 | Putative uncharacterized protein [Source:UniProtKB/TrEM BL;Acc:B8AV88] | 2.5 | 3888- 3907 | target 5' UA-GCCAGCUCUCC UUUGGCA 3' | :: : : :.:::::::::::: | query 3' AUCCCGUUGAGAGGAA ACCGU 5' |
| RI24_ RI08 | osa-miR4 44b.2 | 1.40794 | 1161 8.64 | 4377 .857 | BGIOSG A008494 | -4.3 3781 | 0 | 19.2 2141 | Putative uncharacterized protein [Source:UniProtKB/TrEM BL;Acc:B8AED3] | 0 | 282-3 02 | target 5' AAGCUUGAGACAA CAACUGCA 3' | :::::: :::::::::::::: | query 3' UUCGAACUCUGUUGUU GACGU 5' |
| RI24_ RI08 | osa-miR4 44c.2 | 1.40794 | 1161 8.64 | 4377 .857 | BGIOSG A008494 | -4.3 3781 | 0 | 19.2 2141 | Putative uncharacterized protein [Source:UniProtKB/TrEM BL;Acc:B8AED3] | 0 | 282-3 02 | target 5' AAGCUUGAGACAA CAACUGCA 3' | :::::: :::::::::::::: | query 3' UUCGAACUCUGUUGUU GACGU 5' |
| RI24_ RI08 | osa-miR4 44f | 1.24508 | 1328 2.98 | 5603 .313 | BGIOSG A008494 | -4.3 3781 | 0 | 19.2 2141 | Putative uncharacterized protein [Source:UniProtKB/TrEM BL;Acc:B8AED3] | 2 | 282-3 02 | target 5' AAGCUUGAGACAA CAACUGCA 3' | :::::: ::: :::::::::: | query 3' UUCGAACUCCGUUGUU GACGU 5' |
| RI24_ RI08 | osa-miR5 28 | 1.18012 | 2247 9.79 | 9920 .088 | BGIOSG A013952 | -2.7 8958 | 0 | 5.91 4279 | Putative uncharacterized protein [Source:UniProtKB/TrEM BL;Acc:A2XP44] | 4 | 46-65 | target 5' CUCCUCUGCGUGC UCC-UCCA 3' | :::::: :::.:::.:: :::: | query 3' GAGGAGACGUACGGGG AAGGU 5' |
| RI24_ RI08 | osa-miR5 28 | 1.18012 | 2247 9.79 | 9920 .088 | BGIOSG A030596 | -1.3 0951 | 0 | 1.47 857 | Putative uncharacterized protein [Source:UniProtKB/TrEM BL;Acc:B8BET4] | 4 | 320-3 41 | target 5' CUCCUCUUGCGUG CCCCUACCA 3' | :::::: :::.::::::: ::: | query 3' GAGGAG-ACGUACGGG GAAGGU 5' |
| RI24_ SI24 | osa-miR1 318 | 2.67179 | 237. 3096 | 36.3 9839 | BGIOSG A013804 | -2.3 1244 | 2.56 0915 | 16.6 8782 | Putative uncharacterized protein [Source:UniProtKB/TrEM BL;Acc:A2XNA2] | 1 | 45-64 | target 5' GUCGGUGUCGUCU CUCCUGA 3' | :::::: :::.:::::::::: | query 3' CAGCCACAGUAGAGAG GACU 5' |
| RI24_ SI24 | osa-miR1 318 | 2.67179 | 237. 3096 | 36.3 9839 | BGIOSG A018749 | -1.4 2081 | 185. 0261 | 497. 0585 | Putative uncharacterized protein [Source:UniProtKB/TrEM BL;Acc:A2Y0R2] | 4 | 1060- 1079 | target 5' GUCGAUCUCAUUU CUCUUGA 3' | :::: : ::::.::::.::: | query 3' CAGCCACAGUAGAGAG GACU 5' |
| RI24_ SI24 | osa-miR1 320 | 1.92598 | 76.2 7809 | 19.3 3665 | BGIOSG A003173 | -1.8 65 | 209. 3548 | 765. 2555 | Putative uncharacterized protein [Source:UniProtKB/TrEM BL;Acc:A2WN00] | 4 | 277-2 97 | target 5' CUAGAUACUUCCU CCGUUUCA 3' | ::: : : ::::::::::.:: | query 3' GAUAUUUUAAGGAGG CAAGGU 5' |
| RI24_ SI24 | osa-miR1 320 | 1.92598 | 76.2 7809 | 19.3 3665 | BGIOSG A021582 | -1.8 1416 | 0.64 0229 | 4.76 7947 | Putative uncharacterized protein [Source:UniProtKB/TrEM BL;Acc:B8B4F2] | 4 | 633-6 52 | target 5' CU-UUACCUUCCUC CGUUCCA 3' | :: : : :::::::::::: | query 3' GAUAUUUUAAGGAGG CAAGGU 5' |
| RI24_ SI24 | osa-miR1 427 | -1.3250 8 | 40.2 5788 | 102. 3705 | BGIOSG A015180 | 3.87 816 | 4044 .965 | 274. 157 | Putative uncharacterized protein [Source:UniProtKB/TrEM BL;Acc:B8ATF3] | 2.5 | 53-73 | target 5' GCGGCACCGCGCG GUUCCGCG 3' | ::: :: ::::.:::::::::. | query 3' CGCGGUGGCGUGCCAA GGCGU 5' |
| RI24_ SI24 | osa-miR1 427 | -1.3250 8 | 40.2 5788 | 102. 3705 | BGIOSG A015185 | 1.14 482 | 770. 8355 | 348. 0602 | Putative uncharacterized protein [Source:UniProtKB/TrEM BL;Acc:A2XSX3] | 4 | 164-1 84 | target 5' GCGUCGCCGCGCG GUUCCCCA 3' | :::.:. ::::.::::::: :: | query 3' CGCGGUGGCGUGCCAA GGCGU 5' |
| RI24_ SI24 | osa-miR1 432 | 2.34673 | 357. 0238 | 69.3 8443 | BGIOSG A013804 | -2.3 1244 | 2.56 0915 | 16.6 8782 | Putative uncharacterized protein [Source:UniProtKB/TrEM BL;Acc:A2XNA2] | 1 | 45-65 | target 5' GUCGGUGUCGUCU CUCCUGAU 3' | :::::: :::.:::::::::::: | query 3' CAGCCACAGUAGAGAG GACUA 5' |
| RI24_ SI24 | osa-miR1 432 | 2.34673 | 357. 0238 | 69.3 8443 | BGIOSG A018749 | -1.4 2081 | 185. 0261 | 497. 0585 | Putative uncharacterized protein [Source:UniProtKB/TrEM | 4 | 1060- 1080 | target 5' GUCGAUCUCAUUU CUCUUGAU 3' | :::: : ::::.::::.:::: | query 3' CAGCCACAGUAGAGAG GACUA 5' |

| | | | | | | | | | | | | | | |
|---|---|---|---|---|---|---|---|---|---|---|---|---|---|---|
| | | | | | | | | | BL;Acc:A2Y0R2] | | | | | |
| RI24_SI24 | osa-miR159a.2 | -1.58837 | 46.61439 | 142.1812 | BGIOSGA007552 | 1.54631 | 1.920686 | 0 | Putative uncharacterized protein [Source:UniProtKB/TrEMBL;Acc:B8AHT0] | 3.5 | 2169-2189 | target 5' UGCUCUUCUUGGGGCAUGCAA 3' | ::: .::.::::::::::::: | query 3' ACGUCGAGGACCCCGUACGUU 5' |
| RI24_SI24 | osa-miR159a.2 | -1.58837 | 46.61439 | 142.1812 | BGIOSGA010571 | 3.16398 | 125.4848 | 13.11186 | Putative uncharacterized protein [Source:UniProtKB/TrEMBL;Acc:B8AQZ1] | 4 | 438-458 | target 5' UGCAGCUCCUGGUGCAUCCAA 3' | :::::: :::::: :::: ::: | query 3' ACGUCGAGGACCCCGUACGUU 5' |
| RI24_SI24 | osa-miR159a.2 | -1.58837 | 46.61439 | 142.1812 | BGIOSGA017480 | 1.83225 | 2.560915 | 0 | Putative uncharacterized protein [Source:UniProtKB/TrEMBL;Acc:B8AX70] | 4 | 191-211 | target 5' UGCCGCUCCUGGGGCUUGCAC 3' | ::: :: ::::::::: :::: | query 3' ACGUCGAGGACCCCGUACGUU 5' |
| RI24_SI24 | osa-miR169f.1 | 1.23957 | 1696.128 | 717.7308 | BGIOSGA026125 | -3.08403 | 108.1987 | 924.9818 | Putative uncharacterized protein [Source:UniProtKB/TrEMBL;Acc:A2YNN2] | 4 | 1046-1066 | target 5' UAGGGAAAUUAUCUUUGGCUA 3' | :::: : : :.:::.::::::: | query 3' AUCCGUUCAGUAGGAACCGAU 5' |
| RI24_SI24 | osa-miR169g | 1.23957 | 1696.128 | 717.7308 | BGIOSGA026125 | -3.08403 | 108.1987 | 924.9818 | Putative uncharacterized protein [Source:UniProtKB/TrEMBL;Acc:A2YNN2] | 4 | 1046-1066 | target 5' UAGGGAAAUUAUCUUUGGCUA 3' | :::: : : :.:::.::::::: | query 3' AUCCGUUCAGUAGGAACCGAU 5' |
| RI24_SI24 | osa-miR1850.1 | 2.97513 | 158.9127 | 19.33665 | BGIOSGA012292 | -1.79406 | 191.4284 | 666.3206 | Putative uncharacterized protein [Source:UniProtKB/TrEMBL;Acc:A2XEV9] | 4 | 472-493 | target 5' CACCAUUUCUCCCAACUUUUCA 3' | : ::: :::::::::::::.:: | query 3' GGGGU-UAGAGGGUUGAAAGGU 5' |
| RI24_SI24 | osa-miR1862f | 1.1001 | 1108.151 | 516.4022 | BGIOSGA003085 | -2.71389 | 8.963203 | 64.36729 | | 3.5 | 1034-1052 | target 5' GCAAGA-AGACCAACCUCAU 3' | :::. : :.:::::::::::: | query 3' GGUUUUAUUUGGUUGGAGUA 5' |
| RI24_SI24 | osa-miR1862f | 1.1001 | 1108.151 | 516.4022 | BGIOSGA004475 | -1.04482 | 0.640229 | 2.383974 | Putative uncharacterized protein [Source:UniProtKB/TrEMBL;Acc:B8A9U2] | 4 | 152-170 | target 5' UCAAGA-AAAUUAACCUCAU 3' | .:::.: :::..:::::::: | query 3' GGUUUUAUUUGGUUGGAGUA 5' |
| RI24_SI24 | osa-miR1862g | 1.1001 | 1108.151 | 516.4022 | BGIOSGA003085 | -2.71389 | 8.963203 | 64.36729 | | 3.5 | 1034-1052 | target 5' GCAAGA-AGACCAACCUCAU 3' | :::. : :.:::::::::::: | query 3' GGUUUUAUUUGGUUGGAGUA 5' |
| RI24_SI24 | osa-miR1862g | 1.1001 | 1108.151 | 516.4022 | BGIOSGA004475 | -1.04482 | 0.640229 | 2.383974 | Putative uncharacterized protein [Source:UniProtKB/TrEMBL;Acc:B8A9U2] | 4 | 152-170 | target 5' UCAAGA-AAAUUAACCUCAU 3' | .:::.: :::..:::::::: | query 3' GGUUUUAUUUGGUUGGAGUA 5' |
| RI24_SI24 | osa-miR1879 | 1.965 | 78.39693 | 19.33665 | BGIOSGA001273 | -1.20487 | 5.12183 | 13.11186 | Putative uncharacterized protein [Source:UniProtKB/TrEMBL;Acc:A2WS92] | 2.5 | 640-662 | target 5' CCACC-CACCCCUAAACCAAACAU 3' | :::: :: ::::::::::::::. | query 3' GGUGGAGUAGGGAUUUGGUUUGUG 5' |
| RI24_SI24 | osa-miR394 | -1.95458 | 91.10994 | 356.0218 | BGIOSGA020470 | 3.18054 | 62.10219 | 5.959934 | Putative uncharacterized protein [Source:UniProtKB/TrEMBL;Acc:B8AX92] | 3 | 1366-1385 | target 5' GGAGUUGGACAGAAUGCAAA 3' | :::: : :::::::::: :: | query 3' CCUCCACCUGUCUUACGGUU 5' |
| RI24_SI24 | osa-miR394 | -1.95458 | 91.10994 | 356.0218 | BGIOSGA025510 | 3.34702 | 81.94928 | 7.151921 | Putative uncharacterized protein [Source:UniProtKB/TrEMBL;Acc:B8B516] | 4 | 20-38 | target 5' GGCGGCGGA-AGAAUGCCAA 3' | :: :: ::: ::::::::: | query 3' CCUCCACCUGUCUUACGGUU 5' |
| RI24_SI24 | osa-miR528 | 2.31463 | 22479.79 | 4517.95 | BGIOSGA024054 | -2.59759 | 7.042517 | 47.67947 | Putative uncharacterized protein [Source:UniProtKB/TrEMBL;Acc:B8B7J3] | 2 | 378-397 | target 5' CUCCUCUGC-UGCCCCUUCCA 3' | :::::: ::: :::::::::: | query 3' GAGGAGACGUACGGGGAAGGU 5' |
| RI24_ | osa-miR5 | 2.31463 | 2247 | 4517 | BGIOSG | -1.9 | 15.3 | 64.3 | Putative uncharacterized | 4 | 61-80 | target 5' | :::::: | query 3' |

| | | | | | | | | | | | | | | |
|---|---|---|---|---|---|---|---|---|---|---|---|---|---|---|
| SI24 | 28 | | 9.79 | .95 | A009946 | 9791 | 6549 | 6729 | protein<br>[Source:UniProtKB/TrEM<br>BL;Acc:A2XL83] | | | CUCCUCUGCGUGC<br>UCC-UCCA 3' | :::.:::.:: :::: | GAGGAGACGUACGGGG<br>AAGGU 5' |
| RI24_<br>SI24 | osa-miR5<br>28 | 2.31463 | 2247<br>9.79 | 4517<br>.95 | BGIOSG<br>A013952 | -4.0<br>4403 | 0 | 15.4<br>9583 | Putative uncharacterized<br>protein<br>[Source:UniProtKB/TrEM<br>BL;Acc:A2XP44] | 4 | 46-65 | target 5'<br>CUCCUCUGCGUGC<br>UCC-UCCA 3' | ::::::<br>:::.:::.:: :::: | query 3'<br>GAGGAGACGUACGGGG<br>AAGGU 5' |
| RI24_<br>SI24 | osa-miR5<br>28 | 2.31463 | 2247<br>9.79 | 4517<br>.95 | BGIOSG<br>A029010 | -1.9<br>2617 | 25.6<br>0915 | 100.<br>1269 | Putative uncharacterized<br>protein<br>[Source:UniProtKB/TrEM<br>BL;Acc:A2YX38] | 4 | 188-2<br>08 | target 5'<br>CGGCUCAUCAUGC<br>CCCUUCCA 3' | : :<br>:: :::::::::::: | query 3'<br>GAGGAGACGUACGGGG<br>AAGGU 5' |
| RI24_<br>SI24 | osa-miR5<br>30-3p | 2.52726 | 680.<br>1463 | 117.<br>1573 | BGIOSG<br>A001350 | -2.1<br>4521 | 1.92<br>0686 | 11.9<br>1987 | Putative uncharacterized<br>protein<br>[Source:UniProtKB/TrEM<br>BL;Acc:A2WRS1] | 4 | 32-52 | target 5'<br>CUUGCACCUGCCUC<br>UGCUCCU 3' | ::::<br>: :::::::::: ::: | query 3'<br>CAACGUAGACGGAGAC<br>GUGGA 5' |
| RI24_<br>SI24 | osa-miR8<br>11d.1 | -3.3587 | 26.4<br>8545 | 280.<br>9501 | BGIOSG<br>A021484 | 1.32<br>236 | 4.48<br>1601 | 1.19<br>1987 | Putative uncharacterized<br>protein<br>[Source:UniProtKB/TrEM<br>BL;Acc:A2YBI7] | 1.5 | 1667-<br>1687 | target 5'<br>UGGACGUCCGAGA<br>UUCGUCCA 3' | :::::.<br>:::::::.:::::: | query 3'<br>ACCUGUAGGCUCUAGG<br>CAGGU 5' |
| RI24_<br>SI24 | osa-miR8<br>11d.1 | -3.3587 | 26.4<br>8545 | 280.<br>9501 | BGIOSG<br>A038081 | 1.75<br>573 | 6.40<br>2288 | 1.19<br>1987 | | 4 | 270-2<br>90 | target 5'<br>GGGACAACCGAGA<br>UUCGUCCU 3' | ::::<br>: :::::::.::::: | query 3'<br>ACCUGUAGGCUCUAGG<br>CAGGU 5' |
| RI24_<br>SI24 | osa-miR8<br>12n-5p | 1.13715 | 48.7<br>3323 | 21.6<br>1155 | BGIOSG<br>A034803 | -1.2<br>3766 | 52.4<br>9876 | 125.<br>1586 | Putative uncharacterized<br>protein<br>[Source:UniProtKB/TrEM<br>BL;Acc:B8BJ46] | 4 | 1572-<br>1595 | target 5'<br>GCACGGAAAAUCA<br>UGUUUGCACUU 3' | ::::::<br>::: ::::: .::::::: | query 3'<br>CGUGCCUUUGAGUACC<br>GACGUGAA 5' |
| RI48_<br>RC00 | osa-miR1<br>846d-5p | 1.57621 | 20.2<br>5999 | 6.12<br>9764 | BGIOSG<br>A016941 | -1.7<br>598 | 20.3<br>2762 | 71.2<br>2614 | Putative uncharacterized<br>protein<br>[Source:UniProtKB/TrEM<br>BL;Acc:B8AT39] | 4 | 268-2<br>89 | target 5'<br>GAGAAGCACCUGC<br>UCGGUGGGA 3' | ::::<br>: :::::::::::: | query 3'<br>CUCUAGGCCGACGAGC<br>CACCCU 5' |
| RI48_<br>RC00 | osa-miR1<br>850.1 | 3.7249 | 683.<br>7746 | 50.7<br>8947 | BGIOSG<br>A012292 | -1.0<br>6649 | 306.<br>478 | 642.<br>9603 | Putative uncharacterized<br>protein<br>[Source:UniProtKB/TrEM<br>BL;Acc:A2XEV9] | 4 | 472-4<br>93 | target 5'<br>CACCAUUUCUCCCA<br>ACUUUUCA 3' | : :::<br>:::::::::::.:: | query 3'<br>GGGGU-UAGAGGGUUG<br>AAAGGU 5' |
| RI48_<br>RC00 | osa-miR1<br>861d | -1.3384<br>8 | 10.1<br>2999 | 27.1<br>461 | BGIOSG<br>A029503 | 2.83<br>276 | 60.9<br>8286 | 7.70<br>0124 | Putative uncharacterized<br>protein<br>[Source:UniProtKB/TrEM<br>BL;Acc:B8BD43] | 3.5 | 209-2<br>31 | target 5'<br>CUCCAGCUUCUGCC<br>UCAAGGCCA 3' | :: :::<br>:.::::::::::.::: | query 3'<br>GA-GUCAAGGACGGAG<br>UUCUGGU 5' |
| RI48_<br>RC00 | osa-miR1<br>861d | -1.3384<br>8 | 10.1<br>2999 | 27.1<br>461 | BGIOSG<br>A017432 | 3.48<br>076 | 10.1<br>6381 | 0 | Putative uncharacterized<br>protein<br>[Source:UniProtKB/TrEM<br>BL;Acc:B8AXI3] | 4 | 491-5<br>12 | target 5'<br>CCCAGUUUCUUCC<br>UCAAGACCG 3' | : ::::<br>:.:: ::::::::::. | query 3'<br>GAGUCAAGGACGGAGU<br>UCUGGU 5' |
| RI48_<br>RC00 | osa-miR1<br>861h | -1.1789<br>3 | 11.8<br>1833 | 28.0<br>2178 | BGIOSG<br>A017432 | 3.48<br>076 | 10.1<br>6381 | 0 | Putative uncharacterized<br>protein<br>[Source:UniProtKB/TrEM<br>BL;Acc:B8AXI3] | 3.5 | 491-5<br>12 | target 5'<br>CCCAGUUUCUUCC<br>UCAAGACCG 3' | : ::::<br>:.:: :::::::::: | query 3'<br>GAGUCAAGGACGGAGU<br>UCUGGC 5' |
| RI48_<br>RC00 | osa-miR1<br>861j | -1.1789<br>3 | 11.8<br>1833 | 28.0<br>2178 | BGIOSG<br>A017432 | 3.48<br>076 | 10.1<br>6381 | 0 | Putative uncharacterized<br>protein<br>[Source:UniProtKB/TrEM<br>BL;Acc:B8AXI3] | 3.5 | 491-5<br>12 | target 5'<br>CCCAGUUUCUUCC<br>UCAAGACCG 3' | : ::::<br>:.:: :::::::::: | query 3'<br>GAGUCAAGGACGGAGU<br>UCUGGC 5' |
| RI48_<br>RI24 | osa-miR1<br>879 | -1.3387<br>8 | 30.3<br>8998 | 78.3<br>9693 | BGIOSG<br>A001273 | 1.74<br>681 | 19.5<br>4579 | 5.12<br>183 | Putative uncharacterized<br>protein<br>[Source:UniProtKB/TrEM<br>BL;Acc:A2WS92] | 2.5 | 640-6<br>62 | target 5'<br>CCACC-CACCCCUA<br>AACCAAACAU 3' | :::::<br>:: ::::::::::::::. | query 3'<br>GGUGGAGUAGGGAUU<br>UGGUUUGUG 5' |
| RI48_<br>RI24 | osa-miR3<br>94 | -1.4056<br>5 | 33.7<br>6665 | 91.1<br>0994 | BGIOSG<br>A029027 | 1.00<br>194 | 94.6<br>0162 | 46.7<br>367 | Putative uncharacterized<br>protein<br>[Source:UniProtKB/TrEM | 4 | 560-5<br>78 | target 5'<br>GGAAGGGGA-AGAA<br>UGCCAA 3' | ::: :<br>::: :::::::::: | query 3'<br>CCUCCACCUGUCUUAC<br>GGUU 5' |

| | | | | | | | | | | | | | | |
|---|---|---|---|---|---|---|---|---|---|---|---|---|---|---|
| | | | | | | | | | BL;Acc:B8B8V2] | | | | | |
| RI48_ RI24 | osa-miR5 150-3p | -1.0637 9 | 25.3 2498 | 54.0 3031 | BGIOSG A007830 | 1.47 468 | 258. 0044 | 92.1 9294 | Putative uncharacterized protein [Source:UniProtKB/TrEM BL;Acc:A2X2N8] | 4 | Dec-3 4 | target 5' GGGCAGC-GGCAGC UGCAGCUUCU 3' | :.:: : :.:::::::::::::: | query 3' CUCGAAGACUGUCGAC GUCGAAGA 5' |
| RI48_ RI24 | osa-miR5 28 | -1.6844 9 | 6993 .072 | 2247 9.79 | BGIOSG A024054 | 1.85 758 | 28.1 4594 | 7.04 2517 | Putative uncharacterized protein [Source:UniProtKB/TrEM BL;Acc:B8B7J3] | 2 | 378-3 97 | target 5' CUCCUCUGC-UGCC CCUUCCA 3' | :::::: ::: :::::::::: | query 3' GAGGAGACGUACGGGG AAGGU 5' |
| RI48_ RI24 | osa-miR5 28 | -1.6844 9 | 6993 .072 | 2247 9.79 | BGIOSG A013952 | 2.50 868 | 4.69 0989 | 0 | Putative uncharacterized protein [Source:UniProtKB/TrEM BL;Acc:A2XP44] | 4 | 46-65 | target 5' CUCCUCUGCGUGC UCC-UCCA 3' | :::::: :::.:::.:: :::: | query 3' GAGGAGACGUACGGGG AAGGU 5' |
| RI48_ RI24 | osa-miR5 28 | -1.6844 9 | 6993 .072 | 2247 9.79 | BGIOSG A029896 | 1.02 592 | 14.0 7297 | 6.40 2288 | Putative uncharacterized protein [Source:UniProtKB/TrEM BL;Acc:B8BEW5] | 4 | 22-41 | target 5' CUCCUCUGCA-GCC UCUUCCU 3' | :::::: :::: :::.:::: | query 3' GAGGAGACGUACGGGG AAGGU 5' |
| RI48_ SI48 | osa-miR1 320 | 2.39571 | 400. 1347 | 75.2 2735 | BGIOSG A014361 | -1.3 3284 | 26.5 8227 | 68.4 791 | Putative uncharacterized protein [Source:UniProtKB/TrEM BL;Acc:B8AU51] | 3.5 | 175-1 95 | target 5' CCGCAUAAUUCCU CCGUUCCA 3' | : . : :::::::::::::: | query 3' GAUAUUUUAAGGAGG CAAGGU 5' |
| RI48_ SI48 | osa-miR1 59a.2 | -2.0542 7 | 27.0 1332 | 115. 3486 | BGIOSG A010571 | 1.31 381 | 35.9 6425 | 13.8 6919 | Putative uncharacterized protein [Source:UniProtKB/TrEM BL;Acc:B8AQZ1] | 4 | 438-4 58 | target 5' UGCAGCUCCUGGU GCAUCCAA 3' | :::::: :::::: :::: ::: | query 3' ACGUCGAGGACCCCGU ACGUU 5' |
| RI48_ SI48 | osa-miR2 864.2 | 6.84841 | 2714 .838 | 22.5 6821 | BGIOSG A007922 | -1.0 1483 | 0.78 1832 | 2.60 0472 | Putative uncharacterized protein [Source:UniProtKB/TrEM BL;Acc:A2X370] | 2 | 565-5 86 | target 5' UACCUAUACAAUG ACAAAACAA 3' | :::::: ::::::: ::::::: | query 3' AUGGAUAUGUUAC-GU UUUGUU 5' |
| RI48_ SI48 | osa-miR3 94 | -1.6295 3 | 33.7 6665 | 106. 5721 | BGIOSG A020470 | 1.41 487 | 8.60 0147 | 2.60 0472 | Putative uncharacterized protein [Source:UniProtKB/TrEM BL;Acc:B8AX92] | 3 | 1366- 1385 | target 5' GGAGUUGGACAGA AUGCAAA 3' | :::: : :::::::::: :: | query 3' CCUCCACCUGUCUUAC GGUU 5' |
| RI48_ SI48 | osa-miR3 94 | -1.6295 3 | 33.7 6665 | 106. 5721 | BGIOSG A025510 | 1.24 346 | 38.3 0975 | 15.6 0283 | Putative uncharacterized protein [Source:UniProtKB/TrEM BL;Acc:B8B516] | 4 | 20-38 | target 5' GGCGGCGGA-AGAA UGCCAA 3' | :: :: ::: ::::::::: | query 3' CCUCCACCUGUCUUAC GGUU 5' |
| RI48_ SI48 | osa-miR5 077 | -1.0670 1 | 7761 .263 | 1626 1.65 | BGIOSG A025118 | 1.02 038 | 111. 8019 | 54.6 0992 | Putative uncharacterized protein [Source:UniProtKB/TrEM BL;Acc:B8B742] | 4 | 3050- 3068 | target 5' UAGUGAACCCGAU GCUAAC 3' | : :::: ::::::.:: ::: | query 3' ACCACUUGGGCUGCGC UUG 5' |
| RI48_ SI48 | osa-miR5 161 | 2.04817 | 13.5 0666 | 2.50 7578 | BGIOSG A015421 | -1.5 9932 | 15.6 3663 | 49.4 0897 | Putative uncharacterized protein [Source:UniProtKB/TrEM BL;Acc:B8ARX1] | 3.5 | 187-2 06 | target 5' UAUGGCCCCUCUG AUCCAGC 3' | :::. :::::::::::: | query 3' AUAUGAGGGAGACUA GGUCU 5' |
| SC08_ SC00 | osa-miR1 318 | 1.5033 | 36.3 8101 | 12.1 8595 | BGIOSG A013804 | -1.9 3089 | 3.30 5852 | 15.4 1783 | Putative uncharacterized protein [Source:UniProtKB/TrEM BL;Acc:A2XNA2] | 1 | 45-64 | target 5' GUCGGUGUCGUCU CUCCUGA 3' | :::::: :::.:::::::::: | query 3' CAGCCACAGUAGAGAG GACU 5' |
| SC08_ SC00 | osa-miR1 318 | 1.5033 | 36.3 8101 | 12.1 8595 | BGIOSG A018749 | -2.0 8722 | 100. 8285 | 431. 6992 | Putative uncharacterized protein [Source:UniProtKB/TrEM BL;Acc:A2Y0R2] | 4 | 1060- 1079 | target 5' GUCGAUCUCAUUU CUCUUGA 3' | :::: : ::::.::::.::: | query 3' CAGCCACAGUAGAGAG GACU 5' |
| SC08_ SC00 | osa-miR1 432 | 1.54751 | 54.1 6728 | 17.8 7273 | BGIOSG A013804 | -1.9 3089 | 3.30 5852 | 15.4 1783 | Putative uncharacterized protein [Source:UniProtKB/TrEM | 1 | 45-65 | target 5' GUCGGUGUCGUCU CUCCUGAU 3' | :::::: :::.:::::::::::: | query 3' CAGCCACAGUAGAGAG GACUA 5' |

| | | | | | | | | | | | | | | |
|---|---|---|---|---|---|---|---|---|---|---|---|---|---|---|
| | | | | | | | | | BL;Acc:A2XNA2] | | | | | |
| SC08_ SC00 | osa-miR1 432 | 1.54751 | 54.1 6728 | 17.8 7273 | BGIOSG A018749 | -2.0 8722 | 100. 8285 | 431. 6992 | Putative uncharacterized protein [Source:UniProtKB/TrEM BL;Acc:A2Y0R2] | 4 | 1060- 1080 | target 5' GUCGAUCUCAUUU CUCUUGAU 3' | :::: : ::::.::::.:::: | query 3' CAGCCACAGUAGAGAG GACUA 5' |
| SC08_ SC00 | osa-miR1 56a | 1.03851 | 2312 86.2 | 1125 96.6 | BGIOSG A005075 | -1.3 927 | 41.3 2315 | 110. 1274 | Putative uncharacterized protein [Source:UniProtKB/TrEM BL;Acc:A2WYH5] | 1 | 923-9 42 | target 5' GUGCUCUCUCUCU UCUGUCA 3' | :::::: ::::::::::::: | query 3' CACGAGUGAGAGAAGA CAGU 5' |
| SC08_ SC00 | osa-miR1 56a | 1.03851 | 2312 86.2 | 1125 96.6 | BGIOSG A029541 | -1.5 8003 | 7.43 8168 | 24.2 2802 | Putative uncharacterized protein [Source:UniProtKB/TrEM BL;Acc:B8BD46] | 1 | 578-5 97 | target 5' GUGCUCUCUCUCU UCUGUCA 3' | :::::: ::::::::::::: | query 3' CACGAGUGAGAGAAGA CAGU 5' |
| SC08_ SC00 | osa-miR1 56a | 1.03851 | 2312 86.2 | 1125 96.6 | BGIOSG A007643 | -1.6 8946 | 31.4 056 | 103. 5197 | Putative uncharacterized protein [Source:UniProtKB/TrEM BL;Acc:A2X1G6] | 1 | 716-7 35 | target 5' GUGCUCUCUCUCU UCUGUCA 3' | :::::: ::::::::::::: | query 3' CACGAGUGAGAGAAGA CAGU 5' |
| SC08_ SC00 | osa-miR1 56a | 1.03851 | 2312 86.2 | 1125 96.6 | BGIOSG A036252 | -3.3 4976 | 0.82 6463 | 17.6 2038 | Putative uncharacterized protein [Source:UniProtKB/TrEM BL;Acc:A2ZK57] | 4 | 232-2 50 | target 5' GUGCUCACUCUC-U CUGGCA 3' | :::::: :::::: :::: :: | query 3' CACGAGUGAGAGAAGA CAGU 5' |
| SC08_ SC00 | osa-miR1 56a | 1.03851 | 2312 86.2 | 1125 96.6 | BGIOSG A036253 | -3.8 2937 | 0 | 13.2 1528 | Putative uncharacterized protein [Source:UniProtKB/TrEM BL;Acc:A2ZK56] | 4 | 232-2 50 | target 5' GUGCUCACUCUC-U CUGGCA 3' | :::::: :::::: :::: :: | query 3' CACGAGUGAGAGAAGA CAGU 5' |
| SC08_ SC00 | osa-miR1 56b | 1.03851 | 2312 86.2 | 1125 96.6 | BGIOSG A005075 | -1.3 927 | 41.3 2315 | 110. 1274 | Putative uncharacterized protein [Source:UniProtKB/TrEM BL;Acc:A2WYH5] | 1 | 923-9 42 | target 5' GUGCUCUCUCUCU UCUGUCA 3' | :::::: ::::::::::::: | query 3' CACGAGUGAGAGAAGA CAGU 5' |
| SC08_ SC00 | osa-miR1 56b | 1.03851 | 2312 86.2 | 1125 96.6 | BGIOSG A029541 | -1.5 8003 | 7.43 8168 | 24.2 2802 | Putative uncharacterized protein [Source:UniProtKB/TrEM BL;Acc:B8BD46] | 1 | 578-5 97 | target 5' GUGCUCUCUCUCU UCUGUCA 3' | :::::: ::::::::::::: | query 3' CACGAGUGAGAGAAGA CAGU 5' |
| SC08_ SC00 | osa-miR1 56b | 1.03851 | 2312 86.2 | 1125 96.6 | BGIOSG A007643 | -1.6 8946 | 31.4 056 | 103. 5197 | Putative uncharacterized protein [Source:UniProtKB/TrEM BL;Acc:A2X1G6] | 1 | 716-7 35 | target 5' GUGCUCUCUCUCU UCUGUCA 3' | :::::: ::::::::::::: | query 3' CACGAGUGAGAGAAGA CAGU 5' |
| SC08_ SC00 | osa-miR1 56b | 1.03851 | 2312 86.2 | 1125 96.6 | BGIOSG A036252 | -3.3 4976 | 0.82 6463 | 17.6 2038 | Putative uncharacterized protein [Source:UniProtKB/TrEM BL;Acc:A2ZK57] | 4 | 232-2 50 | target 5' GUGCUCACUCUC-U CUGGCA 3' | :::::: :::::: :::: :: | query 3' CACGAGUGAGAGAAGA CAGU 5' |
| SC08_ SC00 | osa-miR1 56b | 1.03851 | 2312 86.2 | 1125 96.6 | BGIOSG A036253 | -3.8 2937 | 0 | 13.2 1528 | Putative uncharacterized protein [Source:UniProtKB/TrEM BL;Acc:A2ZK56] | 4 | 232-2 50 | target 5' GUGCUCACUCUC-U CUGGCA 3' | :::::: :::::: :::: :: | query 3' CACGAGUGAGAGAAGA CAGU 5' |
| SC08_ SC00 | osa-miR1 56c | 1.03851 | 2312 86.2 | 1125 96.6 | BGIOSG A005075 | -1.3 927 | 41.3 2315 | 110. 1274 | Putative uncharacterized protein [Source:UniProtKB/TrEM BL;Acc:A2WYH5] | 1 | 923-9 42 | target 5' GUGCUCUCUCUCU UCUGUCA 3' | :::::: ::::::::::::: | query 3' CACGAGUGAGAGAAGA CAGU 5' |
| SC08_ SC00 | osa-miR1 56c | 1.03851 | 2312 86.2 | 1125 96.6 | BGIOSG A029541 | -1.5 8003 | 7.43 8168 | 24.2 2802 | Putative uncharacterized protein [Source:UniProtKB/TrEM BL;Acc:B8BD46] | 1 | 578-5 97 | target 5' GUGCUCUCUCUCU UCUGUCA 3' | :::::: ::::::::::::: | query 3' CACGAGUGAGAGAAGA CAGU 5' |
| SC08_ SC00 | osa-miR1 56c | 1.03851 | 2312 86.2 | 1125 96.6 | BGIOSG A007643 | -1.6 8946 | 31.4 056 | 103. 5197 | Putative uncharacterized protein [Source:UniProtKB/TrEM | 1 | 716-7 35 | target 5' GUGCUCUCUCUCU UCUGUCA 3' | :::::: ::::::::::::: | query 3' CACGAGUGAGAGAAGA CAGU 5' |

| | | | | | | | | | | | | | | |
|---|---|---|---|---|---|---|---|---|---|---|---|---|---|---|
| | | | | | | | | | BL;Acc:A2X1G6] | | | | | |
| SC08_<br>SC00 | osa-miR1<br>56c | 1.03851 | 2312<br>86.2 | 1125<br>96.6 | BGIOSG<br>A036252 | -3.3<br>4976 | 0.82<br>6463 | 17.6<br>2038 | Putative uncharacterized protein [Source:UniProtKB/TrEM<br>BL;Acc:A2ZK57] | 4 | 232-2<br>50 | target 5'<br>GUGCUCACUCUC-U<br>CUGGCA 3' | ::::::<br>:::::: :::: :: | query 3'<br>CACGAGUGAGAGAAGA<br>CAGU 5' |
| SC08_<br>SC00 | osa-miR1<br>56c | 1.03851 | 2312<br>86.2 | 1125<br>96.6 | BGIOSG<br>A036253 | -3.8<br>2937 | 0 | 13.2<br>1528 | Putative uncharacterized protein [Source:UniProtKB/TrEM<br>BL;Acc:A2ZK56] | 4 | 232-2<br>50 | target 5'<br>GUGCUCACUCUC-U<br>CUGGCA 3' | ::::::<br>:::::: :::: :: | query 3'<br>CACGAGUGAGAGAAGA<br>CAGU 5' |
| SC08_<br>SC00 | osa-miR1<br>56d | 1.03851 | 2312<br>86.2 | 1125<br>96.6 | BGIOSG<br>A005075 | -1.3<br>927 | 41.3<br>2315 | 110.<br>1274 | Putative uncharacterized protein [Source:UniProtKB/TrEM<br>BL;Acc:A2WYH5] | 1 | 923-9<br>42 | target 5'<br>GUGCUCUCUCUCU<br>UCUGUCA 3' | ::::::<br>::::::::::::: | query 3'<br>CACGAGUGAGAGAAGA<br>CAGU 5' |
| SC08_<br>SC00 | osa-miR1<br>56d | 1.03851 | 2312<br>86.2 | 1125<br>96.6 | BGIOSG<br>A029541 | -1.5<br>8003 | 7.43<br>8168 | 24.2<br>2802 | Putative uncharacterized protein [Source:UniProtKB/TrEM<br>BL;Acc:B8BD46] | 1 | 578-5<br>97 | target 5'<br>GUGCUCUCUCUCU<br>UCUGUCA 3' | ::::::<br>::::::::::::: | query 3'<br>CACGAGUGAGAGAAGA<br>CAGU 5' |
| SC08_<br>SC00 | osa-miR1<br>56d | 1.03851 | 2312<br>86.2 | 1125<br>96.6 | BGIOSG<br>A007643 | -1.6<br>8946 | 31.4<br>056 | 103.<br>5197 | Putative uncharacterized protein [Source:UniProtKB/TrEM<br>BL;Acc:A2X1G6] | 1 | 716-7<br>35 | target 5'<br>GUGCUCUCUCUCU<br>UCUGUCA 3' | ::::::<br>::::::::::::: | query 3'<br>CACGAGUGAGAGAAGA<br>CAGU 5' |
| SC08_<br>SC00 | osa-miR1<br>56d | 1.03851 | 2312<br>86.2 | 1125<br>96.6 | BGIOSG<br>A036252 | -3.3<br>4976 | 0.82<br>6463 | 17.6<br>2038 | Putative uncharacterized protein [Source:UniProtKB/TrEM<br>BL;Acc:A2ZK57] | 4 | 232-2<br>50 | target 5'<br>GUGCUCACUCUC-U<br>CUGGCA 3' | ::::::<br>:::::: :::: :: | query 3'<br>CACGAGUGAGAGAAGA<br>CAGU 5' |
| SC08_<br>SC00 | osa-miR1<br>56d | 1.03851 | 2312<br>86.2 | 1125<br>96.6 | BGIOSG<br>A036253 | -3.8<br>2937 | 0 | 13.2<br>1528 | Putative uncharacterized protein [Source:UniProtKB/TrEM<br>BL;Acc:A2ZK56] | 4 | 232-2<br>50 | target 5'<br>GUGCUCACUCUC-U<br>CUGGCA 3' | ::::::<br>:::::: :::: :: | query 3'<br>CACGAGUGAGAGAAGA<br>CAGU 5' |
| SC08_<br>SC00 | osa-miR1<br>56e | 1.03851 | 2312<br>86.2 | 1125<br>96.6 | BGIOSG<br>A005075 | -1.3<br>927 | 41.3<br>2315 | 110.<br>1274 | Putative uncharacterized protein [Source:UniProtKB/TrEM<br>BL;Acc:A2WYH5] | 1 | 923-9<br>42 | target 5'<br>GUGCUCUCUCUCU<br>UCUGUCA 3' | ::::::<br>::::::::::::: | query 3'<br>CACGAGUGAGAGAAGA<br>CAGU 5' |
| SC08_<br>SC00 | osa-miR1<br>56e | 1.03851 | 2312<br>86.2 | 1125<br>96.6 | BGIOSG<br>A029541 | -1.5<br>8003 | 7.43<br>8168 | 24.2<br>2802 | Putative uncharacterized protein [Source:UniProtKB/TrEM<br>BL;Acc:B8BD46] | 1 | 578-5<br>97 | target 5'<br>GUGCUCUCUCUCU<br>UCUGUCA 3' | ::::::<br>::::::::::::: | query 3'<br>CACGAGUGAGAGAAGA<br>CAGU 5' |
| SC08_<br>SC00 | osa-miR1<br>56e | 1.03851 | 2312<br>86.2 | 1125<br>96.6 | BGIOSG<br>A007643 | -1.6<br>8946 | 31.4<br>056 | 103.<br>5197 | Putative uncharacterized protein [Source:UniProtKB/TrEM<br>BL;Acc:A2X1G6] | 1 | 716-7<br>35 | target 5'<br>GUGCUCUCUCUCU<br>UCUGUCA 3' | ::::::<br>::::::::::::: | query 3'<br>CACGAGUGAGAGAAGA<br>CAGU 5' |
| SC08_<br>SC00 | osa-miR1<br>56e | 1.03851 | 2312<br>86.2 | 1125<br>96.6 | BGIOSG<br>A036252 | -3.3<br>4976 | 0.82<br>6463 | 17.6<br>2038 | Putative uncharacterized protein [Source:UniProtKB/TrEM<br>BL;Acc:A2ZK57] | 4 | 232-2<br>50 | target 5'<br>GUGCUCACUCUC-U<br>CUGGCA 3' | ::::::<br>:::::: :::: :: | query 3'<br>CACGAGUGAGAGAAGA<br>CAGU 5' |
| SC08_<br>SC00 | osa-miR1<br>56e | 1.03851 | 2312<br>86.2 | 1125<br>96.6 | BGIOSG<br>A036253 | -3.8<br>2937 | 0 | 13.2<br>1528 | Putative uncharacterized protein [Source:UniProtKB/TrEM<br>BL;Acc:A2ZK56] | 4 | 232-2<br>50 | target 5'<br>GUGCUCACUCUC-U<br>CUGGCA 3' | ::::::<br>:::::: :::: :: | query 3'<br>CACGAGUGAGAGAAGA<br>CAGU 5' |
| SC08_<br>SC00 | osa-miR1<br>56f | 1.03851 | 2312<br>86.2 | 1125<br>96.6 | BGIOSG<br>A005075 | -1.3<br>927 | 41.3<br>2315 | 110.<br>1274 | Putative uncharacterized protein [Source:UniProtKB/TrEM<br>BL;Acc:A2WYH5] | 1 | 923-9<br>42 | target 5'<br>GUGCUCUCUCUCU<br>UCUGUCA 3' | ::::::<br>::::::::::::: | query 3'<br>CACGAGUGAGAGAAGA<br>CAGU 5' |
| SC08_<br>SC00 | osa-miR1<br>56f | 1.03851 | 2312<br>86.2 | 1125<br>96.6 | BGIOSG<br>A029541 | -1.5<br>8003 | 7.43<br>8168 | 24.2<br>2802 | Putative uncharacterized protein [Source:UniProtKB/TrEM | 1 | 578-5<br>97 | target 5'<br>GUGCUCUCUCUCU<br>UCUGUCA 3' | ::::::<br>::::::::::::: | query 3'<br>CACGAGUGAGAGAAGA<br>CAGU 5' |

| | | | | | | | | | | | | | | |
|---|---|---|---|---|---|---|---|---|---|---|---|---|---|---|
| | | | | | | | | | BL;Acc:B8BD46] | | | | | |
| SC08_ SC00 | osa-miR156f | 1.03851 | 231286.2 | 112596.6 | BGIOSGA007643 | -1.68946 | 31.4056 | 103.5197 | Putative uncharacterized protein [Source:UniProtKB/TrEMBL;Acc:A2X1G6] | 1 | 716-735 | target 5' GUGCUCUCUCUCUUCUGUCA 3' | :::::: ::::::::::::: | query 3' CACGAGUGAGAGAAGACAGU 5' |
| SC08_ SC00 | osa-miR156f | 1.03851 | 231286.2 | 112596.6 | BGIOSGA036252 | -3.34976 | 0.826463 | 17.62038 | Putative uncharacterized protein [Source:UniProtKB/TrEMBL;Acc:A2ZK57] | 4 | 232-250 | target 5' GUGCUCACUCUC-UCUGGCA 3' | :::::: :::::: :::: :: | query 3' CACGAGUGAGAGAAGACAGU 5' |
| SC08_ SC00 | osa-miR156f | 1.03851 | 231286.2 | 112596.6 | BGIOSGA036253 | -3.82937 | 0 | 13.21528 | Putative uncharacterized protein [Source:UniProtKB/TrEMBL;Acc:A2ZK56] | 4 | 232-250 | target 5' GUGCUCACUCUC-UCUGGCA 3' | :::::: :::::: :::: :: | query 3' CACGAGUGAGAGAAGACAGU 5' |
| SC08_ SC00 | osa-miR156g | 1.03851 | 231286.2 | 112596.6 | BGIOSGA005075 | -1.3927 | 41.32315 | 110.1274 | Putative uncharacterized protein [Source:UniProtKB/TrEMBL;Acc:A2WYH5] | 1 | 923-942 | target 5' GUGCUCUCUCUCUUCUGUCA 3' | :::::: ::::::::::::: | query 3' CACGAGUGAGAGAAGACAGU 5' |
| SC08_ SC00 | osa-miR156g | 1.03851 | 231286.2 | 112596.6 | BGIOSGA029541 | -1.58003 | 7.438168 | 24.22802 | Putative uncharacterized protein [Source:UniProtKB/TrEMBL;Acc:B8BD46] | 1 | 578-597 | target 5' GUGCUCUCUCUCUUCUGUCA 3' | :::::: ::::::::::::: | query 3' CACGAGUGAGAGAAGACAGU 5' |
| SC08_ SC00 | osa-miR156g | 1.03851 | 231286.2 | 112596.6 | BGIOSGA007643 | -1.68946 | 31.4056 | 103.5197 | Putative uncharacterized protein [Source:UniProtKB/TrEMBL;Acc:A2X1G6] | 1 | 716-735 | target 5' GUGCUCUCUCUCUUCUGUCA 3' | :::::: ::::::::::::: | query 3' CACGAGUGAGAGAAGACAGU 5' |
| SC08_ SC00 | osa-miR156g | 1.03851 | 231286.2 | 112596.6 | BGIOSGA036252 | -3.34976 | 0.826463 | 17.62038 | Putative uncharacterized protein [Source:UniProtKB/TrEMBL;Acc:A2ZK57] | 4 | 232-250 | target 5' GUGCUCACUCUC-UCUGGCA 3' | :::::: :::::: :::: :: | query 3' CACGAGUGAGAGAAGACAGU 5' |
| SC08_ SC00 | osa-miR156g | 1.03851 | 231286.2 | 112596.6 | BGIOSGA036253 | -3.82937 | 0 | 13.21528 | Putative uncharacterized protein [Source:UniProtKB/TrEMBL;Acc:A2ZK56] | 4 | 232-250 | target 5' GUGCUCACUCUC-UCUGGCA 3' | :::::: :::::: :::: :: | query 3' CACGAGUGAGAGAAGACAGU 5' |
| SC08_ SC00 | osa-miR156h | 1.03851 | 231286.2 | 112596.6 | BGIOSGA005075 | -1.3927 | 41.32315 | 110.1274 | Putative uncharacterized protein [Source:UniProtKB/TrEMBL;Acc:A2WYH5] | 1 | 923-942 | target 5' GUGCUCUCUCUCUUCUGUCA 3' | :::::: ::::::::::::: | query 3' CACGAGUGAGAGAAGACAGU 5' |
| SC08_ SC00 | osa-miR156h | 1.03851 | 231286.2 | 112596.6 | BGIOSGA029541 | -1.58003 | 7.438168 | 24.22802 | Putative uncharacterized protein [Source:UniProtKB/TrEMBL;Acc:B8BD46] | 1 | 578-597 | target 5' GUGCUCUCUCUCUUCUGUCA 3' | :::::: ::::::::::::: | query 3' CACGAGUGAGAGAAGACAGU 5' |
| SC08_ SC00 | osa-miR156h | 1.03851 | 231286.2 | 112596.6 | BGIOSGA007643 | -1.68946 | 31.4056 | 103.5197 | Putative uncharacterized protein [Source:UniProtKB/TrEMBL;Acc:A2X1G6] | 1 | 716-735 | target 5' GUGCUCUCUCUCUUCUGUCA 3' | :::::: ::::::::::::: | query 3' CACGAGUGAGAGAAGACAGU 5' |
| SC08_ SC00 | osa-miR156h | 1.03851 | 231286.2 | 112596.6 | BGIOSGA036252 | -3.34976 | 0.826463 | 17.62038 | Putative uncharacterized protein [Source:UniProtKB/TrEMBL;Acc:A2ZK57] | 4 | 232-250 | target 5' GUGCUCACUCUC-UCUGGCA 3' | :::::: :::::: :::: :: | query 3' CACGAGUGAGAGAAGACAGU 5' |
| SC08_ SC00 | osa-miR156h | 1.03851 | 231286.2 | 112596.6 | BGIOSGA036253 | -3.82937 | 0 | 13.21528 | Putative uncharacterized protein [Source:UniProtKB/TrEMBL;Acc:A2ZK56] | 4 | 232-250 | target 5' GUGCUCACUCUC-UCUGGCA 3' | :::::: :::::: :::: :: | query 3' CACGAGUGAGAGAAGACAGU 5' |
| SC08_ SC00 | osa-miR156i | 1.03851 | 231286.2 | 112596.6 | BGIOSGA005075 | -1.3927 | 41.32315 | 110.1274 | Putative uncharacterized protein [Source:UniProtKB/TrEM | 1 | 923-942 | target 5' GUGCUCUCUCUCUUCUGUCA 3' | :::::: ::::::::::::: | query 3' CACGAGUGAGAGAAGACAGU 5' |

| | | | | | | | | | | | | | | |
|---|---|---|---|---|---|---|---|---|---|---|---|---|---|---|
| | | | | | | | | | BL;Acc:A2WYH5] | | | | | |
| SC08_ SC00 | osa-miR1 56i | 1.03851 | 2312 86.2 | 1125 96.6 | BGIOSG A029541 | -1.5 8003 | 7.43 8168 | 24.2 2802 | Putative uncharacterized protein [Source:UniProtKB/TrEM BL;Acc:B8BD46] | 1 | 578-5 97 | target 5' GUGCUCUCUCUCU UCUGUCA 3' | :::::: :::::::::::::: | query 3' CACGAGUGAGAGAAGA CAGU 5' |
| SC08_ SC00 | osa-miR1 56i | 1.03851 | 2312 86.2 | 1125 96.6 | BGIOSG A007643 | -1.6 8946 | 31.4 056 | 103. 5197 | Putative uncharacterized protein [Source:UniProtKB/TrEM BL;Acc:A2X1G6] | 1 | 716-7 35 | target 5' GUGCUCUCUCUCU UCUGUCA 3' | :::::: :::::::::::::: | query 3' CACGAGUGAGAGAAGA CAGU 5' |
| SC08_ SC00 | osa-miR1 56i | 1.03851 | 2312 86.2 | 1125 96.6 | BGIOSG A036252 | -3.3 4976 | 0.82 6463 | 17.6 2038 | Putative uncharacterized protein [Source:UniProtKB/TrEM BL;Acc:A2ZK57] | 4 | 232-2 50 | target 5' GUGCUCACUCUC-U CUGGCA 3' | :::::: :::::: :::: :: | query 3' CACGAGUGAGAGAAGA CAGU 5' |
| SC08_ SC00 | osa-miR1 56i | 1.03851 | 2312 86.2 | 1125 96.6 | BGIOSG A036253 | -3.8 2937 | 0 | 13.2 1528 | Putative uncharacterized protein [Source:UniProtKB/TrEM BL;Acc:A2ZK56] | 4 | 232-2 50 | target 5' GUGCUCACUCUC-U CUGGCA 3' | :::::: :::::: :::: :: | query 3' CACGAGUGAGAGAAGA CAGU 5' |
| SC08_ SC00 | osa-miR1 56j | 1.03851 | 2312 86.2 | 1125 96.6 | BGIOSG A005075 | -1.3 927 | 41.3 2315 | 110. 1274 | Putative uncharacterized protein [Source:UniProtKB/TrEM BL;Acc:A2WYH5] | 1 | 923-9 42 | target 5' GUGCUCUCUCUCU UCUGUCA 3' | :::::: :::::::::::::: | query 3' CACGAGUGAGAGAAGA CAGU 5' |
| SC08_ SC00 | osa-miR1 56j | 1.03851 | 2312 86.2 | 1125 96.6 | BGIOSG A029541 | -1.5 8003 | 7.43 8168 | 24.2 2802 | Putative uncharacterized protein [Source:UniProtKB/TrEM BL;Acc:B8BD46] | 1 | 578-5 97 | target 5' GUGCUCUCUCUCU UCUGUCA 3' | :::::: :::::::::::::: | query 3' CACGAGUGAGAGAAGA CAGU 5' |
| SC08_ SC00 | osa-miR1 56j | 1.03851 | 2312 86.2 | 1125 96.6 | BGIOSG A007643 | -1.6 8946 | 31.4 056 | 103. 5197 | Putative uncharacterized protein [Source:UniProtKB/TrEM BL;Acc:A2X1G6] | 1 | 716-7 35 | target 5' GUGCUCUCUCUCU UCUGUCA 3' | :::::: :::::::::::::: | query 3' CACGAGUGAGAGAAGA CAGU 5' |
| SC08_ SC00 | osa-miR1 56j | 1.03851 | 2312 86.2 | 1125 96.6 | BGIOSG A036252 | -3.3 4976 | 0.82 6463 | 17.6 2038 | Putative uncharacterized protein [Source:UniProtKB/TrEM BL;Acc:A2ZK57] | 4 | 232-2 50 | target 5' GUGCUCACUCUC-U CUGGCA 3' | :::::: :::::: :::: :: | query 3' CACGAGUGAGAGAAGA CAGU 5' |
| SC08_ SC00 | osa-miR1 56j | 1.03851 | 2312 86.2 | 1125 96.6 | BGIOSG A036253 | -3.8 2937 | 0 | 13.2 1528 | Putative uncharacterized protein [Source:UniProtKB/TrEM BL;Acc:A2ZK56] | 4 | 232-2 50 | target 5' GUGCUCACUCUC-U CUGGCA 3' | :::::: :::::: :::: :: | query 3' CACGAGUGAGAGAAGA CAGU 5' |
| SC08_ SC00 | osa-miR1 56k | 1.03956 | 2279 68.2 | 1109 00.3 | BGIOSG A005075 | -1.3 927 | 41.3 2315 | 110. 1274 | Putative uncharacterized protein [Source:UniProtKB/TrEM BL;Acc:A2WYH5] | 0 | 922-9 42 | target 5' UGUGCUCUCUCUC UUCUGUCA 3' | :::::: ::::::::::::::: | query 3' ACACGAGAGAGAGAAG ACAGU 5' |
| SC08_ SC00 | osa-miR1 56k | 1.03956 | 2279 68.2 | 1109 00.3 | BGIOSG A029541 | -1.5 8003 | 7.43 8168 | 24.2 2802 | Putative uncharacterized protein [Source:UniProtKB/TrEM BL;Acc:B8BD46] | 0 | 577-5 97 | target 5' UGUGCUCUCUCUC UUCUGUCA 3' | :::::: ::::::::::::::: | query 3' ACACGAGAGAGAGAAG ACAGU 5' |
| SC08_ SC00 | osa-miR1 56k | 1.03956 | 2279 68.2 | 1109 00.3 | BGIOSG A007643 | -1.6 8946 | 31.4 056 | 103. 5197 | Putative uncharacterized protein [Source:UniProtKB/TrEM BL;Acc:A2X1G6] | 1 | 715-7 35 | target 5' CGUGCUCUCUCUC UUCUGUCA 3' | :::: :::::::::::::::: | query 3' ACACGAGAGAGAGAAG ACAGU 5' |
| SC08_ SC00 | osa-miR1 66a | -1.0963 5 | 9345 8.77 | 1998 28.5 | BGIOSG A002254 | 1.40 758 | 1.65 2926 | 0 | Putative uncharacterized protein [Source:UniProtKB/TrEM BL;Acc:B8ADK5] | 3.5 | 565-5 86 | target 5' GGGGAUCUGAAGU CUGGUCCGG 3' | ::::: :::::.:::::::. | query 3' CCCCU-UACUUCGGACC AGGCU 5' |
| SC08_ SC00 | osa-miR1 66b | -1.0963 5 | 9345 8.77 | 1998 28.5 | BGIOSG A002254 | 1.40 758 | 1.65 2926 | 0 | Putative uncharacterized protein [Source:UniProtKB/TrEM | 3.5 | 565-5 86 | target 5' GGGGAUCUGAAGU CUGGUCCGG 3' | ::::: :::::.:::::::. | query 3' CCCCU-UACUUCGGACC AGGCU 5' |

| | | | | | | | | | | | | | | |
|---|---|---|---|---|---|---|---|---|---|---|---|---|---|---|
| | | | | | | | | | BL;Acc:B8ADK5] | | | | | |
| SC08_<br>SC00 | osa-miR1<br>66c | -1.0963<br>5 | 9345<br>8.77 | 1998<br>28.5 | BGIOSG<br>A002254 | 1.40<br>758 | 1.65<br>2926 | 0 | Putative uncharacterized protein [Source:UniProtKB/TrEMBL;Acc:B8ADK5] | 3.5 | 565-5<br>86 | target 5'<br>GGGGAUCUGAAGU<br>CUGGUCCGG 3' | :::::<br>:::::.:::::::::. | query 3'<br>CCCCU-UACUUCGGACC<br>AGGCU 5' |
| SC08_<br>SC00 | osa-miR1<br>66d | -1.0963<br>5 | 9345<br>8.77 | 1998<br>28.5 | BGIOSG<br>A002254 | 1.40<br>758 | 1.65<br>2926 | 0 | Putative uncharacterized protein [Source:UniProtKB/TrEMBL;Acc:B8ADK5] | 3.5 | 565-5<br>86 | target 5'<br>GGGGAUCUGAAGU<br>CUGGUCCGG 3' | :::::<br>:::::.:::::::::. | query 3'<br>CCCCU-UACUUCGGACC<br>AGGCU 5' |
| SC08_<br>SC00 | osa-miR1<br>66f | -1.0963<br>5 | 9345<br>8.77 | 1998<br>28.5 | BGIOSG<br>A002254 | 1.40<br>758 | 1.65<br>2926 | 0 | Putative uncharacterized protein [Source:UniProtKB/TrEMBL;Acc:B8ADK5] | 3.5 | 565-5<br>86 | target 5'<br>GGGGAUCUGAAGU<br>CUGGUCCGG 3' | :::::<br>:::::.:::::::::. | query 3'<br>CCCCU-UACUUCGGACC<br>AGGCU 5' |
| SC08_<br>SC00 | osa-miR1<br>66g | -1.0939<br>5 | 8917<br>6.32 | 1903<br>55.2 | BGIOSG<br>A002254 | 1.40<br>758 | 1.65<br>2926 | 0 | Putative uncharacterized protein [Source:UniProtKB/TrEMBL;Acc:B8ADK5] | 4 | 565-5<br>86 | target 5'<br>GGGGAUCUGAAGU<br>CUGGUCCGG 3' | :.:::<br>:::::.:::::::::. | query 3'<br>CUCCU-UACUUCGGAC<br>CAGGCU 5' |
| SC08_<br>SC00 | osa-miR1<br>66h | -1.0939<br>5 | 8917<br>6.32 | 1903<br>55.2 | BGIOSG<br>A002254 | 1.40<br>758 | 1.65<br>2926 | 0 | Putative uncharacterized protein [Source:UniProtKB/TrEMBL;Acc:B8ADK5] | 4 | 565-5<br>86 | target 5'<br>GGGGAUCUGAAGU<br>CUGGUCCGG 3' | :.:::<br>:::::.:::::::::. | query 3'<br>CUCCU-UACUUCGGAC<br>CAGGCU 5' |
| SC08_<br>SC00 | osa-miR1<br>66n | -1.0963<br>5 | 9345<br>8.77 | 1998<br>28.5 | BGIOSG<br>A002254 | 1.40<br>758 | 1.65<br>2926 | 0 | Putative uncharacterized protein [Source:UniProtKB/TrEMBL;Acc:B8ADK5] | 3.5 | 565-5<br>86 | target 5'<br>GGGGAUCUGAAGU<br>CUGGUCCGG 3' | :::::<br>:::::.:::::::::. | query 3'<br>CCCCU-UACUUCGGACC<br>AGGCU 5' |
| SC08_<br>SC00 | osa-miR1<br>861d | -2.2325<br>5 | 8.89<br>3135 | 45.4<br>9423 | BGIOSG<br>A029503 | 3.13<br>409 | 85.1<br>2569 | 8.81<br>0189 | Putative uncharacterized protein [Source:UniProtKB/TrEMBL;Acc:B8BD43] | 3.5 | 209-2<br>31 | target 5'<br>CUCCAGCUUCUGCC<br>UCAAGGCCA 3' | :: :::<br>:.:::::::::::.::: | query 3'<br>GA-GUCAAGGACGGAG<br>UUCUGGU 5' |
| SC08_<br>SC00 | osa-miR1<br>861d | -2.2325<br>5 | 8.89<br>3135 | 45.4<br>9423 | BGIOSG<br>A017432 | 4.26<br>17 | 18.1<br>8219 | 0 | Putative uncharacterized protein [Source:UniProtKB/TrEMBL;Acc:B8AXI3] | 4 | 491-5<br>12 | target 5'<br>CCCAGUUUCUUCC<br>UCAAGACCG 3' | : ::::<br>:.:: ::::::::::. | query 3'<br>GAGUCAAGGACGGAGU<br>UCUGGU 5' |
| SC08_<br>SC00 | osa-miR1<br>861h | -2.2575<br>4 | 8.89<br>3135 | 46.3<br>0662 | BGIOSG<br>A017432 | 4.26<br>17 | 18.1<br>8219 | 0 | Putative uncharacterized protein [Source:UniProtKB/TrEMBL;Acc:B8AXI3] | 3.5 | 491-5<br>12 | target 5'<br>CCCAGUUUCUUCC<br>UCAAGACCG 3' | : ::::<br>:.:: ::::::::::: | query 3'<br>GAGUCAAGGACGGAGU<br>UCUGGC 5' |
| SC08_<br>SC00 | osa-miR1<br>861j | -2.2575<br>4 | 8.89<br>3135 | 46.3<br>0662 | BGIOSG<br>A017432 | 4.26<br>17 | 18.1<br>8219 | 0 | Putative uncharacterized protein [Source:UniProtKB/TrEMBL;Acc:B8AXI3] | 3.5 | 491-5<br>12 | target 5'<br>CCCAGUUUCUUCC<br>UCAAGACCG 3' | : ::::<br>:.:: ::::::::::: | query 3'<br>GAGUCAAGGACGGAGU<br>UCUGGC 5' |
| SC08_<br>SC00 | osa-miR1<br>879 | 1.54733 | 16.1<br>6934 | 4.87<br>4381 | BGIOSG<br>A001273 | -1.2<br>5435 | 4.95<br>8778 | 13.2<br>1528 | Putative uncharacterized protein [Source:UniProtKB/TrEMBL;Acc:A2WS92] | 2.5 | 640-6<br>62 | target 5'<br>CCACC-CACCCCUA<br>AACCAAACAU 3' | :::::<br>:: ::::::::::::::. | query 3'<br>GGUGGAGUAGGGAUU<br>UGGUUUGUG 5' |
| SC08_<br>SC00 | osa-miR5<br>083 | -1.3860<br>2 | 12.1<br>27 | 33.3<br>0827 | BGIOSG<br>A007106 | 2.10<br>884 | 22.3<br>145 | 4.40<br>5094 | Putative uncharacterized protein [Source:UniProtKB/TrEMBL;Acc:A2X127] | 3 | 1947-<br>1965 | target 5'<br>UGAUCAGAUAGUU<br>GUA-UCU 3' | ::::::<br>::::.::::: ::: | query 3'<br>ACUAGUCUAUUAACAU<br>CAGA 5' |
| SC08_<br>SC00 | osa-miR5<br>28 | 2.60408 | 6347<br>.273 | 1043<br>.118 | BGIOSG<br>A024054 | -2.9<br>2353 | 10.7<br>4402 | 88.1<br>0189 | Putative uncharacterized protein [Source:UniProtKB/TrEMBL;Acc:B8B7J3] | 2 | 378-3<br>97 | target 5'<br>CUCCUCUGC-UGCC<br>CCUUCCA 3' | ::::::<br>::: ::::::::::: | query 3'<br>GAGGAGACGUACGGGG<br>AAGGU 5' |
| SC08_<br>SC00 | osa-miR5<br>28 | 2.60408 | 6347<br>.273 | 1043<br>.118 | BGIOSG<br>A009946 | -2.7<br>972 | 9.91<br>7557 | 74.8<br>866 | Putative uncharacterized protein [Source:UniProtKB/TrEM | 4 | 61-80 | target 5'<br>CUCCUCUGCGUGC<br>UCC-UCCA 3' | ::::::<br>:::.:::.:: :::: | query 3'<br>GAGGAGACGUACGGGG<br>AAGGU 5' |

| | | | | | | | | | | | | | | |
|---|---|---|---|---|---|---|---|---|---|---|---|---|---|---|
| | | | | | | | | | BL;Acc:A2XL83] | | | | | |
| SC08_SC00 | osa-miR529b | 2.14474 | 50.12494 | 10.56116 | BGIOSGA029541 | -1.58003 | 7.438168 | 24.22802 | Putative uncharacterized protein [Source:UniProtKB/TrEMBL;Acc:B8BD46] | 1.5 | 573-593 | target 5' CAGCUGUGCUCUCUCUCUUCU 3' | ::::<br>::.::::::::::::: | query 3' UUCGACAUGAGAGAGAGAAGA 5' |
| SC08_SC00 | osa-miR529b | 2.14474 | 50.12494 | 10.56116 | BGIOSGA005075 | -1.3927 | 41.32315 | 110.1274 | Putative uncharacterized protein [Source:UniProtKB/TrEMBL;Acc:A2WYH5] | 2.5 | 918-938 | target 5' CAACUGUGCUCUCUCUCUUCU 3' | : ::<br>::.::::::::::::: | query 3' UUCGACAUGAGAGAGAGAAGA 5' |
| SC08_SC00 | osa-miR529b | 2.14474 | 50.12494 | 10.56116 | BGIOSGA007643 | -1.68946 | 31.4056 | 103.5197 | Putative uncharacterized protein [Source:UniProtKB/TrEMBL;Acc:A2X1G6] | 2.5 | 712-731 | target 5' CAGC-GUGCUCUCUCUCUUCU 3' | :::<br>::.::::::::::::: | query 3' UUCGACAUGAGAGAGAGAAGA 5' |
| SC08_SC00 | osa-miR811d.1 | 1.90351 | 361.3847 | 95.86283 | BGIOSGA005057 | -2.03054 | 2.479389 | 13.21528 | Putative uncharacterized protein [Source:UniProtKB/TrEMBL;Acc:B8A8I0] | 3 | 367-387 | target 5' UGUACAACCGAGAUCUGUCCA 3' | :: :::<br>::::::::.::::: | query 3' ACCUGUAGGCUCUAGGCAGGU 5' |
| SI08_SC00 | osa-miR1318 | 1.47218 | 35.58319 | 12.18595 | BGIOSGA013804 | -1.13683 | 6.466154 | 15.41783 | Putative uncharacterized protein [Source:UniProtKB/TrEMBL;Acc:A2XNA2] | 1 | 45-64 | target 5' GUCGGUGUCGUCUCUCCUGA 3' | ::::::<br>:::.:::::::::: | query 3' CAGCCACAGUAGAGAGGACU 5' |
| SI08_SC00 | osa-miR1320 | 1.62856 | 14.6519 | 4.061984 | BGIOSGA003173 | -1.90233 | 219.041 | 821.5501 | Putative uncharacterized protein [Source:UniProtKB/TrEMBL;Acc:A2WN00] | 4 | 277-297 | target 5' CUAGAUACUUCCUCCGUUUCA 3' | ::: :<br>: ::::::::::.:: | query 3' GAUAUUUUAAGGAGGCAAGGU 5' |
| SI08_SC00 | osa-miR1427 | -1.03658 | 41.86257 | 86.92647 | BGIOSGA015180 | 2.235 | 2637.383 | 559.447 | Putative uncharacterized protein [Source:UniProtKB/TrEMBL;Acc:B8ATF3] | 2.5 | 53-73 | target 5' GCGGCACCGCGCGGUUCCGCG 3' | ::: ::<br>::::.:::::::::. | query 3' CGCGGUGGCGUGCCAAGGCGU 5' |
| SI08_SC00 | osa-miR1432 | 1.4986 | 52.32822 | 17.87273 | BGIOSGA013804 | -1.13683 | 6.466154 | 15.41783 | Putative uncharacterized protein [Source:UniProtKB/TrEMBL;Acc:A2XNA2] | 1 | 45-65 | target 5' GUCGGUGUCGUCUCUCCUGAU 3' | ::::::<br>:::.:::::::::::: | query 3' CAGCCACAGUAGAGAGGACUA 5' |
| SI08_SC00 | osa-miR159a.1 | -1.53232 | 20752.32 | 60028.01 | BGIOSGA004670 | 1.2796 | 81.63519 | 33.03821 | Transcription factor GAMYB [Source:UniProtKB/Swiss-Prot;Acc:A2WW87] | 3.5 | 956-976 | target 5' UGGAGCUCCCUUCACUCCAAG 3' | ..::::<br>:::::::: :::::. | query 3' GUCUCGAGGGAAGUUAGGUUU 5' |
| SI08_SC00 | osa-miR159a.1 | -1.53232 | 20752.32 | 60028.01 | BGIOSGA037152 | 2.52657 | 2466.838 | 427.2941 | Putative uncharacterized protein [Source:UniProtKB/TrEMBL;Acc:B8BNM4] | 3.5 | 966-985 | target 5' UAG-UCUCCCUUCAAUUCAAA 3' | .::<br>:::::::::::.:::: | query 3' GUCUCGAGGGAAGUUAGGUUU 5' |
| SI08_SC00 | osa-miR159a.1 | -1.53232 | 20752.32 | 60028.01 | BGIOSGA021944 | 3.49196 | 59.81192 | 4.405094 | Putative uncharacterized protein [Source:UniProtKB/TrEMBL;Acc:A2Y963] | 3.5 | 424-443 | target 5' CA-AGUUACUUUCAAUCCAAA 3' | :: ::.<br>: :.::::::::::: | query 3' GUCUCGAGGGAAGUUAGGUUU 5' |
| SI08_SC00 | osa-miR159b | -1.53232 | 20752.32 | 60028.01 | BGIOSGA004670 | 1.2796 | 81.63519 | 33.03821 | Transcription factor GAMYB [Source:UniProtKB/Swiss-Prot;Acc:A2WW87] | 3.5 | 956-976 | target 5' UGGAGCUCCCUUCACUCCAAG 3' | ..::::<br>:::::::: :::::. | query 3' GUCUCGAGGGAAGUUAGGUUU 5' |
| SI08_SC00 | osa-miR159b | -1.53232 | 20752.32 | 60028.01 | BGIOSGA037152 | 2.52657 | 2466.838 | 427.2941 | Putative uncharacterized protein [Source:UniProtKB/TrEMBL;Acc:B8BNM4] | 3.5 | 966-985 | target 5' UAG-UCUCCCUUCAAUUCAAA 3' | .::<br>:::::::::::.:::: | query 3' GUCUCGAGGGAAGUUAGGUUU 5' |
| SI08_SC00 | osa-miR159b | -1.53232 | 20752.32 | 60028.01 | BGIOSGA021944 | 3.49196 | 59.81192 | 4.405094 | Putative uncharacterized protein [Source:UniProtKB/TrEM | 3.5 | 424-443 | target 5' CA-AGUUACUUUCAAUCCAAA 3' | :: ::.<br>: :.::::::::::: | query 3' GUCUCGAGGGAAGUUAGGUUU 5' |

| | | | | | | | | | | | | | | |
|---|---|---|---|---|---|---|---|---|---|---|---|---|---|---|
| | | | | | | | | | BL;Acc:A2Y963] | | | | | |
| SI08_SC00 | osa-miR159c | -1.53317 | 19968.45 | 57794.73 | BGIOSGA004670 | 1.2796 | 81.63519 | 33.03821 | Transcription factor GAMYB [Source:UniProtKB/Swiss-Prot;Acc:A2WW87] | 3 | 956-976 | target 5' UGGAGCUCCCUUCACUCCAAG 3' | :::::: :::::::: ::::: | query 3' ACCUCGAGGGAAGUUAGGUUA 5' |
| SI08_SC00 | osa-miR159d | -1.53332 | 19970.54 | 57806.91 | BGIOSGA004670 | 1.2796 | 81.63519 | 33.03821 | Transcription factor GAMYB [Source:UniProtKB/Swiss-Prot;Acc:A2WW87] | 3.5 | 956-976 | target 5' UGGAGCUCCCUUCACUCCAAG 3' | .:::: :::::::: ::::: | query 3' GCCUCGAGGGAAGUUAGGUUA 5' |
| SI08_SC00 | osa-miR159e | -1.53317 | 19968.45 | 57794.73 | BGIOSGA025162 | 1.17398 | 16.16539 | 6.607641 | Putative uncharacterized protein [Source:UniProtKB/TrEMBL;Acc:A2YI86] | 3.5 | 1099-1119 | target 5' AGGGGGACCCUUCAGUCCAAU 3' | :::.: :::::::.:::::: | query 3' UCCUCGAGGGAAGUUAGGUUA 5' |
| SI08_SC00 | osa-miR159e | -1.53317 | 19968.45 | 57794.73 | BGIOSGA004670 | 1.2796 | 81.63519 | 33.03821 | Transcription factor GAMYB [Source:UniProtKB/Swiss-Prot;Acc:A2WW87] | 4 | 956-976 | target 5' UGGAGCUCCCUUCACUCCAAG 3' | :::: ::::::::: ::::: | query 3' UCCUCGAGGGAAGUUAGGUUA 5' |
| SI08_SC00 | osa-miR159f | -1.53323 | 19995.66 | 57875.97 | BGIOSGA004670 | 1.2796 | 81.63519 | 33.03821 | Transcription factor GAMYB [Source:UniProtKB/Swiss-Prot;Acc:A2WW87] | 2.5 | 956-976 | target 5' UGGAGCUCCCUUCACUCCAAG 3' | :.:::: :::::::: :::::: | query 3' AUCUCGAGGGAAGUUAGGUUC 5' |
| SI08_SC00 | osa-miR159f | -1.53323 | 19995.66 | 57875.97 | BGIOSGA037152 | 2.52657 | 2466.838 | 427.2941 | Putative uncharacterized protein [Source:UniProtKB/TrEMBL;Acc:B8BNM4] | 4 | 966-985 | target 5' UAG-UCUCCCUUCAAUUCAAA 3' | ::: :::::::::::.::: | query 3' AUCUCGAGGGAAGUUAGGUUC 5' |
| SI08_SC00 | osa-miR162a | -1.17852 | 616.4264 | 1396.51 | BGIOSGA011645 | 1.36253 | 46.87962 | 17.62038 | Putative uncharacterized protein [Source:UniProtKB/TrEMBL;Acc:B8AKZ8] | 4 | 587-606 | target 5' CUGGAUGCAGA-UUUUAUCGA 3' | :::::: ::::: :::::::: | query 3' GACCUACGUCUCCAAAUAGCU 5' |
| SI08_SC00 | osa-miR166a | -1.57823 | 66920.46 | 199828.5 | BGIOSGA011687 | 1.25215 | 122.0487 | 50.65858 | Homeobox-leucine zipper protein HOX10 [Source:UniProtKB/Swiss-Prot;Acc:A2XBL9] | 3 | 563-583 | target 5' CUGGGAUGAAGCCUGGUCCGG 3' | :: .::::::::::::::. | query 3' CCCCUUACUUCGGACCAGGCU 5' |
| SI08_SC00 | osa-miR166a | -1.57823 | 66920.46 | 199828.5 | BGIOSGA002254 | 2.33381 | 4.041346 | 0 | Putative uncharacterized protein [Source:UniProtKB/TrEMBL;Acc:B8ADK5] | 3.5 | 565-586 | target 5' GGGGAUCUGAAGUCUGGUCCGG 3' | ::::: :::::.::::::::. | query 3' CCCCU-UACUUCGGACCAGGCU 5' |
| SI08_SC00 | osa-miR166a | -1.57823 | 66920.46 | 199828.5 | BGIOSGA009117 | 2.05717 | 113.1577 | 26.43057 | Putative uncharacterized protein [Source:UniProtKB/TrEMBL;Acc:B8AJ56] | 4 | 381-400 | target 5' GGGGGAGGAAG-CUGGUCCGG 3' | ::::.: :::: ::::::::. | query 3' CCCCUUACUUCGGACCAGGCU 5' |
| SI08_SC00 | osa-miR166b | -1.57823 | 66920.46 | 199828.5 | BGIOSGA011687 | 1.25215 | 122.0487 | 50.65858 | Homeobox-leucine zipper protein HOX10 [Source:UniProtKB/Swiss-Prot;Acc:A2XBL9] | 3 | 563-583 | target 5' CUGGGAUGAAGCCUGGUCCGG 3' | :: .::::::::::::::. | query 3' CCCCUUACUUCGGACCAGGCU 5' |
| SI08_SC00 | osa-miR166b | -1.57823 | 66920.46 | 199828.5 | BGIOSGA002254 | 2.33381 | 4.041346 | 0 | Putative uncharacterized protein [Source:UniProtKB/TrEMBL;Acc:B8ADK5] | 3.5 | 565-586 | target 5' GGGGAUCUGAAGUCUGGUCCGG 3' | ::::: :::::.::::::::. | query 3' CCCCU-UACUUCGGACCAGGCU 5' |
| SI08_SC00 | osa-miR166b | -1.57823 | 66920.46 | 199828.5 | BGIOSGA009117 | 2.05717 | 113.1577 | 26.43057 | Putative uncharacterized protein [Source:UniProtKB/TrEMBL;Acc:B8AJ56] | 4 | 381-400 | target 5' GGGGGAGGAAG-CUGGUCCGG 3' | ::::.: :::: ::::::::. | query 3' CCCCUUACUUCGGACCAGGCU 5' |
| SI08_SC00 | osa-miR166c | -1.57823 | 66920.46 | 199828.5 | BGIOSGA011687 | 1.25215 | 122.0487 | 50.65858 | Homeobox-leucine zipper protein HOX10 [Source:UniProtKB/Swiss- | 3 | 563-583 | target 5' CUGGGAUGAAGCCUGGUCCGG 3' | :: .::::::::::::::. | query 3' CCCCUUACUUCGGACCAGGCU 5' |

| | | | | | | | | | | | | | | |
|---|---|---|---|---|---|---|---|---|---|---|---|---|---|---|
| | | | | | | | | | Prot;Acc:A2XBL9] | | | | | |
| SI08_SC00 | osa-miR166c | -1.57823 | 66920.46 | 199828.5 | BGIOSGA002254 | 2.33381 | 4.041346 | 0 | Putative uncharacterized protein [Source:UniProtKB/TrEMBL;Acc:B8ADK5] | 3.5 | 565-586 | target 5'<br>GGGGAUCUGAAGU<br>CUGGUCCGG 3' | :::::<br>:::::.::::::::. | query 3'<br>CCCCU-UACUUCGGACC<br>AGGCU 5' |
| SI08_SC00 | osa-miR166c | -1.57823 | 66920.46 | 199828.5 | BGIOSGA009117 | 2.05717 | 113.1577 | 26.43057 | Putative uncharacterized protein [Source:UniProtKB/TrEMBL;Acc:B8AJ56] | 4 | 381-400 | target 5'<br>GGGGGAGGAAG-CU<br>GGUCCGG 3' | ::::.:<br>:::: ::::::::. | query 3'<br>CCCCUUACUUCGGACC<br>AGGCU 5' |
| SI08_SC00 | osa-miR166d | -1.57823 | 66920.46 | 199828.5 | BGIOSGA011687 | 1.25215 | 122.0487 | 50.65858 | Homeobox-leucine zipper protein HOX10 [Source:UniProtKB/Swiss-Prot;Acc:A2XBL9] | 3 | 563-583 | target 5'<br>CUGGGAUGAAGCC<br>UGGUCCGG 3' | ::<br>.::::::::::::::. | query 3'<br>CCCCUUACUUCGGACC<br>AGGCU 5' |
| SI08_SC00 | osa-miR166d | -1.57823 | 66920.46 | 199828.5 | BGIOSGA002254 | 2.33381 | 4.041346 | 0 | Putative uncharacterized protein [Source:UniProtKB/TrEMBL;Acc:B8ADK5] | 3.5 | 565-586 | target 5'<br>GGGGAUCUGAAGU<br>CUGGUCCGG 3' | :::::<br>:::::.::::::::. | query 3'<br>CCCCU-UACUUCGGACC<br>AGGCU 5' |
| SI08_SC00 | osa-miR166d | -1.57823 | 66920.46 | 199828.5 | BGIOSGA009117 | 2.05717 | 113.1577 | 26.43057 | Putative uncharacterized protein [Source:UniProtKB/TrEMBL;Acc:B8AJ56] | 4 | 381-400 | target 5'<br>GGGGGAGGAAG-CU<br>GGUCCGG 3' | ::::.:<br>:::: ::::::::. | query 3'<br>CCCCUUACUUCGGACC<br>AGGCU 5' |
| SI08_SC00 | osa-miR166f | -1.57823 | 66920.46 | 199828.5 | BGIOSGA011687 | 1.25215 | 122.0487 | 50.65858 | Homeobox-leucine zipper protein HOX10 [Source:UniProtKB/Swiss-Prot;Acc:A2XBL9] | 3 | 563-583 | target 5'<br>CUGGGAUGAAGCC<br>UGGUCCGG 3' | ::<br>.::::::::::::::. | query 3'<br>CCCCUUACUUCGGACC<br>AGGCU 5' |
| SI08_SC00 | osa-miR166f | -1.57823 | 66920.46 | 199828.5 | BGIOSGA002254 | 2.33381 | 4.041346 | 0 | Putative uncharacterized protein [Source:UniProtKB/TrEMBL;Acc:B8ADK5] | 3.5 | 565-586 | target 5'<br>GGGGAUCUGAAGU<br>CUGGUCCGG 3' | :::::<br>:::::.::::::::. | query 3'<br>CCCCU-UACUUCGGACC<br>AGGCU 5' |
| SI08_SC00 | osa-miR166f | -1.57823 | 66920.46 | 199828.5 | BGIOSGA009117 | 2.05717 | 113.1577 | 26.43057 | Putative uncharacterized protein [Source:UniProtKB/TrEMBL;Acc:B8AJ56] | 4 | 381-400 | target 5'<br>GGGGGAGGAAG-CU<br>GGUCCGG 3' | ::::.:<br>:::: ::::::::. | query 3'<br>CCCCUUACUUCGGACC<br>AGGCU 5' |
| SI08_SC00 | osa-miR166g | -1.57881 | 63722.16 | 190355.2 | BGIOSGA011687 | 1.25215 | 122.0487 | 50.65858 | Homeobox-leucine zipper protein HOX10 [Source:UniProtKB/Swiss-Prot;Acc:A2XBL9] | 3 | 563-583 | target 5'<br>CUGGGAUGAAGCC<br>UGGUCCGG 3' | ::<br>.::::::::::::::. | query 3'<br>CUCCUUACUUCGGACC<br>AGGCU 5' |
| SI08_SC00 | osa-miR166g | -1.57881 | 63722.16 | 190355.2 | BGIOSGA002254 | 2.33381 | 4.041346 | 0 | Putative uncharacterized protein [Source:UniProtKB/TrEMBL;Acc:B8ADK5] | 4 | 565-586 | target 5'<br>GGGGAUCUGAAGU<br>CUGGUCCGG 3' | :.:::<br>:::::.::::::::. | query 3'<br>CUCCU-UACUUCGGAC<br>CAGGCU 5' |
| SI08_SC00 | osa-miR166h | -1.57881 | 63722.16 | 190355.2 | BGIOSGA011687 | 1.25215 | 122.0487 | 50.65858 | Homeobox-leucine zipper protein HOX10 [Source:UniProtKB/Swiss-Prot;Acc:A2XBL9] | 3 | 563-583 | target 5'<br>CUGGGAUGAAGCC<br>UGGUCCGG 3' | ::<br>.::::::::::::::. | query 3'<br>CUCCUUACUUCGGACC<br>AGGCU 5' |
| SI08_SC00 | osa-miR166h | -1.57881 | 63722.16 | 190355.2 | BGIOSGA002254 | 2.33381 | 4.041346 | 0 | Putative uncharacterized protein [Source:UniProtKB/TrEMBL;Acc:B8ADK5] | 4 | 565-586 | target 5'<br>GGGGAUCUGAAGU<br>CUGGUCCGG 3' | :.:::<br>:::::.::::::::. | query 3'<br>CUCCU-UACUUCGGAC<br>CAGGCU 5' |
| SI08_SC00 | osa-miR166i | -1.65604 | 13792.67 | 43469.73 | BGIOSGA011687 | 1.25215 | 122.0487 | 50.65858 | Homeobox-leucine zipper protein HOX10 [Source:UniProtKB/Swiss-Prot;Acc:A2XBL9] | 4 | 563-583 | target 5'<br>CUGGGAUGAAGCC<br>UGGUCCGG 3' | ::<br>.::::::::::.::::. | query 3'<br>CUCCUUACUUCGGACU<br>AGGCU 5' |
| SI08_SC00 | osa-miR166i | -1.65604 | 13792.67 | 43469.73 | BGIOSGA017414 | 3.18312 | 8.082693 | 0 | Putative uncharacterized protein [Source:UniProtKB/TrEM | 4 | 3637-3658 | target 5'<br>AAGGAAGUGAAGC<br>UUGAUCUGA 3' | ::::<br>: :::::.:::::.:: | query 3'<br>CUCCUU-ACUUCGGAC<br>UAGGCU 5' |

| | | | | | | | | | | | | | | |
|---|---|---|---|---|---|---|---|---|---|---|---|---|---|---|
| | | | | | | | | | BL;Acc:A2XZA6] | | | | | |
| SI08_SC00 | osa-miR166j | -1.65604 | 13792.67 | 43469.73 | BGIOSGA011687 | 1.25215 | 122.0487 | 50.65858 | Homeobox-leucine zipper protein HOX10 [Source:UniProtKB/Swiss-Prot;Acc:A2XBL9] | 4 | 563-583 | target 5' CUGGGAUGAAGCCUGGUCCGG 3' | :: .::::::::::.::::. | query 3' CUCCUUACUUCGGACUAGGCU 5' |
| SI08_SC00 | osa-miR166j | -1.65604 | 13792.67 | 43469.73 | BGIOSGA017414 | 3.18312 | 8.082693 | 0 | Putative uncharacterized protein [Source:UniProtKB/TrEMBL;Acc:A2XZA6] | 4 | 3637-3658 | target 5' AAGGAAGUGAAGCUUGAUCUGA 3' | :::: : ::::::.:::::.:: | query 3' CUCCUU-ACUUCGGACUAGGCU 5' |
| SI08_SC00 | osa-miR166k | -1.26585 | 9492.338 | 22827.54 | BGIOSGA011687 | 1.25215 | 122.0487 | 50.65858 | Homeobox-leucine zipper protein HOX10 [Source:UniProtKB/Swiss-Prot;Acc:A2XBL9] | 2.5 | 564-583 | target 5' UGGGA-UGAAGCCUGGUCCGG 3' | :::: ::::::::::::::. | query 3' UCCCUAACUUCGGACCAGGCU 5' |
| SI08_SC00 | osa-miR166k | -1.26585 | 9492.338 | 22827.54 | BGIOSGA002254 | 2.33381 | 4.041346 | 0 | Putative uncharacterized protein [Source:UniProtKB/TrEMBL;Acc:B8ADK5] | 3 | 565-586 | target 5' GGGGAUCUGAAGUCUGGUCCGG 3' | .::::: :::::.::::::::. | query 3' UCCCUA-ACUUCGGACCAGGCU 5' |
| SI08_SC00 | osa-miR166l | -1.26585 | 9492.338 | 22827.54 | BGIOSGA011687 | 1.25215 | 122.0487 | 50.65858 | Homeobox-leucine zipper protein HOX10 [Source:UniProtKB/Swiss-Prot;Acc:A2XBL9] | 2.5 | 564-583 | target 5' UGGGA-UGAAGCCUGGUCCGG 3' | :::: ::::::::::::::. | query 3' UCCCUAACUUCGGACCAGGCU 5' |
| SI08_SC00 | osa-miR166l | -1.26585 | 9492.338 | 22827.54 | BGIOSGA002254 | 2.33381 | 4.041346 | 0 | Putative uncharacterized protein [Source:UniProtKB/TrEMBL;Acc:B8ADK5] | 3 | 565-586 | target 5' GGGGAUCUGAAGUCUGGUCCGG 3' | .::::: :::::.::::::::. | query 3' UCCCUA-ACUUCGGACCAGGCU 5' |
| SI08_SC00 | osa-miR166m | -1.51306 | 58663.07 | 167435 | BGIOSGA011687 | 1.25215 | 122.0487 | 50.65858 | Homeobox-leucine zipper protein HOX10 [Source:UniProtKB/Swiss-Prot;Acc:A2XBL9] | 3 | 563-583 | target 5' CUGGGAUGAAGCCUGGUCCGG 3' | :: .::::::::::::::::. | query 3' UCCCUUACUUCGGACCAGGCU 5' |
| SI08_SC00 | osa-miR166n | -1.57823 | 66920.46 | 199828.5 | BGIOSGA011687 | 1.25215 | 122.0487 | 50.65858 | Homeobox-leucine zipper protein HOX10 [Source:UniProtKB/Swiss-Prot;Acc:A2XBL9] | 3 | 563-583 | target 5' CUGGGAUGAAGCCUGGUCCGG 3' | :: .::::::::::::::::. | query 3' CCCCUUACUUCGGACCAGGCU 5' |
| SI08_SC00 | osa-miR166n | -1.57823 | 66920.46 | 199828.5 | BGIOSGA002254 | 2.33381 | 4.041346 | 0 | Putative uncharacterized protein [Source:UniProtKB/TrEMBL;Acc:B8ADK5] | 3.5 | 565-586 | target 5' GGGGAUCUGAAGUCUGGUCCGG 3' | ::::: :::::.::::::::. | query 3' CCCCU-UACUUCGGACCAGGCU 5' |
| SI08_SC00 | osa-miR166n | -1.57823 | 66920.46 | 199828.5 | BGIOSGA009117 | 2.05717 | 113.1577 | 26.43057 | Putative uncharacterized protein [Source:UniProtKB/TrEMBL;Acc:B8AJ56] | 4 | 381-400 | target 5' GGGGGAGGAAG-CUGGUCCGG 3' | ::::.: :::: ::::::::. | query 3' CCCCUUACUUCGGACCAGGCU 5' |
| SI08_SC00 | osa-miR1850.1 | 2.30077 | 43.9557 | 8.123969 | BGIOSGA012292 | -1.7621 | 320.0746 | 1088.058 | Putative uncharacterized protein [Source:UniProtKB/TrEMBL;Acc:A2XEV9] | 4 | 472-493 | target 5' CACCAUUUCUCCCAACUUUUCA 3' | : ::: ::::::::::::.:: | query 3' GGGGU-UAGAGGGUUGAAAGGU 5' |
| SI08_SC00 | osa-miR1861d | -1.77783 | 12.55877 | 45.49423 | BGIOSGA029503 | 3.00206 | 77.59385 | 8.810189 | Putative uncharacterized protein [Source:UniProtKB/TrEMBL;Acc:B8BD43] | 3.5 | 209-231 | target 5' CUCCAGCUUCUGCCUCAAGGCCA 3' | :: ::: :.::::::::::.::: | query 3' GA-GUCAAGGACGGAGUUCUGGU 5' |
| SI08_SC00 | osa-miR1861d | -1.77783 | 12.55877 | 45.49423 | BGIOSGA017432 | 4.51243 | 21.82327 | 0 | Putative uncharacterized protein [Source:UniProtKB/TrEMBL;Acc:B8AXI3] | 4 | 491-512 | target 5' CCCAGUUUCUUCCUCAAGACCG 3' | : :::: :.:: ::::::::::. | query 3' GAGUCAAGGACGGAGUUCUGGU 5' |
| SI08_SC00 | osa-miR1861h | -1.69555 | 13.60534 | 46.30662 | BGIOSGA017432 | 4.51243 | 21.82327 | 0 | Putative uncharacterized protein [Source:UniProtKB/TrEM | 3.5 | 491-512 | target 5' CCCAGUUUCUUCCUCAAGACCG 3' | : :::: :.:: :::::::::: | query 3' GAGUCAAGGACGGAGUUCUGGC 5' |

| | | | | | | | | | | | | | | |
|---|---|---|---|---|---|---|---|---|---|---|---|---|---|---|
| | | | | | | | | | BL;Acc:B8AXI3] | | | | | |
| SI08_SC00 | osa-miR1861j | -1.69555 | 13.60534 | 46.30662 | BGIOSGA017432 | 4.51243 | 21.82327 | 0 | Putative uncharacterized protein [Source:UniProtKB/TrEMBL;Acc:B8AXI3] | 3.5 | 491-512 | target 5'<br>CCCAGUUUCUUCCUCAAGACCG 3' | : ::::<br>:.:: :::::::::: | query 3'<br>GAGUCAAGGACGGAGUUCUGGC 5' |
| SI08_SC00 | osa-miR1862f | -1.31898 | 220.8251 | 552.4299 | BGIOSGA005796 | 3.35646 | 370.1873 | 35.24075 | Putative uncharacterized protein [Source:UniProtKB/TrEMBL;Acc:B8AHB3] | 3 | 1138-1156 | target 5'<br>UCAAAG-AAACCAACUUCAU 3' | .::::.<br>::::::::.:::: | query 3'<br>GGUUUUAUUUGGUUGGAGUA 5' |
| SI08_SC00 | osa-miR1862f | -1.31898 | 220.8251 | 552.4299 | BGIOSGA017434 | 1.4861 | 106.6915 | 37.4433 | Putative uncharacterized protein [Source:UniProtKB/TrEMBL;Acc:B8AXI0] | 3.5 | 385-403 | target 5'<br>UCAAAA-AAACCAAGCUCAU 3' | .:::::<br>::::::: ::::: | query 3'<br>GGUUUUAUUUGGUUGGAGUA 5' |
| SI08_SC00 | osa-miR1862f | -1.31898 | 220.8251 | 552.4299 | BGIOSGA014360 | 3.06935 | 321.6912 | 37.4433 | Putative uncharacterized protein [Source:UniProtKB/TrEMBL;Acc:B8AU52] | 4 | 302-321 | target 5'<br>CUGCAAGAAACCAAUCUCAU 3' | :.. ::<br>::::::::.::::: | query 3'<br>GGUUUUAUUUGGUUGGAGUA 5' |
| SI08_SC00 | osa-miR1862g | -1.31898 | 220.8251 | 552.4299 | BGIOSGA005796 | 3.35646 | 370.1873 | 35.24075 | Putative uncharacterized protein [Source:UniProtKB/TrEMBL;Acc:B8AHB3] | 3 | 1138-1156 | target 5'<br>UCAAAG-AAACCAACUUCAU 3' | .::::.<br>::::::::.:::: | query 3'<br>GGUUUUAUUUGGUUGGAGUA 5' |
| SI08_SC00 | osa-miR1862g | -1.31898 | 220.8251 | 552.4299 | BGIOSGA017434 | 1.4861 | 106.6915 | 37.4433 | Putative uncharacterized protein [Source:UniProtKB/TrEMBL;Acc:B8AXI0] | 3.5 | 385-403 | target 5'<br>UCAAAA-AAACCAAGCUCAU 3' | .:::::<br>::::::: ::::: | query 3'<br>GGUUUUAUUUGGUUGGAGUA 5' |
| SI08_SC00 | osa-miR1862g | -1.31898 | 220.8251 | 552.4299 | BGIOSGA014360 | 3.06935 | 321.6912 | 37.4433 | Putative uncharacterized protein [Source:UniProtKB/TrEMBL;Acc:B8AU52] | 4 | 302-321 | target 5'<br>CUGCAAGAAACCAAUCUCAU 3' | :.. ::<br>::::::::.::::: | query 3'<br>GGUUUUAUUUGGUUGGAGUA 5' |
| SI08_SC00 | osa-miR2055 | -1.36934 | 57.56104 | 150.2934 | BGIOSGA030036 | 1.20397 | 118.0073 | 50.65858 | Putative uncharacterized protein [Source:UniProtKB/TrEMBL;Acc:B8BEA3] | 4 | 1464-1483 | target 5'<br>UGAACC-CCUUCUCAAGGAAG 3' | .:::<br>: :::::.:::::::. | query 3'<br>CUUUGGUGGAAGGGUUCCUUU 5' |
| SI08_SC00 | osa-miR2055 | -1.36934 | 57.56104 | 150.2934 | BGIOSGA014296 | 1.77602 | 2.424808 | 0 | Putative uncharacterized protein [Source:UniProtKB/TrEMBL;Acc:A2XXW3] | 4 | 2255-2275 | target 5'<br>CAAACAAACUUCCUAAGGAAA 3' | ::::<br>: :::::.::::::: | query 3'<br>CUUUGGUGGAAGGGUUCCUUU 5' |
| SI08_SC00 | osa-miR2864.2 | -1.05007 | 19.88472 | 42.24464 | BGIOSGA008486 | 4.61116 | 23.43981 | 0 | Putative uncharacterized protein [Source:UniProtKB/TrEMBL;Acc:A2X6F1] | 4 | 1721-1741 | target 5'<br>UACCUGUCCAAUACAAAACAG 3' | :::::.<br>: :::: :::::::. | query 3'<br>AUGGAUAUGUUACGUUUUGUU 5' |
| SI08_SC00 | osa-miR2871a-3p | -1.13987 | 35.58319 | 79.6149 | BGIOSGA013087 | 4.56264 | 22.63154 | 0 | Putative uncharacterized protein [Source:UniProtKB/TrEMBL;Acc:B8AM58] | 4 | 539-558 | target 5'<br>AUGACC-UAGAAAGUAAAAUA 3' | ::::<br>: :::::: ::::::: | query 3'<br>CACUGGUAUCUUUGAUUUUAU 5' |
| SI08_SC00 | osa-miR2871b | -1.13987 | 35.58319 | 79.6149 | BGIOSGA013087 | 4.56264 | 22.63154 | 0 | Putative uncharacterized protein [Source:UniProtKB/TrEMBL;Acc:B8AM58] | 4 | 539-558 | target 5'<br>AUGACC-UAGAAAGUAAAAUA 3' | ::::<br>: :::::: ::::::: | query 3'<br>CACUGGUAUCUUUGAUUUUAU 5' |
| SI08_SC00 | osa-miR2873b | -1.31847 | 94.19079 | 236.4075 | BGIOSGA013396 | 1.58361 | 259.4544 | 85.89934 | Putative uncharacterized protein [Source:UniProtKB/TrEMBL;Acc:B8AQ13] | 3.5 | 1620-1640 | target 5'<br>CAUGCCAUAUCUCAAGUCUAU 3' | :::.::<br>: ::::::::::.: | query 3'<br>GUAUGGUUUAGAGUUCAGGUU 5' |
| SI08_SC00 | osa-miR394 | -1.38881 | 78.49232 | 207.1612 | BGIOSGA020470 | 6.97496 | 678.9462 | 4.405094 | Putative uncharacterized protein [Source:UniProtKB/TrEM | 3 | 1366-1385 | target 5'<br>GGAGUUGGACAGAAUGCAAA 3' | :::: :<br>:::::::::: :: | query 3'<br>CCUCCACCUGUCUUACGGUU 5' |

| | | | | | | | | | | | | | | |
|---|---|---|---|---|---|---|---|---|---|---|---|---|---|---|
| | | | | | | | | | BL;Acc:B8AX92] | | | | | |
| SI08_SC00_ | osa-miR394 | -1.38881 | 78.49232 | 207.1612 | BGIOSGA025510 | 2.05961 | 30.71423 | 6.607641 | Putative uncharacterized protein [Source:UniProtKB/TrEMBL;Acc:B8B516] | 4 | 20-38 | target 5' GGCGGCGGA-AGAAUGCCAA 3' | :: ::<br>::: ::::::::: | query 3' CCUCCACCUGUCUUACGGUU 5' |
| SI08_SC00_ | osa-miR396a | -1.25267 | 455.2555 | 1086.175 | BGIOSGA001547 | 1.69393 | 536.6908 | 165.191 | Putative uncharacterized protein [Source:UniProtKB/TrEMBL;Acc:B8A8X2] | 3 | 127-147 | target 5' CGGUUUAAGAGAGCUGUGGGA 3' | :.:::.<br>::::.::::::::.: | query 3' GUCAAGUUCUUUCGACACCUU 5' |
| SI08_SC00_ | osa-miR396a | -1.25267 | 455.2555 | 1086.175 | BGIOSGA037400 | 1.77602 | 2.424808 | 0 | Putative uncharacterized protein [Source:UniProtKB/TrEMBL;Acc:A2ZKJ3] | 3 | 576-597 | target 5' CCGUUCAAGAAAGCAUGUGGAA 3' | : ::::<br>:::::::: ::::::: | query 3' GUCAAGUUCUUUCG-ACACCUU 5' |
| SI08_SC00_ | osa-miR396a | -1.25267 | 455.2555 | 1086.175 | BGIOSGA014417 | 1.69755 | 80.82693 | 24.22802 | Putative uncharacterized protein [Source:UniProtKB/TrEMBL;Acc:B8ATM9] | 3 | 450-471 | target 5' CCGUUCAAGAAAGCCUGUGGAA 3' | : ::::<br>::::::: :::::::: | query 3' GUCAAGUUCUUUC-GACACCUU 5' |
| SI08_SC00_ | osa-miR396a | -1.25267 | 455.2555 | 1086.175 | BGIOSGA021248 | 2.75449 | 154.3794 | 22.02547 | Putative uncharacterized protein [Source:UniProtKB/TrEMBL;Acc:B8B225] | 3.5 | 697-717 | target 5' CAGUUCUGGAAAGCUGGGGAA 3' | ::::::<br>.:::::::: :::: | query 3' GUCAAGUUCUUUCGACACCUU 5' |
| SI08_SC00_ | osa-miR396a | -1.25267 | 455.2555 | 1086.175 | BGIOSGA034107 | 2.13786 | 294.21 | 66.07641 | Putative uncharacterized protein [Source:UniProtKB/TrEMBL;Acc:A2ZDM9] | 4 | 1603-1623 | target 5' CUGUCCAAGAAGGCUGUGGAU 3' | : :: :<br>:::::.:::::::: | query 3' GUCAAGUUCUUUCGACACCUU 5' |
| SI08_SC00_ | osa-miR396a | -1.25267 | 455.2555 | 1086.175 | BGIOSGA002892 | 2.81729 | 720.1679 | 101.3172 | Putative uncharacterized protein [Source:UniProtKB/TrEMBL;Acc:A2WLE4] | 4 | 1681-1701 | target 5' GAGUUGCAGAAGGCUGUGGAA 3' | ::::<br>::::.::::::::: | query 3' GUCAAGUUCUUUCGACACCUU 5' |
| SI08_SC00_ | osa-miR396b | -1.25267 | 455.2555 | 1086.175 | BGIOSGA001547 | 1.69393 | 536.6908 | 165.191 | Putative uncharacterized protein [Source:UniProtKB/TrEMBL;Acc:B8A8X2] | 3 | 127-147 | target 5' CGGUUUAAGAGAGCUGUGGGA 3' | :.:::.<br>::::.::::::::.: | query 3' GUCAAGUUCUUUCGACACCUU 5' |
| SI08_SC00_ | osa-miR396b | -1.25267 | 455.2555 | 1086.175 | BGIOSGA037400 | 1.77602 | 2.424808 | 0 | Putative uncharacterized protein [Source:UniProtKB/TrEMBL;Acc:A2ZKJ3] | 3 | 576-597 | target 5' CCGUUCAAGAAAGCAUGUGGAA 3' | : ::::<br>:::::::: ::::::: | query 3' GUCAAGUUCUUUCG-ACACCUU 5' |
| SI08_SC00_ | osa-miR396b | -1.25267 | 455.2555 | 1086.175 | BGIOSGA014417 | 1.69755 | 80.82693 | 24.22802 | Putative uncharacterized protein [Source:UniProtKB/TrEMBL;Acc:B8ATM9] | 3 | 450-471 | target 5' CCGUUCAAGAAAGCCUGUGGAA 3' | : ::::<br>::::::: :::::::: | query 3' GUCAAGUUCUUUC-GACACCUU 5' |
| SI08_SC00_ | osa-miR396b | -1.25267 | 455.2555 | 1086.175 | BGIOSGA021248 | 2.75449 | 154.3794 | 22.02547 | Putative uncharacterized protein [Source:UniProtKB/TrEMBL;Acc:B8B225] | 3.5 | 697-717 | target 5' CAGUUCUGGAAAGCUGGGGAA 3' | ::::::<br>.:::::::: :::: | query 3' GUCAAGUUCUUUCGACACCUU 5' |
| SI08_SC00_ | osa-miR396b | -1.25267 | 455.2555 | 1086.175 | BGIOSGA034107 | 2.13786 | 294.21 | 66.07641 | Putative uncharacterized protein [Source:UniProtKB/TrEMBL;Acc:A2ZDM9] | 4 | 1603-1623 | target 5' CUGUCCAAGAAGGCUGUGGAU 3' | : :: :<br>:::::.:::::::: | query 3' GUCAAGUUCUUUCGACACCUU 5' |
| SI08_SC00_ | osa-miR396b | -1.25267 | 455.2555 | 1086.175 | BGIOSGA002892 | 2.81729 | 720.1679 | 101.3172 | Putative uncharacterized protein [Source:UniProtKB/TrEMBL;Acc:A2WLE4] | 4 | 1681-1701 | target 5' GAGUUGCAGAAGGCUGUGGAA 3' | ::::<br>::::.::::::::: | query 3' GUCAAGUUCUUUCGACACCUU 5' |
| SI08_SC00_ | osa-miR396c | -1.27383 | 457.3486 | 1107.297 | BGIOSGA002892 | 2.81729 | 720.1679 | 101.3172 | Putative uncharacterized protein [Source:UniProtKB/TrEM | 3.5 | 1681-1701 | target 5' GAGUUGCAGAAGGCUGUGGAA 3' | .::::<br>::::.::::::::: | query 3' UUCAAGUUCUUUCGACACCUU 5' |

| | | | | | | | | | | | | | | |
|---|---|---|---|---|---|---|---|---|---|---|---|---|---|---|
| | | | | | | | | | BL;Acc:A2WLE4] | | | | | |
| SI08_SC00 | osa-miR396c | -1.27383 | 457.3486 | 1107.297 | BGIOSGA001547 | 1.69393 | 536.6908 | 165.191 | Putative uncharacterized protein [Source:UniProtKB/TrEMBL;Acc:B8A8X2] | 4 | 127-147 | target 5' CGGUUUAAGAGAGCUGUGGGA 3' | .::: .::::.:::::::::.: | query 3' UUCAAGUUCUUUCGACACCUU 5' |
| SI08_SC00 | osa-miR396c | -1.27383 | 457.3486 | 1107.297 | BGIOSGA034898 | 5.65261 | 49.30442 | 0 | Putative uncharacterized protein [Source:UniProtKB/TrEMBL;Acc:A2ZBY7] | 4 | 1281-1300 | target 5' AAGUUCAAGAAAGC-AUGGAA 3' | :::::: ::::::::   ::::: | query 3' UUCAAGUUCUUUCGACACCUU 5' |
| SI08_SC00 | osa-miR396c | -1.27383 | 457.3486 | 1107.297 | BGIOSGA006677 | 2.79308 | 51.72923 | 6.607641 | Putative uncharacterized protein [Source:UniProtKB/TrEMBL;Acc:A2X3J9] | 4 | 602-621 | target 5' AGAUUCAGGAAAGCUG-GGAA 3' | :. ::: :.:::::::: :::: | query 3' UUCAAGUUCUUUCGACACCUU 5' |
| SI08_SC00 | osa-miR396c | -1.27383 | 457.3486 | 1107.297 | BGIOSGA004420 | 4.7904 | 26.67289 | 0 | Putative uncharacterized protein [Source:UniProtKB/TrEMBL;Acc:A2WUV3] | 4 | 1247-1266 | target 5' AAGUAAAAG-AAGCUGUGGAA 3' | :::: ::: :::::::::: | query 3' UUCAAGUUCUUUCGACACCUU 5' |
| SI08_SC00 | osa-miR396c | -1.27383 | 457.3486 | 1107.297 | BGIOSGA037400 | 1.77602 | 2.424808 | 0 | Putative uncharacterized protein [Source:UniProtKB/TrEMBL;Acc:A2ZKJ3] | 4 | 576-597 | target 5' CCGUUCAAGAAAGCAUGUGGAA 3' | :: :::::::::: ::::::: | query 3' UUCAAGUUCUUUCG-ACACCUU 5' |
| SI08_SC00 | osa-miR396c | -1.27383 | 457.3486 | 1107.297 | BGIOSGA014417 | 1.69755 | 80.82693 | 24.22802 | Putative uncharacterized protein [Source:UniProtKB/TrEMBL;Acc:B8ATM9] | 4 | 450-471 | target 5' CCGUUCAAGAAAGCCUGUGGAA 3' | :: ::::::::: :::::::: | query 3' UUCAAGUUCUUUC-GACACCUU 5' |
| SI08_SC00 | osa-miR396d | -1.1783 | 10486.57 | 23733.36 | BGIOSGA014417 | 1.69755 | 80.82693 | 24.22802 | Putative uncharacterized protein [Source:UniProtKB/TrEMBL;Acc:B8ATM9] | 1 | 450-470 | target 5' CCGUUCAAGAAAGCCUGUGGA 3' | : :::: :::::::::::::: | query 3' GUCAAGUUCUUUCGGACACCU 5' |
| SI08_SC00 | osa-miR396d | -1.1783 | 10486.57 | 23733.36 | BGIOSGA037400 | 1.77602 | 2.424808 | 0 | Putative uncharacterized protein [Source:UniProtKB/TrEMBL;Acc:A2ZKJ3] | 3 | 576-596 | target 5' CCGUUCAAGAAAGCAUGUGGA 3' | : :::: :::::::: :::::: | query 3' GUCAAGUUCUUUCGGACACCU 5' |
| SI08_SC00 | osa-miR396d | -1.1783 | 10486.57 | 23733.36 | BGIOSGA034580 | 1.77602 | 2.424808 | 0 | Putative uncharacterized protein [Source:UniProtKB/TrEMBL;Acc:A2ZB08] | 4 | 966-985 | target 5' CA-UUGAAGAAAGCCUCUGGA 3' | :: :: :::::::::: :::: | query 3' GUCAAGUUCUUUCGGACACCU 5' |
| SI08_SC00 | osa-miR396e | -1.1783 | 10486.57 | 23733.36 | BGIOSGA014417 | 1.69755 | 80.82693 | 24.22802 | Putative uncharacterized protein [Source:UniProtKB/TrEMBL;Acc:B8ATM9] | 1 | 450-470 | target 5' CCGUUCAAGAAAGCCUGUGGA 3' | : :::: :::::::::::::: | query 3' GUCAAGUUCUUUCGGACACCU 5' |
| SI08_SC00 | osa-miR396e | -1.1783 | 10486.57 | 23733.36 | BGIOSGA037400 | 1.77602 | 2.424808 | 0 | Putative uncharacterized protein [Source:UniProtKB/TrEMBL;Acc:A2ZKJ3] | 3 | 576-596 | target 5' CCGUUCAAGAAAGCAUGUGGA 3' | : :::: :::::::: :::::: | query 3' GUCAAGUUCUUUCGGACACCU 5' |
| SI08_SC00 | osa-miR396e | -1.1783 | 10486.57 | 23733.36 | BGIOSGA034580 | 1.77602 | 2.424808 | 0 | Putative uncharacterized protein [Source:UniProtKB/TrEMBL;Acc:A2ZB08] | 4 | 966-985 | target 5' CA-UUGAAGAAAGCCUCUGGA 3' | :: :: :::::::::: :::: | query 3' GUCAAGUUCUUUCGGACACCU 5' |
| SI08_SC00 | osa-miR396f | -1.16678 | 704.3378 | 1582.549 | BGIOSGA014417 | 1.69755 | 80.82693 | 24.22802 | Putative uncharacterized protein [Source:UniProtKB/TrEMBL;Acc:B8ATM9] | 3.5 | 451-472 | target 5' CGUUCAAGAAAGCCUGUGGAAG 3' | :::: :::::::::::::: . | query 3' UCAAGUUCUUUCGGACACCUCU 5' |
| SI08_SC00 | osa-miR396g | -1.17799 | 10287.73 | 23278.42 | BGIOSGA014417 | 1.69755 | 80.82693 | 24.22802 | Putative uncharacterized protein [Source:UniProtKB/TrEM | 0 | 450-470 | target 5' CCGUUCAAGAAAGCCUGUGGA 3' | :::::: :::::::::::::: | query 3' GGCAAGUUCUUUCGGACACCU 5' |

| | | | | | | | | | | | | | | |
|---|---|---|---|---|---|---|---|---|---|---|---|---|---|---|
| | | | | | | | | | BL;Acc:B8ATM9] | | | | | |
| SI08_SC00 | osa-miR396g | -1.17799 | 10287.73 | 23278.42 | BGIOSGA037400 | 1.77602 | 2.424808 | 0 | Putative uncharacterized protein [Source:UniProtKB/TrEMBL;Acc:A2ZKJ3] | 2 | 576-596 | target 5' CCGUUCAAGAAAGCAUGUGGA 3' | :::::: :::::::: :::::: | query 3' GGCAAGUUCUUUCGGACACCU 5' |
| SI08_SC00 | osa-miR396h | -1.17799 | 10287.73 | 23278.42 | BGIOSGA014417 | 1.69755 | 80.82693 | 24.22802 | Putative uncharacterized protein [Source:UniProtKB/TrEMBL;Acc:B8ATM9] | 0 | 450-470 | target 5' CCGUUCAAGAAAGCCUGUGGA 3' | :::::: ::::::::::::::: | query 3' GGCAAGUUCUUUCGGACACCU 5' |
| SI08_SC00 | osa-miR396h | -1.17799 | 10287.73 | 23278.42 | BGIOSGA037400 | 1.77602 | 2.424808 | 0 | Putative uncharacterized protein [Source:UniProtKB/TrEMBL;Acc:A2ZKJ3] | 2 | 576-596 | target 5' CCGUUCAAGAAAGCAUGUGGA 3' | :::::: :::::::: :::::: | query 3' GGCAAGUUCUUUCGGACACCU 5' |
| SI08_SC00 | osa-miR396i | -1.17799 | 10287.73 | 23278.42 | BGIOSGA014417 | 1.69755 | 80.82693 | 24.22802 | Putative uncharacterized protein [Source:UniProtKB/TrEMBL;Acc:B8ATM9] | 0 | 450-470 | target 5' CCGUUCAAGAAAGCCUGUGGA 3' | :::::: ::::::::::::::: | query 3' GGCAAGUUCUUUCGGACACCU 5' |
| SI08_SC00 | osa-miR396i | -1.17799 | 10287.73 | 23278.42 | BGIOSGA037400 | 1.77602 | 2.424808 | 0 | Putative uncharacterized protein [Source:UniProtKB/TrEMBL;Acc:A2ZKJ3] | 2 | 576-596 | target 5' CCGUUCAAGAAAGCAUGUGGA 3' | :::::: :::::::: :::::: | query 3' GGCAAGUUCUUUCGGACACCU 5' |
| SI08_SC00 | osa-miR444b.1 | -1.09026 | 677.1271 | 1442.817 | BGIOSGA008494 | 2.27952 | 14.54885 | 2.202547 | Putative uncharacterized protein [Source:UniProtKB/TrEMBL;Acc:B8AED3] | 0 | 276-296 | target 5' GGCAGCAAGCUUGAGACAACA 3' | :::::: ::::::::::::::: | query 3' CCGUCGUUCGAACUCUGUUGU 5' |
| SI08_SC00 | osa-miR444b.1 | -1.09026 | 677.1271 | 1442.817 | BGIOSGA018711 | 1.62689 | 8.890962 | 2.202547 | Putative uncharacterized protein [Source:UniProtKB/TrEMBL;Acc:B8AYM0] | 4 | 214-234 | target 5' GGCGGCAAGCUUGCGACAGCG 3' | :::.:: ::::::: ::::.:. | query 3' CCGUCGUUCGAACUCUGUUGU 5' |
| SI08_SC00 | osa-miR444b.2 | -1.00775 | 3830.425 | 7703.147 | BGIOSGA008494 | 2.27952 | 14.54885 | 2.202547 | Putative uncharacterized protein [Source:UniProtKB/TrEMBL;Acc:B8AED3] | 0 | 282-302 | target 5' AAGCUUGAGACAACAACUGCA 3' | :::::: ::::::::::::::: | query 3' UUCGAACUCUGUUGUUGACGU 5' |
| SI08_SC00 | osa-miR444b.2 | -1.00775 | 3830.425 | 7703.147 | BGIOSGA013971 | 2.00594 | 1568.851 | 389.8508 | Putative uncharacterized protein [Source:UniProtKB/TrEMBL;Acc:A2XP79] | 3.5 | 295-314 | target 5' AGGC-UGAGACAACAACAGCA 3' | :.:: : :::::::::: ::: | query 3' UUCGAACUCUGUUGUUGACGU 5' |
| SI08_SC00 | osa-miR444b.2 | -1.00775 | 3830.425 | 7703.147 | BGIOSGA030482 | 2.20878 | 319.2664 | 68.27896 | Putative uncharacterized protein [Source:UniProtKB/TrEMBL;Acc:B8BE73] | 4 | 1687-1707 | target 5' AGCCUUGGGACAACAACUGUC 3' | :. ::: :.:::::::::::. | query 3' UUCGAACUCUGUUGUUGACGU 5' |
| SI08_SC00 | osa-miR444c.1 | -1.09026 | 677.1271 | 1442.817 | BGIOSGA008494 | 2.27952 | 14.54885 | 2.202547 | Putative uncharacterized protein [Source:UniProtKB/TrEMBL;Acc:B8AED3] | 0 | 276-296 | target 5' GGCAGCAAGCUUGAGACAACA 3' | :::::: ::::::::::::::: | query 3' CCGUCGUUCGAACUCUGUUGU 5' |
| SI08_SC00 | osa-miR444c.1 | -1.09026 | 677.1271 | 1442.817 | BGIOSGA018711 | 1.62689 | 8.890962 | 2.202547 | Putative uncharacterized protein [Source:UniProtKB/TrEMBL;Acc:B8AYM0] | 4 | 214-234 | target 5' GGCGGCAAGCUUGCGACAGCG 3' | :::.:: ::::::: ::::.:. | query 3' CCGUCGUUCGAACUCUGUUGU 5' |
| SI08_SC00 | osa-miR444c.2 | -1.00775 | 3830.425 | 7703.147 | BGIOSGA008494 | 2.27952 | 14.54885 | 2.202547 | Putative uncharacterized protein [Source:UniProtKB/TrEMBL;Acc:B8AED3] | 0 | 282-302 | target 5' AAGCUUGAGACAACAACUGCA 3' | :::::: ::::::::::::::: | query 3' UUCGAACUCUGUUGUUGACGU 5' |
| SI08_SC00 | osa-miR444c.2 | -1.00775 | 3830.425 | 7703.147 | BGIOSGA013971 | 2.00594 | 1568.851 | 389.8508 | Putative uncharacterized protein [Source:UniProtKB/TrEM | 3.5 | 295-314 | target 5' AGGC-UGAGACAACAACAGCA 3' | :.:: : :::::::::: ::: | query 3' UUCGAACUCUGUUGUUGACGU 5' |

| | | | | | | | | | | | | | | |
|---|---|---|---|---|---|---|---|---|---|---|---|---|---|---|
| | | | | | | | | | BL;Acc:A2XP79] | | | | | |
| SI08_SC00 | osa-miR444c.2 | -1.00775 | 3830.425 | 7703.147 | BGIOSGA030482 | 2.20878 | 319.2664 | 68.27896 | Putative uncharacterized protein [Source:UniProtKB/TrEMBL;Acc:B8BE73] | 4 | 1687-1707 | target 5' AGCCUUGGGACAACAACUGUC 3' | :. ::: :.:::::::::::. | query 3' UUCGAACUCUGUUGUUGACGU 5' |
| SI08_SC00 | osa-miR444d.3 | -1.10466 | 42.90914 | 93.42564 | BGIOSGA016630 | 1.23474 | 369.379 | 156.3808 | Putative uncharacterized protein [Source:UniProtKB/TrEMBL;Acc:A2XUT7] | 4 | 576-595 | target 5' AAACUU-CAAGAAAGCUGCAA 3' | :::: : ::::::::..::: | query 3' GUUGAACGUUCUUUCGGUGUU 5' |
| SI08_SC00 | osa-miR528 | 3.27868 | 10131.79 | 1043.118 | BGIOSGA024054 | -2.44554 | 15.35712 | 88.10189 | Putative uncharacterized protein [Source:UniProtKB/TrEMBL;Acc:B8B7J3] | 2 | 378-397 | target 5' CUCCUCUGC-UGCCCCUUCCA 3' | :::::: ::: :::::::::: | query 3' GAGGAGACGUACGGGGAAGGU 5' |
| SI08_SC00 | osa-miR528 | 3.27868 | 10131.79 | 1043.118 | BGIOSGA009946 | -2.93966 | 8.890962 | 74.8866 | Putative uncharacterized protein [Source:UniProtKB/TrEMBL;Acc:A2XL83] | 4 | 61-80 | target 5' CUCCUCUGCGUGCUCC-UCCA 3' | :::::: :::.:::.:: :::: | query 3' GAGGAGACGUACGGGGAAGGU 5' |
| SI08_SC00 | osa-miR529b | 1.78068 | 38.72288 | 10.56116 | BGIOSGA029541 | -1.75659 | 6.466154 | 24.22802 | Putative uncharacterized protein [Source:UniProtKB/TrEMBL;Acc:B8BD46] | 1.5 | 573-593 | target 5' CAGCUGUGCUCUCUCUCUUCU 3' | :::: ::.::::::::::::: | query 3' UUCGACAUGAGAGAGAGAAGA 5' |
| SI08_SC00 | osa-miR529b | 1.78068 | 38.72288 | 10.56116 | BGIOSGA007643 | -1.64885 | 32.33077 | 103.5197 | Putative uncharacterized protein [Source:UniProtKB/TrEMBL;Acc:A2X1G6] | 2.5 | 712-731 | target 5' CAGC-GUGCUCUCUCUCUUCU 3' | ::: ::.::::::::::::: | query 3' UUCGACAUGAGAGAGAGAAGA 5' |
| SI08_SC08 | osa-miR159a.1 | -2.0501 | 20752.32 | 85945.69 | BGIOSGA037152 | 1.62632 | 2466.838 | 798.3633 | Putative uncharacterized protein [Source:UniProtKB/TrEMBL;Acc:B8BNM4] | 3.5 | 966-985 | target 5' UAG-UCUCCCUUCAAUUCAAA 3' | .:: :::::::::::.:::: | query 3' GUCUCGAGGGAAGUUAGGUUU 5' |
| SI08_SC08 | osa-miR159a.2 | -2.45633 | 34.53662 | 194.032 | BGIOSGA010571 | 1.42649 | 126.09 | 46.28193 | Putative uncharacterized protein [Source:UniProtKB/TrEMBL;Acc:B8AQZ1] | 4 | 438-458 | target 5' UGCAGCUCCUGGUGCAUCCAA 3' | :::::: :::::: :::: ::: | query 3' ACGUCGAGGACCCCGUACGUU 5' |
| SI08_SC08 | osa-miR159b | -2.0501 | 20752.32 | 85945.69 | BGIOSGA037152 | 1.62632 | 2466.838 | 798.3633 | Putative uncharacterized protein [Source:UniProtKB/TrEMBL;Acc:B8BNM4] | 3.5 | 966-985 | target 5' UAG-UCUCCCUUCAAUUCAAA 3' | .:: :::::::::::.:::: | query 3' GUCUCGAGGGAAGUUAGGUUU 5' |
| SI08_SC08 | osa-miR159f | -2.07343 | 19995.66 | 84162.21 | BGIOSGA037152 | 1.62632 | 2466.838 | 798.3633 | Putative uncharacterized protein [Source:UniProtKB/TrEMBL;Acc:B8BNM4] | 4 | 966-985 | target 5' UAG-UCUCCCUUCAAUUCAAA 3' | ::: :::::::::::.::: | query 3' AUCUCGAGGGAAGUUAGGUUC 5' |
| SI08_SC08 | osa-miR1862f | -1.78909 | 220.8251 | 765.6181 | BGIOSGA005796 | 2.99823 | 370.1873 | 45.45547 | Putative uncharacterized protein [Source:UniProtKB/TrEMBL;Acc:B8AHB3] | 3 | 1138-1156 | target 5' UCAAAG-AAACCAACUUCAU 3' | .::::. ::::::::.:::: | query 3' GGUUUUAUUUGGUUGGAGUA 5' |
| SI08_SC08 | osa-miR1862f | -1.78909 | 220.8251 | 765.6181 | BGIOSGA014360 | 1.00751 | 321.6912 | 159.5074 | Putative uncharacterized protein [Source:UniProtKB/TrEMBL;Acc:B8AU52] | 4 | 302-321 | target 5' CUGCAAGAAACCAAUCUCAU 3' | :.. :: :::::::.::::: | query 3' GGUUUUAUUUGGUUGGAGUA 5' |
| SI08_SC08 | osa-miR1862g | -1.78909 | 220.8251 | 765.6181 | BGIOSGA005796 | 2.99823 | 370.1873 | 45.45547 | Putative uncharacterized protein [Source:UniProtKB/TrEMBL;Acc:B8AHB3] | 3 | 1138-1156 | target 5' UCAAAG-AAACCAACUUCAU 3' | .::::. ::::::::.:::: | query 3' GGUUUUAUUUGGUUGGAGUA 5' |
| SI08_SC08 | osa-miR1862g | -1.78909 | 220.8251 | 765.6181 | BGIOSGA014360 | 1.00751 | 321.6912 | 159.5074 | Putative uncharacterized protein [Source:UniProtKB/TrEMBL | 4 | 302-321 | target 5' CUGCAAGAAACCAAUCUCAU 3' | :.. :: :::::::.::::: | query 3' GGUUUUAUUUGGUUGGAGUA 5' |

| | | | | | | | | | | | | | | |
|---|---|---|---|---|---|---|---|---|---|---|---|---|---|---|
| | | | | | | | | | BL;Acc:B8AU52] | | | | | |
| SI08_SC08 | osa-miR396f | -1.30405 | 704.3378 | 1740.629 | BGIOSGA014417 | 1.03822 | 80.82693 | 38.84376 | Putative uncharacterized protein [Source:UniProtKB/TrEMBL;Acc:B8ATM9] | 3.5 | 451-472 | target 5' CGUUCAAGAAAGCCUGUGGAAG 3' | :::: :::::::::::::: . | query 3' UCAAGUUCUUUCGGACACCUCU 5' |
| SI08_SC08 | osa-miR399d | 1.22424 | 758.7591 | 324.1952 | BGIOSGA017239 | -2.07528 | 60.62019 | 258.6829 | Putative uncharacterized protein [Source:UniProtKB/TrEMBL;Acc:B8AV88] | 3 | 3888-3907 | target 5' UA-GCCAGCUCUCCUUUGGCA 3' | .: : : :.::::::::::::: | query 3' GUCCCGUUGAGAGGAAACCGU 5' |
| SI08_SC08 | osa-miR399j | 1.12906 | 685.4996 | 312.8767 | BGIOSGA017239 | -2.07528 | 60.62019 | 258.6829 | Putative uncharacterized protein [Source:UniProtKB/TrEMBL;Acc:B8AV88] | 2.5 | 3888-3907 | target 5' UA-GCCAGCUCUCCUUUGGCA 3' | :: : : :.::::::::::::: | query 3' AUCCCGUUGAGAGGAAACCGU 5' |
| SI08_SC08 | osa-miR811d.1 | -2.62951 | 57.56104 | 361.3847 | BGIOSGA005057 | 1.38429 | 8.082693 | 2.479389 | Putative uncharacterized protein [Source:UniProtKB/TrEMBL;Acc:B8A8I0] | 3 | 367-387 | target 5' UGUACAACCGAGAUCUGUCCA 3' | :: ::: ::::::::.::::: | query 3' ACCUGUAGGCUCUAGGCAGGU 5' |
| SI08_SC08 | osa-miR811d.1 | -2.62951 | 57.56104 | 361.3847 | BGIOSGA005433 | 2.17961 | 7.274423 | 0.826463 | Putative uncharacterized protein [Source:UniProtKB/TrEMBL;Acc:A2XAK6] | 4 | 55-74 | target 5' UGGGUCUCCGAGAUCCG-CCA 3' | :::.. :::::::::: ::: | query 3' ACCUGUAGGCUCUAGGCAGGU 5' |
| SI08_SC08 | osa-miR827a | 1.359 | 8134.944 | 3170.807 | BGIOSGA007427 | -1.12535 | 160.8456 | 352.0733 | Putative uncharacterized protein [Source:UniProtKB/TrEMBL;Acc:B8AGY7] | 4 | 758-777 | target 5' UAUUUGC-CAUGGUCAUCUAA 3' | : :::: : ::::::::::: | query 3' ACAAACGACUACCAGUAGAUU 5' |
| SI08_SC08 | osa-miR827b | 1.359 | 8134.944 | 3170.807 | BGIOSGA007427 | -1.12535 | 160.8456 | 352.0733 | Putative uncharacterized protein [Source:UniProtKB/TrEMBL;Acc:B8AGY7] | 4 | 758-777 | target 5' UAUUUGC-CAUGGUCAUCUAA 3' | : :::: : ::::::::::: | query 3' ACAAACGACUACCAGUAGAUU 5' |
| SI24_SC00 | osa-miR1320 | 2.00631 | 19.33665 | 4.061984 | BGIOSGA014361 | -1.02589 | 17.8798 | 37.4433 | Putative uncharacterized protein [Source:UniProtKB/TrEMBL;Acc:B8AU51] | 3.5 | 175-195 | target 5' CCGCAUAAUUCCUCCGUUCCA 3' | : . : :::::::::::::: | query 3' GAUAUUUUAAGGAGGCAAGGU 5' |
| SI24_SC00 | osa-miR156a | 1.74994 | 378715 | 112596.6 | BGIOSGA029541 | -1.62981 | 7.151921 | 24.22802 | Putative uncharacterized protein [Source:UniProtKB/TrEMBL;Acc:B8BD46] | 1 | 578-597 | target 5' GUGCUCUCUCUCUUCUGUCA 3' | :::::: ::::::::::::: | query 3' CACGAGUGAGAGAAGACAGU 5' |
| SI24_SC00 | osa-miR156a | 1.74994 | 378715 | 112596.6 | BGIOSGA007643 | -1.29081 | 41.71954 | 103.5197 | Putative uncharacterized protein [Source:UniProtKB/TrEMBL;Acc:A2X1G6] | 1 | 716-735 | target 5' GUGCUCUCUCUCUUCUGUCA 3' | :::::: ::::::::::::: | query 3' CACGAGUGAGAGAAGACAGU 5' |
| SI24_SC00 | osa-miR156a | 1.74994 | 378715 | 112596.6 | BGIOSGA036252 | -2.46009 | 2.383974 | 17.62038 | Putative uncharacterized protein [Source:UniProtKB/TrEMBL;Acc:A2ZK57] | 4 | 232-250 | target 5' GUGCUCACUCUC-UCUGGCA 3' | :::::: :::::: :::: :: | query 3' CACGAGUGAGAGAAGACAGU 5' |
| SI24_SC00 | osa-miR156a | 1.74994 | 378715 | 112596.6 | BGIOSGA036253 | -2.69713 | 1.191987 | 13.21528 | Putative uncharacterized protein [Source:UniProtKB/TrEMBL;Acc:A2ZK56] | 4 | 232-250 | target 5' GUGCUCACUCUC-UCUGGCA 3' | :::::: :::::: :::: :: | query 3' CACGAGUGAGAGAAGACAGU 5' |
| SI24_SC00 | osa-miR156a | 1.74994 | 378715 | 112596.6 | BGIOSGA013937 | -1.16617 | 35.75961 | 81.49424 | Putative uncharacterized protein [Source:UniProtKB/TrEMBL;Acc:B8ANA2] | 4 | 655-673 | target 5' GCGC-CACUCUCUUCUGUGA 3' | : :: : :::::::::::: : | query 3' CACGAGUGAGAGAAGACAGU 5' |
| SI24_SC00 | osa-miR156b | 1.74994 | 378715 | 112596.6 | BGIOSGA029541 | -1.62981 | 7.151921 | 24.22802 | Putative uncharacterized protein [Source:UniProtKB/TrEM | 1 | 578-597 | target 5' GUGCUCUCUCUCUUCUGUCA 3' | :::::: ::::::::::::: | query 3' CACGAGUGAGAGAAGACAGU 5' |

| | | | | | | | | | | | | | | |
|---|---|---|---|---|---|---|---|---|---|---|---|---|---|---|
| | | | | | | | | | BL;Acc:B8BD46] | | | | | |
| SI24_SC00 | osa-miR156b | 1.74994 | 378715 | 112596.6 | BGIOSGA007643 | -1.29081 | 41.71954 | 103.5197 | Putative uncharacterized protein [Source:UniProtKB/TrEMBL;Acc:A2X1G6] | 1 | 716-735 | target 5' GUGCUCUCUCUCUUCUGUCA 3' | :::::: :::::::::::: | query 3' CACGAGUGAGAGAAGACAGU 5' |
| SI24_SC00 | osa-miR156b | 1.74994 | 378715 | 112596.6 | BGIOSGA036252 | -2.46009 | 2.383974 | 17.62038 | Putative uncharacterized protein [Source:UniProtKB/TrEMBL;Acc:A2ZK57] | 4 | 232-250 | target 5' GUGCUCACUCUC-UCUGGCA 3' | :::::: :::::: :::: :: | query 3' CACGAGUGAGAGAAGACAGU 5' |
| SI24_SC00 | osa-miR156b | 1.74994 | 378715 | 112596.6 | BGIOSGA036253 | -2.69713 | 1.191987 | 13.21528 | Putative uncharacterized protein [Source:UniProtKB/TrEMBL;Acc:A2ZK56] | 4 | 232-250 | target 5' GUGCUCACUCUC-UCUGGCA 3' | :::::: :::::: :::: :: | query 3' CACGAGUGAGAGAAGACAGU 5' |
| SI24_SC00 | osa-miR156b | 1.74994 | 378715 | 112596.6 | BGIOSGA013937 | -1.16617 | 35.75961 | 81.49424 | Putative uncharacterized protein [Source:UniProtKB/TrEMBL;Acc:B8ANA2] | 4 | 655-673 | target 5' GCGC-CACUCUCUUCUGUGA 3' | : :: : ::::::::::: : | query 3' CACGAGUGAGAGAAGACAGU 5' |
| SI24_SC00 | osa-miR156c | 1.74994 | 378715 | 112596.6 | BGIOSGA029541 | -1.62981 | 7.151921 | 24.22802 | Putative uncharacterized protein [Source:UniProtKB/TrEMBL;Acc:B8BD46] | 1 | 578-597 | target 5' GUGCUCUCUCUCUUCUGUCA 3' | :::::: :::::::::::: | query 3' CACGAGUGAGAGAAGACAGU 5' |
| SI24_SC00 | osa-miR156c | 1.74994 | 378715 | 112596.6 | BGIOSGA007643 | -1.29081 | 41.71954 | 103.5197 | Putative uncharacterized protein [Source:UniProtKB/TrEMBL;Acc:A2X1G6] | 1 | 716-735 | target 5' GUGCUCUCUCUCUUCUGUCA 3' | :::::: :::::::::::: | query 3' CACGAGUGAGAGAAGACAGU 5' |
| SI24_SC00 | osa-miR156c | 1.74994 | 378715 | 112596.6 | BGIOSGA036252 | -2.46009 | 2.383974 | 17.62038 | Putative uncharacterized protein [Source:UniProtKB/TrEMBL;Acc:A2ZK57] | 4 | 232-250 | target 5' GUGCUCACUCUC-UCUGGCA 3' | :::::: :::::: :::: :: | query 3' CACGAGUGAGAGAAGACAGU 5' |
| SI24_SC00 | osa-miR156c | 1.74994 | 378715 | 112596.6 | BGIOSGA036253 | -2.69713 | 1.191987 | 13.21528 | Putative uncharacterized protein [Source:UniProtKB/TrEMBL;Acc:A2ZK56] | 4 | 232-250 | target 5' GUGCUCACUCUC-UCUGGCA 3' | :::::: :::::: :::: :: | query 3' CACGAGUGAGAGAAGACAGU 5' |
| SI24_SC00 | osa-miR156c | 1.74994 | 378715 | 112596.6 | BGIOSGA013937 | -1.16617 | 35.75961 | 81.49424 | Putative uncharacterized protein [Source:UniProtKB/TrEMBL;Acc:B8ANA2] | 4 | 655-673 | target 5' GCGC-CACUCUCUUCUGUGA 3' | : :: : ::::::::::: : | query 3' CACGAGUGAGAGAAGACAGU 5' |
| SI24_SC00 | osa-miR156d | 1.74994 | 378715 | 112596.6 | BGIOSGA029541 | -1.62981 | 7.151921 | 24.22802 | Putative uncharacterized protein [Source:UniProtKB/TrEMBL;Acc:B8BD46] | 1 | 578-597 | target 5' GUGCUCUCUCUCUUCUGUCA 3' | :::::: :::::::::::: | query 3' CACGAGUGAGAGAAGACAGU 5' |
| SI24_SC00 | osa-miR156d | 1.74994 | 378715 | 112596.6 | BGIOSGA007643 | -1.29081 | 41.71954 | 103.5197 | Putative uncharacterized protein [Source:UniProtKB/TrEMBL;Acc:A2X1G6] | 1 | 716-735 | target 5' GUGCUCUCUCUCUUCUGUCA 3' | :::::: :::::::::::: | query 3' CACGAGUGAGAGAAGACAGU 5' |
| SI24_SC00 | osa-miR156d | 1.74994 | 378715 | 112596.6 | BGIOSGA036252 | -2.46009 | 2.383974 | 17.62038 | Putative uncharacterized protein [Source:UniProtKB/TrEMBL;Acc:A2ZK57] | 4 | 232-250 | target 5' GUGCUCACUCUC-UCUGGCA 3' | :::::: :::::: :::: :: | query 3' CACGAGUGAGAGAAGACAGU 5' |
| SI24_SC00 | osa-miR156d | 1.74994 | 378715 | 112596.6 | BGIOSGA036253 | -2.69713 | 1.191987 | 13.21528 | Putative uncharacterized protein [Source:UniProtKB/TrEMBL;Acc:A2ZK56] | 4 | 232-250 | target 5' GUGCUCACUCUC-UCUGGCA 3' | :::::: :::::: :::: :: | query 3' CACGAGUGAGAGAAGACAGU 5' |
| SI24_SC00 | osa-miR156d | 1.74994 | 378715 | 112596.6 | BGIOSGA013937 | -1.16617 | 35.75961 | 81.49424 | Putative uncharacterized protein [Source:UniProtKB/TrEM | 4 | 655-673 | target 5' GCGC-CACUCUCUUCUGUGA 3' | : :: : ::::::::::: : | query 3' CACGAGUGAGAGAAGACAGU 5' |

| | | | | | | | | | | | | | | |
|---|---|---|---|---|---|---|---|---|---|---|---|---|---|---|
| | | | | | | | | | BL;Acc:B8ANA2] | | | | | |
| SI24_S C00 | osa-miR1 56e | 1.74994 | 3787 15 | 1125 96.6 | BGIOSG A029541 | -1.6 2981 | 7.15 1921 | 24.2 2802 | Putative uncharacterized protein [Source:UniProtKB/TrEM BL;Acc:B8BD46] | 1 | 578-5 97 | target 5' GUGCUCUCUCUCU UCUGUCA 3' | :::::: ::::::::::::: | query 3' CACGAGUGAGAGAAGA CAGU 5' |
| SI24_S C00 | osa-miR1 56e | 1.74994 | 3787 15 | 1125 96.6 | BGIOSG A007643 | -1.2 9081 | 41.7 1954 | 103. 5197 | Putative uncharacterized protein [Source:UniProtKB/TrEM BL;Acc:A2X1G6] | 1 | 716-7 35 | target 5' GUGCUCUCUCUCU UCUGUCA 3' | :::::: ::::::::::::: | query 3' CACGAGUGAGAGAAGA CAGU 5' |
| SI24_S C00 | osa-miR1 56e | 1.74994 | 3787 15 | 1125 96.6 | BGIOSG A036252 | -2.4 6009 | 2.38 3974 | 17.6 2038 | Putative uncharacterized protein [Source:UniProtKB/TrEM BL;Acc:A2ZK57] | 4 | 232-2 50 | target 5' GUGCUCACUCUC-U CUGGCA 3' | :::::: :::::: :::: :: | query 3' CACGAGUGAGAGAAGA CAGU 5' |
| SI24_S C00 | osa-miR1 56e | 1.74994 | 3787 15 | 1125 96.6 | BGIOSG A036253 | -2.6 9713 | 1.19 1987 | 13.2 1528 | Putative uncharacterized protein [Source:UniProtKB/TrEM BL;Acc:A2ZK56] | 4 | 232-2 50 | target 5' GUGCUCACUCUC-U CUGGCA 3' | :::::: :::::: :::: :: | query 3' CACGAGUGAGAGAAGA CAGU 5' |
| SI24_S C00 | osa-miR1 56e | 1.74994 | 3787 15 | 1125 96.6 | BGIOSG A013937 | -1.1 6617 | 35.7 5961 | 81.4 9424 | Putative uncharacterized protein [Source:UniProtKB/TrEM BL;Acc:B8ANA2] | 4 | 655-6 73 | target 5' GCGC-CACUCUCUU CUGUGA 3' | : :: : :::::::::::: : | query 3' CACGAGUGAGAGAAGA CAGU 5' |
| SI24_S C00 | osa-miR1 56f | 1.74994 | 3787 15 | 1125 96.6 | BGIOSG A029541 | -1.6 2981 | 7.15 1921 | 24.2 2802 | Putative uncharacterized protein [Source:UniProtKB/TrEM BL;Acc:B8BD46] | 1 | 578-5 97 | target 5' GUGCUCUCUCUCU UCUGUCA 3' | :::::: ::::::::::::: | query 3' CACGAGUGAGAGAAGA CAGU 5' |
| SI24_S C00 | osa-miR1 56f | 1.74994 | 3787 15 | 1125 96.6 | BGIOSG A007643 | -1.2 9081 | 41.7 1954 | 103. 5197 | Putative uncharacterized protein [Source:UniProtKB/TrEM BL;Acc:A2X1G6] | 1 | 716-7 35 | target 5' GUGCUCUCUCUCU UCUGUCA 3' | :::::: ::::::::::::: | query 3' CACGAGUGAGAGAAGA CAGU 5' |
| SI24_S C00 | osa-miR1 56f | 1.74994 | 3787 15 | 1125 96.6 | BGIOSG A036252 | -2.4 6009 | 2.38 3974 | 17.6 2038 | Putative uncharacterized protein [Source:UniProtKB/TrEM BL;Acc:A2ZK57] | 4 | 232-2 50 | target 5' GUGCUCACUCUC-U CUGGCA 3' | :::::: :::::: :::: :: | query 3' CACGAGUGAGAGAAGA CAGU 5' |
| SI24_S C00 | osa-miR1 56f | 1.74994 | 3787 15 | 1125 96.6 | BGIOSG A036253 | -2.6 9713 | 1.19 1987 | 13.2 1528 | Putative uncharacterized protein [Source:UniProtKB/TrEM BL;Acc:A2ZK56] | 4 | 232-2 50 | target 5' GUGCUCACUCUC-U CUGGCA 3' | :::::: :::::: :::: :: | query 3' CACGAGUGAGAGAAGA CAGU 5' |
| SI24_S C00 | osa-miR1 56f | 1.74994 | 3787 15 | 1125 96.6 | BGIOSG A013937 | -1.1 6617 | 35.7 5961 | 81.4 9424 | Putative uncharacterized protein [Source:UniProtKB/TrEM BL;Acc:B8ANA2] | 4 | 655-6 73 | target 5' GCGC-CACUCUCUU CUGUGA 3' | : :: : :::::::::::: : | query 3' CACGAGUGAGAGAAGA CAGU 5' |
| SI24_S C00 | osa-miR1 56g | 1.74994 | 3787 15 | 1125 96.6 | BGIOSG A029541 | -1.6 2981 | 7.15 1921 | 24.2 2802 | Putative uncharacterized protein [Source:UniProtKB/TrEM BL;Acc:B8BD46] | 1 | 578-5 97 | target 5' GUGCUCUCUCUCU UCUGUCA 3' | :::::: ::::::::::::: | query 3' CACGAGUGAGAGAAGA CAGU 5' |
| SI24_S C00 | osa-miR1 56g | 1.74994 | 3787 15 | 1125 96.6 | BGIOSG A007643 | -1.2 9081 | 41.7 1954 | 103. 5197 | Putative uncharacterized protein [Source:UniProtKB/TrEM BL;Acc:A2X1G6] | 1 | 716-7 35 | target 5' GUGCUCUCUCUCU UCUGUCA 3' | :::::: ::::::::::::: | query 3' CACGAGUGAGAGAAGA CAGU 5' |
| SI24_S C00 | osa-miR1 56g | 1.74994 | 3787 15 | 1125 96.6 | BGIOSG A036252 | -2.4 6009 | 2.38 3974 | 17.6 2038 | Putative uncharacterized protein [Source:UniProtKB/TrEM BL;Acc:A2ZK57] | 4 | 232-2 50 | target 5' GUGCUCACUCUC-U CUGGCA 3' | :::::: :::::: :::: :: | query 3' CACGAGUGAGAGAAGA CAGU 5' |
| SI24_S C00 | osa-miR1 56g | 1.74994 | 3787 15 | 1125 96.6 | BGIOSG A036253 | -2.6 9713 | 1.19 1987 | 13.2 1528 | Putative uncharacterized protein [Source:UniProtKB/TrEM | 4 | 232-2 50 | target 5' GUGCUCACUCUC-U CUGGCA 3' | :::::: :::::: :::: :: | query 3' CACGAGUGAGAGAAGA CAGU 5' |

| | | | | | | | | | | | | | | |
|---|---|---|---|---|---|---|---|---|---|---|---|---|---|---|
| | | | | | | | | | BL;Acc:A2ZK56] | | | | | |
| SI24_S C00 | osa-miR1 56g | 1.74994 | 3787 15 | 1125 96.6 | BGIOSG A013937 | -1.1 6617 | 35.7 5961 | 81.4 9424 | Putative uncharacterized protein [Source:UniProtKB/TrEMBL;Acc:B8ANA2] | 4 | 655-6 73 | target 5' GCGC-CACUCUCUU CUGUGA 3' | : :: : :::::::::::: : | query 3' CACGAGUGAGAGAAGA CAGU 5' |
| SI24_S C00 | osa-miR1 56h | 1.74994 | 3787 15 | 1125 96.6 | BGIOSG A029541 | -1.6 2981 | 7.15 1921 | 24.2 2802 | Putative uncharacterized protein [Source:UniProtKB/TrEMBL;Acc:B8BD46] | 1 | 578-5 97 | target 5' GUGCUCUCUCUCU UCUGUCA 3' | :::::: :::::::::::: | query 3' CACGAGUGAGAGAAGA CAGU 5' |
| SI24_S C00 | osa-miR1 56h | 1.74994 | 3787 15 | 1125 96.6 | BGIOSG A007643 | -1.2 9081 | 41.7 1954 | 103. 5197 | Putative uncharacterized protein [Source:UniProtKB/TrEMBL;Acc:A2X1G6] | 1 | 716-7 35 | target 5' GUGCUCUCUCUCU UCUGUCA 3' | :::::: :::::::::::: | query 3' CACGAGUGAGAGAAGA CAGU 5' |
| SI24_S C00 | osa-miR1 56h | 1.74994 | 3787 15 | 1125 96.6 | BGIOSG A036252 | -2.4 6009 | 2.38 3974 | 17.6 2038 | Putative uncharacterized protein [Source:UniProtKB/TrEMBL;Acc:A2ZK57] | 4 | 232-2 50 | target 5' GUGCUCACUCUC-U CUGGCA 3' | :::::: :::::: :::: :: | query 3' CACGAGUGAGAGAAGA CAGU 5' |
| SI24_S C00 | osa-miR1 56h | 1.74994 | 3787 15 | 1125 96.6 | BGIOSG A036253 | -2.6 9713 | 1.19 1987 | 13.2 1528 | Putative uncharacterized protein [Source:UniProtKB/TrEMBL;Acc:A2ZK56] | 4 | 232-2 50 | target 5' GUGCUCACUCUC-U CUGGCA 3' | :::::: :::::: :::: :: | query 3' CACGAGUGAGAGAAGA CAGU 5' |
| SI24_S C00 | osa-miR1 56h | 1.74994 | 3787 15 | 1125 96.6 | BGIOSG A013937 | -1.1 6617 | 35.7 5961 | 81.4 9424 | Putative uncharacterized protein [Source:UniProtKB/TrEMBL;Acc:B8ANA2] | 4 | 655-6 73 | target 5' GCGC-CACUCUCUU CUGUGA 3' | : :: : :::::::::::: : | query 3' CACGAGUGAGAGAAGA CAGU 5' |
| SI24_S C00 | osa-miR1 56i | 1.74994 | 3787 15 | 1125 96.6 | BGIOSG A029541 | -1.6 2981 | 7.15 1921 | 24.2 2802 | Putative uncharacterized protein [Source:UniProtKB/TrEMBL;Acc:B8BD46] | 1 | 578-5 97 | target 5' GUGCUCUCUCUCU UCUGUCA 3' | :::::: :::::::::::: | query 3' CACGAGUGAGAGAAGA CAGU 5' |
| SI24_S C00 | osa-miR1 56i | 1.74994 | 3787 15 | 1125 96.6 | BGIOSG A007643 | -1.2 9081 | 41.7 1954 | 103. 5197 | Putative uncharacterized protein [Source:UniProtKB/TrEMBL;Acc:A2X1G6] | 1 | 716-7 35 | target 5' GUGCUCUCUCUCU UCUGUCA 3' | :::::: :::::::::::: | query 3' CACGAGUGAGAGAAGA CAGU 5' |
| SI24_S C00 | osa-miR1 56i | 1.74994 | 3787 15 | 1125 96.6 | BGIOSG A036252 | -2.4 6009 | 2.38 3974 | 17.6 2038 | Putative uncharacterized protein [Source:UniProtKB/TrEMBL;Acc:A2ZK57] | 4 | 232-2 50 | target 5' GUGCUCACUCUC-U CUGGCA 3' | :::::: :::::: :::: :: | query 3' CACGAGUGAGAGAAGA CAGU 5' |
| SI24_S C00 | osa-miR1 56i | 1.74994 | 3787 15 | 1125 96.6 | BGIOSG A036253 | -2.6 9713 | 1.19 1987 | 13.2 1528 | Putative uncharacterized protein [Source:UniProtKB/TrEMBL;Acc:A2ZK56] | 4 | 232-2 50 | target 5' GUGCUCACUCUC-U CUGGCA 3' | :::::: :::::: :::: :: | query 3' CACGAGUGAGAGAAGA CAGU 5' |
| SI24_S C00 | osa-miR1 56i | 1.74994 | 3787 15 | 1125 96.6 | BGIOSG A013937 | -1.1 6617 | 35.7 5961 | 81.4 9424 | Putative uncharacterized protein [Source:UniProtKB/TrEMBL;Acc:B8ANA2] | 4 | 655-6 73 | target 5' GCGC-CACUCUCUU CUGUGA 3' | : :: : :::::::::::: : | query 3' CACGAGUGAGAGAAGA CAGU 5' |
| SI24_S C00 | osa-miR1 56j | 1.74994 | 3787 15 | 1125 96.6 | BGIOSG A029541 | -1.6 2981 | 7.15 1921 | 24.2 2802 | Putative uncharacterized protein [Source:UniProtKB/TrEMBL;Acc:B8BD46] | 1 | 578-5 97 | target 5' GUGCUCUCUCUCU UCUGUCA 3' | :::::: :::::::::::: | query 3' CACGAGUGAGAGAAGA CAGU 5' |
| SI24_S C00 | osa-miR1 56j | 1.74994 | 3787 15 | 1125 96.6 | BGIOSG A007643 | -1.2 9081 | 41.7 1954 | 103. 5197 | Putative uncharacterized protein [Source:UniProtKB/TrEMBL;Acc:A2X1G6] | 1 | 716-7 35 | target 5' GUGCUCUCUCUCU UCUGUCA 3' | :::::: :::::::::::: | query 3' CACGAGUGAGAGAAGA CAGU 5' |
| SI24_S C00 | osa-miR1 56j | 1.74994 | 3787 15 | 1125 96.6 | BGIOSG A036252 | -2.4 6009 | 2.38 3974 | 17.6 2038 | Putative uncharacterized protein [Source:UniProtKB/TrEM | 4 | 232-2 50 | target 5' GUGCUCACUCUC-U CUGGCA 3' | :::::: :::::: :::: :: | query 3' CACGAGUGAGAGAAGA CAGU 5' |

| | | | | | | | | | | | | | | |
|---|---|---|---|---|---|---|---|---|---|---|---|---|---|---|
| | | | | | | | | | BL;Acc:A2ZK57] | | | | | |
| SI24_SC00 | osa-miR156j | 1.74994 | 378715 | 112596.6 | BGIOSGA036253 | -2.69713 | 1.191987 | 13.21528 | Putative uncharacterized protein [Source:UniProtKB/TrEMBL;Acc:A2ZK56] | 4 | 232-250 | target 5' GUGCUCACUCUC-UCUGGCA 3' | :::::: :::::: :::: :: | query 3' CACGAGUGAGAGAAGACAGU 5' |
| SI24_SC00 | osa-miR156j | 1.74994 | 378715 | 112596.6 | BGIOSGA013937 | -1.16617 | 35.75961 | 81.49424 | Putative uncharacterized protein [Source:UniProtKB/TrEMBL;Acc:B8ANA2] | 4 | 655-673 | target 5' GCGC-CACUCUCUUCUGUGA 3' | : :: : ::::::::::: : | query 3' CACGAGUGAGAGAAGACAGU 5' |
| SI24_SC00 | osa-miR156k | 1.75177 | 373482.8 | 110900.3 | BGIOSGA029541 | -1.62981 | 7.151921 | 24.22802 | Putative uncharacterized protein [Source:UniProtKB/TrEMBL;Acc:B8BD46] | 0 | 577-597 | target 5' UGUGCUCUCUCUCUUCUGUCA 3' | :::::: :::::::::::::: | query 3' ACACGAGAGAGAGAAGACAGU 5' |
| SI24_SC00 | osa-miR156k | 1.75177 | 373482.8 | 110900.3 | BGIOSGA007643 | -1.29081 | 41.71954 | 103.5197 | Putative uncharacterized protein [Source:UniProtKB/TrEMBL;Acc:A2X1G6] | 1 | 715-735 | target 5' CGUGCUCUCUCUCUUCUGUCA 3' | :::: ::::::::::::::: | query 3' ACACGAGAGAGAGAAGACAGU 5' |
| SI24_SC00 | osa-miR156l | 1.47674 | 2337.459 | 839.206 | BGIOSGA029541 | -1.62981 | 7.151921 | 24.22802 | Putative uncharacterized protein [Source:UniProtKB/TrEMBL;Acc:B8BD46] | 2.5 | 577-597 | target 5' UGUGCUCUCUCUCUUCUGUCA 3' | :.::: : ::::::::::: | query 3' AUACGAGUGAGAGAAGACAGC 5' |
| SI24_SC00 | osa-miR156l | 1.47674 | 2337.459 | 839.206 | BGIOSGA007643 | -1.29081 | 41.71954 | 103.5197 | Putative uncharacterized protein [Source:UniProtKB/TrEMBL;Acc:A2X1G6] | 3.5 | 715-735 | target 5' CGUGCUCUCUCUCUUCUGUCA 3' | .::: :: ::::::::::: | query 3' AUACGAGUGAGAGAAGACAGC 5' |
| SI24_SC00 | osa-miR159a.2 | 1.47888 | 142.1812 | 50.36861 | BGIOSGA033431 | -1.67922 | 0 | 2.202547 | Putative uncharacterized protein [Source:UniProtKB/TrEMBL;Acc:B8BI71] | 4 | 1782-1802 | target 5' UGCAGUUCUUAGGGCAUGCAG 3' | :::::. ::.: :::::::::. | query 3' ACGUCGAGGACCCCGUACGUU 5' |
| SI24_SC00 | osa-miR1861d | -1.90968 | 11.3745 | 45.49423 | BGIOSGA017432 | 2.19407 | 3.575961 | 0 | Putative uncharacterized protein [Source:UniProtKB/TrEMBL;Acc:B8AXI3] | 4 | 491-512 | target 5' CCCAGUUUCUUCCUCAAGACCG 3' | : :::: :.:: ::::::::::. | query 3' GAGUCAAGGACGGAGUUCUGGU 5' |
| SI24_SC00 | osa-miR1861h | -1.80781 | 12.51195 | 46.30662 | BGIOSGA017432 | 2.19407 | 3.575961 | 0 | Putative uncharacterized protein [Source:UniProtKB/TrEMBL;Acc:B8AXI3] | 3.5 | 491-512 | target 5' CCCAGUUUCUUCCUCAAGACCG 3' | : :::: :.:: ::::::::::: | query 3' GAGUCAAGGACGGAGUUCUGGC 5' |
| SI24_SC00 | osa-miR1861j | -1.80781 | 12.51195 | 46.30662 | BGIOSGA017432 | 2.19407 | 3.575961 | 0 | Putative uncharacterized protein [Source:UniProtKB/TrEMBL;Acc:B8AXI3] | 3.5 | 491-512 | target 5' CCCAGUUUCUUCCUCAAGACCG 3' | : :::: :.:: ::::::::::: | query 3' GAGUCAAGGACGGAGUUCUGGC 5' |
| SI24_SC00 | osa-miR390 | 1.03813 | 272.9879 | 132.4207 | BGIOSGA033781 | -1.67922 | 0 | 2.202547 | Putative uncharacterized protein [Source:UniProtKB/TrEMBL;Acc:A2ZFH5] | 2.5 | 1033-1053 | target 5' GGCACUGUCCCUUCUGAGCUU 3' | ::: :: .:::::.:::::::: | query 3' CCGCGAUAGGGAGGACUCGAA 5' |
| SI24_SC00 | osa-miR399d | 1.15869 | 1025.98 | 459.0042 | BGIOSGA035900 | -2.46009 | 2.383974 | 17.62038 | Putative uncharacterized protein [Source:UniProtKB/TrEMBL;Acc:B8BMT6] | 4 | 2138-2157 | target 5' CA-GGCGGCUCUCAUUUGGCA 3' | :: ::: ..::::: :::::: | query 3' GUCCCGUUGAGAGGAAACCGU 5' |
| SI24_SC00 | osa-miR399e | 1.59625 | 97.82068 | 31.68348 | BGIOSGA029256 | -1.63311 | 1097.82 | 3407.34 | Putative uncharacterized protein [Source:UniProtKB/TrEMBL;Acc:B8BEL6] | 4 | 721-740 | target 5' GUGGGC-AAUCUCCCUUGGCA 3' | :::: : ::::::: :::::: | query 3' GACCCGUUUAGAGGAAACCGU 5' |
| SI24_SC00 | osa-miR399f | 1.59625 | 97.82068 | 31.68348 | BGIOSGA029256 | -1.63311 | 1097.82 | 3407.34 | Putative uncharacterized protein [Source:UniProtKB/TrEM | 4 | 721-740 | target 5' GUGGGC-AAUCUCCCUUGGCA 3' | :::: : ::::::: :::::: | query 3' GACCCGUUUAGAGGAAACCGU 5' |

| | | | | | | | | | | | | | | |
|---|---|---|---|---|---|---|---|---|---|---|---|---|---|---|
| | | | | | | | | | BL;Acc:B8BEL6] | | | | | |
| SI24_SC00 | osa-miR399g | 1.59625 | 97.82068 | 31.68348 | BGIOSGA029256 | -1.63311 | 1097.82 | 3407.34 | Putative uncharacterized protein [Source:UniProtKB/TrEMBL;Acc:B8BEL6] | 4 | 721-740 | target 5' GUGGGC-AAUCUCCCUUGGCA 3' | :::: : :::::: :::::: | query 3' GACCCGUUUAGAGGAAACCGU 5' |
| SI24_SC00 | osa-miR399i | 1.18164 | 705.2188 | 310.3356 | BGIOSGA035900 | -2.46009 | 2.383974 | 17.62038 | Putative uncharacterized protein [Source:UniProtKB/TrEMBL;Acc:B8BMT6] | 3.5 | 2138-2157 | target 5' CA-GGCGGCUCUCAUUUGGCA 3' | :: ::: .:::::: :::::: | query 3' GUCCCGUCGAGAGGAAACCGU 5' |
| SI24_SC00 | osa-miR5083 | -1.22772 | 13.6494 | 33.30827 | BGIOSGA007106 | 1.38452 | 13.11186 | 4.405094 | Putative uncharacterized protein [Source:UniProtKB/TrEMBL;Acc:A2X127] | 3 | 1947-1965 | target 5' UGAUCAGAUAGUUGUA-UCU 3' | :::::: ::::.:::: ::: | query 3' ACUAGUCUAUUAACAUCAGA 5' |
| SI24_SC00 | osa-miR529b | 1.77888 | 38.67329 | 10.56116 | BGIOSGA029541 | -1.62981 | 7.151921 | 24.22802 | Putative uncharacterized protein [Source:UniProtKB/TrEMBL;Acc:B8BD46] | 1.5 | 573-593 | target 5' CAGCUGUGCUCUCUCUCUUCU 3' | :::: ::.::::::::::: | query 3' UUCGACAUGAGAGAGAGAAGA 5' |
| SI24_SC00 | osa-miR529b | 1.77888 | 38.67329 | 10.56116 | BGIOSGA007643 | -1.29081 | 41.71954 | 103.5197 | Putative uncharacterized protein [Source:UniProtKB/TrEMBL;Acc:A2X1G6] | 2.5 | 712-731 | target 5' CAGC-GUGCUCUCUCUCUUCU 3' | ::: ::.::::::::::: | query 3' UUCGACAUGAGAGAGAGAAGA 5' |
| SI24_SC00 | osa-miR535 | 1.41229 | 3811.594 | 1431.443 | BGIOSGA007643 | -1.29081 | 41.71954 | 103.5197 | Putative uncharacterized protein [Source:UniProtKB/TrEMBL;Acc:A2X1G6] | 4 | 714-735 | target 5' GCGUGCUCUCUCUCUUCUGUCA 3' | :::::: :::::::: : ::::: | query 3' CGCACGAGAGAGAGCA-ACAGU 5' |
| SI24_SC00 | osa-miR811d.1 | 1.54142 | 280.9501 | 95.86283 | BGIOSGA021484 | -3.08657 | 1.191987 | 17.62038 | Putative uncharacterized protein [Source:UniProtKB/TrEMBL;Acc:A2YBI7] | 1.5 | 1667-1687 | target 5' UGGACGUCCGAGAUUCGUCCA 3' | :::::. ::::::::.:::::: | query 3' ACCUGUAGGCUCUAGGCAGGU 5' |
| SI24_SI08 | osa-miR1425-3p | 1.04951 | 1201.147 | 579.7966 | BGIOSGA029652 | -1.52361 | 8.343908 | 25.86462 | Putative uncharacterized protein [Source:UniProtKB/TrEMBL;Acc:B8BCJ5] | 2.5 | 774-793 | target 5' AUCAGGA-CCAGUUCUUGCUG 3' | :: :.: : :::::::::::: | query 3' UAAUUCUAGGUCAAGAACGAC 5' |
| SI24_SI08 | osa-miR1427 | 1.27003 | 102.3705 | 41.86257 | BGIOSGA015180 | -3.26133 | 274.157 | 2637.383 | Putative uncharacterized protein [Source:UniProtKB/TrEMBL;Acc:B8ATF3] | 2.5 | 53-73 | target 5' GCGGCACCGCGCGGUUCCGCG 3' | ::: :: ::::.:::::::::. | query 3' CGCGGUGGCGUGCCAAGGCGU 5' |
| SI24_SI08 | osa-miR159a.2 | 2.01046 | 142.1812 | 34.53662 | BGIOSGA007552 | -1.38766 | 0 | 1.616539 | Putative uncharacterized protein [Source:UniProtKB/TrEMBL;Acc:B8AHT0] | 3.5 | 2169-2189 | target 5' UGCUCUUCUUGGGGCAUGCAA 3' | ::: .::.::::::::::: | query 3' ACGUCGAGGACCCCGUACGUU 5' |
| SI24_SI08 | osa-miR159a.2 | 2.01046 | 142.1812 | 34.53662 | BGIOSGA033431 | -3.30611 | 0 | 8.890962 | Putative uncharacterized protein [Source:UniProtKB/TrEMBL;Acc:B8BI71] | 4 | 1782-1802 | target 5' UGCAGUUCUUAGGGCAUGCAG 3' | :::::. ::.: ::::::::. | query 3' ACGUCGAGGACCCCGUACGUU 5' |
| SI24_SI08 | osa-miR159a.2 | 2.01046 | 142.1812 | 34.53662 | BGIOSGA010571 | -3.17087 | 13.11186 | 126.09 | Putative uncharacterized protein [Source:UniProtKB/TrEMBL;Acc:B8AQZ1] | 4 | 438-458 | target 5' UGCAGCUCCUGGUGCAUCCAA 3' | :::::: :::::: :::: ::: | query 3' ACGUCGAGGACCCCGUACGUU 5' |
| SI24_SI08 | osa-miR159a.2 | 2.01046 | 142.1812 | 34.53662 | BGIOSGA017480 | -1.38766 | 0 | 1.616539 | Putative uncharacterized protein [Source:UniProtKB/TrEMBL;Acc:B8AX70] | 4 | 191-211 | target 5' UGCCGCUCCUGGGGCUUGCAC 3' | ::: :: ::::::::: :::: | query 3' ACGUCGAGGACCCCGUACGUU 5' |
| SI24_SI08 | osa-miR162a | 1.36883 | 1593.567 | 616.4264 | BGIOSGA011556 | -1.99133 | 5.959934 | 26.67289 | Putative uncharacterized protein [Source:UniProtKB/TrEM | 2 | 2977-2998 | target 5' CUGGAUGCAGAGGUUUUAUCGA 3' | :::::: ::::::: ::::::: | query 3' GACCUACGUCUCC-AAAUAGCU 5' |

| | | | | | | | | | | | | | | |
|---|---|---|---|---|---|---|---|---|---|---|---|---|---|---|
| | | | | | | | | | BL;Acc:A2XBW0] | | | | | |
| SI24_SI08 | osa-miR162b | 1.36142 | 1571.956 | 611.1936 | BGIOSGA011556 | -1.99133 | 5.959934 | 26.67289 | Putative uncharacterized protein [Source:UniProtKB/TrEMBL;Acc:A2XBW0] | 3 | 2977-2998 | target 5'<br>CUGGAUGCAGAGG<br>UUUUAUCGA 3' | ::::::<br>::::::: .::::::: | query 3'<br>GACCUACGUCUCC-GA<br>AUAGCU 5' |
| SI24_SI08 | osa-miR162b | 1.36142 | 1571.956 | 611.1936 | BGIOSGA022457 | -1.68755 | 8.343908 | 29.09769 | Putative uncharacterized protein [Source:UniProtKB/TrEMBL;Acc:A2YA75] | 4 | 106-125 | target 5'<br>GUGGA-GAAGAGGC<br>UUAUUGA 3' | ::::<br>: :::::::::.:: | query 3'<br>GACCUACGUCUCCGAA<br>UAGCU 5' |
| SI24_SI08 | osa-miR166a | 1.4635 | 184552.4 | 66920.46 | BGIOSGA009117 | -2.79085 | 15.49583 | 113.1577 | Putative uncharacterized protein [Source:UniProtKB/TrEMBL;Acc:B8AJ56] | 4 | 381-400 | target 5'<br>GGGGGAGGAAG-CU<br>GGUCCGG 3' | ::::.:<br>:::: ::::::::. | query 3'<br>CCCCUUACUUCGGACC<br>AGGCU 5' |
| SI24_SI08 | osa-miR166b | 1.4635 | 184552.4 | 66920.46 | BGIOSGA009117 | -2.79085 | 15.49583 | 113.1577 | Putative uncharacterized protein [Source:UniProtKB/TrEMBL;Acc:B8AJ56] | 4 | 381-400 | target 5'<br>GGGGGAGGAAG-CU<br>GGUCCGG 3' | ::::.:<br>:::: ::::::::. | query 3'<br>CCCCUUACUUCGGACC<br>AGGCU 5' |
| SI24_SI08 | osa-miR166c | 1.4635 | 184552.4 | 66920.46 | BGIOSGA009117 | -2.79085 | 15.49583 | 113.1577 | Putative uncharacterized protein [Source:UniProtKB/TrEMBL;Acc:B8AJ56] | 4 | 381-400 | target 5'<br>GGGGGAGGAAG-CU<br>GGUCCGG 3' | ::::.:<br>:::: ::::::::. | query 3'<br>CCCCUUACUUCGGACC<br>AGGCU 5' |
| SI24_SI08 | osa-miR166d | 1.4635 | 184552.4 | 66920.46 | BGIOSGA009117 | -2.79085 | 15.49583 | 113.1577 | Putative uncharacterized protein [Source:UniProtKB/TrEMBL;Acc:B8AJ56] | 4 | 381-400 | target 5'<br>GGGGGAGGAAG-CU<br>GGUCCGG 3' | ::::.:<br>:::: ::::::::. | query 3'<br>CCCCUUACUUCGGACC<br>AGGCU 5' |
| SI24_SI08 | osa-miR166f | 1.4635 | 184552.4 | 66920.46 | BGIOSGA009117 | -2.79085 | 15.49583 | 113.1577 | Putative uncharacterized protein [Source:UniProtKB/TrEMBL;Acc:B8AJ56] | 4 | 381-400 | target 5'<br>GGGGGAGGAAG-CU<br>GGUCCGG 3' | ::::.:<br>:::: ::::::::. | query 3'<br>CCCCUUACUUCGGACC<br>AGGCU 5' |
| SI24_SI08 | osa-miR166i | 1.69595 | 44689.26 | 13792.67 | BGIOSGA017414 | -3.18312 | 0 | 8.082693 | Putative uncharacterized protein [Source:UniProtKB/TrEMBL;Acc:A2XZA6] | 4 | 3637-3658 | target 5'<br>AAGGAAGUGAAGC<br>UUGAUCUGA 3' | ::::<br>: ::::::.:::::.:: | query 3'<br>CUCCUU-ACUUCGGAC<br>UAGGCU 5' |
| SI24_SI08 | osa-miR166i | 1.69595 | 44689.26 | 13792.67 | BGIOSGA014593 | -2.71534 | 7.151921 | 52.5375 | Putative uncharacterized protein [Source:UniProtKB/TrEMBL;Acc:B8ASY0] | 4 | 22-Feb | target 5'<br>UGGGGAGGAAGCC<br>UGAUCCGU 3' | .::.<br>: ::::::::::::: | query 3'<br>CUCCUUACUUCGGACU<br>AGGCU 5' |
| SI24_SI08 | osa-miR166j | 1.69595 | 44689.26 | 13792.67 | BGIOSGA017414 | -3.18312 | 0 | 8.082693 | Putative uncharacterized protein [Source:UniProtKB/TrEMBL;Acc:A2XZA6] | 4 | 3637-3658 | target 5'<br>AAGGAAGUGAAGC<br>UUGAUCUGA 3' | ::::<br>: ::::::.:::::.:: | query 3'<br>CUCCUU-ACUUCGGAC<br>UAGGCU 5' |
| SI24_SI08 | osa-miR166j | 1.69595 | 44689.26 | 13792.67 | BGIOSGA014593 | -2.71534 | 7.151921 | 52.5375 | Putative uncharacterized protein [Source:UniProtKB/TrEMBL;Acc:B8ASY0] | 4 | 22-Feb | target 5'<br>UGGGGAGGAAGCC<br>UGAUCCGU 3' | .::.<br>: ::::::::::::: | query 3'<br>CUCCUUACUUCGGACU<br>AGGCU 5' |
| SI24_SI08 | osa-miR166n | 1.4635 | 184552.4 | 66920.46 | BGIOSGA009117 | -2.79085 | 15.49583 | 113.1577 | Putative uncharacterized protein [Source:UniProtKB/TrEMBL;Acc:B8AJ56] | 4 | 381-400 | target 5'<br>GGGGGAGGAAG-CU<br>GGUCCGG 3' | ::::.:<br>:::: ::::::::. | query 3'<br>CCCCUUACUUCGGACC<br>AGGCU 5' |
| SI24_SI08 | osa-miR1850.1 | -1.14442 | 19.33665 | 43.9557 | BGIOSGA012292 | 1.05547 | 666.3206 | 320.0746 | Putative uncharacterized protein [Source:UniProtKB/TrEMBL;Acc:A2XEV9] | 4 | 472-493 | target 5'<br>CACCAUUUCUCCCA<br>ACUUUUCA 3' | : :::<br>:::::::::::::.:: | query 3'<br>GGGGU-UAGAGGGUUG<br>AAAGGU 5' |
| SI24_SI08 | osa-miR1860-5p | 1.28895 | 29.57369 | 11.51221 | BGIOSGA036396 | -1.38766 | 0 | 1.616539 | Putative uncharacterized protein [Source:UniProtKB/TrEM | 2.5 | 697-717 | target 5'<br>AGGUCUGGAAGCU<br>UGUUUUCU 3' | ::.:::<br>::::::: ::::::: | query 3'<br>UCUAGACCUUCGACCA<br>AAAGA 5' |

| | | | | | | | | | | | | | | |
|---|---|---|---|---|---|---|---|---|---|---|---|---|---|---|
| | | | | | | | | | BL;Acc:B8BP23] | | | | | |
| SI24_SI08 | osa-miR1860-5p | 1.28895 | 29.57369 | 11.51221 | BGIOSGA031384 | -2.67111 | 7.151921 | 50.92096 | Putative uncharacterized protein [Source:UniProtKB/TrEMBL;Acc:B8BIA4] | 3.5 | 163-182 | target 5' AGG-GUGAAAGCUGGUUUUCU 3' | ::. :: ::::::::::::: | query 3' UCUAGACCUUCGACCAAAAGA 5' |
| SI24_SI08 | osa-miR1862f | 1.22186 | 516.4022 | 220.8251 | BGIOSGA005796 | -5.50886 | 7.151921 | 370.1873 | Putative uncharacterized protein [Source:UniProtKB/TrEMBL;Acc:B8AHB3] | 3 | 1138-1156 | target 5' UCAAAG-AAACCAACUUCAU 3' | .::::. :::::::::.:::: | query 3' GGUUUUAUUUGGUUGGAGUA 5' |
| SI24_SI08 | osa-miR1862f | 1.22186 | 516.4022 | 220.8251 | BGIOSGA017434 | -1.86286 | 28.60768 | 106.6915 | Putative uncharacterized protein [Source:UniProtKB/TrEMBL;Acc:B8AXI0] | 3.5 | 385-403 | target 5' UCAAAA-AAACCAAGCUCAU 3' | .::::: ::::::: ::::: | query 3' GGUUUUAUUUGGUUGGAGUA 5' |
| SI24_SI08 | osa-miR1862f | 1.22186 | 516.4022 | 220.8251 | BGIOSGA014360 | -3.38917 | 29.79967 | 321.6912 | Putative uncharacterized protein [Source:UniProtKB/TrEMBL;Acc:B8AU52] | 4 | 302-321 | target 5' CUGCAAGAAACCAAUCUCAU 3' | :.. :: ::::::::.::::: | query 3' GGUUUUAUUUGGUUGGAGUA 5' |
| SI24_SI08 | osa-miR1862g | 1.22186 | 516.4022 | 220.8251 | BGIOSGA005796 | -5.50886 | 7.151921 | 370.1873 | Putative uncharacterized protein [Source:UniProtKB/TrEMBL;Acc:B8AHB3] | 3 | 1138-1156 | target 5' UCAAAG-AAACCAACUUCAU 3' | .::::. :::::::::.:::: | query 3' GGUUUUAUUUGGUUGGAGUA 5' |
| SI24_SI08 | osa-miR1862g | 1.22186 | 516.4022 | 220.8251 | BGIOSGA017434 | -1.86286 | 28.60768 | 106.6915 | Putative uncharacterized protein [Source:UniProtKB/TrEMBL;Acc:B8AXI0] | 3.5 | 385-403 | target 5' UCAAAA-AAACCAAGCUCAU 3' | .::::: ::::::: ::::: | query 3' GGUUUUAUUUGGUUGGAGUA 5' |
| SI24_SI08 | osa-miR1862g | 1.22186 | 516.4022 | 220.8251 | BGIOSGA014360 | -3.38917 | 29.79967 | 321.6912 | Putative uncharacterized protein [Source:UniProtKB/TrEMBL;Acc:B8AU52] | 4 | 302-321 | target 5' CUGCAAGAAACCAAUCUCAU 3' | :.. :: ::::::::.::::: | query 3' GGUUUUAUUUGGUUGGAGUA 5' |
| SI24_SI08 | osa-miR2055 | 1.11973 | 126.2569 | 57.56104 | BGIOSGA030036 | -1.56093 | 39.33557 | 118.0073 | Putative uncharacterized protein [Source:UniProtKB/TrEMBL;Acc:B8BEA3] | 4 | 1464-1483 | target 5' UGAACC-CCUUCUCAAGGAAG 3' | .::: : :::::.:::::::. | query 3' CUUUGGUGGAAGGGUUCCUUU 5' |
| SI24_SI08 | osa-miR2055 | 1.11973 | 126.2569 | 57.56104 | BGIOSGA014296 | -1.77602 | 0 | 2.424808 | Putative uncharacterized protein [Source:UniProtKB/TrEMBL;Acc:A2XXW3] | 4 | 2255-2275 | target 5' CAAACAAACUUCCUAAGGAAA 3' | :::: : :::::.::::::: | query 3' CUUUGGUGGAAGGGUUCCUUU 5' |
| SI24_SI08 | osa-miR2873b | 1.11989 | 205.8784 | 94.19079 | BGIOSGA013396 | -2.97249 | 32.18364 | 259.4544 | Putative uncharacterized protein [Source:UniProtKB/TrEMBL;Acc:B8AQ13] | 3.5 | 1620-1640 | target 5' CAUGCCAUAUCUCAAGUCUAU 3' | :::.:: : :::::::::::.: | query 3' GUAUGGUUUAGAGUUCAGGUU 5' |
| SI24_SI08 | osa-miR394 | 2.16712 | 356.0218 | 78.49232 | BGIOSGA020470 | -6.6102 | 5.959934 | 678.9462 | Putative uncharacterized protein [Source:UniProtKB/TrEMBL;Acc:B8AX92] | 3 | 1366-1385 | target 5' GGAGUUGGACAGAAUGCAAA 3' | :::: : ::::::::::: :: | query 3' CCUCCACCUGUCUUACGGUU 5' |
| SI24_SI08 | osa-miR394 | 2.16712 | 356.0218 | 78.49232 | BGIOSGA025510 | -1.95992 | 7.151921 | 30.71423 | Putative uncharacterized protein [Source:UniProtKB/TrEMBL;Acc:B8B516] | 4 | 20-38 | target 5' GGCGGCGGA-AGAAUGCCAA 3' | :: :: ::: :::::::::: | query 3' CCUCCACCUGUCUUACGGUU 5' |
| SI24_SI08 | osa-miR396a | 1.05407 | 946.3582 | 455.2555 | BGIOSGA001547 | -1.5614 | 181.182 | 536.6908 | Putative uncharacterized protein [Source:UniProtKB/TrEMBL;Acc:B8A8X2] | 3 | 127-147 | target 5' CGGUUUAAGAGAGCUGUGGGA 3' | :.:::. ::::.:::::::::.: | query 3' GUCAAGUUCUUUCGACACCUU 5' |
| SI24_SI08 | osa-miR396a | 1.05407 | 946.3582 | 455.2555 | BGIOSGA021248 | -3.13497 | 16.68782 | 154.3794 | Putative uncharacterized protein [Source:UniProtKB/TrEM | 3.5 | 697-717 | target 5' CAGUUCUGGAAAGCUGGGGAA 3' | :::::: .:::::::: :::: | query 3' GUCAAGUUCUUUCGACACCUU 5' |

| | | | | | | | | | | | | | | |
|---|---|---|---|---|---|---|---|---|---|---|---|---|---|---|
| | | | | | | | | | BL;Acc:B8B225] | | | | | |
| SI24_SI08 | osa-miR396a | 1.05407 | 946.3582 | 455.2555 | BGIOSGA034107 | -2.53138 | 50.06345 | 294.21 | Putative uncharacterized protein [Source:UniProtKB/TrEMBL;Acc:A2ZDM9] | 4 | 1603-1623 | target 5' CUGUCCAAGAAGG CUGUGGAU 3' | : :: : :::::.::::::: | query 3' GUCAAGUUCUUUCGAC ACCUU 5' |
| SI24_SI08 | osa-miR396a | 1.05407 | 946.3582 | 455.2555 | BGIOSGA002892 | -2.24264 | 151.3823 | 720.1679 | Putative uncharacterized protein [Source:UniProtKB/TrEMBL;Acc:A2WLE4] | 4 | 1681-1701 | target 5' GAGUUGCAGAAGG CUGUGGAA 3' | :::: ::::.::::::::: | query 3' GUCAAGUUCUUUCGAC ACCUU 5' |
| SI24_SI08 | osa-miR396a | 1.05407 | 946.3582 | 455.2555 | BGIOSGA006799 | -1.78053 | 107.2788 | 370.9956 | Putative uncharacterized protein [Source:UniProtKB/TrEMBL;Acc:A2X2X1] | 4 | 296-315 | target 5' CUGUUC-AGAGAGC UGUGGGA 3' | : :::: :::.:::::::::.: | query 3' GUCAAGUUCUUUCGAC ACCUU 5' |
| SI24_SI08 | osa-miR396b | 1.05407 | 946.3582 | 455.2555 | BGIOSGA001547 | -1.5614 | 181.182 | 536.6908 | Putative uncharacterized protein [Source:UniProtKB/TrEMBL;Acc:B8A8X2] | 3 | 127-147 | target 5' CGGUUUAAGAGAG CUGUGGGA 3' | :.:::. ::::.::::::::.: | query 3' GUCAAGUUCUUUCGAC ACCUU 5' |
| SI24_SI08 | osa-miR396b | 1.05407 | 946.3582 | 455.2555 | BGIOSGA021248 | -3.13497 | 16.68782 | 154.3794 | Putative uncharacterized protein [Source:UniProtKB/TrEMBL;Acc:B8B225] | 3.5 | 697-717 | target 5' CAGUUCUGGAAAG CUGGGGAA 3' | :::::: .:::::::: :::: | query 3' GUCAAGUUCUUUCGAC ACCUU 5' |
| SI24_SI08 | osa-miR396b | 1.05407 | 946.3582 | 455.2555 | BGIOSGA034107 | -2.53138 | 50.06345 | 294.21 | Putative uncharacterized protein [Source:UniProtKB/TrEMBL;Acc:A2ZDM9] | 4 | 1603-1623 | target 5' CUGUCCAAGAAGG CUGUGGAU 3' | : :: : :::::.::::::: | query 3' GUCAAGUUCUUUCGAC ACCUU 5' |
| SI24_SI08 | osa-miR396b | 1.05407 | 946.3582 | 455.2555 | BGIOSGA002892 | -2.24264 | 151.3823 | 720.1679 | Putative uncharacterized protein [Source:UniProtKB/TrEMBL;Acc:A2WLE4] | 4 | 1681-1701 | target 5' GAGUUGCAGAAGG CUGUGGAA 3' | :::: ::::.::::::::: | query 3' GUCAAGUUCUUUCGAC ACCUU 5' |
| SI24_SI08 | osa-miR396b | 1.05407 | 946.3582 | 455.2555 | BGIOSGA006799 | -1.78053 | 107.2788 | 370.9956 | Putative uncharacterized protein [Source:UniProtKB/TrEMBL;Acc:A2X2X1] | 4 | 296-315 | target 5' CUGUUC-AGAGAGC UGUGGGA 3' | : :::: :::.:::::::::.: | query 3' GUCAAGUUCUUUCGAC ACCUU 5' |
| SI24_SI08 | osa-miR396c | 1.06981 | 961.145 | 457.3486 | BGIOSGA002892 | -2.24264 | 151.3823 | 720.1679 | Putative uncharacterized protein [Source:UniProtKB/TrEMBL;Acc:A2WLE4] | 3.5 | 1681-1701 | target 5' GAGUUGCAGAAGG CUGUGGAA 3' | .:::: ::::.::::::::: | query 3' UUCAAGUUCUUUCGAC ACCUU 5' |
| SI24_SI08 | osa-miR396c | 1.06981 | 961.145 | 457.3486 | BGIOSGA001547 | -1.5614 | 181.182 | 536.6908 | Putative uncharacterized protein [Source:UniProtKB/TrEMBL;Acc:B8A8X2] | 4 | 127-147 | target 5' CGGUUUAAGAGAG CUGUGGGA 3' | .::: .::::.::::::::.: | query 3' UUCAAGUUCUUUCGAC ACCUU 5' |
| SI24_SI08 | osa-miR396c | 1.06981 | 961.145 | 457.3486 | BGIOSGA034898 | -3.12456 | 4.767947 | 49.30442 | Putative uncharacterized protein [Source:UniProtKB/TrEMBL;Acc:A2ZBY7] | 4 | 1281-1300 | target 5' AAGUUCAAGAAAG C-AUGGAA 3' | :::::: ::::::: ::::: | query 3' UUCAAGUUCUUUCGAC ACCUU 5' |
| SI24_SI08 | osa-miR396c | 1.06981 | 961.145 | 457.3486 | BGIOSGA028990 | -1.67957 | 33.37563 | 109.1163 | Putative uncharacterized protein [Source:UniProtKB/TrEMBL;Acc:A2YX00] | 4 | 466-486 | target 5' AUGAUCAAGAAGG CUGUGGAU 3' | : : :: :::::.::::::: | query 3' UUCAAGUUCUUUCGAC ACCUU 5' |
| SI24_SI08 | osa-miR396c | 1.06981 | 961.145 | 457.3486 | BGIOSGA004420 | -1.99133 | 5.959934 | 26.67289 | Putative uncharacterized protein [Source:UniProtKB/TrEMBL;Acc:A2WUV3] | 4 | 1247-1266 | target 5' AAGUAAAAG-AAGC UGUGGAA 3' | :::: ::: :::::::::: | query 3' UUCAAGUUCUUUCGAC ACCUU 5' |
| SI24_SI08 | osa-miR396d | 1.40707 | 27811.78 | 10486.57 | BGIOSGA034580 | -1.77602 | 0 | 2.424808 | Putative uncharacterized protein [Source:UniProtKB/TrEM | 4 | 966-985 | target 5' CA-UUGAAGAAAGC CUCUGGA 3' | :: :: ::::::::: :::: | query 3' GUCAAGUUCUUUCGGA CACCU 5' |

| | | | | | | | | | | | | | | |
|---|---|---|---|---|---|---|---|---|---|---|---|---|---|---|
| | | | | | | | | | BL;Acc:A2ZB08] | | | | | |
| SI24_SI08 | osa-miR396e | 1.40707 | 27811.78 | 10486.57 | BGIOSGA034580 | -1.77602 | 0 | 2.424808 | Putative uncharacterized protein [Source:UniProtKB/TrEMBL;Acc:A2ZB08] | 4 | 966-985 | target 5' CA-UUGAAGAAAGCCUCUGGA 3' | :: :: ::::::::: :::: | query 3' GUCAAGUUCUUUCGGACACCU 5' |
| SI24_SI08 | osa-miR444b.2 | 1.11283 | 8285.184 | 3830.425 | BGIOSGA008494 | -3.95874 | 0 | 14.54885 | Putative uncharacterized protein [Source:UniProtKB/TrEMBL;Acc:B8AED3] | 0 | 282-302 | target 5' AAGCUUGAGACAACAACUGCA 3' | :::::: ::::::::::::::: | query 3' UUCGAACUCUGUUGUUGACGU 5' |
| SI24_SI08 | osa-miR444b.2 | 1.11283 | 8285.184 | 3830.425 | BGIOSGA013971 | -2.86448 | 214.5576 | 1568.851 | Putative uncharacterized protein [Source:UniProtKB/TrEMBL;Acc:A2XP79] | 3.5 | 295-314 | target 5' AGGC-UGAGACAACAACAGCA 3' | :.:: : :::::::::: ::: | query 3' UUCGAACUCUGUUGUUGACGU 5' |
| SI24_SI08 | osa-miR444b.2 | 1.11283 | 8285.184 | 3830.425 | BGIOSGA030482 | -2.75365 | 46.48749 | 319.2664 | Putative uncharacterized protein [Source:UniProtKB/TrEMBL;Acc:B8BE73] | 4 | 1687-1707 | target 5' AGCCUUGGGACAACAACUGUC 3' | :. ::: :.::::::::::. | query 3' UUCGAACUCUGUUGUUGACGU 5' |
| SI24_SI08 | osa-miR444b.2 | 1.11283 | 8285.184 | 3830.425 | BGIOSGA008396 | -2.46403 | 3.575961 | 24.24808 | Putative uncharacterized protein [Source:UniProtKB/TrEMBL;Acc:B8AJC5] | 4 | 1818-1838 | target 5' AAGCUUUAAGCAACAACUGCA 3' | :::::: : .:::::::::: | query 3' UUCGAACUCUGUUGUUGACGU 5' |
| SI24_SI08 | osa-miR444c.2 | 1.11283 | 8285.184 | 3830.425 | BGIOSGA008494 | -3.95874 | 0 | 14.54885 | Putative uncharacterized protein [Source:UniProtKB/TrEMBL;Acc:B8AED3] | 0 | 282-302 | target 5' AAGCUUGAGACAACAACUGCA 3' | :::::: ::::::::::::::: | query 3' UUCGAACUCUGUUGUUGACGU 5' |
| SI24_SI08 | osa-miR444c.2 | 1.11283 | 8285.184 | 3830.425 | BGIOSGA013971 | -2.86448 | 214.5576 | 1568.851 | Putative uncharacterized protein [Source:UniProtKB/TrEMBL;Acc:A2XP79] | 3.5 | 295-314 | target 5' AGGC-UGAGACAACAACAGCA 3' | :.:: : :::::::::: ::: | query 3' UUCGAACUCUGUUGUUGACGU 5' |
| SI24_SI08 | osa-miR444c.2 | 1.11283 | 8285.184 | 3830.425 | BGIOSGA030482 | -2.75365 | 46.48749 | 319.2664 | Putative uncharacterized protein [Source:UniProtKB/TrEMBL;Acc:B8BE73] | 4 | 1687-1707 | target 5' AGCCUUGGGACAACAACUGUC 3' | :. ::: :.::::::::::. | query 3' UUCGAACUCUGUUGUUGACGU 5' |
| SI24_SI08 | osa-miR444c.2 | 1.11283 | 8285.184 | 3830.425 | BGIOSGA008396 | -2.46403 | 3.575961 | 24.24808 | Putative uncharacterized protein [Source:UniProtKB/TrEMBL;Acc:B8AJC5] | 4 | 1818-1838 | target 5' AAGCUUUAAGCAACAACUGCA 3' | :::::: : .:::::::::: | query 3' UUCGAACUCUGUUGUUGACGU 5' |
| SI24_SI08 | osa-miR444f | 1.07038 | 10662.45 | 5076.884 | BGIOSGA008494 | -3.95874 | 0 | 14.54885 | Putative uncharacterized protein [Source:UniProtKB/TrEMBL;Acc:B8AED3] | 2 | 282-302 | target 5' AAGCUUGAGACAACAACUGCA 3' | :::::: ::: :::::::::: | query 3' UUCGAACUCCGUUGUUGACGU 5' |
| SI24_SI08 | osa-miR444f | 1.07038 | 10662.45 | 5076.884 | BGIOSGA008396 | -2.46403 | 3.575961 | 24.24808 | Putative uncharacterized protein [Source:UniProtKB/TrEMBL;Acc:B8AJC5] | 3 | 1818-1838 | target 5' AAGCUUUAAGCAACAACUGCA 3' | :::::: : ::::::::::: | query 3' UUCGAACUCCGUUGUUGACGU 5' |
| SI24_SI08 | osa-miR5144-5p | 1.34898 | 1526.458 | 598.6348 | BGIOSGA024088 | -1.20157 | 1.191987 | 4.041346 | F-box protein; MM724 [Source:UniProtKB/TrEMBL;Acc:A2YMM3] | 4 | 52-73 | target 5' UUCUCUUGCAGCAGCACGAGGA 3' | :::: :: :::::::.::.: | query 3' CAGAGAA-GUCGUCGUGUUCUU 5' |
| SI24_SI08 | osa-miR5144-5p | 1.34898 | 1526.458 | 598.6348 | BGIOSGA035800 | -2.04164 | 2.383974 | 12.93231 | Putative uncharacterized protein [Source:UniProtKB/TrEMBL;Acc:A2ZMY0] | 4 | 1231-1251 | target 5' GUCUGUUCAGCAGCGCAUGAA 3' | :::: : :::::::.:: ::: | query 3' CAGAGAAGUCGUCGUGUUCUU 5' |
| SI24_SI08 | osa-miR528 | -1.16497 | 4517.95 | 10131.79 | BGIOSGA024054 | 1.5734 | 47.67947 | 15.35712 | Putative uncharacterized protein [Source:UniProtKB/TrEMBL;Acc:B8B7J3] | 2 | 378-397 | target 5' CUCCUCUGC-UGCCCCUUCCA 3' | :::::: ::: :::::::::: | query 3' GAGGAGACGUACGGGGAAGGU 5' |

| | | | | | | | | | | | | | | |
|---|---|---|---|---|---|---|---|---|---|---|---|---|---|---|
| SI24_SI08 | osa-miR528 | -1.16497 | 4517.95 | 10131.79 | BGIOSGA009946 | 2.72439 | 64.36729 | 8.890962 | Putative uncharacterized protein [Source:UniProtKB/TrEMBL;Acc:A2XL83] | 4 | 61-80 | target 5' CUCCUCUGCGUGCUCC-UCCA 3' | :::::: :::.:::.:: :::: | query 3' GAGGAGACGUACGGGGAAGGU 5' |
| SI24_SI08 | osa-miR528 | -1.16497 | 4517.95 | 10131.79 | BGIOSGA029010 | 1.40526 | 100.1269 | 37.18039 | Putative uncharacterized protein [Source:UniProtKB/TrEMBL;Acc:A2YX38] | 4 | 188-208 | target 5' CGGCUCAUCAUGCCCCUUCCA 3' | : : :: ::::::::::::: | query 3' GAGGAGACGUACGGGGAAGGU 5' |
| SI24_SI08 | osa-miR530-3p | -1.31529 | 117.1573 | 293.038 | BGIOSGA001350 | 2.30386 | 11.91987 | 1.616539 | Putative uncharacterized protein [Source:UniProtKB/TrEMBL;Acc:A2WRS1] | 4 | 32-52 | target 5' CUUGCACCUGCCUCUGCUCCU 3' | :::: : :::::::::: ::: | query 3' CAACGUAGACGGAGACGUGGA 5' |
| SI48_SC00 | osa-miR156a | 1.24374 | 266643.4 | 112596.6 | BGIOSGA029541 | -1.8357 | 6.067769 | 24.22802 | Putative uncharacterized protein [Source:UniProtKB/TrEMBL;Acc:B8BD46] | 1 | 578-597 | target 5' GUGCUCUCUCUCUUCUGUCA 3' | :::::: ::::::::::::: | query 3' CACGAGUGAGAGAAGACAGU 5' |
| SI48_SC00 | osa-miR156a | 1.24374 | 266643.4 | 112596.6 | BGIOSGA026705 | -1.23065 | 6.934593 | 17.62038 | Putative uncharacterized protein [Source:UniProtKB/TrEMBL;Acc:A2YWW1] | 1 | 818-837 | target 5' GUGCUCUCUCUCUUCUGUCA 3' | :::::: ::::::::::::: | query 3' CACGAGUGAGAGAAGACAGU 5' |
| SI48_SC00 | osa-miR156a | 1.24374 | 266643.4 | 112596.6 | BGIOSGA007643 | -2.65427 | 15.60283 | 103.5197 | Putative uncharacterized protein [Source:UniProtKB/TrEMBL;Acc:A2X1G6] | 1 | 716-735 | target 5' GUGCUCUCUCUCUUCUGUCA 3' | :::::: ::::::::::::: | query 3' CACGAGUGAGAGAAGACAGU 5' |
| SI48_SC00 | osa-miR156b | 1.24374 | 266643.4 | 112596.6 | BGIOSGA029541 | -1.8357 | 6.067769 | 24.22802 | Putative uncharacterized protein [Source:UniProtKB/TrEMBL;Acc:B8BD46] | 1 | 578-597 | target 5' GUGCUCUCUCUCUUCUGUCA 3' | :::::: ::::::::::::: | query 3' CACGAGUGAGAGAAGACAGU 5' |
| SI48_SC00 | osa-miR156b | 1.24374 | 266643.4 | 112596.6 | BGIOSGA026705 | -1.23065 | 6.934593 | 17.62038 | Putative uncharacterized protein [Source:UniProtKB/TrEMBL;Acc:A2YWW1] | 1 | 818-837 | target 5' GUGCUCUCUCUCUUCUGUCA 3' | :::::: ::::::::::::: | query 3' CACGAGUGAGAGAAGACAGU 5' |
| SI48_SC00 | osa-miR156b | 1.24374 | 266643.4 | 112596.6 | BGIOSGA007643 | -2.65427 | 15.60283 | 103.5197 | Putative uncharacterized protein [Source:UniProtKB/TrEMBL;Acc:A2X1G6] | 1 | 716-735 | target 5' GUGCUCUCUCUCUUCUGUCA 3' | :::::: ::::::::::::: | query 3' CACGAGUGAGAGAAGACAGU 5' |
| SI48_SC00 | osa-miR156c | 1.24374 | 266643.4 | 112596.6 | BGIOSGA029541 | -1.8357 | 6.067769 | 24.22802 | Putative uncharacterized protein [Source:UniProtKB/TrEMBL;Acc:B8BD46] | 1 | 578-597 | target 5' GUGCUCUCUCUCUUCUGUCA 3' | :::::: ::::::::::::: | query 3' CACGAGUGAGAGAAGACAGU 5' |
| SI48_SC00 | osa-miR156c | 1.24374 | 266643.4 | 112596.6 | BGIOSGA026705 | -1.23065 | 6.934593 | 17.62038 | Putative uncharacterized protein [Source:UniProtKB/TrEMBL;Acc:A2YWW1] | 1 | 818-837 | target 5' GUGCUCUCUCUCUUCUGUCA 3' | :::::: ::::::::::::: | query 3' CACGAGUGAGAGAAGACAGU 5' |
| SI48_SC00 | osa-miR156c | 1.24374 | 266643.4 | 112596.6 | BGIOSGA007643 | -2.65427 | 15.60283 | 103.5197 | Putative uncharacterized protein [Source:UniProtKB/TrEMBL;Acc:A2X1G6] | 1 | 716-735 | target 5' GUGCUCUCUCUCUUCUGUCA 3' | :::::: ::::::::::::: | query 3' CACGAGUGAGAGAAGACAGU 5' |
| SI48_SC00 | osa-miR156d | 1.24374 | 266643.4 | 112596.6 | BGIOSGA029541 | -1.8357 | 6.067769 | 24.22802 | Putative uncharacterized protein [Source:UniProtKB/TrEMBL;Acc:B8BD46] | 1 | 578-597 | target 5' GUGCUCUCUCUCUUCUGUCA 3' | :::::: ::::::::::::: | query 3' CACGAGUGAGAGAAGACAGU 5' |
| SI48_SC00 | osa-miR156d | 1.24374 | 266643.4 | 112596.6 | BGIOSGA026705 | -1.23065 | 6.934593 | 17.62038 | Putative uncharacterized protein [Source:UniProtKB/TrEMBL;Acc:A2YWW1] | 1 | 818-837 | target 5' GUGCUCUCUCUCUUCUGUCA 3' | :::::: ::::::::::::: | query 3' CACGAGUGAGAGAAGACAGU 5' |

| | | | | | | | | | | | | | | |
|---|---|---|---|---|---|---|---|---|---|---|---|---|---|---|
| SI48_SC00 | osa-miR156d | 1.24374 | 266643.4 | 112596.6 | BGIOSGA007643 | -2.65427 | 15.60283 | 103.5197 | Putative uncharacterized protein [Source:UniProtKB/TrEMBL;Acc:A2X1G6] | 1 | 716-735 | target 5' GUGCUCUCUCUCUUCUGUCA 3' | :::::: ::::::::::::: | query 3' CACGAGUGAGAGAAGACAGU 5' |
| SI48_SC00 | osa-miR156e | 1.24374 | 266643.4 | 112596.6 | BGIOSGA029541 | -1.8357 | 6.067769 | 24.22802 | Putative uncharacterized protein [Source:UniProtKB/TrEMBL;Acc:B8BD46] | 1 | 578-597 | target 5' GUGCUCUCUCUCUUCUGUCA 3' | :::::: ::::::::::::: | query 3' CACGAGUGAGAGAAGACAGU 5' |
| SI48_SC00 | osa-miR156e | 1.24374 | 266643.4 | 112596.6 | BGIOSGA026705 | -1.23065 | 6.934593 | 17.62038 | Putative uncharacterized protein [Source:UniProtKB/TrEMBL;Acc:A2YWW1] | 1 | 818-837 | target 5' GUGCUCUCUCUCUUCUGUCA 3' | :::::: ::::::::::::: | query 3' CACGAGUGAGAGAAGACAGU 5' |
| SI48_SC00 | osa-miR156e | 1.24374 | 266643.4 | 112596.6 | BGIOSGA007643 | -2.65427 | 15.60283 | 103.5197 | Putative uncharacterized protein [Source:UniProtKB/TrEMBL;Acc:A2X1G6] | 1 | 716-735 | target 5' GUGCUCUCUCUCUUCUGUCA 3' | :::::: ::::::::::::: | query 3' CACGAGUGAGAGAAGACAGU 5' |
| SI48_SC00 | osa-miR156f | 1.24374 | 266643.4 | 112596.6 | BGIOSGA029541 | -1.8357 | 6.067769 | 24.22802 | Putative uncharacterized protein [Source:UniProtKB/TrEMBL;Acc:B8BD46] | 1 | 578-597 | target 5' GUGCUCUCUCUCUUCUGUCA 3' | :::::: ::::::::::::: | query 3' CACGAGUGAGAGAAGACAGU 5' |
| SI48_SC00 | osa-miR156f | 1.24374 | 266643.4 | 112596.6 | BGIOSGA026705 | -1.23065 | 6.934593 | 17.62038 | Putative uncharacterized protein [Source:UniProtKB/TrEMBL;Acc:A2YWW1] | 1 | 818-837 | target 5' GUGCUCUCUCUCUUCUGUCA 3' | :::::: ::::::::::::: | query 3' CACGAGUGAGAGAAGACAGU 5' |
| SI48_SC00 | osa-miR156f | 1.24374 | 266643.4 | 112596.6 | BGIOSGA007643 | -2.65427 | 15.60283 | 103.5197 | Putative uncharacterized protein [Source:UniProtKB/TrEMBL;Acc:A2X1G6] | 1 | 716-735 | target 5' GUGCUCUCUCUCUUCUGUCA 3' | :::::: ::::::::::::: | query 3' CACGAGUGAGAGAAGACAGU 5' |
| SI48_SC00 | osa-miR156g | 1.24374 | 266643.4 | 112596.6 | BGIOSGA029541 | -1.8357 | 6.067769 | 24.22802 | Putative uncharacterized protein [Source:UniProtKB/TrEMBL;Acc:B8BD46] | 1 | 578-597 | target 5' GUGCUCUCUCUCUUCUGUCA 3' | :::::: ::::::::::::: | query 3' CACGAGUGAGAGAAGACAGU 5' |
| SI48_SC00 | osa-miR156g | 1.24374 | 266643.4 | 112596.6 | BGIOSGA026705 | -1.23065 | 6.934593 | 17.62038 | Putative uncharacterized protein [Source:UniProtKB/TrEMBL;Acc:A2YWW1] | 1 | 818-837 | target 5' GUGCUCUCUCUCUUCUGUCA 3' | :::::: ::::::::::::: | query 3' CACGAGUGAGAGAAGACAGU 5' |
| SI48_SC00 | osa-miR156g | 1.24374 | 266643.4 | 112596.6 | BGIOSGA007643 | -2.65427 | 15.60283 | 103.5197 | Putative uncharacterized protein [Source:UniProtKB/TrEMBL;Acc:A2X1G6] | 1 | 716-735 | target 5' GUGCUCUCUCUCUUCUGUCA 3' | :::::: ::::::::::::: | query 3' CACGAGUGAGAGAAGACAGU 5' |
| SI48_SC00 | osa-miR156h | 1.24374 | 266643.4 | 112596.6 | BGIOSGA029541 | -1.8357 | 6.067769 | 24.22802 | Putative uncharacterized protein [Source:UniProtKB/TrEMBL;Acc:B8BD46] | 1 | 578-597 | target 5' GUGCUCUCUCUCUUCUGUCA 3' | :::::: ::::::::::::: | query 3' CACGAGUGAGAGAAGACAGU 5' |
| SI48_SC00 | osa-miR156h | 1.24374 | 266643.4 | 112596.6 | BGIOSGA026705 | -1.23065 | 6.934593 | 17.62038 | Putative uncharacterized protein [Source:UniProtKB/TrEMBL;Acc:A2YWW1] | 1 | 818-837 | target 5' GUGCUCUCUCUCUUCUGUCA 3' | :::::: ::::::::::::: | query 3' CACGAGUGAGAGAAGACAGU 5' |
| SI48_SC00 | osa-miR156h | 1.24374 | 266643.4 | 112596.6 | BGIOSGA007643 | -2.65427 | 15.60283 | 103.5197 | Putative uncharacterized protein [Source:UniProtKB/TrEMBL;Acc:A2X1G6] | 1 | 716-735 | target 5' GUGCUCUCUCUCUUCUGUCA 3' | :::::: ::::::::::::: | query 3' CACGAGUGAGAGAAGACAGU 5' |
| SI48_SC00 | osa-miR156i | 1.24374 | 266643.4 | 112596.6 | BGIOSGA029541 | -1.8357 | 6.067769 | 24.22802 | Putative uncharacterized protein [Source:UniProtKB/TrEMBL;Acc:B8BD46] | 1 | 578-597 | target 5' GUGCUCUCUCUCUUCUGUCA 3' | :::::: ::::::::::::: | query 3' CACGAGUGAGAGAAGACAGU 5' |

| | | | | | | | | | | | | | | |
|---|---|---|---|---|---|---|---|---|---|---|---|---|---|---|
| SI48_SC00 | osa-miR156i | 1.24374 | 266643.4 | 112596.6 | BGIOSGA026705 | -1.23065 | 6.934593 | 17.62038 | Putative uncharacterized protein [Source:UniProtKB/TrEMBL;Acc:A2YWW1] | 1 | 818-837 | target 5' GUGCUCUCUCUCUUCUGUCA 3' | :::::: :::::::::::: | query 3' CACGAGUGAGAGAAGACAGU 5' |
| SI48_SC00 | osa-miR156i | 1.24374 | 266643.4 | 112596.6 | BGIOSGA007643 | -2.65427 | 15.60283 | 103.5197 | Putative uncharacterized protein [Source:UniProtKB/TrEMBL;Acc:A2X1G6] | 1 | 716-735 | target 5' GUGCUCUCUCUCUUCUGUCA 3' | :::::: :::::::::::: | query 3' CACGAGUGAGAGAAGACAGU 5' |
| SI48_SC00 | osa-miR156j | 1.24374 | 266643.4 | 112596.6 | BGIOSGA029541 | -1.8357 | 6.067769 | 24.22802 | Putative uncharacterized protein [Source:UniProtKB/TrEMBL;Acc:B8BD46] | 1 | 578-597 | target 5' GUGCUCUCUCUCUUCUGUCA 3' | :::::: :::::::::::: | query 3' CACGAGUGAGAGAAGACAGU 5' |
| SI48_SC00 | osa-miR156j | 1.24374 | 266643.4 | 112596.6 | BGIOSGA026705 | -1.23065 | 6.934593 | 17.62038 | Putative uncharacterized protein [Source:UniProtKB/TrEMBL;Acc:A2YWW1] | 1 | 818-837 | target 5' GUGCUCUCUCUCUUCUGUCA 3' | :::::: :::::::::::: | query 3' CACGAGUGAGAGAAGACAGU 5' |
| SI48_SC00 | osa-miR156j | 1.24374 | 266643.4 | 112596.6 | BGIOSGA007643 | -2.65427 | 15.60283 | 103.5197 | Putative uncharacterized protein [Source:UniProtKB/TrEMBL;Acc:A2X1G6] | 1 | 716-735 | target 5' GUGCUCUCUCUCUUCUGUCA 3' | :::::: :::::::::::: | query 3' CACGAGUGAGAGAAGACAGU 5' |
| SI48_SC00 | osa-miR156k | 1.24638 | 263106.4 | 110900.3 | BGIOSGA026705 | -1.23065 | 6.934593 | 17.62038 | Putative uncharacterized protein [Source:UniProtKB/TrEMBL;Acc:A2YWW1] | 0 | 817-837 | target 5' UGUGCUCUCUCUCUUCUGUCA 3' | :::::: :::::::::::::: | query 3' ACACGAGAGAGAGAAGACAGU 5' |
| SI48_SC00 | osa-miR156k | 1.24638 | 263106.4 | 110900.3 | BGIOSGA029541 | -1.8357 | 6.067769 | 24.22802 | Putative uncharacterized protein [Source:UniProtKB/TrEMBL;Acc:B8BD46] | 0 | 577-597 | target 5' UGUGCUCUCUCUCUUCUGUCA 3' | :::::: :::::::::::::: | query 3' ACACGAGAGAGAGAAGACAGU 5' |
| SI48_SC00 | osa-miR156k | 1.24638 | 263106.4 | 110900.3 | BGIOSGA007643 | -2.65427 | 15.60283 | 103.5197 | Putative uncharacterized protein [Source:UniProtKB/TrEMBL;Acc:A2X1G6] | 1 | 715-735 | target 5' CGUGCUCUCUCUCUUCUGUCA 3' | :::: ::::::::::::::: | query 3' ACACGAGAGAGAGAAGACAGU 5' |
| SI48_SC00 | osa-miR156l | 1.09127 | 1789.157 | 839.206 | BGIOSGA026705 | -1.23065 | 6.934593 | 17.62038 | Putative uncharacterized protein [Source:UniProtKB/TrEMBL;Acc:A2YWW1] | 2.5 | 817-837 | target 5' UGUGCUCUCUCUCUUCUGUCA 3' | :.:::: : :::::::::::: | query 3' AUACGAGUGAGAGAAGACAGC 5' |
| SI48_SC00 | osa-miR156l | 1.09127 | 1789.157 | 839.206 | BGIOSGA029541 | -1.8357 | 6.067769 | 24.22802 | Putative uncharacterized protein [Source:UniProtKB/TrEMBL;Acc:B8BD46] | 2.5 | 577-597 | target 5' UGUGCUCUCUCUCUUCUGUCA 3' | :.:::: : :::::::::::: | query 3' AUACGAGUGAGAGAAGACAGC 5' |
| SI48_SC00 | osa-miR156l | 1.09127 | 1789.157 | 839.206 | BGIOSGA007643 | -2.65427 | 15.60283 | 103.5197 | Putative uncharacterized protein [Source:UniProtKB/TrEMBL;Acc:A2X1G6] | 3.5 | 715-735 | target 5' CGUGCUCUCUCUCUUCUGUCA 3' | .::: :: :::::::::::: | query 3' AUACGAGUGAGAGAAGACAGC 5' |
| SI48_SC00 | osa-miR166a | -1.88318 | 54169.96 | 199828.5 | BGIOSGA005688 | 1.00572 | 80.61464 | 39.64585 | Putative uncharacterized protein [Source:UniProtKB/TrEMBL;Acc:B8AI06] | 1 | 2356-2375 | target 5' GGGGAA-GAAGCCUGGUCCGA 3' | :::::: ::::::::::::: | query 3' CCCCUUACUUCGGACCAGGCU 5' |
| SI48_SC00 | osa-miR166a | -1.88318 | 54169.96 | 199828.5 | BGIOSGA008799 | 5.32218 | 39.00708 | 0 | Putative uncharacterized protein [Source:UniProtKB/TrEMBL;Acc:A2X894] | 3 | 177-197 | target 5' GGUGAAGGAAGCCUGGUCCGU 3' | :: ::: :::::::::::: | query 3' CCCCUUACUUCGGACCAGGCU 5' |
| SI48_SC00 | osa-miR166a | -1.88318 | 54169.96 | 199828.5 | BGIOSGA002254 | 2.63249 | 5.200944 | 0 | Putative uncharacterized protein [Source:UniProtKB/TrEMBL;Acc:B8ADK5] | 3.5 | 565-586 | target 5' GGGGAUCUGAAGUCUGGUCCGG 3' | ::::: :::::.::::::::. | query 3' CCCCU-UACUUCGGACCAGGCU 5' |

| | | | | | | | | | | | | | | |
|---|---|---|---|---|---|---|---|---|---|---|---|---|---|---|
| SI48_S<br>C00 | osa-miR1<br>66b | -1.8831<br>8 | 5416<br>9.96 | 1998<br>28.5 | BGIOSG<br>A005688 | 1.00<br>572 | 80.6<br>1464 | 39.6<br>4585 | Putative uncharacterized protein [Source:UniProtKB/TrEMBL;Acc:B8AI06] | 1 | 2356-<br>2375 | target 5'<br>GGGGAA-GAAGCCU<br>GGUCCGA 3' | ::::::<br>::::::::::::: | query 3'<br>CCCCUUACUUCGGACC<br>AGGCU 5' |
| SI48_S<br>C00 | osa-miR1<br>66b | -1.8831<br>8 | 5416<br>9.96 | 1998<br>28.5 | BGIOSG<br>A008799 | 5.32<br>218 | 39.0<br>0708 | 0 | Putative uncharacterized protein [Source:UniProtKB/TrEMBL;Acc:A2X894] | 3 | 177-1<br>97 | target 5'<br>GGUGAAGGAAGCC<br>UGGUCCGU 3' | :: :::<br>:::::::::::: | query 3'<br>CCCCUUACUUCGGACC<br>AGGCU 5' |
| SI48_S<br>C00 | osa-miR1<br>66b | -1.8831<br>8 | 5416<br>9.96 | 1998<br>28.5 | BGIOSG<br>A002254 | 2.63<br>249 | 5.20<br>0944 | 0 | Putative uncharacterized protein [Source:UniProtKB/TrEMBL;Acc:B8ADK5] | 3.5 | 565-5<br>86 | target 5'<br>GGGGAUCUGAAGU<br>CUGGUCCGG 3' | :::::<br>:::::.::::::::. | query 3'<br>CCCCU-UACUUCGGACC<br>AGGCU 5' |
| SI48_S<br>C00 | osa-miR1<br>66c | -1.8831<br>8 | 5416<br>9.96 | 1998<br>28.5 | BGIOSG<br>A005688 | 1.00<br>572 | 80.6<br>1464 | 39.6<br>4585 | Putative uncharacterized protein [Source:UniProtKB/TrEMBL;Acc:B8AI06] | 1 | 2356-<br>2375 | target 5'<br>GGGGAA-GAAGCCU<br>GGUCCGA 3' | ::::::<br>::::::::::::: | query 3'<br>CCCCUUACUUCGGACC<br>AGGCU 5' |
| SI48_S<br>C00 | osa-miR1<br>66c | -1.8831<br>8 | 5416<br>9.96 | 1998<br>28.5 | BGIOSG<br>A008799 | 5.32<br>218 | 39.0<br>0708 | 0 | Putative uncharacterized protein [Source:UniProtKB/TrEMBL;Acc:A2X894] | 3 | 177-1<br>97 | target 5'<br>GGUGAAGGAAGCC<br>UGGUCCGU 3' | :: :::<br>:::::::::::: | query 3'<br>CCCCUUACUUCGGACC<br>AGGCU 5' |
| SI48_S<br>C00 | osa-miR1<br>66c | -1.8831<br>8 | 5416<br>9.96 | 1998<br>28.5 | BGIOSG<br>A002254 | 2.63<br>249 | 5.20<br>0944 | 0 | Putative uncharacterized protein [Source:UniProtKB/TrEMBL;Acc:B8ADK5] | 3.5 | 565-5<br>86 | target 5'<br>GGGGAUCUGAAGU<br>CUGGUCCGG 3' | :::::<br>:::::.::::::::. | query 3'<br>CCCCU-UACUUCGGACC<br>AGGCU 5' |
| SI48_S<br>C00 | osa-miR1<br>66d | -1.8831<br>8 | 5416<br>9.96 | 1998<br>28.5 | BGIOSG<br>A005688 | 1.00<br>572 | 80.6<br>1464 | 39.6<br>4585 | Putative uncharacterized protein [Source:UniProtKB/TrEMBL;Acc:B8AI06] | 1 | 2356-<br>2375 | target 5'<br>GGGGAA-GAAGCCU<br>GGUCCGA 3' | ::::::<br>::::::::::::: | query 3'<br>CCCCUUACUUCGGACC<br>AGGCU 5' |
| SI48_S<br>C00 | osa-miR1<br>66d | -1.8831<br>8 | 5416<br>9.96 | 1998<br>28.5 | BGIOSG<br>A008799 | 5.32<br>218 | 39.0<br>0708 | 0 | Putative uncharacterized protein [Source:UniProtKB/TrEMBL;Acc:A2X894] | 3 | 177-1<br>97 | target 5'<br>GGUGAAGGAAGCC<br>UGGUCCGU 3' | :: :::<br>:::::::::::: | query 3'<br>CCCCUUACUUCGGACC<br>AGGCU 5' |
| SI48_S<br>C00 | osa-miR1<br>66d | -1.8831<br>8 | 5416<br>9.96 | 1998<br>28.5 | BGIOSG<br>A002254 | 2.63<br>249 | 5.20<br>0944 | 0 | Putative uncharacterized protein [Source:UniProtKB/TrEMBL;Acc:B8ADK5] | 3.5 | 565-5<br>86 | target 5'<br>GGGGAUCUGAAGU<br>CUGGUCCGG 3' | :::::<br>:::::.::::::::. | query 3'<br>CCCCU-UACUUCGGACC<br>AGGCU 5' |
| SI48_S<br>C00 | osa-miR1<br>66e | -1.8605<br>7 | 4018<br>2.69 | 1459<br>26.8 | BGIOSG<br>A005688 | 1.00<br>572 | 80.6<br>1464 | 39.6<br>4585 | Putative uncharacterized protein [Source:UniProtKB/TrEMBL;Acc:B8AI06] | 3 | 2356-<br>2375 | target 5'<br>GGGGAA-GAAGCCU<br>GGUCCGA 3' | ::::::<br>:::::::::: ::: | query 3'<br>CCCCUUACUUCGGACC<br>AAGCU 5' |
| SI48_S<br>C00 | osa-miR1<br>66f | -1.8831<br>8 | 5416<br>9.96 | 1998<br>28.5 | BGIOSG<br>A005688 | 1.00<br>572 | 80.6<br>1464 | 39.6<br>4585 | Putative uncharacterized protein [Source:UniProtKB/TrEMBL;Acc:B8AI06] | 1 | 2356-<br>2375 | target 5'<br>GGGGAA-GAAGCCU<br>GGUCCGA 3' | ::::::<br>::::::::::::: | query 3'<br>CCCCUUACUUCGGACC<br>AGGCU 5' |
| SI48_S<br>C00 | osa-miR1<br>66f | -1.8831<br>8 | 5416<br>9.96 | 1998<br>28.5 | BGIOSG<br>A008799 | 5.32<br>218 | 39.0<br>0708 | 0 | Putative uncharacterized protein [Source:UniProtKB/TrEMBL;Acc:A2X894] | 3 | 177-1<br>97 | target 5'<br>GGUGAAGGAAGCC<br>UGGUCCGU 3' | :: :::<br>:::::::::::: | query 3'<br>CCCCUUACUUCGGACC<br>AGGCU 5' |
| SI48_S<br>C00 | osa-miR1<br>66f | -1.8831<br>8 | 5416<br>9.96 | 1998<br>28.5 | BGIOSG<br>A002254 | 2.63<br>249 | 5.20<br>0944 | 0 | Putative uncharacterized protein [Source:UniProtKB/TrEMBL;Acc:B8ADK5] | 3.5 | 565-5<br>86 | target 5'<br>GGGGAUCUGAAGU<br>CUGGUCCGG 3' | :::::<br>:::::.::::::::. | query 3'<br>CCCCU-UACUUCGGACC<br>AGGCU 5' |
| SI48_S<br>C00 | osa-miR1<br>66g | -1.8622<br>2 | 5235<br>6.98 | 1903<br>55.2 | BGIOSG<br>A005688 | 1.00<br>572 | 80.6<br>1464 | 39.6<br>4585 | Putative uncharacterized protein [Source:UniProtKB/TrEMBL;Acc:B8AI06] | 1.5 | 2356-<br>2375 | target 5'<br>GGGGAA-GAAGCCU<br>GGUCCGA 3' | :.::::<br>::::::::::::: | query 3'<br>CUCCUUACUUCGGACC<br>AGGCU 5' |

| | | | | | | | | | | | | | | |
|---|---|---|---|---|---|---|---|---|---|---|---|---|---|---|
| SI48_SC00 | osa-miR166g | -1.86222 | 52356.98 | 190355.2 | BGIOSGA016985 | 1.45083 | 1.733648 | 0 | Putative uncharacterized protein [Source:UniProtKB/TrEMBL;Acc:B8ATB5] | 3.5 | 788-809 | target 5' CAGGGAUGGAAGCCUGGUCCGU 3' | :::.<br>:: :::::::::::: | query 3' CUCCUUA-CUUCGGACCAGGCU 5' |
| SI48_SC00 | osa-miR166g | -1.86222 | 52356.98 | 190355.2 | BGIOSGA008799 | 5.32218 | 39.00708 | 0 | Putative uncharacterized protein [Source:UniProtKB/TrEMBL;Acc:A2X894] | 3.5 | 177-197 | target 5' GGUGAAGGAAGCCUGGUCCGU 3' | :. :::<br>:::::::::::: | query 3' CUCCUUACUUCGGACCAGGCU 5' |
| SI48_SC00 | osa-miR166g | -1.86222 | 52356.98 | 190355.2 | BGIOSGA002254 | 2.63249 | 5.200944 | 0 | Putative uncharacterized protein [Source:UniProtKB/TrEMBL;Acc:B8ADK5] | 4 | 565-586 | target 5' GGGGAUCUGAAGUCUGGUCCGG 3' | :.:::<br>:::::.::::::::. | query 3' CUCCU-UACUUCGGACCAGGCU 5' |
| SI48_SC00 | osa-miR166h | -1.86222 | 52356.98 | 190355.2 | BGIOSGA005688 | 1.00572 | 80.61464 | 39.64585 | Putative uncharacterized protein [Source:UniProtKB/TrEMBL;Acc:B8AI06] | 1.5 | 2356-2375 | target 5' GGGGAA-GAAGCCUGGUCCGA 3' | :.::::<br>::::::::::::: | query 3' CUCCUUACUUCGGACCAGGCU 5' |
| SI48_SC00 | osa-miR166h | -1.86222 | 52356.98 | 190355.2 | BGIOSGA016985 | 1.45083 | 1.733648 | 0 | Putative uncharacterized protein [Source:UniProtKB/TrEMBL;Acc:B8ATB5] | 3.5 | 788-809 | target 5' CAGGGAUGGAAGCCUGGUCCGU 3' | :::.<br>:: :::::::::::: | query 3' CUCCUUA-CUUCGGACCAGGCU 5' |
| SI48_SC00 | osa-miR166h | -1.86222 | 52356.98 | 190355.2 | BGIOSGA008799 | 5.32218 | 39.00708 | 0 | Putative uncharacterized protein [Source:UniProtKB/TrEMBL;Acc:A2X894] | 3.5 | 177-197 | target 5' GGUGAAGGAAGCCUGGUCCGU 3' | :. :::<br>:::::::::::: | query 3' CUCCUUACUUCGGACCAGGCU 5' |
| SI48_SC00 | osa-miR166h | -1.86222 | 52356.98 | 190355.2 | BGIOSGA002254 | 2.63249 | 5.200944 | 0 | Putative uncharacterized protein [Source:UniProtKB/TrEMBL;Acc:B8ADK5] | 4 | 565-586 | target 5' GGGGAUCUGAAGUCUGGUCCGG 3' | :.:::<br>:::::.::::::::. | query 3' CUCCU-UACUUCGGACCAGGCU 5' |
| SI48_SC00 | osa-miR166i | -1.79826 | 12497.77 | 43469.73 | BGIOSGA005688 | 1.00572 | 80.61464 | 39.64585 | Putative uncharacterized protein [Source:UniProtKB/TrEMBL;Acc:B8AI06] | 2.5 | 2356-2375 | target 5' GGGGAA-GAAGCCUGGUCCGA 3' | :.::::<br>::::::::.::::: | query 3' CUCCUUACUUCGGACUAGGCU 5' |
| SI48_SC00 | osa-miR166j | -1.79826 | 12497.77 | 43469.73 | BGIOSGA005688 | 1.00572 | 80.61464 | 39.64585 | Putative uncharacterized protein [Source:UniProtKB/TrEMBL;Acc:B8AI06] | 2.5 | 2356-2375 | target 5' GGGGAA-GAAGCCUGGUCCGA 3' | :.::::<br>::::::::.::::: | query 3' CUCCUUACUUCGGACUAGGCU 5' |
| SI48_SC00 | osa-miR166k | -1.58673 | 7599.216 | 22827.54 | BGIOSGA016985 | 1.45083 | 1.733648 | 0 | Putative uncharacterized protein [Source:UniProtKB/TrEMBL;Acc:B8ATB5] | 2 | 789-809 | target 5' AGGGAUGGAAGCCUGGUCCGU 3' | ::::::<br>:::::::::::: | query 3' UCCCUAACUUCGGACCAGGCU 5' |
| SI48_SC00 | osa-miR166k | -1.58673 | 7599.216 | 22827.54 | BGIOSGA002254 | 2.63249 | 5.200944 | 0 | Putative uncharacterized protein [Source:UniProtKB/TrEMBL;Acc:B8ADK5] | 3 | 565-586 | target 5' GGGGAUCUGAAGUCUGGUCCGG 3' | .:::::<br>:::::.::::::::. | query 3' UCCCUA-ACUUCGGACCAGGCU 5' |
| SI48_SC00 | osa-miR166k | -1.58673 | 7599.216 | 22827.54 | BGIOSGA005688 | 1.00572 | 80.61464 | 39.64585 | Putative uncharacterized protein [Source:UniProtKB/TrEMBL;Acc:B8AI06] | 3.5 | 2355-2375 | target 5' CGGGGAAGAAGCCUGGUCCGA 3' | :::.<br>::::::::::::: | query 3' UCCCUAACUUCGGACCAGGCU 5' |
| SI48_SC00 | osa-miR166l | -1.58673 | 7599.216 | 22827.54 | BGIOSGA016985 | 1.45083 | 1.733648 | 0 | Putative uncharacterized protein [Source:UniProtKB/TrEMBL;Acc:B8ATB5] | 2 | 789-809 | target 5' AGGGAUGGAAGCCUGGUCCGU 3' | ::::::<br>:::::::::::: | query 3' UCCCUAACUUCGGACCAGGCU 5' |
| SI48_SC00 | osa-miR166l | -1.58673 | 7599.216 | 22827.54 | BGIOSGA002254 | 2.63249 | 5.200944 | 0 | Putative uncharacterized protein [Source:UniProtKB/TrEMBL;Acc:B8ADK5] | 3 | 565-586 | target 5' GGGGAUCUGAAGUCUGGUCCGG 3' | .:::::<br>:::::.::::::::. | query 3' UCCCUA-ACUUCGGACCAGGCU 5' |

| | | | | | | | | | | | | | | |
|---|---|---|---|---|---|---|---|---|---|---|---|---|---|---|
| SI48_SC00 | osa-miR166l | -1.58673 | 7599.216 | 22827.54 | BGIOSGA005688 | 1.00572 | 80.61464 | 39.64585 | Putative uncharacterized protein [Source:UniProtKB/TrEMBL;Acc:B8AI06] | 3.5 | 2355-2375 | target 5' CGGGGAAGAAGCCUGGUCCGA 3' | :::.<br>:::::::::::::: | query 3' UCCCUAACUUCGGACCAGGCU 5' |
| SI48_SC00 | osa-miR166m | -1.8378 | 46839.06 | 167435 | BGIOSGA005688 | 1.00572 | 80.61464 | 39.64585 | Putative uncharacterized protein [Source:UniProtKB/TrEMBL;Acc:B8AI06] | 2.5 | 2355-2375 | target 5' CGGGGAAGAAGCCUGGUCCGA 3' | :::.<br>: ::::::::::::: | query 3' UCCCUUACUUCGGACCAGGCU 5' |
| SI48_SC00 | osa-miR166m | -1.8378 | 46839.06 | 167435 | BGIOSGA016985 | 1.45083 | 1.733648 | 0 | Putative uncharacterized protein [Source:UniProtKB/TrEMBL;Acc:B8ATB5] | 3 | 789-809 | target 5' AGGGAUGGAAGCCUGGUCCGU 3' | :::::<br>::::::::::::: | query 3' UCCCUUACUUCGGACCAGGCU 5' |
| SI48_SC00 | osa-miR166m | -1.8378 | 46839.06 | 167435 | BGIOSGA008799 | 5.32218 | 39.00708 | 0 | Putative uncharacterized protein [Source:UniProtKB/TrEMBL;Acc:A2X894] | 3.5 | 177-197 | target 5' GGUGAAGGAAGCCUGGUCCGU 3' | .: :::<br>::::::::::::: | query 3' UCCCUUACUUCGGACCAGGCU 5' |
| SI48_SC00 | osa-miR166n | -1.88318 | 54169.96 | 199828.5 | BGIOSGA005688 | 1.00572 | 80.61464 | 39.64585 | Putative uncharacterized protein [Source:UniProtKB/TrEMBL;Acc:B8AI06] | 1 | 2356-2375 | target 5' GGGGAA-GAAGCCUGGUCCGA 3' | ::::::<br>:::::::::::::: | query 3' CCCCUUACUUCGGACCAGGCU 5' |
| SI48_SC00 | osa-miR166n | -1.88318 | 54169.96 | 199828.5 | BGIOSGA008799 | 5.32218 | 39.00708 | 0 | Putative uncharacterized protein [Source:UniProtKB/TrEMBL;Acc:A2X894] | 3 | 177-197 | target 5' GGUGAAGGAAGCCUGGUCCGU 3' | :: :::<br>::::::::::::: | query 3' CCCCUUACUUCGGACCAGGCU 5' |
| SI48_SC00 | osa-miR166n | -1.88318 | 54169.96 | 199828.5 | BGIOSGA002254 | 2.63249 | 5.200944 | 0 | Putative uncharacterized protein [Source:UniProtKB/TrEMBL;Acc:B8ADK5] | 3.5 | 565-586 | target 5' GGGGAUCUGAAGUCUGGUCCGG 3' | :::::<br>:::::.::::::::. | query 3' CCCCU-UACUUCGGACCAGGCU 5' |
| SI48_SC00 | osa-miR167a | -1.11069 | 32455.59 | 70088.73 | BGIOSGA031293 | 2.03087 | 201.1032 | 48.45604 | Putative uncharacterized protein [Source:UniProtKB/TrEMBL;Acc:B8BEM7] | 4 | 2389-2409 | target 5' GAGACCCAGCUGGCAGCUUCA 3' | :::<br>: ::::::::::::: | query 3' AUCUAGUACGACCGUCGAAGU 5' |
| SI48_SC00 | osa-miR167a* | -1.00394 | 366.1064 | 735.2192 | BGIOSGA031980 | 2.22828 | 251.379 | 52.86113 | Putative uncharacterized protein [Source:UniProtKB/TrEMBL;Acc:A2Z6Y9] | 4 | 837-857 | target 5' AAAUGAGGGUGUCAUG-AUGAC 3' | ::::::<br>:: ::::::: :::: | query 3' UUUACUCCGACAGUACGUACUA 5' |
| SI48_SC00 | osa-miR167b | -1.11069 | 32455.59 | 70088.73 | BGIOSGA031293 | 2.03087 | 201.1032 | 48.45604 | Putative uncharacterized protein [Source:UniProtKB/TrEMBL;Acc:B8BEM7] | 4 | 2389-2409 | target 5' GAGACCCAGCUGGCAGCUUCA 3' | :::<br>: ::::::::::::: | query 3' AUCUAGUACGACCGUCGAAGU 5' |
| SI48_SC00 | osa-miR167c | -1.11069 | 32455.59 | 70088.73 | BGIOSGA031293 | 2.03087 | 201.1032 | 48.45604 | Putative uncharacterized protein [Source:UniProtKB/TrEMBL;Acc:B8BEM7] | 4 | 2389-2409 | target 5' GAGACCCAGCUGGCAGCUUCA 3' | :::<br>: ::::::::::::: | query 3' AUCUAGUACGACCGUCGAAGU 5' |
| SI48_SC00 | osa-miR167d | -1.10609 | 32163.45 | 69236.53 | BGIOSGA019025 | 1.45083 | 1.733648 | 0 | Putative uncharacterized protein [Source:UniProtKB/TrEMBL;Acc:B8AWX6] | 3.5 | 911-930 | target 5' CAGAUGGUGCUGGCAG-UUCA 3' | :::::<br>.::::::::: :::: | query 3' GUCUAGUACGACCGUCGAAGU 5' |
| SI48_SC00 | osa-miR167d | -1.10609 | 32163.45 | 69236.53 | BGIOSGA000020 | 4.55688 | 22.53743 | 0 | Putative uncharacterized protein [Source:UniProtKB/TrEMBL;Acc:B8A9X0] | 4 | 1057-1078 | target 5' CAGAUCAAUGCUGGUGGUUUCA 3' | ::::::<br>:::::::..:.:::: | query 3' GUCUAG-UACGACCGUCGAAGU 5' |
| SI48_SC00 | osa-miR167d | -1.10609 | 32163.45 | 69236.53 | BGIOSGA031293 | 2.03087 | 201.1032 | 48.45604 | Putative uncharacterized protein [Source:UniProtKB/TrEMBL;Acc:B8BEM7] | 4 | 2389-2409 | target 5' GAGACCCAGCUGGCAGCUUCA 3' | :::<br>: ::::::::::::: | query 3' GUCUAGUACGACCGUCGAAGU 5' |

| | | | | | | | | | | | | | | |
|---|---|---|---|---|---|---|---|---|---|---|---|---|---|---|
| SI48_SC00 | osa-miR167e | -1.10609 | 32163.45 | 69236.53 | BGIOSGA019025 | 1.45083 | 1.733648 | 0 | Putative uncharacterized protein [Source:UniProtKB/TrEMBL;Acc:B8AWX6] | 3.5 | 911-930 | target 5' CAGAUGGUGCUGGCAG-UUCA 3' | ::::: .:::::::: :::: | query 3' GUCUAGUACGACCGUCGAAGU 5' |
| SI48_SC00 | osa-miR167e | -1.10609 | 32163.45 | 69236.53 | BGIOSGA000020 | 4.55688 | 22.53743 | 0 | Putative uncharacterized protein [Source:UniProtKB/TrEMBL;Acc:B8A9X0] | 4 | 1057-1078 | target 5' CAGAUCAAUGCUGGUGGUUUCA 3' | :::::: :::::::..:.:::: | query 3' GUCUAG-UACGACCGUCGAAGU 5' |
| SI48_SC00 | osa-miR167e | -1.10609 | 32163.45 | 69236.53 | BGIOSGA031293 | 2.03087 | 201.1032 | 48.45604 | Putative uncharacterized protein [Source:UniProtKB/TrEMBL;Acc:B8BEM7] | 4 | 2389-2409 | target 5' GAGACCCAGCUGGCAGCUUCA 3' | ::: : :::::::::::: | query 3' GUCUAGUACGACCGUCGAAGU 5' |
| SI48_SC00 | osa-miR167f | -1.10609 | 32163.45 | 69236.53 | BGIOSGA019025 | 1.45083 | 1.733648 | 0 | Putative uncharacterized protein [Source:UniProtKB/TrEMBL;Acc:B8AWX6] | 3.5 | 911-930 | target 5' CAGAUGGUGCUGGCAG-UUCA 3' | ::::: .:::::::: :::: | query 3' GUCUAGUACGACCGUCGAAGU 5' |
| SI48_SC00 | osa-miR167f | -1.10609 | 32163.45 | 69236.53 | BGIOSGA000020 | 4.55688 | 22.53743 | 0 | Putative uncharacterized protein [Source:UniProtKB/TrEMBL;Acc:B8A9X0] | 4 | 1057-1078 | target 5' CAGAUCAAUGCUGGUGGUUUCA 3' | :::::: :::::::..:.:::: | query 3' GUCUAG-UACGACCGUCGAAGU 5' |
| SI48_SC00 | osa-miR167f | -1.10609 | 32163.45 | 69236.53 | BGIOSGA031293 | 2.03087 | 201.1032 | 48.45604 | Putative uncharacterized protein [Source:UniProtKB/TrEMBL;Acc:B8BEM7] | 4 | 2389-2409 | target 5' GAGACCCAGCUGGCAGCUUCA 3' | ::: : :::::::::::: | query 3' GUCUAGUACGACCGUCGAAGU 5' |
| SI48_SC00 | osa-miR167g | -1.10609 | 32163.45 | 69236.53 | BGIOSGA019025 | 1.45083 | 1.733648 | 0 | Putative uncharacterized protein [Source:UniProtKB/TrEMBL;Acc:B8AWX6] | 3.5 | 911-930 | target 5' CAGAUGGUGCUGGCAG-UUCA 3' | ::::: .:::::::: :::: | query 3' GUCUAGUACGACCGUCGAAGU 5' |
| SI48_SC00 | osa-miR167g | -1.10609 | 32163.45 | 69236.53 | BGIOSGA000020 | 4.55688 | 22.53743 | 0 | Putative uncharacterized protein [Source:UniProtKB/TrEMBL;Acc:B8A9X0] | 4 | 1057-1078 | target 5' CAGAUCAAUGCUGGUGGUUUCA 3' | :::::: :::::::..:.:::: | query 3' GUCUAG-UACGACCGUCGAAGU 5' |
| SI48_SC00 | osa-miR167g | -1.10609 | 32163.45 | 69236.53 | BGIOSGA031293 | 2.03087 | 201.1032 | 48.45604 | Putative uncharacterized protein [Source:UniProtKB/TrEMBL;Acc:B8BEM7] | 4 | 2389-2409 | target 5' GAGACCCAGCUGGCAGCUUCA 3' | ::: : :::::::::::: | query 3' GUCUAGUACGACCGUCGAAGU 5' |
| SI48_SC00 | osa-miR167h | -1.10609 | 32163.45 | 69236.53 | BGIOSGA019025 | 1.45083 | 1.733648 | 0 | Putative uncharacterized protein [Source:UniProtKB/TrEMBL;Acc:B8AWX6] | 3.5 | 911-930 | target 5' CAGAUGGUGCUGGCAG-UUCA 3' | ::::: .:::::::: :::: | query 3' GUCUAGUACGACCGUCGAAGU 5' |
| SI48_SC00 | osa-miR167h | -1.10609 | 32163.45 | 69236.53 | BGIOSGA000020 | 4.55688 | 22.53743 | 0 | Putative uncharacterized protein [Source:UniProtKB/TrEMBL;Acc:B8A9X0] | 4 | 1057-1078 | target 5' CAGAUCAAUGCUGGUGGUUUCA 3' | :::::: :::::::..:.:::: | query 3' GUCUAG-UACGACCGUCGAAGU 5' |
| SI48_SC00 | osa-miR167h | -1.10609 | 32163.45 | 69236.53 | BGIOSGA031293 | 2.03087 | 201.1032 | 48.45604 | Putative uncharacterized protein [Source:UniProtKB/TrEMBL;Acc:B8BEM7] | 4 | 2389-2409 | target 5' GAGACCCAGCUGGCAGCUUCA 3' | ::: : :::::::::::: | query 3' GUCUAGUACGACCGUCGAAGU 5' |
| SI48_SC00 | osa-miR167i | -1.10609 | 32163.45 | 69236.53 | BGIOSGA019025 | 1.45083 | 1.733648 | 0 | Putative uncharacterized protein [Source:UniProtKB/TrEMBL;Acc:B8AWX6] | 3.5 | 911-930 | target 5' CAGAUGGUGCUGGCAG-UUCA 3' | ::::: .:::::::: :::: | query 3' GUCUAGUACGACCGUCGAAGU 5' |
| SI48_SC00 | osa-miR167i | -1.10609 | 32163.45 | 69236.53 | BGIOSGA000020 | 4.55688 | 22.53743 | 0 | Putative uncharacterized protein [Source:UniProtKB/TrEMBL;Acc:B8A9X0] | 4 | 1057-1078 | target 5' CAGAUCAAUGCUGGUGGUUUCA 3' | :::::: :::::::..:.:::: | query 3' GUCUAG-UACGACCGUCGAAGU 5' |

| | | | | | | | | | | | | | | | |
|---|---|---|---|---|---|---|---|---|---|---|---|---|---|---|---|
| SI48_SC00 | osa-miR167i | -1.10609 | 32163.45 | 69236.53 | BGIOSGA031293 | 2.03087 | 201.1032 | 48.45604 | Putative uncharacterized protein [Source:UniProtKB/TrEMBL;Acc:B8BEM7] | 4 | 2389-2409 | target 5' GAGACCCAGCUGGCAGCUUCA 3' | :::<br>: :::::::::::: | query 3' GUCUAGUACGACCGUCGAAGU 5' |
| SI48_SC00 | osa-miR167j | -1.10609 | 32163.45 | 69236.53 | BGIOSGA019025 | 1.45083 | 1.733648 | 0 | Putative uncharacterized protein [Source:UniProtKB/TrEMBL;Acc:B8AWX6] | 3.5 | 911-930 | target 5' CAGAUGGUGCUGGCAG-UUCA 3' | :::::<br>.::::::::: :::: | query 3' GUCUAGUACGACCGUCGAAGU 5' |
| SI48_SC00 | osa-miR167j | -1.10609 | 32163.45 | 69236.53 | BGIOSGA000020 | 4.55688 | 22.53743 | 0 | Putative uncharacterized protein [Source:UniProtKB/TrEMBL;Acc:B8A9X0] | 4 | 1057-1078 | target 5' CAGAUCAAUGCUGGUGGUUUCA 3' | ::::::<br>::::::::..:.:::: | query 3' GUCUAG-UACGACCGUCGAAGU 5' |
| SI48_SC00 | osa-miR167j | -1.10609 | 32163.45 | 69236.53 | BGIOSGA031293 | 2.03087 | 201.1032 | 48.45604 | Putative uncharacterized protein [Source:UniProtKB/TrEMBL;Acc:B8BEM7] | 4 | 2389-2409 | target 5' GAGACCCAGCUGGCAGCUUCA 3' | :::<br>: :::::::::::: | query 3' GUCUAGUACGACCGUCGAAGU 5' |
| SI48_SC00 | osa-miR1850.1 | 5.22832 | 341.0307 | 8.123969 | BGIOSGA012292 | -1.28061 | 447.2812 | 1088.058 | Putative uncharacterized protein [Source:UniProtKB/TrEMBL;Acc:A2XEV9] | 4 | 472-493 | target 5' CACCAUUUCUCCCAACUUUUCA 3' | : :::<br>::::::::::::.:: | query 3' GGGGU-UAGAGGGUUGAAAGGU 5' |
| SI48_SC00 | osa-miR1861d | -2.24966 | 8.776524 | 45.49423 | BGIOSGA029503 | 2.1763 | 43.3412 | 8.810189 | Putative uncharacterized protein [Source:UniProtKB/TrEMBL;Acc:B8BD43] | 3.5 | 209-231 | target 5' CUCCAGCUUCUGCCUCAAGGCCA 3' | :: :::<br>:.::::::::::.::: | query 3' GA-GUCAAGGACGGAGUUCUGGU 5' |
| SI48_SC00 | osa-miR1861d | -2.24966 | 8.776524 | 45.49423 | BGIOSGA017432 | 2.63249 | 5.200944 | 0 | Putative uncharacterized protein [Source:UniProtKB/TrEMBL;Acc:B8AXI3] | 4 | 491-512 | target 5' CCCAGUUUCUUCCUCAAGACCG 3' | : ::::<br>:.:: ::::::::::. | query 3' GAGUCAAGGACGGAGUUCUGGU 5' |
| SI48_SC00 | osa-miR1861h | -2.10057 | 10.03031 | 46.30662 | BGIOSGA017432 | 2.63249 | 5.200944 | 0 | Putative uncharacterized protein [Source:UniProtKB/TrEMBL;Acc:B8AXI3] | 3.5 | 491-512 | target 5' CCCAGUUUCUUCCUCAAGACCG 3' | : ::::<br>:.:: :::::::::: | query 3' GAGUCAAGGACGGAGUUCUGGC 5' |
| SI48_SC00 | osa-miR1861j | -2.10057 | 10.03031 | 46.30662 | BGIOSGA017432 | 2.63249 | 5.200944 | 0 | Putative uncharacterized protein [Source:UniProtKB/TrEMBL;Acc:B8AXI3] | 3.5 | 491-512 | target 5' CCCAGUUUCUUCCUCAAGACCG 3' | : ::::<br>:.:: :::::::::: | query 3' GAGUCAAGGACGGAGUUCUGGC 5' |
| SI48_SC00 | osa-miR2871a-3p | -1.37455 | 30.09094 | 79.6149 | BGIOSGA013087 | 2.98816 | 6.934593 | 0 | Putative uncharacterized protein [Source:UniProtKB/TrEMBL;Acc:B8AM58] | 4 | 539-558 | target 5' AUGACC-UAGAAAGUAAAAUA 3' | ::::<br>: :::::: ::::::: | query 3' CACUGGUAUCUUUGAUUUUAU 5' |
| SI48_SC00 | osa-miR2871b | -1.37455 | 30.09094 | 79.6149 | BGIOSGA013087 | 2.98816 | 6.934593 | 0 | Putative uncharacterized protein [Source:UniProtKB/TrEMBL;Acc:B8AM58] | 4 | 539-558 | target 5' AUGACC-UAGAAAGUAAAAUA 3' | ::::<br>: :::::: ::::::: | query 3' CACUGGUAUCUUUGAUUUUAU 5' |
| SI48_SC00 | osa-miR396a | -1.02944 | 531.6066 | 1086.175 | BGIOSGA002892 | 2.06864 | 428.2111 | 101.3172 | Putative uncharacterized protein [Source:UniProtKB/TrEMBL;Acc:A2WLE4] | 4 | 1681-1701 | target 5' GAGUUGCAGAAGGCUGUGGAA 3' | ::::<br>::::.:::::::::: | query 3' GUCAAGUUCUUUCGACACCUU 5' |
| SI48_SC00 | osa-miR396b | -1.02944 | 531.6066 | 1086.175 | BGIOSGA002892 | 2.06864 | 428.2111 | 101.3172 | Putative uncharacterized protein [Source:UniProtKB/TrEMBL;Acc:A2WLE4] | 4 | 1681-1701 | target 5' GAGUUGCAGAAGGCUGUGGAA 3' | ::::<br>::::.:::::::::: | query 3' GUCAAGUUCUUUCGACACCUU 5' |
| SI48_SC00 | osa-miR396c | -1.04705 | 535.368 | 1107.297 | BGIOSGA002892 | 2.06864 | 428.2111 | 101.3172 | Putative uncharacterized protein [Source:UniProtKB/TrEMBL;Acc:A2WLE4] | 3.5 | 1681-1701 | target 5' GAGUUGCAGAAGGCUGUGGAA 3' | .::::<br>::::.:::::::::: | query 3' UUCAAGUUCUUUCGACACCUU 5' |

| | | | | | | | | | | | | | | |
|---|---|---|---|---|---|---|---|---|---|---|---|---|---|---|
| SI48_SC00 | osa-miR396c | -1.04705 | 535.368 | 1107.297 | BGIOSGA034898 | 3.8076 | 13.00236 | 0 | Putative uncharacterized protein [Source:UniProtKB/TrEMBL;Acc:A2ZBY7] | 4 | 1281-1300 | target 5' AAGUUCAAGAAAGC-AUGGAA 3' | :::::: :::::::: ::::: | query 3' UUCAAGUUCUUUCGACACCUU 5' |
| SI48_SC00 | osa-miR396c | -1.04705 | 535.368 | 1107.297 | BGIOSGA006677 | 2.72816 | 49.40897 | 6.607641 | Putative uncharacterized protein [Source:UniProtKB/TrEMBL;Acc:A2X3J9] | 4 | 602-621 | target 5' AGAUUCAGGAAAGCUG-GGAA 3' | :. ::: :.:::::::: :::: | query 3' UUCAAGUUCUUUCGACACCUU 5' |
| SI48_SC00 | osa-miR396c | -1.04705 | 535.368 | 1107.297 | BGIOSGA004420 | 2.41525 | 4.33412 | 0 | Putative uncharacterized protein [Source:UniProtKB/TrEMBL;Acc:A2WUV3] | 4 | 1247-1266 | target 5' AAGUAAAAG-AAGCUGUGGAA 3' | :::: ::: :::::::::: | query 3' UUCAAGUUCUUUCGACACCUU 5' |
| SI48_SC00 | osa-miR396d | -1.04132 | 11531.1 | 23733.36 | BGIOSGA034580 | 4.88778 | 28.60519 | 0 | Putative uncharacterized protein [Source:UniProtKB/TrEMBL;Acc:A2ZB08] | 4 | 966-985 | target 5' CA-UUGAAGAAAGCCUCUGGA 3' | :: :: :::::::::: :::: | query 3' GUCAAGUUCUUUCGGACACCU 5' |
| SI48_SC00 | osa-miR396e | -1.04132 | 11531.1 | 23733.36 | BGIOSGA034580 | 4.88778 | 28.60519 | 0 | Putative uncharacterized protein [Source:UniProtKB/TrEMBL;Acc:A2ZB08] | 4 | 966-985 | target 5' CA-UUGAAGAAAGCCUCUGGA 3' | :: :: :::::::::: :::: | query 3' GUCAAGUUCUUUCGGACACCU 5' |
| SI48_SC00 | osa-miR444b.1 | -1.06925 | 687.0765 | 1442.817 | BGIOSGA018711 | 2.58406 | 18.20331 | 2.202547 | Putative uncharacterized protein [Source:UniProtKB/TrEMBL;Acc:B8AYM0] | 4 | 214-234 | target 5' GGCGGCAAGCUUGCGACAGCG 3' | :::.:: ::::::: ::::.:. | query 3' CCGUCGUUCGAACUCUGUUGU 5' |
| SI48_SC00 | osa-miR444c.1 | -1.06925 | 687.0765 | 1442.817 | BGIOSGA018711 | 2.58406 | 18.20331 | 2.202547 | Putative uncharacterized protein [Source:UniProtKB/TrEMBL;Acc:B8AYM0] | 4 | 214-234 | target 5' GGCGGCAAGCUUGCGACAGCG 3' | :::.:: ::::::: ::::.:. | query 3' CCGUCGUUCGAACUCUGUUGU 5' |
| SI48_SC00 | osa-miR5083 | -2.23874 | 6.268946 | 33.30827 | BGIOSGA007106 | 2.12256 | 22.53743 | 4.405094 | Putative uncharacterized protein [Source:UniProtKB/TrEMBL;Acc:A2X127] | 3 | 1947-1965 | target 5' UGAUCAGAUAGUUGUA-UCU 3' | :::::: ::::.:::: ::: | query 3' ACUAGUCUAUUAACAUCAGA 5' |
| SI48_SC00 | osa-miR5161 | -1.37919 | 2.507578 | 8.123969 | BGIOSGA012773 | 1.33583 | 18.20331 | 6.607641 | Putative uncharacterized protein [Source:UniProtKB/TrEMBL;Acc:B8AQL0] | 4 | 2560-2579 | target 5' UAUAUUCCUGCUGAUCCAGG 3' | ::::.: ::. :::::::::. | query 3' AUAUGAGGGAGACUAGGUCU 5' |
| SI48_SC00 | osa-miR5161 | -1.37919 | 2.507578 | 8.123969 | BGIOSGA036829 | 1.78062 | 25.1379 | 6.607641 | Putative uncharacterized protein [Source:UniProtKB/TrEMBL;Acc:B8BLR2] | 4 | 1807-1826 | target 5' CAUACUCCCUGUGGUCCAGA 3' | :::: ::::: ::.::::: | query 3' AUAUGAGGGAGACUAGGUCU 5' |
| SI48_SC00 | osa-miR528 | 2.66422 | 6617.499 | 1043.118 | BGIOSGA009946 | -1.49063 | 26.00472 | 74.8866 | Putative uncharacterized protein [Source:UniProtKB/TrEMBL;Acc:A2XL83] | 4 | 61-80 | target 5' CUCCUCUGCGUGCUCC-UCCA 3' | :::::: :::.:::.:: :::: | query 3' GAGGAGACGUACGGGGAAGGU 5' |
| SI48_SC00 | osa-miR529b | 2.43308 | 61.43567 | 10.56116 | BGIOSGA026705 | -1.23065 | 6.934593 | 17.62038 | Putative uncharacterized protein [Source:UniProtKB/TrEMBL;Acc:A2YWW1] | 1 | 813-833 | target 5' GAGCUGUGCUCUCUCUCUUCU 3' | .::::: :.:::::::::::: | query 3' UUCGACAUGAGAGAGAGAAGA 5' |
| SI48_SC00 | osa-miR529b | 2.43308 | 61.43567 | 10.56116 | BGIOSGA029541 | -1.8357 | 6.067769 | 24.22802 | Putative uncharacterized protein [Source:UniProtKB/TrEMBL;Acc:B8BD46] | 1.5 | 573-593 | target 5' CAGCUGUGCUCUCUCUCUUCU 3' | :::: ::.:::::::::::: | query 3' UUCGACAUGAGAGAGAGAAGA 5' |
| SI48_SC00 | osa-miR529b | 2.43308 | 61.43567 | 10.56116 | BGIOSGA007643 | -2.65427 | 15.60283 | 103.5197 | Putative uncharacterized protein [Source:UniProtKB/TrEMBL;Acc:A2X1G6] | 2.5 | 712-731 | target 5' CAGC-GUGCUCUCUCUCUUCU 3' | ::: ::.:::::::::::: | query 3' UUCGACAUGAGAGAGAGAAGA 5' |

| | | | | | | | | | | | | | | |
|---|---|---|---|---|---|---|---|---|---|---|---|---|---|---|
| SI48_S C00 | osa-miR5 35 | 1.0477 | 2960 .196 | 1431 .443 | BGIOSG A007643 | -2.6 5427 | 15.6 0283 | 103. 5197 | Putative uncharacterized protein [Source:UniProtKB/TrEMBL;Acc:A2X1G6] | 4 | 714-7 35 | target 5' GCGUGCUCUCUCU CUUCUGUCA 3' | :::::: <br> :::::::: : ::::: | query 3' CGCACGAGAGAGAGCA -ACAGU 5' |
| SI48_S I24 | osa-miR1 318 | 1.34547 | 94.0 3419 | 36.3 9839 | BGIOSG A018749 | -1.0 4144 | 240. 9771 | 497. 0585 | Putative uncharacterized protein [Source:UniProtKB/TrEMBL;Acc:A2Y0R2] | 4 | 1060- 1079 | target 5' GUCGAUCUCAUUU CUCUUGA 3' | :::: : <br> ::::.::::.::: | query 3' CAGCCACAGUAGAGAG GACU 5' |
| SI48_S I24 | osa-miR1 320 | 1.90623 | 75.2 2735 | 19.3 3665 | BGIOSG A021582 | -2.5 2806 | 0 | 4.76 7947 | Putative uncharacterized protein [Source:UniProtKB/TrEMBL;Acc:B8B4F2] | 4 | 633-6 52 | target 5' CU-UUACCUUCCUC CGUUCCA 3' | :: : : <br> ::::::::::::: | query 3' GAUAUUUUAAGGAGG CAAGGU 5' |
| SI48_S I24 | osa-miR1 425-3p | -1.1744 7 | 531. 6066 | 1201 .147 | BGIOSG A029652 | 2.00 12 | 36.4 0661 | 8.34 3908 | Putative uncharacterized protein [Source:UniProtKB/TrEMBL;Acc:B8BCJ5] | 2.5 | 774-7 93 | target 5' AUCAGGA-CCAGUU CUUGCUG 3' | :: :.: <br> : ::::::::::::: | query 3' UAAUUCUAGGUCAAGA ACGAC 5' |
| SI48_S I24 | osa-miR1 425-3p | -1.1744 7 | 531. 6066 | 1201 .147 | BGIOSG A027518 | 1.30 673 | 2168 .794 | 876. 1103 | Putative uncharacterized protein [Source:UniProtKB/TrEMBL;Acc:A2YS08] | 3 | 331-3 50 | target 5' AUUAA-AUCCAGUU GUUGCUG 3' | ::::: <br> :::::::: :::::: | query 3' UAAUUCUAGGUCAAGA ACGAC 5' |
| SI48_S I24 | osa-miR1 432 | 1.08147 | 147. 9471 | 69.3 8443 | BGIOSG A018749 | -1.0 4144 | 240. 9771 | 497. 0585 | Putative uncharacterized protein [Source:UniProtKB/TrEMBL;Acc:A2Y0R2] | 4 | 1060- 1080 | target 5' GUCGAUCUCAUUU CUCUUGAU 3' | :::: : <br> ::::.::::.:::: | query 3' CAGCCACAGUAGAGAG GACUA 5' |
| SI48_S I24 | osa-miR1 59a.1 | 1.91906 | 1419 89.1 | 3754 4.94 | BGIOSG A002134 | -1.1 3224 | 0 | 1.19 1987 | Putative uncharacterized protein [Source:UniProtKB/TrEMBL;Acc:B8AAM9] | 3.5 | 452-4 72 | target 5' CGGAGCCCCCUUCA AACCAAA 3' | :.:::: <br> :::::::: ::::: | query 3' GUCUCGAGGGAAGUUA GGUUU 5' |
| SI48_S I24 | osa-miR1 59b | 1.91906 | 1419 89.1 | 3754 4.94 | BGIOSG A002134 | -1.1 3224 | 0 | 1.19 1987 | Putative uncharacterized protein [Source:UniProtKB/TrEMBL;Acc:B8AAM9] | 3.5 | 452-4 72 | target 5' CGGAGCCCCCUUCA AACCAAA 3' | :.:::: <br> :::::::: ::::: | query 3' GUCUCGAGGGAAGUUA GGUUU 5' |
| SI48_S I24 | osa-miR1 59d | 1.96584 | 1402 47.6 | 3590 1.33 | BGIOSG A002134 | -1.1 3224 | 0 | 1.19 1987 | Putative uncharacterized protein [Source:UniProtKB/TrEMBL;Acc:B8AAM9] | 4 | 452-4 72 | target 5' CGGAGCCCCCUUCA AACCAAA 3' | :::::: <br> :::::::: :::: | query 3' GCCUCGAGGGAAGUUA GGUUA 5' |
| SI48_S I24 | osa-miR1 62a | -1.6303 7 | 514. 0536 | 1593 .567 | BGIOSG A011556 | 1.75 781 | 22.5 3743 | 5.95 9934 | Putative uncharacterized protein [Source:UniProtKB/TrEMBL;Acc:A2XBW0] | 2 | 2977- 2998 | target 5' CUGGAUGCAGAGG UUUUAUCGA 3' | :::::: <br> ::::::: :::::::: | query 3' GACCUACGUCUCC-AA AUAGCU 5' |
| SI48_S I24 | osa-miR1 62b | -1.6212 6 | 510. 2922 | 1571 .956 | BGIOSG A011556 | 1.75 781 | 22.5 3743 | 5.95 9934 | Putative uncharacterized protein [Source:UniProtKB/TrEMBL;Acc:A2XBW0] | 3 | 2977- 2998 | target 5' CUGGAUGCAGAGG UUUUAUCGA 3' | :::::: <br> ::::::: .::::::: | query 3' GACCUACGUCUCC-GA AUAGCU 5' |
| SI48_S I24 | osa-miR1 66a | -1.7684 5 | 5416 9.96 | 1845 52.4 | BGIOSG A005688 | 1.01 678 | 80.6 1464 | 39.3 3557 | Putative uncharacterized protein [Source:UniProtKB/TrEMBL;Acc:B8AI06] | 1 | 2356- 2375 | target 5' GGGGAA-GAAGCCU GGUCCGA 3' | :::::: <br> :::::::::::::: | query 3' CCCCUUACUUCGGACC AGGCU 5' |
| SI48_S I24 | osa-miR1 66a | -1.7684 5 | 5416 9.96 | 1845 52.4 | BGIOSG A008799 | 2.29 504 | 39.0 0708 | 7.15 1921 | Putative uncharacterized protein [Source:UniProtKB/TrEMBL;Acc:A2X894] | 3 | 177-1 97 | target 5' GGUGAAGGAAGCC UGGUCCGU 3' | :: ::: <br> ::::::::::::: | query 3' CCCCUUACUUCGGACC AGGCU 5' |
| SI48_S I24 | osa-miR1 66b | -1.7684 5 | 5416 9.96 | 1845 52.4 | BGIOSG A005688 | 1.01 678 | 80.6 1464 | 39.3 3557 | Putative uncharacterized protein [Source:UniProtKB/TrEMBL;Acc:B8AI06] | 1 | 2356- 2375 | target 5' GGGGAA-GAAGCCU GGUCCGA 3' | :::::: <br> :::::::::::::: | query 3' CCCCUUACUUCGGACC AGGCU 5' |

| | | | | | | | | | | | | | | |
|---|---|---|---|---|---|---|---|---|---|---|---|---|---|---|
| SI48_SI24 | osa-miR166b | -1.76845 | 54169.96 | 184552.4 | BGIOSGA008799 | 2.29504 | 39.00708 | 7.151921 | Putative uncharacterized protein [Source:UniProtKB/TrEMBL;Acc:A2X894] | 3 | 177-197 | target 5'<br>GGUGAAGGAAGCCUGGUCCGU 3' | :: :::<br>:::::::::::: | query 3'<br>CCCCUUACUUCGGACCAGGCU 5' |
| SI48_SI24 | osa-miR166c | -1.76845 | 54169.96 | 184552.4 | BGIOSGA005688 | 1.01678 | 80.61464 | 39.33557 | Putative uncharacterized protein [Source:UniProtKB/TrEMBL;Acc:B8AI06] | 1 | 2356-2375 | target 5'<br>GGGGAA-GAAGCCUGGUCCGA 3' | ::::::<br>::::::::::::: | query 3'<br>CCCCUUACUUCGGACCAGGCU 5' |
| SI48_SI24 | osa-miR166c | -1.76845 | 54169.96 | 184552.4 | BGIOSGA008799 | 2.29504 | 39.00708 | 7.151921 | Putative uncharacterized protein [Source:UniProtKB/TrEMBL;Acc:A2X894] | 3 | 177-197 | target 5'<br>GGUGAAGGAAGCCUGGUCCGU 3' | :: :::<br>:::::::::::: | query 3'<br>CCCCUUACUUCGGACCAGGCU 5' |
| SI48_SI24 | osa-miR166d | -1.76845 | 54169.96 | 184552.4 | BGIOSGA005688 | 1.01678 | 80.61464 | 39.33557 | Putative uncharacterized protein [Source:UniProtKB/TrEMBL;Acc:B8AI06] | 1 | 2356-2375 | target 5'<br>GGGGAA-GAAGCCUGGUCCGA 3' | ::::::<br>::::::::::::: | query 3'<br>CCCCUUACUUCGGACCAGGCU 5' |
| SI48_SI24 | osa-miR166d | -1.76845 | 54169.96 | 184552.4 | BGIOSGA008799 | 2.29504 | 39.00708 | 7.151921 | Putative uncharacterized protein [Source:UniProtKB/TrEMBL;Acc:A2X894] | 3 | 177-197 | target 5'<br>GGUGAAGGAAGCCUGGUCCGU 3' | :: :::<br>:::::::::::: | query 3'<br>CCCCUUACUUCGGACCAGGCU 5' |
| SI48_SI24 | osa-miR166e | -1.70778 | 40182.69 | 131261.7 | BGIOSGA005688 | 1.01678 | 80.61464 | 39.33557 | Putative uncharacterized protein [Source:UniProtKB/TrEMBL;Acc:B8AI06] | 3 | 2356-2375 | target 5'<br>GGGGAA-GAAGCCUGGUCCGA 3' | ::::::<br>:::::::::: ::: | query 3'<br>CCCCUUACUUCGGACCAAGCU 5' |
| SI48_SI24 | osa-miR166f | -1.76845 | 54169.96 | 184552.4 | BGIOSGA005688 | 1.01678 | 80.61464 | 39.33557 | Putative uncharacterized protein [Source:UniProtKB/TrEMBL;Acc:B8AI06] | 1 | 2356-2375 | target 5'<br>GGGGAA-GAAGCCUGGUCCGA 3' | ::::::<br>::::::::::::: | query 3'<br>CCCCUUACUUCGGACCAGGCU 5' |
| SI48_SI24 | osa-miR166f | -1.76845 | 54169.96 | 184552.4 | BGIOSGA008799 | 2.29504 | 39.00708 | 7.151921 | Putative uncharacterized protein [Source:UniProtKB/TrEMBL;Acc:A2X894] | 3 | 177-197 | target 5'<br>GGUGAAGGAAGCCUGGUCCGU 3' | :: :::<br>:::::::::::: | query 3'<br>CCCCUUACUUCGGACCAGGCU 5' |
| SI48_SI24 | osa-miR166g | -1.75681 | 52356.98 | 176942.8 | BGIOSGA005688 | 1.01678 | 80.61464 | 39.33557 | Putative uncharacterized protein [Source:UniProtKB/TrEMBL;Acc:B8AI06] | 1.5 | 2356-2375 | target 5'<br>GGGGAA-GAAGCCUGGUCCGA 3' | :.::::<br>::::::::::::: | query 3'<br>CUCCUUACUUCGGACCAGGCU 5' |
| SI48_SI24 | osa-miR166g | -1.75681 | 52356.98 | 176942.8 | BGIOSGA008799 | 2.29504 | 39.00708 | 7.151921 | Putative uncharacterized protein [Source:UniProtKB/TrEMBL;Acc:A2X894] | 3.5 | 177-197 | target 5'<br>GGUGAAGGAAGCCUGGUCCGU 3' | :. :::<br>:::::::::::: | query 3'<br>CUCCUUACUUCGGACCAGGCU 5' |
| SI48_SI24 | osa-miR166h | -1.75681 | 52356.98 | 176942.8 | BGIOSGA005688 | 1.01678 | 80.61464 | 39.33557 | Putative uncharacterized protein [Source:UniProtKB/TrEMBL;Acc:B8AI06] | 1.5 | 2356-2375 | target 5'<br>GGGGAA-GAAGCCUGGUCCGA 3' | :.::::<br>::::::::::::: | query 3'<br>CUCCUUACUUCGGACCAGGCU 5' |
| SI48_SI24 | osa-miR166h | -1.75681 | 52356.98 | 176942.8 | BGIOSGA008799 | 2.29504 | 39.00708 | 7.151921 | Putative uncharacterized protein [Source:UniProtKB/TrEMBL;Acc:A2X894] | 3.5 | 177-197 | target 5'<br>GGUGAAGGAAGCCUGGUCCGU 3' | :. :::<br>:::::::::::: | query 3'<br>CUCCUUACUUCGGACCAGGCU 5' |
| SI48_SI24 | osa-miR166i | -1.83817 | 12497.77 | 44689.26 | BGIOSGA005688 | 1.01678 | 80.61464 | 39.33557 | Putative uncharacterized protein [Source:UniProtKB/TrEMBL;Acc:B8AI06] | 2.5 | 2356-2375 | target 5'<br>GGGGAA-GAAGCCUGGUCCGA 3' | :.::::<br>::::::::.::::: | query 3'<br>CUCCUUACUUCGGACUAGGCU 5' |
| SI48_SI24 | osa-miR166i | -1.83817 | 12497.77 | 44689.26 | BGIOSGA014593 | 1.94274 | 30.33884 | 7.151921 | Putative uncharacterized protein [Source:UniProtKB/TrEMBL;Acc:B8ASY0] | 4 | 22-Feb | target 5'<br>UGGGGAGGAAGCCUGAUCCGU 3' | .::.<br>: :::::::::::: | query 3'<br>CUCCUUACUUCGGACUAGGCU 5' |

| | | | | | | | | | | | | | | |
|---|---|---|---|---|---|---|---|---|---|---|---|---|---|---|
| SI48_SI24 | osa-miR166j | -1.83817 | 12497.77 | 44689.26 | BGIOSGA005688 | 1.01678 | 80.61464 | 39.33557 | Putative uncharacterized protein [Source:UniProtKB/TrEMBL;Acc:B8AI06] | 2.5 | 2356-2375 | target 5' GGGGAA-GAAGCCU GGUCCGA 3' | :.:::: ::::::::.::::: | query 3' CUCCUUACUUCGGACU AGGCU 5' |
| SI48_SI24 | osa-miR166j | -1.83817 | 12497.77 | 44689.26 | BGIOSGA014593 | 1.94274 | 30.33884 | 7.151921 | Putative uncharacterized protein [Source:UniProtKB/TrEMBL;Acc:B8ASY0] | 4 | 22-Feb | target 5' UGGGGAGGAAGCC UGAUCCGU 3' | .::. : :::::::::::: | query 3' CUCCUUACUUCGGACU AGGCU 5' |
| SI48_SI24 | osa-miR166k | -1.42928 | 7599.216 | 20467.27 | BGIOSGA005688 | 1.01678 | 80.61464 | 39.33557 | Putative uncharacterized protein [Source:UniProtKB/TrEMBL;Acc:B8AI06] | 3.5 | 2355-2375 | target 5' CGGGGAAGAAGCC UGGUCCGA 3' | :::. ::::::::::::: | query 3' UCCCUAACUUCGGACC AGGCU 5' |
| SI48_SI24 | osa-miR166l | -1.42928 | 7599.216 | 20467.27 | BGIOSGA005688 | 1.01678 | 80.61464 | 39.33557 | Putative uncharacterized protein [Source:UniProtKB/TrEMBL;Acc:B8AI06] | 3.5 | 2355-2375 | target 5' CGGGGAAGAAGCC UGGUCCGA 3' | :::. ::::::::::::: | query 3' UCCCUAACUUCGGACC AGGCU 5' |
| SI48_SI24 | osa-miR166m | -1.68542 | 46839.06 | 150652.9 | BGIOSGA005688 | 1.01678 | 80.61464 | 39.33557 | Putative uncharacterized protein [Source:UniProtKB/TrEMBL;Acc:B8AI06] | 2.5 | 2355-2375 | target 5' CGGGGAAGAAGCC UGGUCCGA 3' | :::. : ::::::::::::: | query 3' UCCCUUACUUCGGACC AGGCU 5' |
| SI48_SI24 | osa-miR166m | -1.68542 | 46839.06 | 150652.9 | BGIOSGA008799 | 2.29504 | 39.00708 | 7.151921 | Putative uncharacterized protein [Source:UniProtKB/TrEMBL;Acc:A2X894] | 3.5 | 177-197 | target 5' GGUGAAGGAAGCC UGGUCCGU 3' | .: ::: :::::::::::: | query 3' UCCCUUACUUCGGACC AGGCU 5' |
| SI48_SI24 | osa-miR166n | -1.76845 | 54169.96 | 184552.4 | BGIOSGA005688 | 1.01678 | 80.61464 | 39.33557 | Putative uncharacterized protein [Source:UniProtKB/TrEMBL;Acc:B8AI06] | 1 | 2356-2375 | target 5' GGGGAA-GAAGCCU GGUCCGA 3' | :::::: ::::::::::::: | query 3' CCCCUUACUUCGGACC AGGCU 5' |
| SI48_SI24 | osa-miR166n | -1.76845 | 54169.96 | 184552.4 | BGIOSGA008799 | 2.29504 | 39.00708 | 7.151921 | Putative uncharacterized protein [Source:UniProtKB/TrEMBL;Acc:A2X894] | 3 | 177-197 | target 5' GGUGAAGGAAGCC UGGUCCGU 3' | :: ::: :::::::::::: | query 3' CCCCUUACUUCGGACC AGGCU 5' |
| SI48_SI24 | osa-miR167a | -1.54867 | 32455.59 | 94949.76 | BGIOSGA024307 | 1.22944 | 6.934593 | 2.383974 | Putative uncharacterized protein [Source:UniProtKB/TrEMBL;Acc:B8B612] | 4 | 2765-2785 | target 5' UAGAUCAUGCUGA CAGCCUCA 3' | :::::: :::::: :::: ::: | query 3' AUCUAGUACGACCGUC GAAGU 5' |
| SI48_SI24 | osa-miR167a | -1.54867 | 32455.59 | 94949.76 | BGIOSGA014119 | 1.85928 | 15.60283 | 3.575961 | Auxin response factor 12 [Source:UniProtKB/Swiss-Prot;Acc:Q258Y5] | 4 | 2334-2355 | target 5' UAGAUCAGGCUGG CAGCUUGUA 3' | :::::: : ::::::::::: .: | query 3' AUCUAGUACGACCGUC GAA-GU 5' |
| SI48_SI24 | osa-miR167a | -1.54867 | 32455.59 | 94949.76 | BGIOSGA031293 | 1.57677 | 201.1032 | 66.75126 | Putative uncharacterized protein [Source:UniProtKB/TrEMBL;Acc:B8BEM7] | 4 | 2389-2409 | target 5' GAGACCCAGCUGG CAGCUUCA 3' | ::: : :::::::::::: | query 3' AUCUAGUACGACCGUC GAAGU 5' |
| SI48_SI24 | osa-miR167b | -1.54867 | 32455.59 | 94949.76 | BGIOSGA024307 | 1.22944 | 6.934593 | 2.383974 | Putative uncharacterized protein [Source:UniProtKB/TrEMBL;Acc:B8B612] | 4 | 2765-2785 | target 5' UAGAUCAUGCUGA CAGCCUCA 3' | :::::: :::::: :::: ::: | query 3' AUCUAGUACGACCGUC GAAGU 5' |
| SI48_SI24 | osa-miR167b | -1.54867 | 32455.59 | 94949.76 | BGIOSGA014119 | 1.85928 | 15.60283 | 3.575961 | Auxin response factor 12 [Source:UniProtKB/Swiss-Prot;Acc:Q258Y5] | 4 | 2334-2355 | target 5' UAGAUCAGGCUGG CAGCUUGUA 3' | :::::: : ::::::::::: .: | query 3' AUCUAGUACGACCGUC GAA-GU 5' |
| SI48_SI24 | osa-miR167b | -1.54867 | 32455.59 | 94949.76 | BGIOSGA031293 | 1.57677 | 201.1032 | 66.75126 | Putative uncharacterized protein [Source:UniProtKB/TrEMBL;Acc:B8BEM7] | 4 | 2389-2409 | target 5' GAGACCCAGCUGG CAGCUUCA 3' | ::: : :::::::::::: | query 3' AUCUAGUACGACCGUC GAAGU 5' |
| SI48_SI24 | osa-miR167c | -1.54867 | 32455.59 | 94949.76 | BGIOSGA024307 | 1.22944 | 6.934593 | 2.383974 | Putative uncharacterized protein [Source:UniProtKB/TrEMBL | 4 | 2765-2785 | target 5' UAGAUCAUGCUGA CAGCCUCA 3' | :::::: :::::: :::: ::: | query 3' AUCUAGUACGACCGUC GAAGU 5' |

| | | | | | | | | | | | | | | |
|---|---|---|---|---|---|---|---|---|---|---|---|---|---|---|
| | | | | | | | | | BL;Acc:B8B612] | | | | | |
| SI48_SI24 | osa-miR167c | -1.54867 | 32455.59 | 94949.76 | BGIOSGA014119 | 1.85928 | 15.60283 | 3.575961 | Auxin response factor 12 [Source:UniProtKB/Swiss-Prot;Acc:Q258Y5] | 4 | 2334-2355 | target 5' UAGAUCAGGCUGGCAGCUUGUA 3' | ::::::<br>: ::::::::::: .: | query 3' AUCUAGUACGACCGUCGAA-GU 5' |
| SI48_SI24 | osa-miR167c | -1.54867 | 32455.59 | 94949.76 | BGIOSGA031293 | 1.57677 | 201.1032 | 66.75126 | Putative uncharacterized protein [Source:UniProtKB/TrEMBL;Acc:B8BEM7] | 4 | 2389-2409 | target 5' GAGACCCAGCUGGCAGCUUCA 3' | :::<br>: ::::::::::::: | query 3' AUCUAGUACGACCGUCGAAGU 5' |
| SI48_SI24 | osa-miR167d | -1.54805 | 32163.45 | 94054.58 | BGIOSGA000020 | 2.02883 | 22.53743 | 4.767947 | Putative uncharacterized protein [Source:UniProtKB/TrEMBL;Acc:B8A9X0] | 4 | 1057-1078 | target 5' CAGAUCAAUGCUGGUGGUUUCA 3' | ::::::<br>:::::::..:.:::: | query 3' GUCUAG-UACGACCGUCGAAGU 5' |
| SI48_SI24 | osa-miR167d | -1.54805 | 32163.45 | 94054.58 | BGIOSGA031293 | 1.57677 | 201.1032 | 66.75126 | Putative uncharacterized protein [Source:UniProtKB/TrEMBL;Acc:B8BEM7] | 4 | 2389-2409 | target 5' GAGACCCAGCUGGCAGCUUCA 3' | :::<br>: ::::::::::::: | query 3' GUCUAGUACGACCGUCGAAGU 5' |
| SI48_SI24 | osa-miR167e | -1.54805 | 32163.45 | 94054.58 | BGIOSGA000020 | 2.02883 | 22.53743 | 4.767947 | Putative uncharacterized protein [Source:UniProtKB/TrEMBL;Acc:B8A9X0] | 4 | 1057-1078 | target 5' CAGAUCAAUGCUGGUGGUUUCA 3' | ::::::<br>:::::::..:.:::: | query 3' GUCUAG-UACGACCGUCGAAGU 5' |
| SI48_SI24 | osa-miR167e | -1.54805 | 32163.45 | 94054.58 | BGIOSGA031293 | 1.57677 | 201.1032 | 66.75126 | Putative uncharacterized protein [Source:UniProtKB/TrEMBL;Acc:B8BEM7] | 4 | 2389-2409 | target 5' GAGACCCAGCUGGCAGCUUCA 3' | :::<br>: ::::::::::::: | query 3' GUCUAGUACGACCGUCGAAGU 5' |
| SI48_SI24 | osa-miR167f | -1.54805 | 32163.45 | 94054.58 | BGIOSGA000020 | 2.02883 | 22.53743 | 4.767947 | Putative uncharacterized protein [Source:UniProtKB/TrEMBL;Acc:B8A9X0] | 4 | 1057-1078 | target 5' CAGAUCAAUGCUGGUGGUUUCA 3' | ::::::<br>:::::::..:.:::: | query 3' GUCUAG-UACGACCGUCGAAGU 5' |
| SI48_SI24 | osa-miR167f | -1.54805 | 32163.45 | 94054.58 | BGIOSGA031293 | 1.57677 | 201.1032 | 66.75126 | Putative uncharacterized protein [Source:UniProtKB/TrEMBL;Acc:B8BEM7] | 4 | 2389-2409 | target 5' GAGACCCAGCUGGCAGCUUCA 3' | :::<br>: ::::::::::::: | query 3' GUCUAGUACGACCGUCGAAGU 5' |
| SI48_SI24 | osa-miR167g | -1.54805 | 32163.45 | 94054.58 | BGIOSGA000020 | 2.02883 | 22.53743 | 4.767947 | Putative uncharacterized protein [Source:UniProtKB/TrEMBL;Acc:B8A9X0] | 4 | 1057-1078 | target 5' CAGAUCAAUGCUGGUGGUUUCA 3' | ::::::<br>:::::::..:.:::: | query 3' GUCUAG-UACGACCGUCGAAGU 5' |
| SI48_SI24 | osa-miR167g | -1.54805 | 32163.45 | 94054.58 | BGIOSGA031293 | 1.57677 | 201.1032 | 66.75126 | Putative uncharacterized protein [Source:UniProtKB/TrEMBL;Acc:B8BEM7] | 4 | 2389-2409 | target 5' GAGACCCAGCUGGCAGCUUCA 3' | :::<br>: ::::::::::::: | query 3' GUCUAGUACGACCGUCGAAGU 5' |
| SI48_SI24 | osa-miR167h | -1.54805 | 32163.45 | 94054.58 | BGIOSGA000020 | 2.02883 | 22.53743 | 4.767947 | Putative uncharacterized protein [Source:UniProtKB/TrEMBL;Acc:B8A9X0] | 4 | 1057-1078 | target 5' CAGAUCAAUGCUGGUGGUUUCA 3' | ::::::<br>:::::::..:.:::: | query 3' GUCUAG-UACGACCGUCGAAGU 5' |
| SI48_SI24 | osa-miR167h | -1.54805 | 32163.45 | 94054.58 | BGIOSGA031293 | 1.57677 | 201.1032 | 66.75126 | Putative uncharacterized protein [Source:UniProtKB/TrEMBL;Acc:B8BEM7] | 4 | 2389-2409 | target 5' GAGACCCAGCUGGCAGCUUCA 3' | :::<br>: ::::::::::::: | query 3' GUCUAGUACGACCGUCGAAGU 5' |
| SI48_SI24 | osa-miR167i | -1.54805 | 32163.45 | 94054.58 | BGIOSGA000020 | 2.02883 | 22.53743 | 4.767947 | Putative uncharacterized protein [Source:UniProtKB/TrEMBL;Acc:B8A9X0] | 4 | 1057-1078 | target 5' CAGAUCAAUGCUGGUGGUUUCA 3' | ::::::<br>:::::::..:.:::: | query 3' GUCUAG-UACGACCGUCGAAGU 5' |
| SI48_SI24 | osa-miR167i | -1.54805 | 32163.45 | 94054.58 | BGIOSGA031293 | 1.57677 | 201.1032 | 66.75126 | Putative uncharacterized protein [Source:UniProtKB/TrEMBL;Acc:B8BEM7] | 4 | 2389-2409 | target 5' GAGACCCAGCUGGCAGCUUCA 3' | :::<br>: ::::::::::::: | query 3' GUCUAGUACGACCGUCGAAGU 5' |

| | | | | | | | | | | | | | | |
|---|---|---|---|---|---|---|---|---|---|---|---|---|---|---|
| SI48_SI24 | osa-miR167j | -1.54805 | 32163.45 | 94054.58 | BGIOSGA000020 | 2.02883 | 22.53743 | 4.767947 | Putative uncharacterized protein [Source:UniProtKB/TrEMBL;Acc:B8A9X0] | 4 | 1057-1078 | target 5' CAGAUCAAUGCUGGUGGUUUCA 3' | ::::::<br>::::::::..:.:::: | query 3' GUCUAG-UACGACCGUCGAAGU 5' |
| SI48_SI24 | osa-miR167j | -1.54805 | 32163.45 | 94054.58 | BGIOSGA031293 | 1.57677 | 201.1032 | 66.75126 | Putative uncharacterized protein [Source:UniProtKB/TrEMBL;Acc:B8BEM7] | 4 | 2389-2409 | target 5' GAGACCCAGCUGGCAGCUUCA 3' | :::<br>: :::::::::::: | query 3' GUCUAGUACGACCGUCGAAGU 5' |
| SI48_SI24 | osa-miR169f.2 | 2.97608 | 105.3183 | 12.51195 | BGIOSGA015823 | -1.13224 | 0 | 1.191987 | Putative uncharacterized protein [Source:UniProtKB/TrEMBL;Acc:B8AUM1] | 4 | 356-375 | target 5' CCGACGC-GCUCUUGUCCUCC 3' | ::::<br>: ::::::::::: | query 3' GGCUUAGUCGAGAACAGGAGU 5' |
| SI48_SI24 | osa-miR1860-3p | -1.38237 | 33.85231 | 89.85853 | BGIOSGA025231 | 2.1594 | 3.467296 | 0 | Putative uncharacterized protein [Source:UniProtKB/TrEMBL;Acc:B8B7N6] | 4 | 3022-3043 | target 5' GAAGAUGACCUAGCUUCCAGAG 3' | . :::<br>.:::::::::::::: | query 3' UCUCUUUUGGAUCGAAGGUCUA 5' |
| SI48_SI24 | osa-miR1860-5p | -1.6449 | 8.776524 | 29.57369 | BGIOSGA036396 | 1.45083 | 1.733648 | 0 | Putative uncharacterized protein [Source:UniProtKB/TrEMBL;Acc:B8BP23] | 2.5 | 697-717 | target 5' AGGUCUGGAAGCUUGUUUUCU 3' | ::.:::<br>::::::: ::::::: | query 3' UCUAGACCUUCGACCAAAAGA 5' |
| SI48_SI24 | osa-miR1860-5p | -1.6449 | 8.776524 | 29.57369 | BGIOSGA031384 | 1.36084 | 19.93695 | 7.151921 | Putative uncharacterized protein [Source:UniProtKB/TrEMBL;Acc:B8BIA4] | 3.5 | 163-182 | target 5' AGG-GUGAAAGCUGGUUUUCU 3' | ::.<br>:: :::::::::::: | query 3' UCUAGACCUUCGACCAAAAGA 5' |
| SI48_SI24 | osa-miR2871a-3p | -1.13136 | 30.09094 | 67.10954 | BGIOSGA013087 | 2.98816 | 6.934593 | 0 | Putative uncharacterized protein [Source:UniProtKB/TrEMBL;Acc:B8AM58] | 4 | 539-558 | target 5' AUGACC-UAGAAAGUAAAAUA 3' | ::::<br>: :::::: ::::::: | query 3' CACUGGUAUCUUUGAUUUUAU 5' |
| SI48_SI24 | osa-miR2871b | -1.13136 | 30.09094 | 67.10954 | BGIOSGA013087 | 2.98816 | 6.934593 | 0 | Putative uncharacterized protein [Source:UniProtKB/TrEMBL;Acc:B8AM58] | 4 | 539-558 | target 5' AUGACC-UAGAAAGUAAAAUA 3' | ::::<br>: :::::: ::::::: | query 3' CACUGGUAUCUUUGAUUUUAU 5' |
| SI48_SI24 | osa-miR390 | -1.00617 | 135.4092 | 272.9879 | BGIOSGA003637 | 1.45083 | 1.733648 | 0 | Putative uncharacterized protein [Source:UniProtKB/TrEMBL;Acc:B8A9B8] | 4 | 1276-1296 | target 5' GGCGGUAUACCUUCUGAGCUU 3' | :::: :<br>:: :::.:::::::: | query 3' CCGCGAUAGGGAGGACUCGAA 5' |
| SI48_SI24 | osa-miR394 | -1.73071 | 106.5721 | 356.0218 | BGIOSGA025510 | 1.02622 | 15.60283 | 7.151921 | Putative uncharacterized protein [Source:UniProtKB/TrEMBL;Acc:B8B516] | 4 | 20-38 | target 5' GGCGGCGGA-AGAAUGCCAA 3' | :: ::<br>::: :::::::::: | query 3' CCUCCACCUGUCUUACGGUU 5' |
| SI48_SI24 | osa-miR396d | -1.27009 | 11531.1 | 27811.78 | BGIOSGA036704 | 1.02716 | 3.467296 | 1.191987 | Putative uncharacterized protein [Source:UniProtKB/TrEMBL;Acc:A2ZHQ2] | 4 | 966-985 | target 5' CA-UUGAAGAAAGCCUCUGGA 3' | :: ::<br>:::::::::: :::: | query 3' GUCAAGUUCUUUCGGACACCU 5' |
| SI48_SI24 | osa-miR396d | -1.27009 | 11531.1 | 27811.78 | BGIOSGA034580 | 4.88778 | 28.60519 | 0 | Putative uncharacterized protein [Source:UniProtKB/TrEMBL;Acc:A2ZB08] | 4 | 966-985 | target 5' CA-UUGAAGAAAGCCUCUGGA 3' | :: ::<br>:::::::::: :::: | query 3' GUCAAGUUCUUUCGGACACCU 5' |
| SI48_SI24 | osa-miR396e | -1.27009 | 11531.1 | 27811.78 | BGIOSGA036704 | 1.02716 | 3.467296 | 1.191987 | Putative uncharacterized protein [Source:UniProtKB/TrEMBL;Acc:A2ZHQ2] | 4 | 966-985 | target 5' CA-UUGAAGAAAGCCUCUGGA 3' | :: ::<br>:::::::::: :::: | query 3' GUCAAGUUCUUUCGGACACCU 5' |
| SI48_SI24 | osa-miR396e | -1.27009 | 11531.1 | 27811.78 | BGIOSGA034580 | 4.88778 | 28.60519 | 0 | Putative uncharacterized protein [Source:UniProtKB/TrEMBL;Acc:A2ZB08] | 4 | 966-985 | target 5' CA-UUGAAGAAAGCCUCUGGA 3' | :: ::<br>:::::::::: :::: | query 3' GUCAAGUUCUUUCGGACACCU 5' |

| | | | | | | | | | | | | | | |
|---|---|---|---|---|---|---|---|---|---|---|---|---|---|---|
| SI48_SI24 | osa-miR399d | -1.05293 | 493.9929 | 1025.98 | BGIOSGA017239 | 1.35023 | 110.9535 | 42.91153 | Putative uncharacterized protein [Source:UniProtKB/TrEMBL;Acc:B8AV88] | 3 | 3888-3907 | target 5' UA-GCCAGCUCUCC UUUGGCA 3' | .: : :<br>:.:::::::::::: | query 3' GUCCCGUUGAGAGGAA ACCGU 5' |
| SI48_SI24 | osa-miR399d | -1.05293 | 493.9929 | 1025.98 | BGIOSGA035900 | 2.9007 | 24.27107 | 2.383974 | Putative uncharacterized protein [Source:UniProtKB/TrEMBL;Acc:B8BMT6] | 4 | 2138-2157 | target 5' CA-GGCGGCUCUCA UUUGGCA 3' | :: :::<br>..::::: ::::::: | query 3' GUCCCGUUGAGAGGAA ACCGU 5' |
| SI48_SI24 | osa-miR399i | -1.09439 | 329.7466 | 705.2188 | BGIOSGA017239 | 1.35023 | 110.9535 | 42.91153 | Putative uncharacterized protein [Source:UniProtKB/TrEMBL;Acc:B8AV88] | 2.5 | 3888-3907 | target 5' UA-GCCAGCUCUCC UUUGGCA 3' | .: : :<br>:::::::::::::: | query 3' GUCCCGUCGAGAGGAA ACCGU 5' |
| SI48_SI24 | osa-miR399i | -1.09439 | 329.7466 | 705.2188 | BGIOSGA035900 | 2.9007 | 24.27107 | 2.383974 | Putative uncharacterized protein [Source:UniProtKB/TrEMBL;Acc:B8BMT6] | 3.5 | 2138-2157 | target 5' CA-GGCGGCUCUCA UUUGGCA 3' | :: :::<br>.:::::: ::::::: | query 3' GUCCCGUCGAGAGGAA ACCGU 5' |
| SI48_SI24 | osa-miR399j | -1.0515 | 453.8717 | 941.8084 | BGIOSGA017239 | 1.35023 | 110.9535 | 42.91153 | Putative uncharacterized protein [Source:UniProtKB/TrEMBL;Acc:B8AV88] | 2.5 | 3888-3907 | target 5' UA-GCCAGCUCUCC UUUGGCA 3' | :: : :<br>:.:::::::::::: | query 3' AUCCCGUUGAGAGGAA ACCGU 5' |
| SI48_SI24 | osa-miR435 | -1.0733 | 117.8562 | 249.1015 | BGIOSGA023934 | 2.68779 | 20.80378 | 2.383974 | Putative uncharacterized protein [Source:UniProtKB/TrEMBL;Acc:A2YNH7] | 3.5 | 1091-1111 | target 5' UCAACUUCAACUA CCGGAUGA 3' | ::::::<br>.::: ::::::::.: | query 3' AGUUGAGGUU-AUGGC CUAUU 5' |
| SI48_SI24 | osa-miR435 | -1.0733 | 117.8562 | 249.1015 | BGIOSGA016338 | 1.02716 | 3.467296 | 1.191987 | Putative uncharacterized protein [Source:UniProtKB/TrEMBL;Acc:B8ATH9] | 3.5 | 25-Jul | target 5' UUAACUGCAAUAC CGG-UAA 3' | :.::::<br>:::::::: ::: | query 3' AGUUGAGGUUAUGGCC UAUU 5' |
| SI48_SI24 | osa-miR5144-5p | -1.49307 | 541.6369 | 1526.458 | BGIOSGA030251 | 1.85819 | 11.26871 | 2.383974 | Putative uncharacterized protein [Source:UniProtKB/TrEMBL;Acc:A2YYB8] | 3 | 693-713 | target 5' GUCUCUUCGGCAG CCCAAGAA 3' | ::::::<br>::.::::: ::::: | query 3' CAGAGAAGUCGUCGUG UUCUU 5' |
| SI48_SI24 | osa-miR5144-5p | -1.49307 | 541.6369 | 1526.458 | BGIOSGA024088 | 2.48467 | 11.26871 | 1.191987 | F-box protein; MM724 [Source:UniProtKB/TrEMBL;Acc:A2YMM3] | 4 | 52-73 | target 5' UUCUCUUGCAGCA GCACGAGGA 3' | ::::<br>:: :::::::::.::.: | query 3' CAGAGAA-GUCGUCGU GUUCUU 5' |
| SI48_SI24 | osa-miR5144-5p | -1.49307 | 541.6369 | 1526.458 | BGIOSGA035800 | 1.06254 | 6.067769 | 2.383974 | Putative uncharacterized protein [Source:UniProtKB/TrEMBL;Acc:A2ZMY0] | 4 | 1231-1251 | target 5' GUCUGUUCAGCAG CGCAUGAA 3' | :::: :<br>::::::::.:: ::: | query 3' CAGAGAAGUCGUCGUG UUCUU 5' |
| SI48_SI24 | osa-miR5160 | -1.40298 | 21.31442 | 58.00994 | BGIOSGA009387 | 2.93483 | 207.171 | 26.22371 | Putative uncharacterized protein [Source:UniProtKB/TrEMBL;Acc:B8AP67] | 3.5 | 357-377 | target 5' UAGAAA-AGACCAU CGAUUUCG 3' | .:::::<br>: :::::::::.::: | query 3' GUCUUUAUAUGGUAGC UAGAGC 5' |
| SI48_SI24 | osa-miR5161 | -2.27055 | 2.507578 | 15.9243 | BGIOSGA012773 | 1.73522 | 18.20331 | 4.767947 | Putative uncharacterized protein [Source:UniProtKB/TrEMBL;Acc:B8AQL0] | 4 | 2560-2579 | target 5' UAUAUUCCUGCUG AUCCAGG 3' | ::::.:<br>::. :::::::::. | query 3' AUAUGAGGGAGACUA GGUCU 5' |
| SI48_SI24 | osa-miR5161 | -2.27055 | 2.507578 | 15.9243 | BGIOSGA036828 | 1.7064 | 37.27344 | 10.72788 | Putative uncharacterized protein [Source:UniProtKB/TrEMBL;Acc:B8BLQ7] | 4 | 1690-1709 | target 5' CAUACUCCCUGUG GUCCAGA 3' | ::::<br>::::: ::.:::::: | query 3' AUAUGAGGGAGACUA GGUCU 5' |
| SI48_SI24 | osa-miR5161 | -2.27055 | 2.507578 | 15.9243 | BGIOSGA036829 | 1.909 | 25.1379 | 5.959934 | Putative uncharacterized protein [Source:UniProtKB/TrEMBL;Acc:B8BLR2] | 4 | 1807-1826 | target 5' CAUACUCCCUGUG GUCCAGA 3' | ::::<br>::::: ::.:::::: | query 3' AUAUGAGGGAGACUA GGUCU 5' |
| SI48_SI24 | osa-miR530-3p | 2.20261 | 542.8907 | 117.1573 | BGIOSGA001350 | -3.69152 | 0 | 11.91987 | Putative uncharacterized protein | 4 | 32-52 | target 5' CUUGCACCUGCCUC | ::::<br>: ::::::::: ::: | query 3' CAACGUAGACGGAGAC |

| | | | | | | | | | | | | | | |
|---|---|---|---|---|---|---|---|---|---|---|---|---|---|---|
| | | | | | | | | | [Source:UniProtKB/TrEMBL;Acc:A2WRS1] | | | UGCUCCU 3' | | GUGGA 5' |
| SI48_SI24 | osa-miR5512 | -1.07512 | 35.1061 | 75.07168 | BGIOSGA014297 | 1.45083 | 1.733648 | 0 | Putative uncharacterized protein [Source:UniProtKB/TrEMBL;Acc:B8AUU5] | 4 | 1300-1319 | target 5' GAUGAG-AUUACCAUAUCCUA 3' | : :: ::::::::::::: | query 3' AAAAUCGUAAUGGUAUAGGAU 5' |
| SI48_SI24 | osa-miR811d.1 | -1.70872 | 85.25767 | 280.9501 | BGIOSGA038081 | 1.02716 | 3.467296 | 1.191987 | | 4 | 270-290 | target 5' GGGACAACCGAGAUUCGUCCU 3' | :::: : :::::::.::::: | query 3' ACCUGUAGGCUCUAGGCAGGU 5' |
| SI48_SI24 | osa-miR820a | -1.64741 | 50.15157 | 159.243 | BGIOSGA011693 | 1.11452 | 269.5823 | 123.9666 | Putative uncharacterized protein [Source:UniProtKB/TrEMBL;Acc:B8ALG0] | 2.5 | 344-364 | target 5' CUGGUCCGUCCACGAGGCUGC 3' | :::::: :.::::::::::.: | query 3' GACCAGGUAGGUGCUCCGGCU 5' |
| SI48_SI24 | osa-miR820b | -1.64741 | 50.15157 | 159.243 | BGIOSGA011693 | 1.11452 | 269.5823 | 123.9666 | Putative uncharacterized protein [Source:UniProtKB/TrEMBL;Acc:B8ALG0] | 2.5 | 344-364 | target 5' CUGGUCCGUCCACGAGGCUGC 3' | :::::: :.::::::::::.: | query 3' GACCAGGUAGGUGCUCCGGCU 5' |
| SI48_SI24 | osa-miR820c | -1.64741 | 50.15157 | 159.243 | BGIOSGA011693 | 1.11452 | 269.5823 | 123.9666 | Putative uncharacterized protein [Source:UniProtKB/TrEMBL;Acc:B8ALG0] | 2.5 | 344-364 | target 5' CUGGUCCGUCCACGAGGCUGC 3' | :::::: :.::::::::::.: | query 3' GACCAGGUAGGUGCUCCGGCU 5' |
